# Visible‐Light‐Mediated Decarboxylative Radical Additions to Vinyl Boronic Esters: Rapid Access to γ‐Amino Boronic Esters

**DOI:** 10.1002/anie.201712186

**Published:** 2018-01-26

**Authors:** Adam Noble, Riccardo S. Mega, Daniel Pflästerer, Eddie L. Myers, Varinder K. Aggarwal

**Affiliations:** ^1^ School of Chemistry University of Bristol Cantock's Close Bristol BS8 1TS UK

**Keywords:** amino acids, boron, photochemistry, radical reactions, reaction mechanisms

## Abstract

The synthesis of alkyl boronic esters by direct decarboxylative radical addition of carboxylic acids to vinyl boronic esters is described. The reaction proceeds under mild photoredox catalysis and involves an unprecedented single‐electron reduction of an α‐boryl radical intermediate to the corresponding anion. The reaction is amenable to a diverse range of substrates, including α‐amino, α‐oxy, and alkyl carboxylic acids, thus providing a novel method to rapidly access boron‐containing molecules of potential biological importance.

Boronic acids and their derivatives occupy a central position in the chemical sciences,^1^ with broad applications in synthesis,^2^ polymer chemistry,^3^ and as chemical receptors.^4^ They also play an increasingly important role in medicinal chemistry since they can act as carboxylic acid bioisosteres.^5^ For example, peptidyl boronic acids can function as inhibitors of serine proteases, an attribute that has led to the commercialization of the antineoplastic drug, bortezomib, for the treatment of relapsed and refractory multiple myeloma (Scheme 1 a).^6^ Whilst boronic acid analogues of α‐amino acids are now well established, there have been very few investigations into the use of boron analogues of other biologically important amino acids, such as the neuromodulator γ‐amino butyric acid (GABA).^7^, ^8^, ^9^ The paucity of such investigations could be due to the lack of availability of these molecules, therefore we decided to address this by developing new methodologies.

**Scheme 1:**
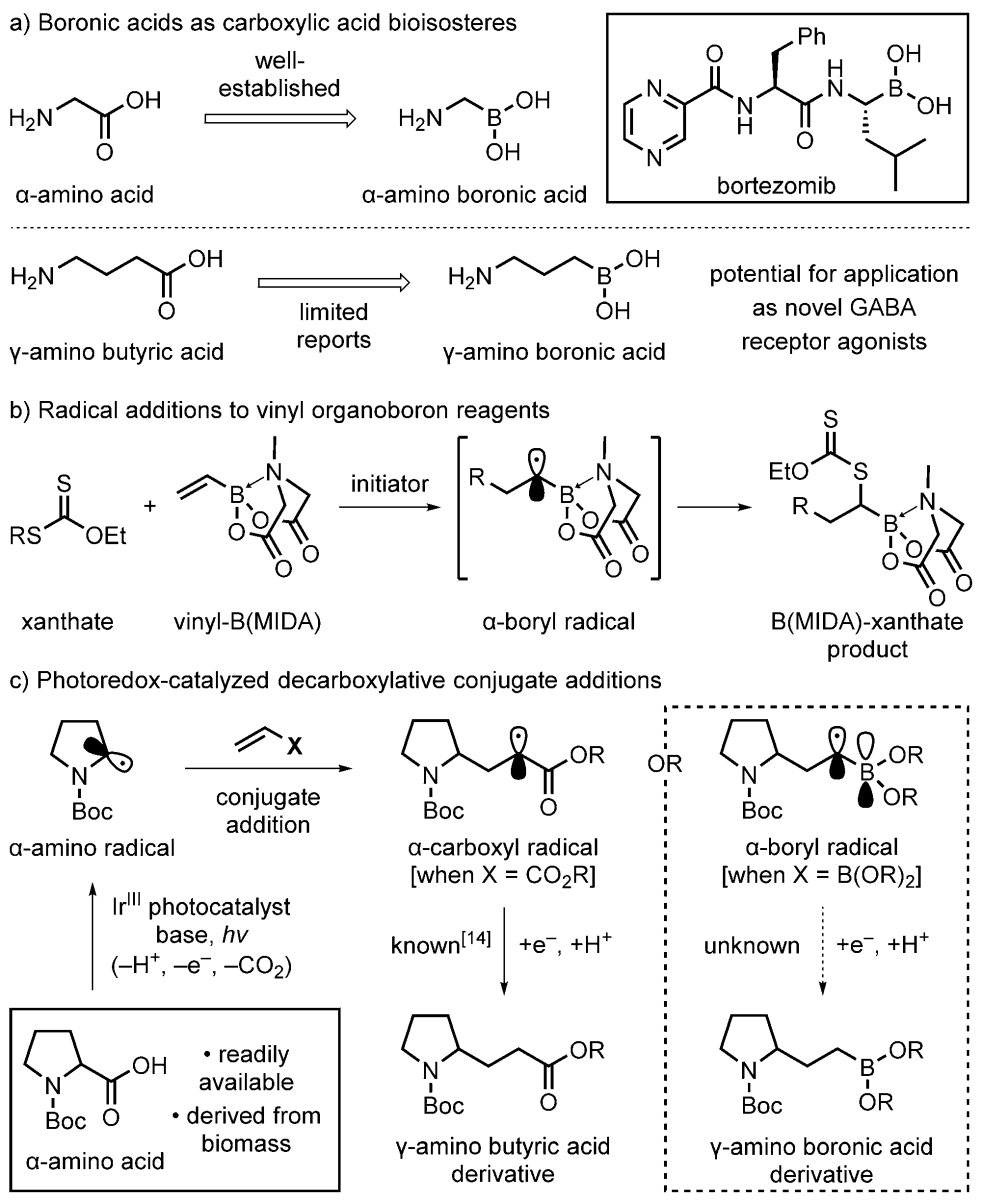
Boronic acids as carboxylic acid bioisosteres and proposed synthesis of γ‐amino boronic acid derivatives. Boc=*tert*‐butoxycarbonyl.

We wondered whether γ‐amino boronic esters could be accessed through the addition of α‐amino radicals to vinyl boronic esters. The addition of carbon‐centered radicals to vinyl organoboron molecules has been reported.^10^, ^11^, ^12^ However, the yield of the desired product, regiochemistry (addition at the α or the β carbon center of the vinyl substrate), and the ability to suppress deleterious polymerization is highly dependent on the nature of the two substrates. Recently, Zard and co‐workers addressed some of these issues through the addition of a variety of carbon‐centered radicals to tetracoordinate *N*‐methylimidodiacetate (MIDA)‐ligated vinyl organoborons (Scheme 1 b).11a Accessing the carbon‐centered radicals by the fragmentation of xanthates resulted in a group‐transfer reaction, with the dithiocarbonyl moiety ending up at the position α to the boron atom. Despite the wide substrate scope and good regiocontrol, the xanthate starting materials need to be prepared and the dithiocarbonyl group next to boron ultimately transformed into something more useful.

We reasoned that photoredox catalysis would allow direct access to γ‐amino organoborons through the radical‐based decarboxylative union of readily available α‐amino acids and vinyl boronic esters, where the nature of the photocatalyst could be used to ensure good yield and wide substrate scope.^13^ MacMillan and co‐workers reported related reactions with acrylates and other electron‐deficient olefins (Scheme 1 c, X=CO_2_R).^14^ In their process, following addition of the nucleophilic α‐amino radical (generated by decarboxylation of a carboxylic acid) to the acrylate, the resulting α‐carboxyl radical intermediate was reduced by the photocatalyst to give an enolate, which was then protonated to give the γ‐amino butyric acid derivative. It was unclear whether vinyl boronic esters would participate in a similar transformation as they are only weakly electron‐deficient and the photocatalyst‐mediated single‐electron reduction of an α‐boryl radical, which should be stabilized through π‐donation,11a is unknown (Scheme 1 c, X=B(OR)_2_). We hereby report that photoredox‐catalyzed decarboxylative radical addition of α‐amino acids (and a variety of other acids) to vinyl boronic esters is feasible, thus providing rapid access to novel organoborons for augmenting medicinal chemistry libraries.

To investigate our proposed reaction, initial studies focused on the reaction between Boc‐Pro‐OH (**1**) and the commercially available vinyl boronic acid pinacol ester **2** (Table 1). We were delighted to discover that the cesium salt, formed by deprotonation of **1** with Cs_2_CO_3_, reacted with **2** upon irradiation with 24 W blue LED strips in the presence of Ir[dF(CF_3_)ppy]_2_(dtbbpy)PF_6_ (**A**) in DMF to yield the γ‐amino boronic ester **3** in 44 % yield (entry 1). A significant increase in yield was observed upon switching to the more reducing photocatalyst Ir(ppy)_2_(dtbbpy)PF_6_ (**B**), likely because of the more favorable single‐electron transfer (SET) between the Ir^II^ state of the photocatalyst and the intermediate α‐boryl radical, whereas using the ruthenium photocatalyst **C** proved ineffective (entries 2 and 3). Evaluation of other solvents (entries 4–6) and bases gave no improvements in yield.^15^ We next investigated the effect of the light source on the reaction outcome. While compact fluorescent light (CFL) was relatively ineffective (entry 7), the use of a more powerful 40 W blue LED lamp gave a dramatic improvement in reaction efficiency, with **3** being formed in 82 % yield (entry 8). In addition, the use of the organic photocatalyst 4CzIPN (**D**) under the optimized reaction conditions afforded **3** in 66 % yield, thus demonstrating that **D** is a viable alternative to the iridium photocatalyst (entry 9).^16^ Finally, control experiments were performed to confirm the essential roles of the base, photocatalyst, and light (entries 10–12).

**Table 1:** Optimization studies.^[a]^ 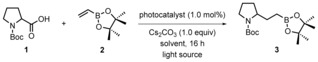

Entry	Photocatalyst	Solvent	Light source	Yield [%]^[b]^
1	**A**	DMF	24 W blue LED strips	44
2	**B**	DMF	24 W blue LED strips	62
3	**C**	DMF	24 W blue LED strips	0
4	**B**	DMA	24 W blue LED strips	29
5	**B**	DMSO	24 W blue LED strips	39
6	**B**	MeCN	24 W blue LED strips	5
7	**B**	DMF	20 W CFL	8
8	**B**	DMF	40 W blue LED lamp	82
9^[c]^	**D**	DMF	40 W blue LED lamp	66
10^[d]^	**B**	DMF	40 W blue LED lamp	1
11	none	DMF	40 W blue LED lamp	0
12	**B**	DMF	none	0

[a] All reactions were carried out with **1** (0.050 mmol), **2** (0.075 mmol), Cs_2_CO_3_ (0.050 mmol), the photocatalyst (0.50 μmol), and solvent (1.0 mL). [b] Determined by GC using 1,2,4‐trimethoxybenzene as an internal standard. [c] Using 5.0 mol % of the photocatalyst **D**. [d] Reaction performed without Cs_2_CO_3_. **A**=Ir[dF(CF_3_)ppy]_2_(dtbbpy)PF_6_, **B**=Ir(ppy)_2_(dtbbpy)PF_6_, **C**=Ru(phen)_3_Cl_2_⋅*x* H_2_O, **D**=4CzIPN. DMA=*N*,*N*‐dimethylacetamide, DMF=*N*,*N*‐dimethylformamide.

Having established optimal reaction conditions for the decarboxylative radical addition to vinyl boronic esters, we explored the scope of the transformation (Table 2). Various cyclic amino acids possessing different carbamoyl protecting groups and different ring sizes gave the corresponding products in moderate to good yields (**3**–**7**). Protected acyclic amino acids, including secondary and tertiary, also reacted with good efficiency (**8**–**10**). When these reaction conditions were applied to substrates bearing free NH groups, dramatically reduced yields were observed.^15^ However, slight modification of the reaction conditions (using photocatalyst **A** and DMA as the solvent) allowed these important substrates to participate (Table 2, Conditions B). While primary carboxylic acids reacted with low efficiency (**11**), secondary and tertiary substrates gave the corresponding products in good yields (**12**–**16**). Various functional groups, including aromatic and heteroaromatic rings, thioethers, esters, and primary amides were tolerated (**17**–**21**). Furthermore, the dipeptides Z‐Gly‐Phe‐OH and Z‐Phe‐Leu‐OH gave the γ‐amino boronic esters **22** (48 %) and **23** (63 %), respectively.

**Table 2:** Scope of the synthesis of γ‐amino boronic esters from α‐amino acids.^[a]^

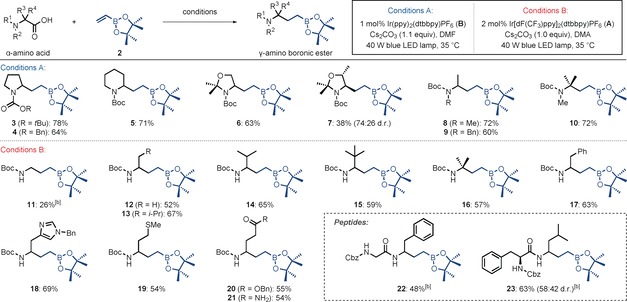

[a] Reactions were carried out on a 0.30 mmol scale with irradiation times of between 30 and 72 h. See the Supporting Information for exact experimental procedures. Yields are of isolated products after chromatographic purification. [b] Reactions were carried out on a 0.20 mmol scale. Cbz=benzyloxycarbonyl.

Next, we investigated the use of different alkenyl boronic esters in the reaction with **1** (Table 3). Both 1‐ and 2‐propenyl boronic esters reacted with high yields (**24** and **25**), whereas the products from the reactions with trisubstituted alkenyl boronic esters were formed in lower yields (**26** and **27**), presumably because of the increased steric hindrance. Pleasingly, protected alcohols could be incorporated into the vinyl boronic ester (**28** and **29**). Surprisingly, however, when an α‐styrenyl boronic ester was employed, the desired product **30** was not obtained because of the rapid protodeboronation under the reaction conditions.^15^

**Table 3:** Alkenyl boronic ester substrates.^[a]^

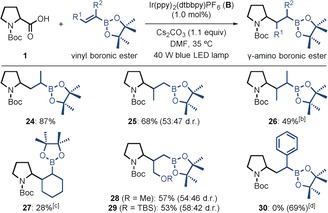

[a] Reactions were carried out on a 0.30 mmol scale. Yields are of isolated product after chromatographic purification. [b] Undetermined d.r. [c] Formed with 44:33:19:4 d.r. [d] Protodeboronation occurred under the reaction conditions; the yield of the corresponding product is given within parentheses.

To further demonstrate the utility of this decarboxylative alkyl boronic ester synthesis we wanted to extend the scope to include simple alkyl carboxylic acids (Table 4). Although initial attempts demonstrated that **A** could promote the desired reaction, it was found that optimum yields were obtained when using the more reducing photocatalyst Ir[dF(Me)ppy]_2_(dtbbpy)PF_6_ (**E**).^17^ We were delighted to discover that simple cycloalkane carboxylic acids of various ring sizes and acyclic secondary carboxylic acids reacted to give the corresponding alkyl boronic esters in moderate to good yields (**31**–**35**). Carboxylic acids with α‐oxy groups, including both cyclic and acyclic examples, also performed well (**36** and **37**). Furthermore, cyclic and acyclic tertiary carboxylic acids reacted to give the corresponding products with moderate to excellent yields (**38**–**41**). Finally, this methodology was applied to a variety of carboxylic acid natural products and drugs, including dehydroabietic acid (**42**), the vitamin E analogue Trolox (**43** and **44**), the fibrate‐drugs bezafibrate (**45**) and gemfibrozil (**46**), and a tartaric acid derivative (**47**). Importantly, the successful application of these substrates demonstrates the potential for late‐stage introduction of an alkyl boronic ester group into complex molecules possessing diverse functionality, including aryl ethers, phenols, aryl halides, amides, and esters.

**Table 4:** Alkyl carboxylic acid substrates.^[a]^

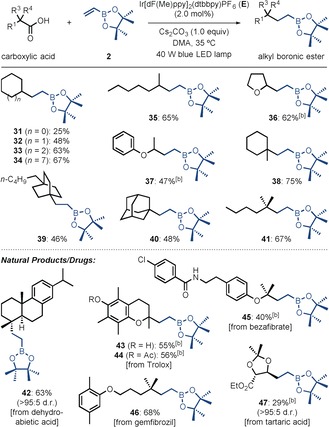

[a] Reactions were carried out on a 0.20 mmol scale. Yields are of isolated products after chromatographic purification. [b] Reactions were carried out on a 0.30 mmol scale with photocatalyst **A**.

Our proposed mechanism for this decarboxylative conjugate addition reaction is outlined in Scheme 2 a. Initial SET between the photoexcited iridium catalyst and the carboxylate formed upon deprotonation of **1** generates a carboxyl radical that undergoes decarboxylation to form the α‐amino radical **48**. Conjugate addition to **2** produces the stabilized α‐boryl radical **49**, which undergoes SET with the reduced Ir^II^ photocatalyst to give the α‐boryl anion **50**.^18^ Final protonation then furnishes the γ‐amino boronic ester product **3**. Support for the proposed single‐electron reduction of **49** to **50** was provided by performing deuterium‐labeling studies (Scheme 2 b).^15^ When the reaction of vinyl boronic ester **2** was carried out with the preformed cesium salt **51** in the presence of deuterium oxide the desired product **3** was formed with 58 % D incorporation α to the boronic ester group, thus confirming the formation of α‐boryl anion **50**. Calculation of the single‐electron reduction potential for **49** using DFT calculations gave a value of −1.25 V vs. SCE in MeCN,^19^, ^20^ thus suggesting that thermodynamically favorable SET will occur with both **A** (*E*_1/2_ [Ir^III^/Ir^II^]=−1.37 V vs. SCE in MeCN) and **B** (*E*_1/2_ [Ir^III^/Ir^II^]=−1.51 V vs. SCE in MeCN).13a

**Scheme 2:**
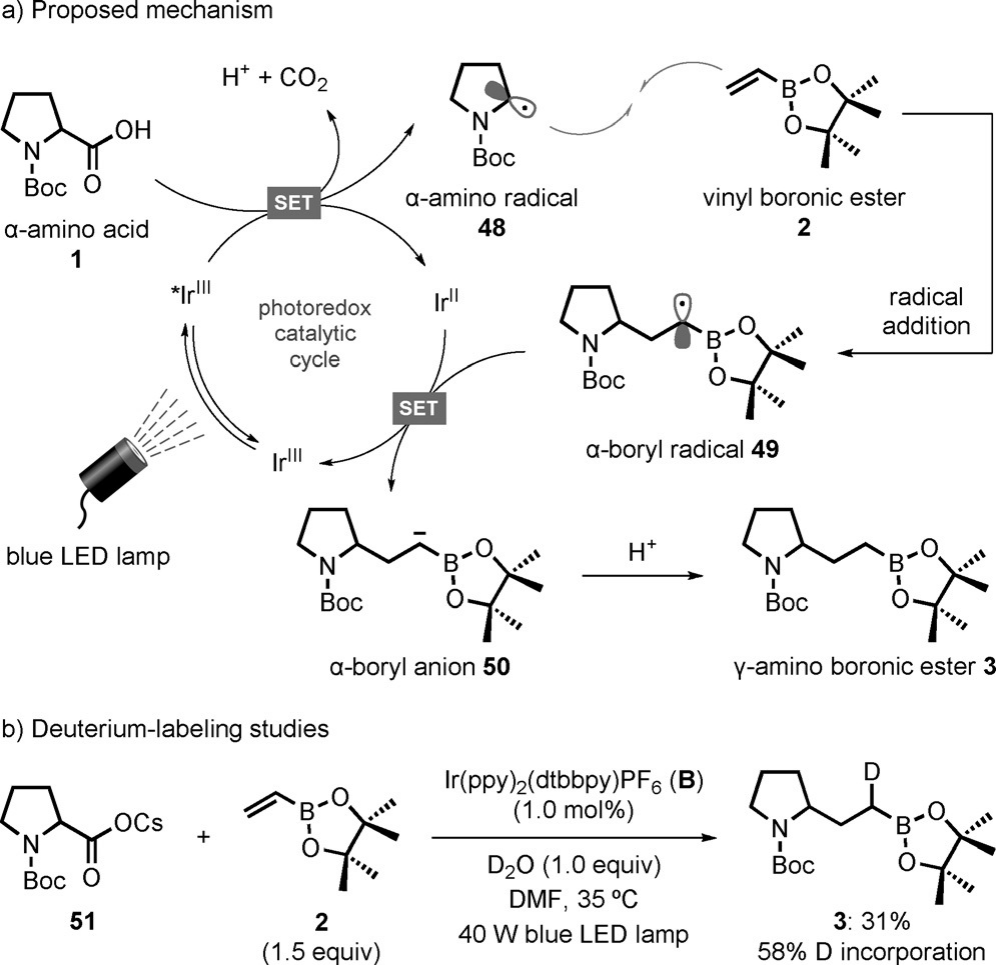
Proposed mechanism and deuterium‐labeling studies.

In conclusion, a novel visible‐light‐mediated decarboxylative radical addition to vinyl boronic esters has been developed for synthesizing alkyl boronic esters from abundant alkyl carboxylic acid substrates. A mechanism involving an unprecedented single‐electron reduction of an α‐boryl radical by the reduced‐state photocatalyst was supported by deuterium‐labeling studies and DFT calculations. Excellent functional‐group tolerance was demonstrated through application to a vast array of substrates, including various natural products and drug scaffolds. Importantly, the rapid access to structurally diverse alkyl boronic esters provided by this methodology will likely be of significant value to medicinal chemists, both as an end‐point and as a site for subsequent diversification.

## Conflict of interest

The authors declare no conflict of interest.

## Supporting information

As a service to our authors and readers, this journal provides supporting information supplied by the authors. Such materials are peer reviewed and may be re‐organized for online delivery, but are not copy‐edited or typeset. Technical support issues arising from supporting information (other than missing files) should be addressed to the authors.

Supplementary
