# Supplementary material for: Visible‐Light‐Mediated Decarboxylative Radical Additions to Vinyl Boronic Esters: Rapid Access to γ‐Amino Boronic Esters
Source: Angew Chem Int Ed Engl. 2018 Jan 26;57(8):2155–9. doi: 10.1002/anie.201712186 (PMC5838549; doi:10.1002/anie.201712186)

Angewandte Chemie
Eine Zeitschrift der Gesellschaft Deutscher Chemiker

Supporting Information

Visible-Light-Mediated Decarboxylative Radical Additions to Vinyl Boronic Esters: Rapid Access to γ-Amino Boronic Esters

*Adam Noble[+], Riccardo S. Mega[+], Daniel Pflästerer, Eddie L. Myers, and Varinder K. Aggarwal**

anie_201712186_sm_miscellaneous_information.pdf

Contents

1. General Information 2

2. Photocatalyst Structures 3

3. Base Optimisation Studies 4

4. Optimisation Studies for α-Amino Acids with Free N–H Groups 5

5. Reaction Setup 5

6. General Procedures 6

7. Product Characterisation 7

8. Deuteration Studies 48

9. Cyclic Voltammetry Studies 50

10. DFT Calculation of Reduction Potential 51

11. Complete List of Authors for Reference 8 53

12. ^{1}H and ^{13}C NMR Spectra 54

1. General Information

All reactions were carried out at room temperature (r.t.). Heat generated from the LED lamps resulted in warming of the reactions to 35 ºC, if necessary fan cooling was used to maintain this temperature. Water is de-ionised and brine refers to a saturated aqueous solution of NaCl. Vinylboronic acid pinacol ester (**2**) was purified by Kugelrohr distillation (room temperature, 1.0 mbar, with a –78 °C collection flask) prior to use. DMF was reagent grade (unless otherwise stated). DMA was anhydrous purchased from Sigma-Aldrich. *N*-Bn-Boc-Ala-OH,[1] Boc-serine acetonide,[2] Boc-threonine acetonide,[3] dehydroabietic acid,[4] Trolox acetate,[5] and monoethyl tartrate acetonide[6] were prepared according to literature procedures. All other reagents were used as received unless otherwise stated.

40 W Kessil Blue LED lamps were Kessil A160WE tuna blue LED aquarium lights (purchased from http://charterhouse-aquatics.com) and were used with colour dial turned fully anticlockwise and intensity turned fully clockwise.

Flash column chromatography was carried out using silica gel (Aldrich, silica gel 60, 40-63 μm). Analytical thin-layer chromatography (TLC) was performed using aluminum-backed silica plates (0.25 mm, Merck, silica gel 60 F254). Compounds were visualised under UV light or by staining with aqueous basic potassium permanganate, an ethanolic solution of phosphomolybdic acid, or an ethanolic solution of nihydrin.

^{1}H, ^{13}C and ^{11}B NMR spectra were acquired at various field strengths, as indicated, using Bruker 400 MHz, Varian VNMR 400 MHz, Varian VNMR 500 MHz, and Bruker Cryo 500 MHz spectrometers. Chemical shifts (δ) are given in parts per million (ppm) and referenced to $CDCl_3$ (7.26 ppm) or DMSO-d_6 (2.50 ppm). Coupling constants (*J*) are given in Hertz (Hz) and refer to apparent multiplicities (s = singlet, br. s = broad singlet, d = doublet, t = triplet, q = quartet, quin = quintet, sex = sextet, h = heptet, m = multiplet, dd = doublet of doublets, etc.). The ^{1}H NMR spectra are reported as follows: chemical shift (multiplicity, coupling constants, number of protons).

[1] Yoon, U. C.; Jin, Y. X.; Oh, S. W.; Park, C. H.; Park, J. H.; Campana, C. F.; Cai, X.; Duesler, E. N.; Mariano, P. S. *J. Am. Chem. Soc.* **2003**, *125*, 10664.
[2] Anderson, Z. J.; Fox, D. J. *Org. Biomol. Chem.* **2016**, *14*, 1450.
[3] Sharma, A.; Blair, P. M.; Mitchell, D. A. *Org. Lett.* **2013**, *15*, 5076.
[4] Thommen, C.; Jana, C. K.; Neuburger, M.; Gademann, K. *Org. Lett.* **2013**, *15*, 1390
[5] (a) Gurken, A. S.; Karabay, A. Z.; Buyukbingol, Z.; Buyukbingol, E. *Eur. J. Med. Chem.* **2011**, *46*, 468. (b) Witkowski, S.; Paradowska, K.; Wawer, I. *Magn. Res. Che.* **2004**, *42*, 863.
[6] Yeager, A. R.; Finney, N. S. *Bioorg. Med. Chem.* **2004**, *12*, 6451.

High-resolution mass spectra (HRMS) were recorded on a Bruker micrOTOF instrument using electrospray ionisation (ESI) or Bruker UltrafleXtreme using matrix-assisted laser desorption/ionisation (MALDI).

Infra-red (IR) spectra were recorded on a Perkin Elmer Spectrum One FT-IR spectrometer as a thin film. Selected absorption maxima (ν_{max}) are reported in wavenumbers (cm^{-1}).

Melting points were recorded in degrees Celsius (°C), using a Kofler hot-stage microscope apparatus and are reported uncorrected.

Optical rotation ($[\alpha]_D^T$) was measured on a Bellingham and Stanley Ltd. ADP220 polarimeter and is quoted in (° mL)(g dm)$^{-1}$.

Cyclic voltammetry was preformed using an Autolab PGSTAT30 potentiostat. Analysis was performed using general purpose electrochemical system (GPES) software.

2. Photocatalyst Structures

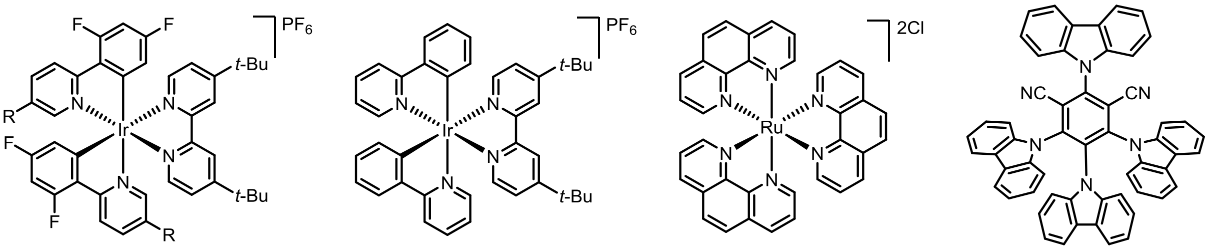

A = Ir[dF(CF_3)ppy]$_2$(dtbbpy)PF_6 (R = CF_3)
E = Ir[dF(Me)ppy]$_2$(dtbbpy)PF_6 (R = Me)
B = Ir(ppy)$_2$(dtbbpy)PF_6
C = Ru(phen)$_3Cl_2$
D = 4CzIPN

Photocatalysts **A**, **B** and **E** were prepared following the procedure of Stephenson and co-workers.[7] Photocatalyst **C** was purchased from Aldrich and used as received. Photocatalyst **D** was prepared following the method of Zhang and co-workers.[8]

---

[7] Monos, T. M.; Sun, A. C.; McAtee, R. C.; Devery, J. J., III.; Stephenson, C. R. J. *J. Org. Chem.* **2016**, *81*, 6988.
[8] Luo, J.; Zhang, J. *ACS Catal.* **2016**, *6*, 873.

3. Base Optimisation Studies

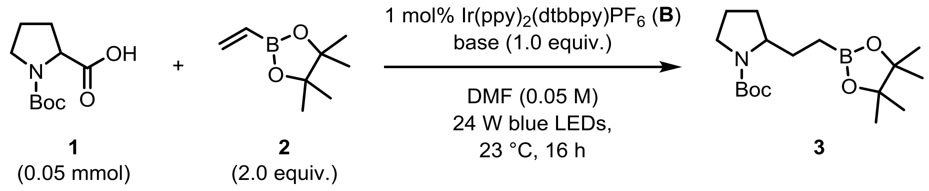

Entry	base	**3** (% yield)[a]
1	None	0
2	Li_2CO_3	1
3	Na_2CO_3	25
4	K_2CO_3	40
**5**	**Cs_2CO_3**	**62**
6	$NaHCO_3$	8
7	$KHCO_3$	24
8	$LiOH \cdot H_2O$	22
9	NaOH	6
10	KOH	43
11	NaOAc	8
12	KOAc	27
13	NaH_2PO_4	0
14	Na_2HPO_4	0
15	KH_2PO_4	0
16	K_2HPO_4	12
17	K_3PO_4	34
18	NaF	0
19	quinuclidine	4
20	DABCO	0
21	DBU	41
22	pyridine	0
23	2,6-lutidine	0

[a] Determined by GC using 1,2,4-trimethoxybenznene as an internal standard.

4. Optimisation Studies for α-Amino Acids with Free N–H Groups

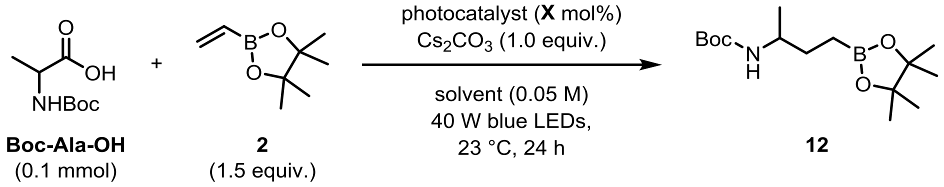

Entry	photocatalyst	photocatalyst loading (mol%)	solvent	**12** (% yield)[a]
1	**B**	1	DMF	8
2	**A**	1	DMF	39
3	**A**	1	DMSO	38
4	**A**	1	DMA	58
**5**	**A**	**2**	**DMA**	**71**

[a] Determined by ^{1}H NMR using 1,2,4-trimethoxybenznene as an internal standard.

5. Reaction Setup

The Kessil lamps were positioned 5 cm from the reaction vial. When 1 × lamp was used, a mirror was placed beneath the vial at an angle of 45° (see diagram A below). When 2 × lamps were used, the lamps were positioned on opposite sides of the reaction vial, each at a distance of 5 cm (see diagram B below).

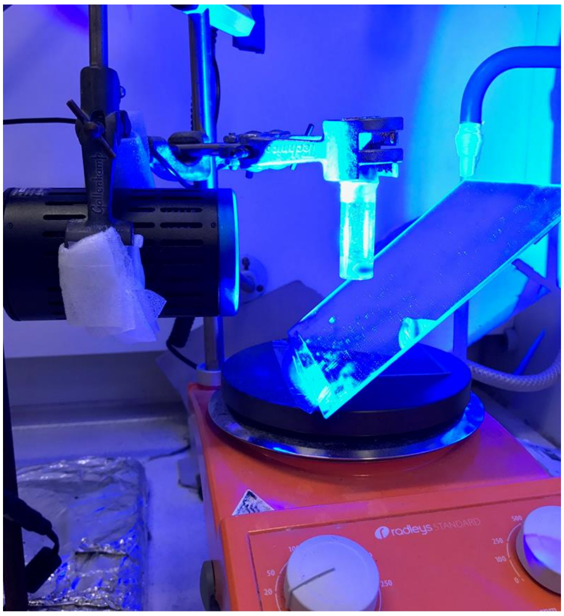

Diagram A

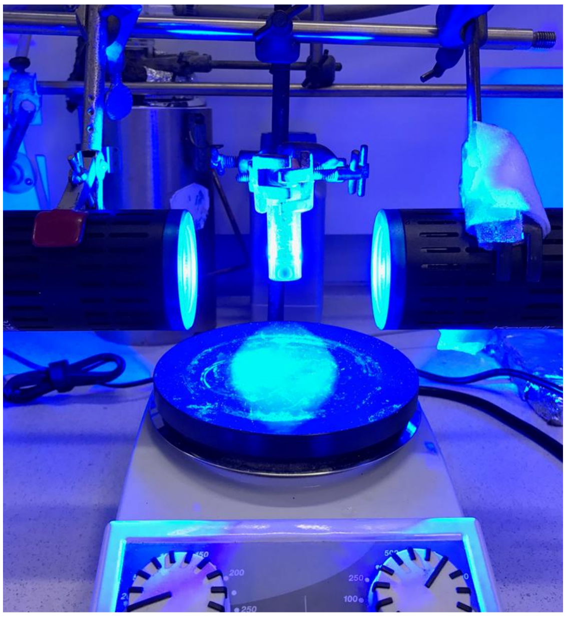

Diagram B

6. General Procedures

General Procedure A [For use with fully protected α-amino acids (Table 2, Conditions A and Table 3)]:

To a 7 mL vial equipped with a stir bar was added the amino acid (1.0 equiv.), Ir(ppy)$_2$(dtbbpy)PF$_6$ (1.0 mol%) and Cs_2CO_3 (1.1 equiv.). DMF (0.10 M) was then added followed by vinyl boronic ester pinacol ester (1.5 equiv.). The vial was sealed with a suba-seal and the reaction mixture degassed by sparging with nitrogen for 10 minutes. The nitrogen inlet was removed and the vial further sealed with parafilm. The reaction mixture was stirred at 800 rpm and irradiated with 40 W blue Kessil LED lamps for between 30 and 62 h. The reaction mixture was diluted with water (20 mL) and extracted into ethyl acetate (3 × 20 ml). The organic phase was washed with water (20 mL), brine (20 mL), dried (Na_2SO_4) and filtered before removal of the solvent *in vacuo*. The crude product was then purified by normal-phase flash column chromatography.

General Procedure B [For use with α-amino acids possessing free N–H groups (Table 2, Conditions B), and α-oxy acids (Table 4)]:

To a 7 mL vial equipped with a stir bar was added the amino acid (1.0 equiv.), Ir[dF(CF$_3$)ppy]$_2$(dtbbpy)PF$_6$ (2.0 mol%) and Cs_2CO_3 (1.0 equiv.). Anhydrous DMA (0.05 M or 0.10 M) was then added followed by vinyl boronic ester pinacol ester (1.5 equiv.). The vial was sealed with a suba-seal and the reaction mixture degassed by sparging with nitrogen for 10 minutes. The nitrogen inlet was removed and the vial further sealed with parafilm. The reaction mixture was stirred at 800 rpm and irradiated with 40 W blue Kessil LED lamps for between 30 and 62 h. The reaction mixture was diluted with water (20 mL) and extracted into ethyl acetate (3 × 20 ml). The organic phase was washed with water (20 mL), brine (20 mL), dried (Na_2SO_4) and filtered before removal of the solvent *in vacuo*. The crude product was then purified by normal-phase flash column chromatography.

General Procedure C [For alkyl carboxylic acids (Table 4)]:

To a 7 mL vial equipped with a stir bar was added the amino acid (1.0 equiv.), Ir[dF(Me)ppy]$_2$(dtbbpy)PF$_6$ (2.0 mol%) and Cs_2CO_3 (1.0 equiv.). Anhydrous DMA (0.050 M

or 0.10 M) was then added followed by vinyl boronic ester pinacol ester (1.5 equiv.). The vial was sealed with a suba-seal and the reaction mixture degassed by sparging with nitrogen for 10 minutes. The nitrogen inlet was removed and the vial further sealed with parafilm. The reaction mixture was stirred at 800 rpm and irradiated with 40 W blue Kessil LED lamps for between 30 and 62 h. The reaction mixture was diluted with water (20 mL) and extracted into ethyl acetate (3 × 20 ml). The organic phase was washed with water (20 mL), brine (20 mL), dried (Na_2SO_4) and filtered before removal of the solvent *in vacuo*. The crude product was then purified by normal-phase flash column chromatography.

7. Product Characterisation

***tert*-Butyl 2-(2-(4,4,5,5-tetramethyl-1,3,2-dioxaborolan-2-yl)ethyl)pyrrolidine-1-carboxylate (3)**

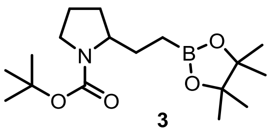

3

Prepared following General Procedure A with Boc-Pro-OH (65 mg, 0.30 mmol, 1.0 equiv.), $Ir(ppy)_2(dtbbpy)PF_6$ (2.7 mg, 0.0030 mmol, 1.0 mol%), Cs_2CO_3 (108 mg, 0.330 mmol, 1.10 equiv.), vinyl boronic acid pinacol ester (76 µL, 0.45 mmol, 1.5 equiv.) and DMF (3.0 mL), which was irradiated with 1 × Kessil lamp for 40 h. Purification by flash column chromatography (10% EtOAc/pentane) gave the title compound (76 mg, 0.23 mmol, 78%) as a colourless oil.

TLC: R_f = 0.33 (10% EtOAc/pentane, $KMnO_4$ stain).

^{1}H NMR (400 MHz, $CDCl_3$): δ_H 3.77 – 3.60 (br. m, 1H), 3.44 – 3.20 (br. m, 2H), 1.93 – 1.59 (m, 5H), 1.43 (s, 9H), 1.43 – 1.30 (m, 1H), 1.21 (s, 12H), 0.74 – 0.64 (m, 2H) ppm.

^{13}C NMR (101 MHz, $CDCl_3$): δ_C 154.8, 82.9, 78.8, 59.1, 46.4 + 46.1 (rotameric peaks), 30.0 + 29.1 (rotameric peaks), 28.6, 28.5 + 28.0 (rotameric peaks), 24.82, 24.80, 23.7 + 23.0 (rotameric peaks), 7.6 (br.) ppm.

^{11}B NMR (128 MHz, $CDCl_3$): δ_B 34.1 (br. s, 1B) ppm.

IR (film) ν_{max}: 2975 – 2874, 1690, 1389, 1379, 1364, 1322, 1166, 1144, 1108, 969 cm^{-1}.

HRMS (ESI$^+$) calcd. for $C_{17}H_{33}BNO_4$ [M+H]$^+$ 326.2500, found 326.2513.

Benzyl 2-(2-(4,4,5,5-tetramethyl-1,3,2-dioxaborolan-2-yl)ethyl)pyrrolidine-1-carboxylate (4)

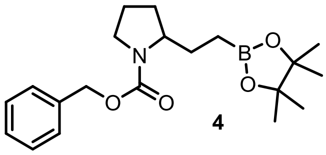

Prepared following General Procedure A using Z-Pro-OH (75 mg, 0.30 mmol, 1.0 equiv.), Ir (ppy)$_2$(dtbbpy)PF_6 (2.7 mg, 0.0030 mmol, 1.0 mol%), Cs_2CO_3 (108 mg, 0.330 mmol, 1.10 equiv.), vinyl boronic acid pinacol ester (76 µL, 0.45 mmol, 1.5 equiv.) and DMF (3.0 mL), which was irradiated with 1 × Kessil lamp for 40 h. Purification by flash column chromatography (20% EtOAc/pentane) gave the title compound (69 mg, 0.19 mmol, 64% yield) as a colourless oil.

TLC: R_f = 0.38 (20% EtOAc/pentane, $KMnO_4$ stain).

^{1}H NMR (400 MHz, $CDCl_3$): δ_H 7.42 – 7.27 (m, 5H), 5.21 – 5.04 (m, 2H), 3.87 – 3.73 (br. s, 1H), 3.53 – 3.30 (m, 2H), 1.98 – 1.64 (br. m, 5H), 1.51 – 1.35 (br. m, 1H), 1.23 (s, 12H), 0.80 – 0.62 (br. m, 2H) ppm.

^{13}C NMR (101 MHZ, $CDCl_3$): δ_C 155.1 + 154.8 (rotameric peaks), 137.3, 128.4, 127.8, 127.7, 83.0 (2C), 66.5 + 66.4 (rotameric peaks), 59.7 + 59.0 (rotameric peaks), 46.7 + 46.3 (rotameric peaks), 29.9 + 29.1 (rotameric peaks), 28.6 + 27.8 (rotameric peaks), 24.9 (2C), 24.8 (2C), 23.7 + 23.0 (rotameric peaks), 7.7 ppm.

^{11}B NMR (128 MHz, $CDCl_3$): δ_B 34.4 (br. s, 1B) ppm.

IR (film) ν_{max}: 2975, 1698, 1408, 1356, 1327, 1143, 1101 cm^{-1}.

HRMS (ESI$^+$): calcd. for $C_{20}H_{31}BNO_4$ [M+H]$^+$ 360.2344, found 360.2342.

tert-Butyl 2-(2-(4,4,5,5-tetramethyl-1,3,2-dioxaborolan-2-yl)ethyl)piperidine-1-carboxylate (5)

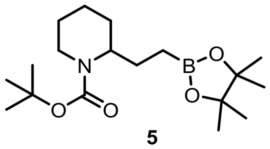

5

Prepared following General Procedure A with Boc-Pip-OH (69 mg, 0.30 mmol, 1.0 equiv.), Ir(ppy)$_2$(dtbbpy)PF$_6$ (2.7 mg, 0.0030 mmol, 1.0 mol%), Cs_2CO_3 (108 mg, 0.330 mmol, 1.10 equiv.), vinyl boronic acid pinacol ester (76 µL, 0.45 mmol, 1.5 equiv.) and DMF (3.0 mL), which was irradiated with 1 × Kessil lamp for 30 h. Purification by flash column chromatography (10% EtOAc/pentane) gave the title compound (72 mg, 0.21 mmol, 71%) as a colourless oil.

TLC: R_f = 0.46 (10% EtOAc/pentane, $KMnO_4$ stain).

^{1}H NMR (400 MHz, $CDCl_3$): δ_H 4.10 (br. s, 1H), 3.93 (br. d, J = 13.5 Hz, 1H), 2.75 (td, J = 13.2, 2.6 Hz, 1H), 1.80 – 1.70 (m, 1H), 1.58 – 1.46 (m, 6H), 1.43 (s, 9H), 1.40 – 1.30 (m, 1H), 1.22 (s, 12H), 0.69 (t, J = 8.3 Hz, 2H) ppm.

^{13}C NMR (101 MHz, $CDCl_3$): δ_C 155.2, 83.0, 78.8, 52.5 (br.), 38.7 (br.), 28.5, 28.1 (br.), 25.7, 24.8, 23.9, 19.1, 8.0 (br.) ppm.

^{11}B NMR (128 MHz, $CDCl_3$): δ_B 34.0 (br. s, 1B) ppm.

IR (film) ν_{max}: 2975 – 2868, 1686, 1415, 1371, 1324, 1269, 1253, 1146, 1046 cm^{-1}.

HRMS (ESI$^+$) calcd. for $C_{18}H_{35}BNO_4$ [M+Na]$^+$ 340.2657, found 340.2664.

tert-Butyl 2,2-dimethyl-4-(2-(4,4,5,5-tetramethyl-1,3,2-dioxaborolan-2-yl)ethyl)oxazolidine-3-carboxylate (6)

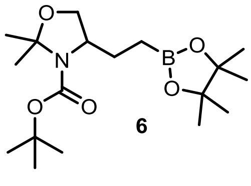

6

Prepared following General Procedure A with 3-(*tert*-butoxycarbonyl)-2,2-dimethyloxazolidine-4-carboxylic acid (74 mg, 0.30 mmol, 1.0 equiv.), Ir(ppy)$_2$(dtbbpy)PF$_6$

(2.7 mg, 0.0030 mmol, 1.0 mol%), Cs_2CO_3 (108 mg, 0.330 mmol, 1.10 equiv.), vinyl boronic acid pinacol ester (76 µL, 0.45 mmol, 1.5 equiv.) and DMF (3.0 mL), which was irradiated with 2 × Kessil lamps for 30 h. Purification by flash column chromatography (5–10% EtOAc/pentane) gave the title compound (67 mg, 0.19 mmol, 63%) as a colourless oil.

TLC: R_f = 0.22 (5% EtOAc/pentane, $KMnO_4$ stain).

^{1}H NMR (400 MHz, $CDCl_3$): δ_H 3.88 (dd, *J* = 8.8, 5.8 Hz, 1H), 3.84 – 3.66 (br. m, 1H), 3.76 (d, *J* = 9.0 Hz, 1H), 1.91 – 1.70 (m, 1H), 1.62 – 1.50 (m, 4H), 1.45 (s, 9H), 1.44 (s, 3H), 1.22 (s, 12H), 0.76 – 0.65 (m, 2H) ppm.

^{13}C NMR (101 MHz, $CDCl_3$): Mixture of rotamers: δ_C 152.2 + 152.0 (rotameric peaks), 93.7 + 93.2 (rotameric peaks), 83.1, 79.8 + 79.3 (rotameric peaks), 66.5 + 66.3 (rotameric peaks), 59.5 + 59.1 (rotameric peaks), 28.5, 27.5 + 26.8 (rotameric peaks), 27.5 + 26.7 (rotameric peaks), 24.82, 24.81, 24.5 + 23.3 (rotameric peaks), 7.7 (br.) ppm.

^{11}B NMR (128 MHz, $CDCl_3$): δ_B 33.8 (br. s, 1B) ppm.

IR (film) ν_{max}: 2978 – 2872, 1694, 1387, 1364, 1324, 1254, 1172, 1144, 1095, 1079 cm^{-1}.

HRMS (ESI^+) calcd. for $C_{18}H_{34}BNO_5Na$ $[M+Na]^+$ 378.2426, found 378.2435.

tert-Butyl (4*R*,5*R*)-2,2,5-trimethyl-4-(2-(4,4,5,5-tetramethyl-1,3,2-dioxaborolan-2-yl)ethyl)oxazolidine-3-carboxylate (**7**)

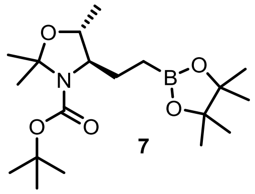

7

Prepared following General Procedure A with (4*S*,5*R*)-3-(tert-butoxycarbonyl)-2,2,5-trimethyloxazolidine-4-carboxylic acid (78 mg, 0.30 mmol, 1.0 equiv.), $Ir(ppy)_2(dtbbpy)PF_6$ (2.7 mg, 0.0030 mmol, 1.0 mol%), Cs_2CO_3 (108 mg, 0.330 mmol, 1.10 equiv.), vinyl boronic acid pinacol ester (76 µl, 0.45 mmol, 1.5 equiv.) and DMF (6.0 ml), which was irradiated with 2 × Kessil lamps for 62 h. Purification by flash column chromatography (10% EtOAc/pentane) gave the title compound (42 mg, 0.11 mmol, 38%) as a colourless oil. The diastereomeric ratio was determined to be 74:26 by high temperature NMR in DMSO-d_6.

TLC: R_f = 0.45 (10% EtOAc/pentane, $KMnO_4$ stain).

^{1}H NMR (500 MHz, DMSO-d_6, 100 °C): 74:26 ratio of diastereomers: δ_H 4.20 – 4.14 (minor, m, 1H), 3.95 – 3.90 (major, m, 1H), 3.71 – 3.67 (minor, m, 1H), 3.34 – 3.31 (major, m, 1H), 1.80 – 1.53 (m, 2H), 1.51 (major, s, 3H), 1.47 (minor, s, 3H), 1.45 (minor, s, 3H), 1.44 (minor, s, 9H), 1.44 (major, s, 9H), 1.41 (major, s, 3H), 1.23 (major, d, J = 6.5 Hz, 3H), 1.21 (minor, s, 12H), 1.21 (major, s, 12H), 1.17 (major, d, J = 6.4 Hz, 3H), 0.75 – 0.57 (m, 2H) ppm.

^{13}C NMR (126 MHz, DMSO-d_6, 100 °C): Mixture of diastereomers: δ_C 151.0 (minor), 150.9 (major), 92.5 (major), 91.0 (minor), 82.2 (major), 82.1 (minor), 78.3 (major), 78.2 (minor), 73.8 (major), 71.4 (minor), 64.1 (major), 60.4 (minor), 27.62 (minor), 27.58 (major), 27.2, 26.0, 25.1, 24.07 (major), 24.05 (minor), 19.8, 13.6 ppm. The carbon directly attached to boron was not detected due to the boron quadrupole.

^{11}B NMR (128 MHz, $CDCl_3$): δ_B 33.3 (br. s, 1B) ppm.

IR (film) ν_{max}: 2977 – 2933, 1693, 1454, 1386, 1363, 1316, 1253, 1165, 1144, 1120, 1074, 968 cm^{-1}.

HRMS (ESI$^+$) calcd. for $C_{19}H_{36}BNNaO_5$ $[M+Na]^+$ 392.2582, found 392.2580.

***tert*-Butyl methyl(4-(4,4,5,5-tetramethyl-1,3,2-dioxaborolan-2-yl)butan-2-yl)carbamate (8)**

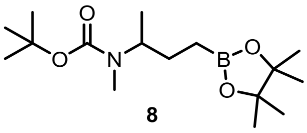

8

Prepared following General Procedure A with *N*-Me-Boc-Ala-OH (61 mg, 0.30 mmol, 1.0 equiv.), $Ir(ppy)_2(dtbbpy)PF_6$ (2.7 mg, 0.0030 mmol, 1.0 mol%), Cs_2CO_3 (108 mg, 0.330 mmol, 1.10 equiv.), vinyl boronic acid pinacol ester (76 µL, 0.45 mmol, 1.5 equiv.) and DMF (3.0 mL), which was irradiated with 1 × Kessil lamp for 40 h. Purification by flash column chromatography (10% EtOAc/pentane) gave the title compound (68 mg, 0.22 mmol, 72%) as a colourless oil.

TLC: R_f = 0.41 (10% EtOAc/pentane, $KMnO_4$ stain).

^{1}H NMR (400 MHz, $CDCl_3$): δ_H 4.17 + 3.96 (rotameric peaks, 2 × br. s, 1H), 2.65 + 2.62 (rotameric peaks, 2 × br. s, 3H), 1.57 – 1.38 (br. m, 2H), 1.44 (s, 9H), 1.23 (s, 12H), 1.04 (d, *J* = 6.8 Hz, 3H), 0.71 – 0.67 (m, 2H) ppm.

^{13}C NMR (101 MHz, $CDCl_3$): δ_C 156.0, 83.0, 79.0 + 78.8 (rotameric peaks), 53.0 + 51.6 (rotameric peaks), 28.5, 28.4 + 28.2 (rotameric peaks), 27.0, 24.84, 24.82, 18.2 + 17.8 (rotameric peaks), 8.1 (br.) ppm.

^{11}B NMR (128 MHz, $CDCl_3$): δ_B 33.8 (br. s, 1B) ppm.

IR (film) ν_{max}: 2976 – 2931, 1691, 1379, 1367, 1336, 1165, 1144 cm^{-1}.

HRMS (ESI^+) calcd. for $C_{16}H_{33}BNO_4$ $[M+H]^+$ 314.2500, found 314.2509.

***tert*-Butyl benzyl(4-(4,4,5,5-tetramethyl-1,3,2-dioxaborolan-2-yl)butan-2-yl)carbamate (9)**

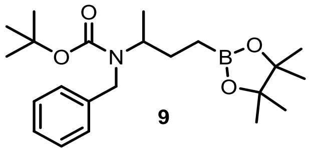

9

Prepared following General Procedure A with *N*-Bn-Boc-Ala-OH (84 mg, 0.30 mmol, 1.0 equiv.), $Ir(ppy)_2(dtbbpy)PF_6$ (2.7 mg, 0.0030 mmol, 1.0 mol%), Cs_2CO_3 (108 mg, 0.330 mmol, 1.10 equiv.), vinyl boronic acid pinacol ester (76 μL, 0.45 mmol, 1.5 equiv.) and DMF (3.0 mL), which was irradiated with 1 × Kessil lamp for 30 h. Purification by flash column chromatography (10% EtOAc/pentane) gave the title compound (70 mg, 0.18 mmol, 60%) as a colourless oil.

TLC: R_f = 0.50 (10% EtOAc/pentane, $KMnO_4$ stain).

^{1}H NMR (400 MHz, $CDCl_3$): δ_H 7.29 – 7.17 (m, 5H), 4.43 – 3.71 (br. m, 3H), 1.70 – 1.27 (br. m, 11H), 1.23 (s, 12H), 1.04 (br. s, 3H), 0.72 (br. s, 2H) ppm.

^{13}C NMR (126 MHz, $CDCl_3$): δ_C 156.4 + 155.9 (rotameric peaks), 140.6 + 140.0 (rotameric peaks), 128.1, 127.4, 126.6 + 126.4 (rotameric peaks), 83.0, 79.3, 54.9 + 53.3 (rotameric peaks), 47.5 + 46.2 (rotameric peaks), 29.2 + 29.0 (rotameric peaks), 28.5 + 28.3 (rotameric peaks), 24.8, 19.4 + 18.8 (rotameric peaks), 8.2 (br.) ppm.

^{11}B NMR (128 MHz, $CDCl_3$): δ_B 34.3 (br. s, 1B) ppm.

IR (film) ν_{max}: 2976 – 2931, 1688, 1453, 1406, 1366, 1338, 1321, 1249, 1165, 1145 cm^{-1}.

HRMS (ESI$^+$) calcd. for $C_{22}H_{37}BNO_4$ [M+H]$^+$ 390.2814, found 390.2824.

***tert*-Butyl methyl(2-methyl-4-(4,4,5,5-tetramethyl-1,3,2-dioxaborolan-2-yl)butan-2-yl)carbamate (10)**

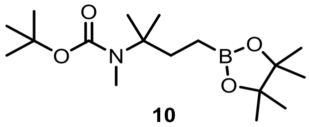

10

Prepared following General Procedure A with *N*-Me-Boc-Aib-OH (65 mg, 0.30 mmol, 1.0 equiv.), Ir(ppy)$_2$(dtbbpy)PF_6 (2.7 mg, 0.0030 mmol, 1.0 mol%), Cs_2CO_3 (108 mg, 0.330 mmol, 1.10 equiv.), vinyl boronic acid pinacol ester (76 µL, 0.45 mmol, 1.5 equiv.) and DMF (3.0 mL), which was irradiated with 1 × Kessil lamp for 40 h. Purification by flash column chromatography (10% EtOAc/pentane) gave the title compound (71 mg, 0.22 mmol, 72%) as a colourless oil.

TLC: R_f = 0.49 (10% EtOAc/pentane, $KMnO_4$ stain).

^{1}H NMR (400 MHz, $CDCl_3$): δ_H 2.83 (s, 3H), 1.56 – 1.80 (m, 1H), 1.44 (s, 9H), 1.28 (s, 6H), 1.21 (s, 12H), 0.68 – 0.64 (m, 2H) ppm.

^{13}C NMR (101 MHz, $CDCl_3$): δ_C 156.2, 82.9, 79.1, 58.1, 34.8, 32.1, 28.6, 27.3, 24.8, 5.6 (br.) ppm.

^{11}B NMR (128 MHz, $CDCl_3$): δ_B 34.4 (br. s, 1B) ppm.

IR (film) ν_{max}: 2976 – 2930, 1698, 1681, 1364, 1325, 1144, 1097 cm^{-1}.

HRMS (ESI$^+$) calcd. for $C_{17}H_{35}BNO_4$ [M+H]$^+$ 328.2657, found 328.2654.

***tert*-Butyl (3-(4,4,5,5-tetramethyl-1,3,2-dioxaborolan-2-yl)propyl)carbamate** (**11**)

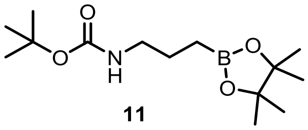

11

Prepared following General Procedure B with Boc-Gly-OH (35 mg, 0.20 mmol, 1.0 equiv.), Ir[dF(CF$_3$)ppy]$_2$(dtbbpy)PF$_6$ (4.5 mg, 0.0040 mmol, 2.0 mol%), Cs_2CO_3 (65 mg, 0.20 mmol, 1.0 equiv.), vinyl boronic acid pinacol ester (51 µL, 0.30 mmol, 1.5 equiv.) and DMA (4.0 mL), which was irradiated with 2 × Kessil lamps for 62 h. Purification by flash column chromatography (10% EtOAc/pentane) gave the title compound (15 mg, 0.053 mmol, 26%) as a colourless oil.

TLC: R_f = 0.18 (10% EtOAc/pentane, $KMnO_4$ stain).

^{1}H NMR (400 MHz, $CDCl_3$): δ_H 4.72 + 4.35 (rotameric peaks, 2 × br. s, 1H), 3.12 – 3.07 (m, 2H), 1.59 (p, *J* = 7.3 Hz, 1H), 1.43 (s, 9H), 1.24 (s, 12H), 0.79 (t, *J* = 7.7 Hz, 2H) ppm.

^{13}C NMR (101 MHz, $CDCl_3$): δ_C 156.0, 83.2, 78.8, 42.6, 28.5, 24.8, 24.1, 8.5 (br.) ppm.

^{11}B NMR (128 MHz, $CDCl_3$): δ_B 34.1 (br. s, 1B) ppm.

IR (film) ν_{max}: 3374, 2978 – 2874, 1705, 1517, 1380, 1366, 1320, 1247, 1168, 1145 cm^{-1}.

HRMS (ESI$^+$) calcd. for $C_{14}H_{29}BNO_4$ $[M+H]^+$ 286.2187, found 286.2195.

***tert*-Butyl (4-(4,4,5,5-tetramethyl-1,3,2-dioxaborolan-2-yl)butan-2-yl)carbamate** (**12**)

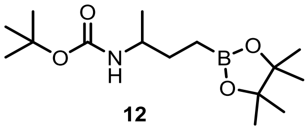

12

Prepared following General Procedure B using Boc-Ala-OH (57 mg, 0.30 mmol, 1.0 equiv.), Ir[dF(CF$_3$)ppy]$_2$(dtbbpy)PF$_6$ (6.7 mg, 0.0060 mmol, 2.0 mol%), Cs_2CO_3 (98 mg, 0.30 mmol, 1.0 equiv.), vinyl boronic acid pinacol ester (76 µL, 0.45 mmol, 1.5 equiv.) and DMA (3.0 mL), which was irradiated with 2 × Kessil lamps for 40 h. Purification by flash column chromatography (10% EtOAc/pentane) gave the title compound (47 mg, 0.16 mmol, 52% yield) as a colourless crystalline solid.

TLC: R_f = 0.38 (10% EtOAc/pentane, $KMnO_4$ stain).

Mpt: 43 – 44 ºC (EtOAc).

^{1}H NMR (400 MHz, $CDCl_3$): δ_H 4.53 (br. s, 1H), 3.61 – 3.46 (br. m, 1H), 1.59 – 1.43 (m, 2H), 1.40 (s, 9H), 1.23 (s, 6H), 1.22 (s, 6H), 1.07 (d, *J* = 6.5 Hz, 3H), 0.77 (t, *J* = 7.9 Hz, 2H) ppm.

^{13}C NMR (101 MHZ, $CDCl_3$): δ_C 155.5, 83.1, 78.7, 48.4, 30.9, 28.4, 24.9, 24.8, 21.2, 7.7 (br.) ppm.

^{11}B NMR (128 MHz, $CDCl_3$): δ_B 34.3 (br. s, 1B) ppm.

IR (film) ν_{max}: 3361 (NH), 2977, 1670, 1511, 1366, 1167, 1145 cm^{-1}.

HRMS (ESI^+): calcd. for $C_{15}H_{31}BNO_4$ $[M+H]^+$ 300.2343, found 300.2337.

***tert*-Butyl (5-methyl-1-(4,4,5,5-tetramethyl-1,3,2-dioxaborolan-2-yl)hexan-3-yl)-carbamate** (**13**)

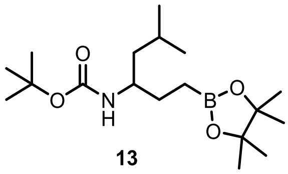

13

Prepared following General Procedure B with Boc-Leu-OH (69 mg, 0.30 mmol, 1.0 equiv.), $Ir[dF(CF_3)ppy]_2(dtbbpy)PF_6$ (6.7 mg, 0.0060 mmol, 2.0 mol%), Cs_2CO_3 (98 mg, 0.30 mmol, 1.0 equiv.), vinyl boronic acid pinacol ester (76 µL, 0.45 mmol, 1.5 equiv.) and DMA (3.0 mL), which was irradiated with 1 × Kessil lamp for 72 h. Purification by flash column chromatography (10% EtOAc/pentane) gave the title compound (69 mg, 0.20 mmol, 67%) as a colourless oil.

TLC: R_f = 0.43 (10% EtOAc/pentane, $KMnO_4$ stain).

^{1}H NMR (400 MHz, $CDCl_3$): δ_H 4.33 (br. d, *J* = 9.4 Hz, 1H), 3.95 (rotameric peak, br. s, 1H), 3.63 – 3.51 (br. m, 1H), 3.45 (rotameric peak, br. s, 1H), 1.66 – 1.55 (m, 2H), 1.44 – 1.32 (m, 1H), 1.42 (br. s, 9H), 1.25 – 1.18 (m, 2H), 1.24 (s, 6H), 1.24 (s, 6H), 0.89 (d, *J* = 6.5 Hz, 3H), 0.89 (d, *J* = 6.6 Hz, 3H), 0.82 – 0.73 (m, 2H) ppm.

^{13}**C NMR** (101 MHz, $CDCl_3$): δ_C 155.8, 83.2, 78.7, 50.6, 45.1, 29.8, 28.6, 25.1, 24.97, 24.95, 23.1, 22.7, 7.3 (br.) ppm.

^{11}**B NMR** (128 MHz, $CDCl_3$): δ_B 33.3 (br. s, 1B) ppm.

IR (film) ν_{max}: 3367, 2976, 2949, 1700, 1505, 1365, 1319, 1249, 1165, 1143, 967 cm^{-1}.

HRMS (ESI$^+$) calcd. for $C_{18}H_{36}BNNaO_4$ $[M+Na]^+$ 364.2633, found 364.2638.

***tert*-Butyl (4-methyl-1-(4,4,5,5-tetramethyl-1,3,2-dioxaborolan-2-yl)pentan-3-yl)-carbamate (14)**

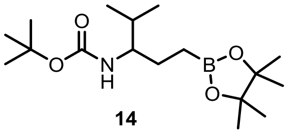

14

Prepared following General Procedure B with Boc-Val-OH (65 mg, 0.30 mmol, 1.0 equiv.), Ir[dF(CF_3)ppy]$_2$(dtbbpy)PF_6 (6.7 mg, 0.0060 mmol, 2.0 mol%), Cs_2CO_3 (98 mg, 0.30 mmol, 1.0 equiv.), vinyl boronic acid pinacol ester (76 µl, 0.45 mmol, 1.5 equiv.) and DMA (3.0 mL), which was irradiated with 1 × Kessil lamp for 72 h. Purification by flash column chromatography (10% EtOAc/pentane) gave the title compound (64 mg, 0.20 mmol, 65%) as an amorphous solid.

TLC: R_f = 0.43 (10% EtOAc/pentane, $KMnO_4$ stain).

^{1}**H NMR** (400 MHz, $CDCl_3$): δ_H 4.38 (br. d, *J* = 9.7 Hz, 1H), 4.02 (rotameric peak, br. s, 1H), 3.33 (tt, *J* = 9.7 Hz, 4.7 Hz, 1H), 3.22 (rotameric peak, br. s, 1H), 1.77 – 1.67 (m, 1H), 1.65 – 1.54 (m, 1H), 1.42 (br. s, 9H), 1.40 – 1.27 (m, 1H), 1.24 (s, 6H), 1.24 (s, 6H), 0.88 (d, *J* = 6.8 Hz, 1H), 0.85 (d, *J* = 6.9 Hz, 1H), 0.84 – 0.71 (m, 2H) ppm.

^{13}**C NMR** (101 MHz, $CDCl_3$): δ_C 156.2, 83.2, 78.7, 57.8, 32.2, 28.6, 26.1, 25.1, 24.9, 19.2, 18.0, 8.0 (br.) ppm.

^{11}**B NMR** (128 MHz, $CDCl_3$): δ_B 34.2 (br. s, 1B) ppm.

IR (film) ν_{max}: 3288, 2976, 2949, 1700, 1678, 1528, 1366, 1314, 1249, 1164, 1146, 1015, 968 cm^{-1}.

HRMS (ESI$^+$) calcd. for $C_{17}H_{35}BNO_4$ $[M+H]^+$ 328.2657, found 328.2669.

***tert*-Butyl (4,4-dimethyl-1-(4,4,5,5-tetramethyl-1,3,2-dioxaborolan-2-yl)pentan-3-yl)carbamate** (**15**)

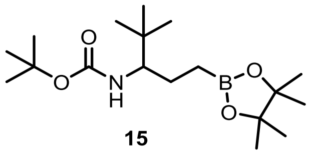

15

Prepared following General Procedure B with Boc-Tle-OH (69 mg, 0.30 mmol, 1.0 equiv.), Ir[dF(CF_3)ppy]$_2$(dtbbpy)PF_6 (6.7 mg, 0.0060 mmol, 2.0 mol%), Cs_2CO_3 (97 mg, 0.30 mmol, 1.0 equiv.), vinyl boronic acid pinacol ester (76 µL, 0.45 mmol, 1.5 equiv.) and DMA (3.0 mL), which was irradiated with 1 × Kessil lamp for 40 h. Purification by flash column chromatography (10% EtOAc/pentane) gave the title compound (60 mg, 0.18 mmol, 59%) as a white solid.

TLC: R_f = 0.73 (10% EtOAc/pentane, $KMnO_4$ stain).

Mpt: 116 – 118 ºC (hexane).

^{1}H NMR (500 MHz, $CDCl_3$): δ_H (83:17 ratio of rotamers) 4.24 (d, *J* = 10.4 Hz, 0.83H), 3.96 (d, *J* = 10.6 Hz), 3.24 (ddd, *J* = 11.5, 10.4, 2.9 Hz, 0.83H), 3.09 (td, *J* = 11.1, 2.5 Hz, 0.17H), 1.73 (dddd, *J* = 13.8, 9.5, 6.5, 2.9 Hz, 1H), 1.44 (s, 1.53H), 1.42 (s, 7.47H), 1.24 (s, 12H), 1.15 (dddd, *J* = 13.8, 11.6, 9.6, 5.8 Hz, 1H), 0.87 (s, 9H), 0.85 (ddd, *J* = 15.8, 9.8, 6.0, 1H), 0.75 (ddd, *J* = 16.2, 9.6, 6.5 Hz) ppm.

^{13}C NMR (126 MHz, $CDCl_3$): Mixture of rotamers: δ_C 156.7 (minor), 156.3 (major), 83.03 (minor), 83.00 (major), 79.3 (minor), 78.5 (major), 63.1 (minor), 61.1 (major), 35.0, 28.5 (major), 28.4 (minor), 26.4, 25.0 (minor), 24.9 (major), 24.8, 24.4 (minor), 24.0 (major), 8.4 (br.) ppm.

^{11}B NMR (128 MHz, $CDCl_3$): δ_B 34.2 (br. s, 1B) ppm.

IR (film) ν_{max}: 3347, 2977 – 2870, 1709, 1531, 1363, 1340, 1328, 1278, 1245, 1175, 1144 cm^{-1}.

HRMS (ESI^+) calcd. for $C_{18}H_{37}BNO_4$ $[M+H]^+$ 342.2814, found 342. 2823.

***tert*-Butyl (2-methyl-4-(4,4,5,5-tetramethyl-1,3,2-dioxaborolan-2-yl)butan-2-yl)carbamate (16)**

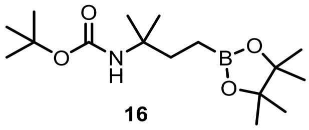

16

Prepared following General Procedure B using Boc-Aib-OH (61 mg, 0.30 mmol, 1.0 equiv.), Ir[dF(CF_3)ppy]$_2$(dtbbpy)PF_6 (6.7 mg, 0.0060 mmol, 2.0 mol%), Cs_2CO_3 (98 mg, 0.30 mmol, 1.0 equiv.), vinyl boronic acid pinacol ester (76 µL, 0.45 mmol, 1.5 equiv.) and DMA (6.0 mL), which was irradiated with 2 × Kessil lamps for 62 h. Purification by flash column chromatography (7% EtOAc/pentane) gave the title compound (54 mg, 0.17 mmol, 57% yield) as a colourless solid.

TLC: R_f = 0.54 (10% EtOAc/pentane, $KMnO_4$ stain).

Mpt: 58 – 60 ºC (EtOAc).

^{1}H NMR (400 MHz, $CDCl_3$): δ_H 4.61 (br. s, 1H), 1.69 – 1.63 (m, 2H), 1.41 (s, 9H), 1.25 (s, 6H), 1.24 (s, 12H), 0.79 – 0.70 (m, 2H) ppm.

^{13}C NMR (101 MHZ, $CDCl_3$): δ_C 154.6, 83.1, 78.4, 52.8, 35.3, 28.5, 26.4, 24.8, 5.3 (br.) ppm.

^{11}B NMR (128 MHz, $CDCl_3$): δ_B 33.8 (br. s, 1B) ppm.

IR (film) ν_{max}: 3394, 2977, 2931, 1720, 1499, 1453, 1366, 1329, 1270, 1168, 1145, 1070 cm^{-1}.

HRMS (MALDI): calcd. for $C_{16}H_{32}BNO_4Na$ $[M+Na]^+$ 336.2320, found 336.2332.

***tert*-Butyl (1-phenyl-4-(4,4,5,5-tetramethyl-1,3,2-dioxaborolan-2-yl)butan-2-yl)carbamate (17)**

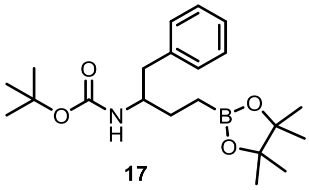

17

Prepared following General Procedure B using Boc-Phe-OH (80 mg, 0.30 mmol, 1.0 equiv.), Ir[dF(CF_3)ppy]$_2$(dtbbpy)PF_6 (6.7 mg, 0.0060 mmol, 2.0 mol%), Cs_2CO_3 (98 mg, 0.30 mmol,

1.0 equiv.), vinyl boronic acid pinacol ester (76 µL, 0.45 mmol, 1.5 equiv.) and DMA (3.0 mL), which was irradiated with 2 × Kessil lamps for 62 h. Purification by flash column chromatography (12% EtOAc/pentane) gave the title compound (71 mg, 0.19 mmol, 63% yield) as a colourless oil.

TLC: R_f = 0.37 (12% EtOAc/pentane, $KMnO_4$ stain).

^{1}H NMR (400 MHz, $CDCl_3$): δ_H 7.30 – 7.19 (m, 2H), 7.20 – 7.10 (m, 3H), 4.59 (d, *J* = 8.7 Hz, 1H), 3.68 (s, 1H), 2.81 (dd, *J* = 13.3, 5.0 Hz, 1H), 2.66 (dd, *J* = 13.2, 7.2 Hz, 1H), 1.61 (dtd, *J* = 14.0, 7.6, 4.4 Hz, 1H), 1.46 – 1.30 (m, 1H), 1.37 (s, 9H), 1.22 (s, 6H), 1.21 (s, 6H), 0.79 (t, *J* = 7.6 Hz, 2H) ppm.

^{13}C NMR (101 MHZ, $CDCl_3$): δ_C 155.5, 138.6, 129.5, 128.2, 126.1, 83.2, 78.8, 53.8, 41.6, 28.4, 27.7, 25.0, 24.8, 7.7 (br.) ppm.

^{11}B NMR (128 MHz, $CDCl_3$): δ_B 34.6 (br. s, 1B) ppm.

IR (film) ν_{max}: 3365, 2977, 2930, 1700, 1497, 1365, 1166, 1143 cm^{-1}.

HRMS (MALDI): calcd. for $C_{21}H_{34}BNO_4Na$ $[M+Na]^+$ 398.2477, found 398.2490.

***tert*-Butyl (1-(1-benzyl-1*H*-imidazol-4-yl)-4-(4,4,5,5-tetramethyl-1,3,2-dioxaborolan-2-yl)butan-2-yl)carbamate (18)**

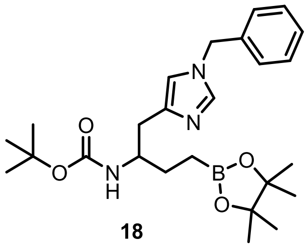

18

Prepared following General Procedure B with Boc-His(Bzl)-OH (104 mg, 0.300 mmol, 1.00 equiv.), $Ir[dF(CF_3)ppy]_2(dtbbpy)PF_6$ (6.7 mg, 0.0060 mmol, 2.0 mol%), Cs_2CO_3 (97 mg, 0.30 mmol, 1.0 equiv.), vinyl boronic acid pinacol ester (76 µL, 0.45 mmol, 1.5 equiv.) and DMA (3.0 mL), which was irradiated with 1 × Kessil lamp for 40 h. Purification by flash column chromatography (5% MeOH/CH_2Cl_2) gave the title compound (94 mg, 0.21 mmol, 69%) as a pale yellow oil.

TLC: R_f = 0.20 (5% MeOH/CH_2Cl_2, $KMnO_4$ stain).

^{1}H NMR (400 MHz, $CDCl_3$): δ_H 7.46 – 7.42 (m, 1H), 7.35 – 7.27 (m, 3H), 7.13 – 7.10 (m, 2H), 6.76 – 6.66 (m, 1H), 5.10 (br. d, *J* = 8.3 Hz, 1H), 5.02 (s, 2H), 3.84 – 3.66 (br. m, 1H), 2.75 – 2.65 (m, 2H), 1.64 – 1.55 (m, 1H), 1.52 – 1.42 (m, 1H), 1.37 (s, 9H), 1.20 (s, 12H), 0.77 (t, *J* = 8.0 Hz, 2H) ppm.

^{13}C NMR (101 MHz, $CDCl_3$): δ_C 155.7, 139.7, 136.4, 136.2, 128.9, 128.2, 127.2, 116.8, 82.9, 78.5, 52.4, 50.8, 33.2, 28.7, 28.4, 24.9, 24.8, 8.0 (br.) ppm.

^{11}B NMR (128 MHz, $CDCl_3$): δ_B 33.7 (br. s, 1B) ppm.

IR (film) ν_{max}: 3362, 2977 – 2872, 1699, 1498, 1455, 1366, 1314, 1241, 1165, 1142 cm^{-1}.

HRMS (ESI^+) calcd. for $C_{25}H_{39}BN_3O_4$ $[M+H]^+$ 456.3033, found 456.3030.

***tert*-Butyl (1-(methylthio)-5-(4,4,5,5-tetramethyl-1,3,2-dioxaborolan-2-yl)pentan-3-yl)carbamate** (**19**)

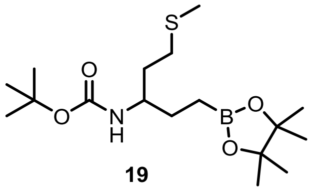

19

Prepared following General Procedure B with Boc-Met-OH (75 mg, 0.30 mmol, 1.0 equiv.), $Ir[dF(CF_3)ppy]_2(dtbbpy)PF_6$ (6.7 mg, 0.0060 mmol, 2.0 mol%), Cs_2CO_3 (98 mg, 0.30 mmol, 1.0 equiv.), vinyl boronic acid pinacol ester (76 µl, 0.45 mmol, 1.5 equiv.) and DMA (3.0 mL), which was irradiated with 1 × Kessil lamp for 72 h. Purification by flash column chromatography (10% EtOAc/pentane) gave the title compound (58 mg, 0.16 mmol, 54%) as a colourless oil.

TLC: R_f = 0.32 (10% EtOAc/pentane, $KMnO_4$ stain).

^{1}H NMR (500 MHz, $CDCl_3$): δ_H 4.50 (br. d, *J* = 9.2 Hz, 1H), 4.11 (rotameric peak, br. s, 1H), 3.61 – 3.46 (br. m, 1H), 2.55 – 2.46 (m, 2H), 2.09 (rotameric peak, s, 3H), 2.09 (s, 3H), 1.81 – 1.72 (m, 1H), 1.67 – 1.58 (m, 2H), 1.50 – 1.39 (m, 1H), 1.44 (rotameric peak, s, 9H), 1.42 (s, 9H), 1.25 (s, 6H), 1.24 (s, 6H), 0.84 – 0.74 (m, 2H) ppm.

^{13}C NMR (126 MHz, $CDCl_3$): δ_C 155.8, 83.3, 79.0, 52.2, 35.5, 30.9, 29.2, 28.6, 25.1, 24.9, 15.7, 7.6 (br.) ppm.

^{11}B NMR (128 MHz, $CDCl_3$): δ_B 33.4 (br. s, 1B) ppm.

IR (film) ν_{max}: 3300, 2974 – 2923, 1698, 1675, 1532, 1425, 1363, 1297, 1250, 1165, 1143, 1048, 1023 cm^{-1}.

HRMS (ESI$^+$) calcd. for $C_{17}H_{35}BNO_4$ [M+H]$^+$ 360.2378, found 360.2388.

Benzyl 4-((*tert*-butoxycarbonyl)amino)-6-(4,4,5,5-tetramethyl-1,3,2-dioxaborolan-2-yl)hexanoate (**20**)

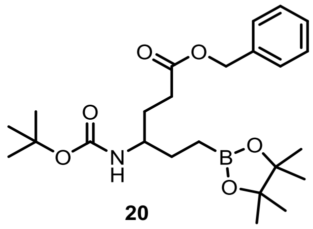

20

Prepared following General Procedure B with Boc-Glu(OBzl)-OH (101 mg, 0.300 mmol, 1.00 equiv.), Ir[dF(CF_3)ppy]$_2$(dtbbpy)PF_6 (6.7 mg, 0.0060 mmol, 2.0 mol%), Cs_2CO_3 (97 mg, 0.30 mmol, 1.0 equiv.), vinyl boronic acid pinacol ester (76 µL, 0.45 mmol, 1.5 equiv.) and DMA (3.0 mL), which was irradiated with 2 × Kessil lamps for 40 h. Purification by flash column chromatography (15% EtOAc/pentane) gave the title compound (74 mg, 0.17 mmol, 55%) as a colourless oil.

TLC: R_f = 0.30 (15% EtOAc/pentane, $KMnO_4$ stain).

^{1}H NMR (400 MHz, $CDCl_3$): δ_H 7.37 – 7.28 (m, 5H), 5.13 – 5.06 (m, 2H), 4.47 (br. d, *J* = 9.2 Hz, 1H), 3.56 – 3.47 (m, 1H), 2.41 (t, *J* = 7.8 Hz, 2H), 1.89 – 1.80 (m, 1H), 1.69 – 1.56 (m, 2H), 1.48 – 1.40 (m, 1H), 1.40 (s, 9H), 1.23 (s, 6H), 1.23 (s, 6H), 0.79 (t, *J* = 7.5 Hz, 2H) ppm.

^{13}C NMR (101 MHz, $CDCl_3$): δ_C 173.4, 155.7, 136.0, 128.5, 128.2, 128.1, 83.2, 78.9, 66.2, 52.1, 31.1, 30.6, 29.2, 28.4, 24.9, 24.8, 7.5 (br.) ppm.

^{11}B NMR (128 MHz, $CDCl_3$): δ_B 33.7 (br. s, 1B) ppm.

IR (film) ν_{max}: 3366, 2977 – 2868, 1735, 1712, 1512, 1452, 1379, 1366, 1319, 1244, 1215, 1165, 1144 cm^{-1}.

HRMS (ESI$^+$) calcd. for $C_{24}H_{39}BNO_5$ [M+H]$^+$ 448.2869, found 448.2873.

***tert*-Butyl (6-amino-6-oxo-1-(4,4,5,5-tetramethyl-1,3,2-dioxaborolan-2-yl)hexan-3-yl)carbamate** (**21**)

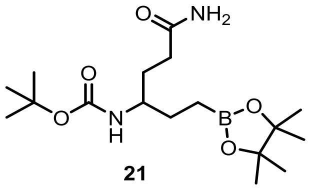

21

Prepared following General Procedure B with Boc-Gln-OH (74 mg, 0.30 mmol, 1.0 equiv.), Ir[dF(CF_3)ppy]$_2$(dtbbpy)PF_6 (6.7 mg, 0.0060 mmol, 2.0 mol%), Cs_2CO_3 (97 mg, 0.30 mmol, 1.0 equiv.), vinyl boronic acid pinacol ester (76 µL, 0.45 mmol, 1.5 equiv.) and DMA (3.0 mL), which was irradiated with 1 × Kessil lamp for 40 h. Purification by flash column chromatography (75–100% EtOAc/pentane) gave the title compound (58 mg, 0.16 mmol, 54%) as a pale yellow oil.

TLC: R_f = 0.24 (75% EtOAc/pentane, ninhydrin stain).

^{1}H NMR (400 MHz, $CDCl_3$): δ_H 6.88 + 6.71 (rotameric peaks, 2 × br. s, 1H), 5.90 + 5.64 (rotameric peaks, 2 × br. s, 1H), 4.69 (d, *J* = 9.1 Hz, 1H), 3.64 – 3.39 (br. m, 1H), 2.29 – 2.16 (m, 2H), 1.82 – 1.74 (m, 1H), 1.66 – 1.55 (m, 2H), 1.47 – 1.39 (m, 1H), 1.39 (s, 9H), 1.21 (s, 6H), 1.21 (s, 6H), 0.78 (t, *J* = 7.7 Hz, 2H) ppm.

^{13}C NMR (101 MHz, $CDCl_3$): δ_C 175.9, 156.7, 83.2, 79.3, 52.1, 32.8, 32.3, 29.3, 28.4, 24.9, 24.7, 7.5 (br.) ppm.

^{11}B NMR (128 MHz, $CDCl_3$): δ_B 33.7 (br. s, 1B) ppm.

IR (film) ν_{max}: 3344, 3203, 2977 – 2870, 1667, 1623, 1516, 1450, 1379, 1366, 1317, 1272, 1245, 1166, 1143 cm^{-1}.

HRMS (ESI$^+$) calcd. for $C_{17}H_{34}BN_2O_5$ [M+H]$^+$ 357.2558, found 357.2560.

Benzyl (2-oxo-2-((1-phenyl-4-(4,4,5,5-tetramethyl-1,3,2-dioxaborolan-2-yl)butan-2-yl)amino)ethyl)carbamate (**22**)

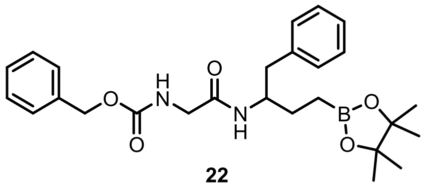

22

Prepared following General Procedure B with Z-Gly-Phe-OH (71 mg, 0.20 mmol, 1.0 equiv.), $Ir[dF(CF_3)ppy]_2(dtbbpy)PF_6$ (4.5 mg, 0.0040 mmol, 2.0 mol%), Cs_2CO_3 (65 mg, 0.20 mmol, 1.0 equiv.), vinyl boronic acid pinacol ester (51 µL, 0.30 mmol, 1.5 equiv.) and DMA (4.0 mL), which was irradiated with 2 × Kessil lamps for 62 h. Purification by flash column chromatography (40% EtOAc/pentane) gave the title compound (45 mg, 0.096 mmol, 48%) as a colourless oil.

TLC: R_f = 0.30 (40% EtOAc/pentane, PMA stain).

^{1}H NMR (400 MHz, $CDCl_3$): δ_H 7.39 – 7.29 (m, 5H), 7.28 – 7.23 (m, 2H), 7.21 – 7.17 (m, 1H), 7.14 (d, *J* = 7.0 Hz, 2H), 5.83 (br. d, *J* = 8.9 Hz, 1H), 5.35 (br. s, 1H), 5.12 (s, 2H), 4.16 – 4.07 (m, 1H), 3.77 (d, *J* = 5.5 Hz, 1H), 2.76 (d, *J* = 6.4 Hz, 1H), 1.70 – 1.62 (m, 1H), 1.49 – 1.40 (m, 1H), 1.23 (s, 6H), 1.23 (s, 6H), 0.79 (t, *J* = 7.9 Hz, 2H) ppm.

^{13}C NMR (101 MHz, $CDCl_3$): δ_C 168.2, 156.4, 137.9, 136.2, 129.4, 128.6, 128.4, 128.2, 128.1, 126.4, 83.3, 67.1, 52.3, 44.6, 40.5, 27.9, 24.9, 24.7 ppm. The carbon directly attached to boron was not detected due to the boron quadrupole.

^{11}B NMR (128 MHz, $CDCl_3$): δ_B 34.7 (br. s, 1B) ppm.

IR (film) ν_{max}: 3401, 3319, 3087 – 2860, 1714, 1661, 1526, 1498, 1455, 1379, 1372, 1319, 1243, 1215, 1165, 1143 cm^{-1}.

HRMS (ESI^+) calcd. for $C_{26}H_{36}BN_2O_5$ $[M+H]^+$ 467.2716, found 467.2719.

Benzyl ((2*S*)-1-((5-methyl-1-(4,4,5,5-tetramethyl-1,3,2-dioxaborolan-2-yl)hexan-3-yl)amino)-1-oxo-3-phenylpropan-2-yl)carbamate (**23**)

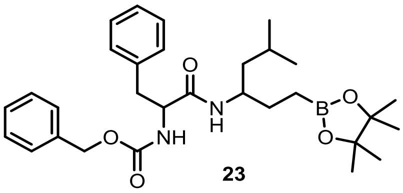

23

Prepared following General Procedure B with Z-Phe-Leu-OH (82 mg, 0.20 mmol, 1.0 equiv.), Ir[dF(CF$_3$)ppy]$_2$(dtbbpy)PF$_6$ (4.5 mg, 0.0040 mmol, 2.0 mol%), Cs_2CO_3 (65 mg, 0.20 mmol, 1.0 equiv.), vinyl boronic acid pinacol ester (51 µL, 0.30 mmol, 1.5 equiv.) and DMA (4.0 mL), which was irradiated with 2 × Kessil lamps for 62 h. Purification by flash column chromatography (15% EtOAc/pentane) gave the title compound (66 mg, 0.13 mmol, 63%) as a colourless oil. The diastereomeric ratio was determined to be 58:42 by ^{1}H NMR.

TLC: R_f = 0.19 (15% EtOAc/pentane, PMA stain).

^{1}H NMR (400 MHz, $CDCl_3$): 58:42 ratio of diastereomers: δ_H 7.36 – 7.19 (m, 10H), 5.50 – 5.42 (br. m, 2H), 5.11 – 5.04 (m, 2H), 4.37 – 4.30 (br. m, 1H), 3.92 – 3.87 (br. m, 1H), 3.13 – 2.97 (m, 2H), 1.58 – 1.24 (m, 3H), 1.24 – 1.21 (m, 12H), 1.21 – 0.99 (m, 2H), 0.85 (d, *J* = 6.7 Hz, 1.74H), 0.83 (d, *J* = 6.6 Hz, 1.26H), 0.82 (d, *J* = 6.7 Hz, 1.26H), 0.80 (d, *J* = 6.8 Hz, 1.74H), 0.67 (t, *J* = 8.1 Hz, 1.16H), 0.58 (ddd, *J* = 9.1, 6.8, 2.2 Hz, 0.84H) ppm.

^{13}C NMR (101 MHz, $CDCl_3$): Mixture of diastereomers: δ_C 170.0, 155.8, 136.6, 136.2, 129.39 (minor), 129.36 (major), 128.7, 128.53 (minor), 128.52 (major), 128.17 (minor), 128.15 (major), 128.02 (major), 128.00 (minor), 127.0 (minor), 126.9 (major), 83.12 (major), 83.06 (minor), 67.0, 56.6, 49.3 (major), 49.2 (minor), 44.1 (major), 43.9 (minor), 39.0 (major), 38.7 (minor), 29.5 (minor), 29.4 (major), 24.9, 24.81 (major), 24.75 (minor), 24.6, 23.1 (minor), 23.0 (major), 22.2, 7.0 (br.) ppm.

^{11}B NMR (128 MHz, $CDCl_3$): δ_B 33.7 (br. s, 1B) ppm.

IR (film) ν_{max}: 3301, 3089 – 2868, 1702, 1651, 1538, 1498, 1455, 1371, 1319, 1286, 1260, 1216, 1166, 1144, 1044, 1028 cm^{-1}.

HRMS (ESI$^+$) calcd. for $C_{30}H_{43}BN_2O_5Na$ [M+Na]$^+$ 545.3163, found 545.3166.

tert-Butyl 2-(2-(4,4,5,5-tetramethyl-1,3,2-dioxaborolan-2-yl)propyl)pyrrolidine-1-carboxylate (**24**)

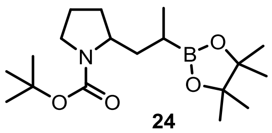

24

Prepared following General Procedure A with Boc-Pro-OH (65 mg, 0.30 mmol, 1.0 equiv.), Ir(ppy)$_2$(dtbbpy)PF$_6$ (2.7 mg, 0.0030 mmol, 1.0 mol%), Cs_2CO_3 (108 mg, 0.330 mmol, 1.10 equiv.), 2-propenyl boronic acid pinacol ester (85 μL, 0.45 mmol, 1.5 equiv.) and DMF (3.0 mL), which was irradiated with 1 × Kessil lamp for 30 h. Purification by flash column chromatography (10% EtOAc/pentane) gave the title compound (89 mg, 0.26 mmol, 87%) as a colourless oil. The diastereomeric ratio was determined to be 64:36 by high temperature NMR in DMSO-d_6.

TLC: R_f = 0.42 (10% EtOAc/pentane, $KMnO_4$ stain).

^{1}H NMR (500 MHz, DMSO-d_6, 100 °C): 64:36 ratio of diastereomers: δ_H 3.80 – 3.74 (m, 1H), 3.34 – 3.25 (m, 1H), 3.20 (dddd, J = 10.7, 7.6, 4.7, 1.6 Hz, 0.36H), 3.16 (dddd, J = 10.8, 7.2, 5.2, 1.6 Hz, 0.64H), 1.91 – 1.76 (m, 2H), 1.77 – 1.67 (m, 1H), 1.64 – 1.58 (m, 1H), 1.42 + 1.42 (diastereomeric peaks, 2 × s, 9H), 1.20 (s, 6H), 1.20 (s, 6H), 1.00 – 0.86 (m, 1H), 0.94 + 0.93 (diastereomeric peaks, 2 × s, 3H) ppm.

^{13}C NMR (126 MHz, DMSO-d_6, 100 °C): Mixture of diastereomers: δ_C 154.2, 83.08 (minor), 83.07 (major), 78.4, 57.0 (major), 56.6 (minor), 46.3 (minor), (major), 38.3 (major), 37.5 (minor), 30.7, 28.79 (major), 28.76 (minor), 25.1 (major), 25.02 (minor), 25.00 (minor), 24.99 (major), 23.3, 16.6 (major), 15.5 (minor) ppm. The carbon directly attached to boron was not detected due to the boron quadrupole.

^{11}B NMR (128 MHz, $CDCl_3$): δ_B 34.4 (br. s, 1B) ppm.

IR (film) ν_{max}: 2975 – 2873, 1691, 1459, 1387, 1364, 1315, 1247, 1166, 1144, 1105 cm^{-1}.

HRMS (ESI$^+$) calcd. for $C_{18}H_{35}BNO_4$ [M+H]$^+$ 340.2657, found 340.2660.

***tert*-Butyl 2-(1-(4,4,5,5-tetramethyl-1,3,2-dioxaborolan-2-yl)propan-2-yl)-pyrrolidine-1-carboxylate** (**25**)

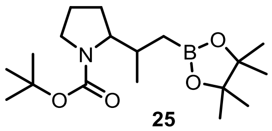

25

Prepared following General Procedure A with Boc-Pro-OH (65 mg, 0.30 mmol, 1.0 equiv.), $Ir(ppy)_2(dtbbpy)PF_6$ (2.7 mg, 0.0030 mmol, 1.0 mol%), Cs_2CO_3 (108 mg, 0.330 mmol, 1.10 equiv.), (*E*)-4,4,5,5-tetramethyl-2-(prop-1-en-1-yl)-1,3,2-dioxaborolane (85 µl, 0.45 mmol, 1.5 equiv.) and DMF (3.0 mL), which was irradiated with 1 × Kessil lamp for 62 h. Purification by flash column chromatography (10% EtOAc/pentane) gave the title compound (69 mg, 0.20 mmol, 68%) as a colourless oil. The diastereomeric ratio was determined to be 53:47 by high temperature NMR in DMSO-d_6.

TLC: R_f = 0.44 (10% EtOAc/pentane, $KMnO_4$ stain).

^{1}H NMR (500 MHz, DMSO-d_6, 100 °C): 53:47 ratio of diastereomers: δ_H 3.67 – 3.60 (m, 1H), 3.47 – 3.38 (m, 1H), 3.16 – 3.09 (m, 1H), 2.26 – 2.12 (m, 1H), 1.81 – 1.65 (m, 4H), 1.42 (minor, s, 9H), 1.42 (major, s, 9H), 1.20 (s, 12H), 0.86 (minor, d, *J* = 7.0 Hz, 3H), 0.78 (major, d, *J* = 6.7 Hz, 3H), 0.73 – 0.67 (m, 1H), 0.54 (major, dd, *J* = 15.4 Hz, 9.0 Hz, 1H), 0.43 (dd, *J* = 15.1 Hz, 9.9 Hz, 1H) ppm.

^{13}C NMR (126 MHz, DMSO-d_6, 100 °C): δ_C 153.60 (minor), 153.56 (major), 82.0, 77.4, 62.1 (minor), 62.0 (major), 46.3 (major), 46.2 (minor), 31.2 (major), 30.9 (minor), 27.72 (major), 27.68 (minor), 25.6 (minor), 25.4 (major), 24.1, 24.03 (major), 24.00 (minor), 23.0 (major), 22.9 (minor), 18.4 (major), 16.0 (minor) ppm. The carbon directly attached to boron was not detected due to the boron quadrupole.

^{11}B NMR (128 MHz, $CDCl_3$): δ_B 33.7 (br. s, 1B) ppm.

IR (film) ν_{max}: 2974 – 2874, 1724, 1689, 1478, 1389, 1364, 1316, 1164, 1143, 1104 cm^{-1}.

HRMS (ESI^+) calcd. for $C_{18}H_{35}BNO_4$ $[M+H]^+$ 340.2657, found 340.2671.

***tert*-Butyl 2-(3-(4,4,5,5-tetramethyl-1,3,2-dioxaborolan-2-yl)butan-2-yl)pyrrolidine-1-carboxylate** (**26**)

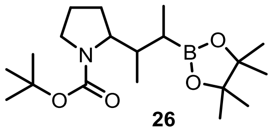

26

Prepared following General Procedure A with Boc-Pro-OH (65 mg, 0.30 mmol, 1.0 equiv.), Ir(ppy)$_2$(dtbbpy)PF$_6$ (2.7 mg, 0.0030 mmol, 1.0 mol%), Cs_2CO_3 (108 mg, 0.330 mmol, 1.10 equiv.), (*Z*)-2-(but-2-en-2-yl)-4,4,5,5-tetramethyl-1,3,2-dioxaborolane (92 µl, 0.45 mmol, 1.5 equiv.) and DMF (3.0 mL), which was irradiated with 1 × Kessil lamp for 24 h. Purification by flash column chromatography (10% EtOAc/pentane) gave the title compound (52 mg, 0.15 mmol, 49%) as a colourless oil. The product was formed as an undetermined mixture of diastereomers.

TLC: R_f = 0.50 (10% EtOAc/pentane, $KMnO_4$ stain).

^{1}H NMR (500 MHz, DMSO-*d*$_6$, 100 °C): Undetermined mixture of diastereomers: δ_H 3.93 – 3.88 + 3.83 – 3.76 (2 × m, 1H), 3.50 – 3.37 (m, 1H), 3.17 – 3.05 (m, 1H), 2.20 – 2.07 + 1.85 – 1.63 (2 × m, 5H), 1.42 (s, 9H), 1.21 + 1.20 (2 × s, 12H), 0.97 – 0.80 + 0.77 – 0.73 (2 × m, 7H) ppm.

^{13}C NMR (126 MHz, DMSO-*d*$_6$, 100 °C): Undetermined mixture of diastereomers: δ_C 153.7, 153.6, 153.3, 82.02, 81.98, 81.95, 77.5, 77.4, 60.5, 60.4, 60.2, 58.8, 46.6, 46.3, 45.6, 45.5, 36.7, 36.6, 36.5, 27.8, 27.7, 27.6, 25.8, 24.3, 24.2, 24.1, 24.04, 24.02, 23.98, 23.94, 23.20, 23.05, 22.50, 14.8, 14.1, 13.7, 13.1, 12.8, 12.1, 11.8 ppm. The carbon directly attached to boron was not detected due to the boron quadrupole.

^{11}B NMR (128 MHz, $CDCl_3$): δ_B 32.9 (br. s, 1B) ppm.

IR (film) ν_{max}: 2972 – 2838, 1724, 1687, 1611, 1511, 1454, 1379, 1365, 1244, 1173, 1143, 1105, 1033, 967 cm^{-1}.

HRMS (ESI$^+$) calcd. for $C_{19}H_{36}BNNaO_4$ [M+Na]$^+$ 376.2633, found 376.2640.

tert-Butyl 2-(2-(4,4,5,5-tetramethyl-1,3,2-dioxaborolan-2-yl)cyclohexyl)pyrrolidine-1-carboxylate (**27**)

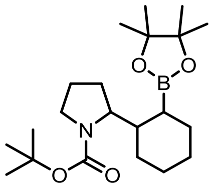

27

Prepared following General Procedure A with Boc-Pro-OH (65 mg, 0.30 mmol, 1.0 equiv.), Ir(ppy)$_2$(dtbbpy)PF$_6$ (2.7 mg, 0.0030 mmol, 1.0 mol%), Cs_2CO_3 (108 mg, 0.330 mmol, 1.10 equiv.), 1-cyclohexen-1-yl boronic acid pinacol ester (94 mg, 0.45 mmol, 1.5 equiv.) and DMF (3.0 mL), which was irradiated with 2 × Kessil lamps for 40 h. Purification by flash column chromatography (5% EtOAc/pentane) gave the title compound (32 mg, 0.084 mmol, 28%) as a colourless oil. The diastereomeric ratio was determined to be 44:33:19:4 by high temperature NMR in DMSO-d_6. Separation of the major diastereomer from this mixture was possible by flash column chromatography. The remaining three diastereomers were isolated as an inseparable mixture (59:33:8 d.r.).

TLC: R_f = 0.49 and 0.23 (5% EtOAc/pentane, $KMnO_4$ stain).

Major Diastereomer (>95:5 d.r.):

^{1}H NMR (500 MHz, DMSO-d_6, 100 ºC): δ_H 3.80 (br. s, 1H), 3.39 – 3.35 (m, 1H), 3.14 – 3.10 (m, 1H), 1.85 – 1.65 (m, 6H), 1.54 – 1.32 (m, 6H), 1.41 (s, 9H), 1.29 – 1.09 (m, 2H), 1.23 (s, 6H), 1.22 (s, 6H) ppm.

^{13}C NMR (126 MHz, DMSO-d_6, 100 ºC): δ_C 154.9, 82.7, 78.5, 61.3, 46.7, 44.7, 29.4, 28.7, 27.9, 26.8, 26.1, 25.3, 24.7, 24.1 ppm. The carbon directly attached to boron was not detected due to the boron quadrupole.

^{11}B NMR (128 MHz, $CDCl_3$): δ_B 33.9 (br. s, 1B) ppm.

Mixture of three minor diastereomers (59:33:8 d.r.):

^{1}H NMR (500 MHz, DMSO-d_6, 100 ºC): δ_H 3.99 – 3.95 (m, 0.33H), 3.78 – 3.74 (m, 0.59H), 3.66 – 3.63 (m, 0.08H), 3.47 – 3.40 (m, 0.92H), 3.40 – 3.35 (m, 0.08H), 3.16 – 3.11 (m, 0.08H), 3.09 – 3.03 (m, 0.92H), 1.97 (tdd, J = 11.6, 5.8, 3.3 Hz, 0.59H), 1.84 – 1.49 (m, 7.41H), 1.43 + 1.42 (2 × s, 9H), 1.37 – 1.28 (m, 2H), 1.23 + 1.20 + 1.29 (3 × s, 12H), 1.19 – 1.06 (m, 2.66H),

1.02 (qd, J = 11.6, 3.6 Hz, 0.08H), 0.91 (qd, J = 12.1, 3.6 Hz, 0.59H), 0.79 (td, J = 10.4, 2.9 Hz, 0.08H), 0.70 (td, J = 11.6, 3.0 Hz, 0.59H) ppm.

^{13}C NMR (126 MHz, DMSO-d_6, 100 °C): Only the two major diastereomers were observed: δ_C 155.0, 154.6, 83.0, 82.9, 78.5, 78.3, 61.2, 61.1, 47.5, 45.6, 43.9, 41.4, 28.9, 28.78, 28.76, 28.58, 28.55, 28.0, 27.23, 27.17, 27.1, 26.9, 26.4, 25.4, 25.2, 25.04, 24.98, 24.97, 24.6, 24.1, 23.2 ppm. The carbon directly attached to boron was not detected due to the boron quadrupole.

^{11}B NMR (128 MHz, $CDCl_3$): δ_B 34.1 (br. s, 1B) ppm.

IR (film) ν_{max}: 2974 – 2851, 1694, 1389, 1365, 1318, 1253, 1244, 1165, 1146, 1100 cm^{-1}.

HRMS (ESI$^+$) calcd. for $C_{21}H_{38}BNO_4$ [M+H]$^+$ 380.2971, found 380.2980.

***tert*-Butyl 2-(1-methoxy-3-(4,4,5,5-tetramethyl-1,3,2-dioxaborolan-2-yl)propan-2-yl)pyrrolidine-1-carboxylate** (**28**)

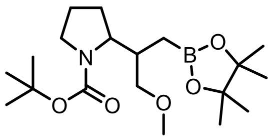

28

Prepared following General Procedure A using Boc-Pro-OH (65 mg, 0.30 mmol, 1.0 equiv.), Ir(ppy)$_2$(dtbbpy)PF_6 (2.7 mg, 0.0030 mmol, 1.0 mol%), Cs_2CO_3 (108 mg, 0.33 mmol, 1.1 equiv.), (*Z*)-2-(3-methoxyprop-1-en-1-yl)-4,4,5,5-tetramethyl-1,3,2-dioxaborolane (96 µL, 0.45 mmol, 1.5 equiv.) and DMF (3.0 mL), which was irradiated with 1 × Kessil lamp for 71 h. Purification by flash column chromatography (15% EtOAc/pentane) gave the title compound (64 mg, 0.17 mmol, 57% yield) as a colourless oil. The diastereomeric ratio was determined to be 54:46 by high temperature NMR in DMSO-d_6.

TLC: R_f = 0.22 (15% EtOAc/pentane, $KMnO_4$ stain).

^{1}H NMR (500 MHz, DMSO-d_6, 100 °C): 54:46 mixture of diastereomers: δ_H 3.85 – 3.77 (minor, m, 1H), 3.77 – 3.71 (major, m, 1H), 3.47 – 3.37 (m, 1H), 3.34 – 3.27 (m, 1H), 3.27 – 3.20 (m, 3H), 3.20 – 3.07 (m, 2H), 2.48 – 2.42 (minor, m, 1H), 2.31 – 2.22 (major, m, 1H), 1.86 – 1.63 (m, 4H), 1.43 (major, s, 9H), 1.42 (minor, s, 9H), 1.20 (major, s, 12H), 1.20 (minor, s, 12H), 0.76 – 0.62 (m, 1H), 0.62 – 0.55 (major, m, 1H), 0.52 – 0.44 (minor, m, 1H) ppm.

^{13}**C NMR** (126 MHz, DMSO-d_6, 100 °C): Mixture of diastereomers: 154.6 (major), 154.5 (minor), 83.0 (minor), 82.9 (major), 78.57 (minor), 78.55 (major), 76.0 (minor), 75.2 (major), 60.6 (major), 59.5 (minor), 58.5 (major), 58.4 (minor), 47.3 (minor), 47.2 (major), 38.2 (major), 37.3 (minor), 28.71 (major), 28.68 (minor), 28.0 (major), 26.6 (minor), 25.2 (major), 25.1 (minor), 25.04 (major), 25.01 (minor), 23.9 (minor), 23.8 (major) ppm. The carbon directly attached to boron was not detected due to the boron quadrupole.

^{11}**B NMR** (128 MHz, $CDCl_3$): δ_B 34.2 (br. s, 1B) ppm.

IR (film) ν_{max}: 2975, 2928, 2878, 1689, 1455, 1364, 1254, 1144, 1106 cm^{-1}.

HRMS (ESI$^+$): calcd. for $C_{19}H_{36}BNO_5Na$ [M+Na]$^+$ 392.2582, found 392.2591.

***tert*-Butyl 2-(1-((tert-butyldimethylsilyl)oxy)-3-(4,4,5,5-tetramethyl-1,3,2-dioxaborolan-2-yl)propan-2-yl)pyrrolidine-1-carboxylate** (**29**)

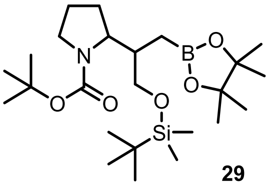

Prepared following General Procedure A using Boc-Pro-OH (65 mg, 0.30 mmol, 1.0 equiv.), Ir $(ppy)_2(dtbbpy)PF_6$ (2.7 mg, 0.0030 mmol, 1.0 mol%), Cs_2CO_3 (108 mg, 0.33 mmol, 1.1 equiv.), (*Z*)-*tert*-butyldimethyl((3-(4,4,5,5-tetramethyl-1,3,2-dioxaborolan-2-yl)allyl)oxy)silane (148 µL, 0.45 mmol, 1.5 equiv.) and DMF (3.0 mL), which was irradiated with 1 × Kessil lamp for 64 h. Purification by flash column chromatography (10% EtOAc/pentane) gave the title compound (75 mg, 0.16 mmol, 53% yield) as a colourless oil. The diastereomeric ratio was determined to be 58:42 by high temperature NMR in DMSO-d_6. The product was isolated as a mixture of diastereomers, although partial resolution of the diastereomeric products was observed by TLC.

TLC: R_f = 0.52 and 0.62 (10% EtOAc/pentane, $KMnO_4$ stain).

^{1}**H NMR** (500 MHz, DMSO-d_6, 100 ºC): 58:42 mixture of diastereomers: δ_H 3.92 – 3.86 (minor, m, 1H), 3.82 – 3.77 (major, m, 1H), 3.59 – 3.37 and 3.25 – 3.20 (2 × m, 3H), 3.19 – 3.09 (m, 1H), 2.41 – 2.34 (minor, m, 1H), 2.14 – 2.06 (major, m, 1H), 1.88 – 1.64 (m, 4H), 1.45 – 1.40 (m, 9H), 1.23 – 1.17 (m, 12H), 0.93 – 0.87 (m, 9H), 0.79 (major, dd, J = 15.8, 7.4 Hz,

1H), 0.67 (major, dd, J = 15.9, 7.2 Hz, 1H), 0.59 (minor, dd, J = 15.2, 5.2 Hz, 1H), 0.52 (minor, dd, J = 15.4, 8.6 Hz, 1H) 0.08 – 0.01 (m, 6H) ppm.

^{13}C NMR (126 MHz, DMSO-d_6, 100 °C): Mixture of diastereomers: δ_C 83.1 (minor), 83.0 (major), 78.5, 66.2 (minor), 65.2 (major), 60.5 (major), 59.3 (minor), 47.3 (major), 47.1 (minor), 39.7, 28.7 (major), 28.7 (minor), 28.1 (minor), 26.5 (major), 26.3, 25.20 (major), 25.15 (minor), 25.1 (major), 25.0 (minor), 23.9 (minor), 23.7 (major), 18.4, –5.0 (minor), –5.1 (major) ppm. Carbonyl carbon was not detected. The carbon directly attached to boron was not detected due to the boron quadrupole.

^{11}B NMR (128 MHz, $CDCl_3$): δ_B 34.6 (br. s, 1B) ppm.

IR (film) ν_{max}: 2975 – 2858, 1694, 1472, 1389, 1366, 1255, 1167, 1145, 1105 cm^{-1}.

HRMS (ESI$^+$): calcd. for $C_{24}H_{48}BNO_5SiNa$ [M+Na]$^+$ 492.3292, found 492.3284.

***tert*-Butyl 2-(2-phenyl-2-(4,4,5,5-tetramethyl-1,3,2-dioxaborolan-2-yl)ethyl)pyrrolidine-1-carboxylate (30)**

The reaction of Boc-Pro-OH (**1**) with 4,4,5,5-tetramethyl-2-(1-phenylvinyl)-1,3,2-dioxaborolane (**S1**) under General Procedure A gave none of the desired product **30**. Instead, the protodeboronation product (**S2**) was isolated in 69% yield (Scheme S1).

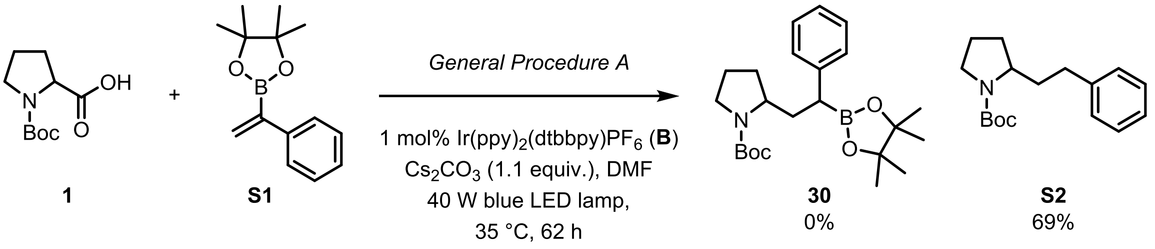

Scheme S1: Generation of **S2** by protodeboronation of **30**

Studies to determine the origin of this protodeboronation process revealed that the protodeboronation likely occurred after initial formation of **30**. Subjecting **S3** to the photochemical conditions in the absence of **1** and with only 0.5 equivalents of **S1** (to mimic the reaction conditions at full conversion) resulted in the formation of >99% of ethylbenzene (Scheme S2). The formation of ethylbenzene from **S3** suggests that protodeboronation to generate **S2** occurs after initial formation of the desired product **30**. Furthermore, the

observation that styrene was not formed under the reaction conditions disfavours the possibility of protodeboronation of **S1** followed by reaction of **1** with styrene.[9]

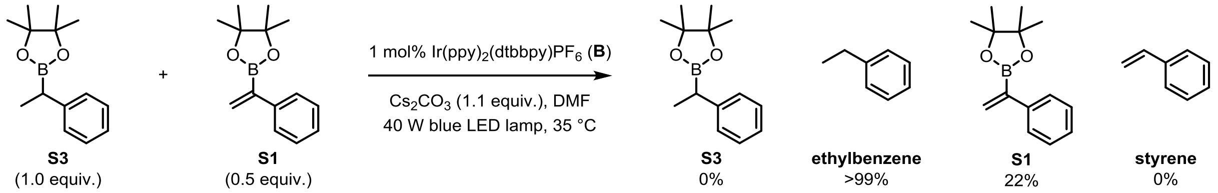

Scheme S2: Studies to determine the origin of protodeboronation product **S2**. Yields determined by GC using 1,2,4-trimethoxybenzene as an internal standard

The mechanism of the protodeboronation reaction is believed to proceed through a radical mechanism. Recent work by Ley and co-workers has shown that benzylic pinacol boronic esters, in the presence of a Lewis base to generate the corresponding boronate complex, undergo single electron oxidation under photoredox catalysis to cleave the C–B bond.[10] The resulting benzylic radical can then be trapped by an appropriate acceptor.

In the reaction shown in Scheme S2, transient boronate complexes could arise from the reaction of **S3** with Cs_2CO_3 or DMF. After photoredox-catalyzed oxidative formation of the benzylic radical, subsequent single electron reduction to a benzylic anion and protonation would provide ethylbenzene.[9]

***tert*-Butyl 2-phenethylpyrrolidine-1-carboxylate** (S2)

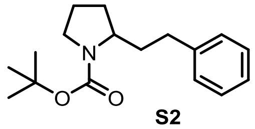

S2

Prepared following General Procedure A using Boc-Pro-OH (65 mg, 0.30 mmol, 1.0 equiv.), $Ir(ppy)_2(dtbbpy)PF_6$ (2.7 mg, 0.0030 mmol, 1.0 mol%), Cs_2CO_3 (108 mg, 0.330 mmol, 1.10 equiv.), 4,4,5,5-tetramethyl-2-(1-phenylvinyl)-1,3,2-dioxaborolane (104 mg, 0.450 mmol, 1.50 equiv.) and DMF (6.0 mL), which was irradiated with 2 × Kessil lamps for 62 h. Purification by flash column chromatography (10% EtOAc/pentane) gave the title compound (57 mg, 0.21 mmol, 69% yield) as a colourless oil.

---

[9] Lovett, G. H.; Sparling, B. A. *Org. Lett.* **2016**, *18*, 3494.
[10] (a) Lima, F.; Kabeshov, M. A.; Tran, D. N.; Battilocchio, C.; Sedelmeier, J.; Sedelmeier, G.; Schenkel, B.; Ley, S. V. *Angew. Chem. Int. Ed.* **2016**, *55*, 14085. (b) Lima, F.; Sharma, U. K.; Grunenberg, L.; Saha, D.; Johannsen, S.; Sedelmeier, J.; Van der Eycken, E. V.; Ley, S. V. *Angew. Chem. Int. Ed.* **2017**, *56*, 15136.

^{1}H NMR (400 MHz, $CDCl_3$): δ_H 7.30 – 7.07 (m, 5H), 3.92 – 3.63 (br. m, 1H), 3.44 – 3.17 (br. m, 2H), 2.67 – 2.45 (br. m, 2H), 2.20 – 1.48 (m, 6H), 1.41 (s, 9H) ppm.

^{13}C NMR (101 MHZ, $CDCl_3$): δ_C 154.7, 142.1, 128.3, 125.8, 79.0, 57.3 + 57.0 (rotameric peaks), 46.5 + 46.2 (rotameric peaks), 36.4 + 36.0 (rotameric peaks), 32.8, 30.6 + 30.1 (rotameric peaks), 28.6, 23.8 + 23.2 (rotameric peaks) ppm.

All spectroscopic data are consistent with previous literature reports.[9]

2-(2-Cyclopentylethyl)-4,4,5,5-tetramethyl-1,3,2-dioxaborolane (**31**)

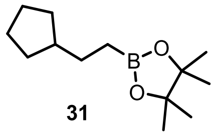

31

Prepared following General Procedure C with cyclopentanecarboxylic acid (22 μL, 0.20 mmol, 1.0 equiv.), Ir[dF(Me)ppy]$_2$(dtbbpy)PF_6 (4.1 mg, 0.0040 mmol, 2.0 mol%), Cs_2CO_3 (65 mg, 0.20 mmol, 1.0 equiv.), vinyl boronic acid pinacol ester (51 μL, 0.30 mmol, 1.5 equiv.) and DMA (4.0 mL), which was irradiated with 2 × Kessil lamps for 62 h. Purification by flash column chromatography (2% EtOAc/pentane) gave the title compound (11 mg, 0.049 mmol, 25%) as a colourless oil.

TLC: R_f = 0.46 (2% EtOAc/pentane, $KMnO_4$ stain).

^{1}H NMR (400 MHz, $CDCl_3$): δ_H 1.77 – 1.64 (m, 3H), 1.62 – 1.45 (m, 4H), 1.43 – 1.37 (m, 2H), 1.24 (s, 12H), 1.11 – 1.02 (m, 2H), 0.79 – 0.75 (m, 2H) ppm.

^{13}C NMR (101 MHz, $CDCl_3$): δ_C 82.8, 42.6, 32.3, 30.2, 25.2, 24.8 ppm. The carbon directly attached to boron was not detected due to the boron quadrupole.

^{11}B NMR (128 MHz, $CDCl_3$): δ_B 34.5 (br. s, 1B) ppm.

All spectroscopic data are consistent with previous literature reports.[11]

---

[11] Hu, D.; Wang, L.; Li, P. *Org. Lett.* **2017**, *19*, 2770.

2-(2-Cyclohexylethyl)-4,4,5,5-tetramethyl-1,3,2-dioxaborolane (**32**)

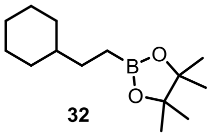

32

Prepared following General Procedure C with cyclohexanecarboxylic acid (26 mg, 0.20 mmol, 1.0 equiv.), Ir[dF(Me)ppy)$_2$(dtbbpy]PF_6 (4.1 mg, 0.0040 mmol, 2.0 mol%), Cs_2CO_3 (65 mg, 0.20 mmol, 1.0 equiv.), vinyl boronic acid pinacol ester (51 µl, 0.30 mmol, 1.5 equiv.) and DMA (4.0 mL), which was irradiated with 2 × Kessil lamps for 62 h. Purification by flash column chromatography (2% EtOAc/pentane) gave the title compound (23 mg, 0.097 mmol, 48%) as a colourless oil.

TLC: R_f = 0.76 (4% EtOAc/pentane, $KMnO_4$ stain).

^{1}H NMR (500 MHz, $CDCl_3$): δ_H 1.74 - 1.58 (m, 5H), 1.31 – 1.25 (m, 2H), 1.24 (s, 12H), 1.23 – 1.07 (m, 4H), 0.90 – 0.77 (m, 2H), 0.73 – 0.66 (br. m, 2H) ppm.

^{13}C NMR (126 MHz, $CDCl_3$): δ_C 83.0, 41.7, 34.3, 32.2, 28.8, 26.7, 25.0, 8.5 (br.) ppm.

^{11}B NMR (128 MHz, $CDCl_3$): δ_B 33.1 (br. s, 1B) ppm.

IR (film) ν_{max}: 2977 – 2850, 1449, 1371, 1316, 1145, 968 cm^{-1}.

HRMS (ESI$^+$) calcd. for $C_{14}H_{27}BNaO_2$ $[M+Na]^+$ 261.1999, found 261.1991.

2-(2-Cycloheptylethyl)-4,4,5,5-tetramethyl-1,3,2-dioxaborolane (**33**)

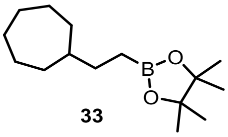

33

Prepared following General Procedure C with cycloheptanecarboxylic acid (28 mg, 0.20 mmol, 1.0 equiv.), Ir[dF(Me)ppy]$_2$(dtbbpy)PF_6 (4.1 mg, 0.0040 mmol, 2.0 mol%), Cs_2CO_3 (65 mg, 0.20 mmol, 1.0 equiv.), vinyl boronic acid pinacol ester (51 µl, 0.30 mmol, 1.5 equiv.) and DMA (4.0 mL), which was irradiated with 2 × Kessil lamps for 62 h. Purification by flash column chromatography (2% EtOAc/pentane) gave the title compound (32 mg, 0.13 mmol, 63%) as a colourless oil.

TLC: R_f = 0.76 (4% EtOAc/pentane, $KMnO_4$ stain).

^{1}H NMR (400 MHz, $CDCl_3$): δ_H 1.70 – 1.30 (m, 13H), 1.23 (s, 12H), 1.17 – 1.07 (m, 2H), 0.78 – 0.71 (br. m, 2H) ppm.

^{13}C NMR (101MHz, $CDCl_3$): δ_C 83.0, 41.7, 34.3, 32.2, 28.8, 26.7, 25.0, 9.2 (br.) ppm.

^{11}B NMR (128 MHz, $CDCl_3$): δ_B 33.4 (br. s, 1B) ppm.

IR (film) ν_{max}: 2977 – 2852, 1459, 1370, 1314, 1144, 967, 847 cm^{-1}.

HRMS (ESI$^+$) calcd. for $C_{15}H_{29}BNaO_2$ $[M+Na]^+$ 275.2156, found 275.2144.

2-(2-Cyclododecylethyl)-4,4,5,5-tetramethyl-1,3,2-dioxaborolane (**34**)

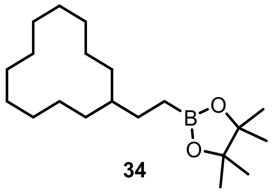

34

Prepared following General Procedure C with cyclododecanecarboxylic acid (42 mg, 0.20 mmol, 1.0 equiv.), Ir[dF(Me)ppy]$_2$(dtbbpy)PF_6 (4.1 mg, 0.0040 mmol, 2.0 mol%), Cs_2CO_3 (65 mg, 0.20 mmol, 1.0 equiv.), vinyl boronic acid pinacol ester (51 µL, 0.30 mmol, 1.5 equiv.) and DMA (2.0 mL), which was irradiated with 2 × Kessil lamps for 62 h. Purification by flash column chromatography (5% EtOAc/pentane) gave the title compound (43 mg, 0.13 mmol, 67%) as a colourless oil.

TLC: R_f = 0.77 (5% EtOAc/pentane, $KMnO_4$ stain).

^{1}H NMR (400 MHz, $CDCl_3$): δ_H 1.39 – 1.24 (m, 25H), 1.24 (s, 12H), 0.78 – 0.74 (m, 2H) ppm.

^{13}C NMR (101 MHz, $CDCl_3$): δ_C 82.8, 36.0, 29.0, 28.8, 24.8, 24.7, 24.0, 23.7, 23.51, 23.45, 21.9 ppm. The carbon directly attached to boron was not detected due to the boron quadrupole.

^{11}B NMR (128 MHz, $CDCl_3$): δ_B 34.2 (br. s, 1B) ppm.

IR (film) ν_{max}: 2977 – 2848, 1470, 1445, 1370, 1315, 1145, 969 cm^{-1}.

HRMS (ESI$^+$) calcd. for $C_{20}H_{40}BO_2$ $[M+H]^+$ 323.3120, found 323.3128.

4,4,5,5-Tetramethyl-2-(3-methyloctyl)-1,3,2-dioxaborolane (**35**)

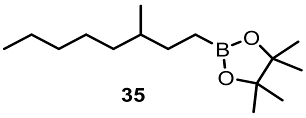

35

Prepared following General Procedure C with 2-methylheptanoic acid (32 μL, 0.20 mmol, 1.0 equiv.), Ir[dF(Me)ppy]$_2$(dtbbpy)PF$_6$ (4.1 mg, 0.0040 mmol, 2.0 mol%), Cs_2CO_3 (65 mg, 0.20 mmol, 1.0 equiv.), vinyl boronic acid pinacol ester (51 μL, 0.30 mmol, 1.5 equiv.) and DMA (2.0 mL), which was irradiated with 1 × Kessil lamp for 62 h. Purification by flash column chromatography (5% EtOAc/pentane) gave the title compound (33 mg, 0.13 mmol, 65%) as a colourless oil.

TLC: R_f = 0.78 (5% EtOAc/pentane, $KMnO_4$ stain).

^{1}H NMR (400 MHz, $CDCl_3$): δ_H 1.41 (dddd, *J* = 13.1, 10.2, 6.4, 4.9 Hz, 1H), 1.35 – 1.16 (m, 8H), 1.24 (s, 12H), 1.12 – 1.01 (m, 1H), 0.87 (t, *J* = 7.0 Hz, 3H), 0.83 (d, *J* = 6.4 Hz, 3H), 0.82 – 0.67 (m, 2H) ppm.

^{13}C NMR (101 MHz, $CDCl_3$): δ_C 82.8, 36.6, 34.9, 32.2, 31.0, 26.8, 24.82, 24.80, 22.7, 19.2, 14.1, 8.4 (br.) ppm.

^{11}B NMR (128 MHz, $CDCl_3$): δ_B 34.2 (br. s, 1B) ppm.

IR (film) ν_{max}: 2978 – 2858, 1466, 1405, 1378, 1371, 1318, 1272, 1214, 1165, 1145 cm^{-1}.

HRMS (ESI$^+$) calcd. for $C_{15}H_{32}BO_2$ [M+H]$^+$ 255.2492, found 255.2492.

4,4,5,5-Tetramethyl-2-(2-(tetrahydrofuran-2-yl)ethyl)-1,3,2-dioxaborolane (**36**)

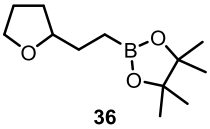

36

Prepared following General Procedure B with tetrahydrofuran-2-carboxylic acid (35 mg, 0.30 mmol, 1.0 equiv.), Ir[dF(CF_3)ppy]$_2$(dtbbpy)PF$_6$ (6.7 mg, 0.0060 mmol, 2.0 mol%), Cs_2CO_3 (98 mg, 0.30 mmol, 1.0 equiv.), vinyl boronic acid pinacol ester (76 μL, 0.45 mmol, 1.5 equiv.) and DMA (3.0 mL), which was irradiated with 1 × Kessil lamp for 72 h. Purification by flash

column chromatography (10% EtOAc/pentane) gave the title compound (42 mg, 0.19 mmol, 62%) as a colourless oil.

TLC: R_f = 0.54 (10% EtOAc/pentane, $KMnO_4$ stain).

^{1}H NMR (400 MHz, $CDCl_3$): δ_H 3.86 – 3.68 (m, 3H), 1.99 – 1.77 (m, 3H), 1.71 – 1.54 (m, 2H), 1.52 – 1.40 (m, 1H), 1.24 (s, 12H), 0.88 – 0.72 (m, 2H) ppm.

^{13}C NMR (101 MHz, $CDCl_3$): δ_C 83.1, 81.0, 67.8, 31.0, 30.0, 26.0, 24.98, 24.95 ppm. The carbon directly attached to boron was not detected due to the boron quadrupole.

^{11}B NMR (128 MHz, $CDCl_3$): δ_B 32.4 (br. s, 1B) ppm.

IR (film) ν_{max}: 2975 – 2868, 1440, 1368, 1317, 1272, 1144, 1055, 968, 847 cm^{-1}.

HRMS (ESI$^+$) calcd. for $C_{12}H_{24}BO_3$ $[M+H]^+$ 227.1815, found 227.1820.

4,4,5,5-Tetramethyl-2-(3-phenoxybutyl)-1,3,2-dioxaborolane (**37**)

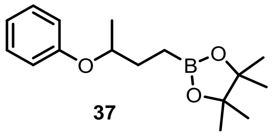

37

Prepared following General Procedure B with 2-phenoxypropionic acid (50 mg, 0.30 mmol, 1.0 equiv.), Ir[dF(CF_3)ppy]$_2$(dtbbpy)PF_6 (6.7 mg, 0.0060 mmol, 2.0 mol%), Cs_2CO_3 (97 mg, 0.30 mmol, 1.0 equiv.), vinyl boronic acid pinacol ester (76 µL, 0.45 mmol, 1.5 equiv.) and DMA (3.0 mL), which was irradiated with 2 × Kessil lamps for 40 h. Purification by flash column chromatography (2% EtOAc/pentane) gave the title compound (39 mg, 0.14 mmol, 47%) as a colourless oil.

TLC: R_f = 0.30 (5% EtOAc/pentane, $KMnO_4$ stain).

^{1}H NMR (400 MHz, $CDCl_3$): δ_H 7.28 – 7.22 (m, 2H), 6.91 – 6.88 (m, 3H), 4.32 (h, *J* = 6.1 Hz, 1H), 1.85 (ddt, *J* = 13.2, 9.6, 6.5 Hz, 1H), 1.68 (ddt, *J* = 13.8, 9.4, 6.2 Hz, 1H), 1.28 (d, *J* = 6.1 Hz, 3H), 1.23 (s, 6H), 1.23 (s, 6H), 9.96 – 0.82 (m, 1H) ppm.

^{13}C NMR (101 MHz, $CDCl_3$): δ_C 158.4, 129.4, 120.3, 116.1, 83.0, 75.3, 30.6, 24.9, 24.8, 19.4, 7.2 (br.) ppm.

^{11}B NMR (128 MHz, $CDCl_3$): δ_B 34.1 (br. s, 1B) ppm.

IR (film) ν_{max}: 3094–2874, 1600, 1586, 1494, 1379, 1372, 1322, 1301, 1242, 1167, 1145 cm^{-1}.

HRMS (ESI$^+$) calcd. for $C_{16}H_{26}BO_3$ [M+H]$^+$ 277.1972, found 277.1975.

4,4,5,5-Tetramethyl-2-(2-(1-methylcyclohexyl)ethyl)-1,3,2-dioxaborolane (**38**)

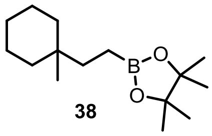

38

Prepared following General Procedure C with 1-methylcyclohexane-1-carboxylic acid (28 mg, 0.20 mmol, 1.0 equiv.), Ir[dF(Me)ppy]$_2$(dtbbpy)PF$_6$ (4.1 mg, 0.0040 mmol, 2.0 mol%), Cs_2CO_3 (65 mg, 0.20 mmol, 1.0 equiv.), vinyl boronic acid pinacol ester (51 μL, 0.30 mmol, 1.5 equiv.) and DMA (2.0 mL), which was irradiated with 2 × Kessil lamps for 62 h. Purification by flash column chromatography (5% EtOAc/pentane) gave the title compound (38 mg, 0.15 mmol, 75%) as a colourless oil.

TLC: R_f = 0.74 (5% EtOAc/pentane, $KMnO_4$ stain).

^{1}H NMR (400 MHz, $CDCl_3$): δ_H 1.47 – 1.35 (m, 5H), 1.34 – 1.25 (m, 3H), 1.24 (s, 12H), 1.21 – 1.18 (m, 4H), 0.80 (s, 3H), 0.69 – 0.65 (m, 2H) ppm.

^{13}C NMR (101 MHz, $CDCl_3$): δ_C 82.8, 37.3, 36.0, 33.0, 26.7, 24.8, 24.1, 22.2, 4.4 (br.) ppm.

^{11}B NMR (128 MHz, $CDCl_3$): δ_B 34.5 (br. s, 1B) ppm.

IR (film) ν_{max}: 2977 – 2850, 1447, 1402, 1370, 1313, 1272, 1145, 968 cm^{-1}.

HRMS (ESI$^+$) calcd. for $C_{15}H_{30}BO_2$ [M+H]$^+$ 253.2336, found 253.2340.

4,4,5,5-Tetramethyl-2-(2-(4-pentylbicyclo[2.2.2]octan-1-yl)ethyl)-1,3,2-dioxaborolane (39)

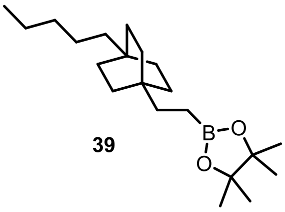

39

Prepared following General Procedure C with 4-pentylbicyclo[2.2.2]octane-1-carboxylic acid (45 mg, 0.20 mmol, 1.0 equiv.), Ir[dF(Me)ppy]$_2$(dtbbpy)PF_6 (4.1 mg, 0.0040 mmol, 2.0 mol%), Cs_2CO_3 (65 mg, 0.20 mmol, 1.0 equiv.), vinyl boronic acid pinacol ester (51 μL, 0.30 mmol, 1.5 equiv.) and DMA (4.0 mL), which was irradiated with 2 × Kessil lamps for 62 h. Purification by flash column chromatography (5% EtOAc/pentane) gave the title compound (31 mg, 0.093 mmol, 46%) as a colourless oily solid.

TLC: R_f = 0.72 (5% EtOAc/pentane, $KMnO_4$ stain).

^{1}H NMR (400 MHz, $CDCl_3$): δ_H 1.29 (s, 12H), 1.29 – 1.23 (m, 2H), 1.23 (s, 12H), 1.20 – 1.10 (m, 6H), 1.03 – 0.99 (m, 2H), 0.86 (t, *J* = 7.2 Hz, 2H), 0.64 – 0.60 (m, 2H) ppm.

^{13}C NMR (101 MHz, $CDCl_3$): δ_C 82.8, 41.8, 35.2, 32.9, 31.4, 31.2, 30.9, 30.7, 24.8, 23.4, 22.7, 14.1, 4.9 (br.) ppm.

^{11}B NMR (128 MHz, $CDCl_3$): δ_B 34.6 (br. s, 1B) ppm.

IR (film) ν_{max}: 2977 – 2856, 1455, 1404, 1372, 1336, 1316, 1146, 970 cm^{-1}.

HRMS (ESI$^+$) calcd. for $C_{21}H_{40}BO_2$ [M+H]$^+$ 335.3120, found 335.3116.

2-(2-((Adamantan-1-yl)ethyl)-4,4,5,5-tetramethyl-1,3,2-dioxaborolane (40)

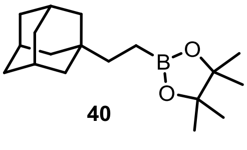

40

Prepared following General Procedure C with 1-adamantanecarboxylic acid (36 mg, 0.20 mmol, 1.0 equiv.), Ir[dF(Me)ppy]$_2$(dtbbpy)PF_6 (4.1 mg, 0.0040 mmol, 2.0 mol%), Cs_2CO_3 (65 mg, 0.20 mmol, 1.0 equiv.), vinyl boronic acid pinacol ester (51 μL, 0.30 mmol, 1.5 equiv.) and

DMA (4.0 mL), which was irradiated with 2 × Kessil lamps for 62 h. Purification by flash column chromatography (5% EtOAc/pentane) gave the title compound (28 mg, 0.096 mmol, 48%) as an oily colourless solid.

TLC: R_f = 0.68 (5% EtOAc/pentane, $KMnO_4$ stain).

^{1}H NMR (400 MHz, $CDCl_3$): δ_H 1.92 (p, *J* = 3.2 Hz, 3H), 1.68 (dt, *J* = 12.2, 2.9 Hz, 3H), 1.62 – 1.57 (m, 3H), 1.42 (d, *J* = 2.7 Hz, 6H), 1.24 (s, 12H), 1.19 – 1.14 (m, 2H), 0.70 – 0.65 (m, 2H) ppm.

^{13}C NMR (101 MHz, $CDCl_3$): δ_C 82.8, 41.9, 38.1, 37.3, 32.6, 28.8, 24.8, 3.4 (br.) ppm.

^{11}B NMR (128 MHz, $CDCl_3$): δ_B 34.4 (br. s, 1B) ppm.

IR (film) ν_{max}: 2977 – 2845, 1451, 1402, 1370, 1346, 1326, 1309, 1145, 967 cm^{-1}.

HRMS (ESI^+) calcd. for $C_{18}H_{32}BO_2$ $[M+H]^+$ 291.2493, found 291.2493.

2-(3,3-dimethylheptyl)-4,4,5,5-tetramethyl-1,3,2-dioxaborolane (**41**)

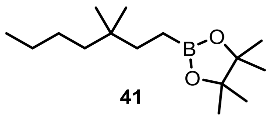

41

Prepared following General Procedure C using 2,2-dimethylhexanoic acid (32 μL, 0.20 mmol, 1.0 equiv.), Ir[dF(Me)ppy]$_2$(dtbbpy)PF_6 (4.1 mg, 0.0040 mmol, 2.0 mol%), Cs_2CO_3 (65 mg, 0.20 mmol, 1.0 equiv.), vinyl boronic acid pinacol ester (51 μL, 0.30 mmol, 1.5 equiv.) and DMA (2.0 mL), which was irradiated with 1 × Kessil lamps for 64 h. Purification by flash column chromatography (1% EtOAc/pentane) gave the title compound (34 mg, 0.13 mmol, 67% yield) as a yellow oil.

TLC: R_f = 0.57 (100% pentane, $KMnO_4$ stain).

^{1}H NMR (400 MHz, $CDCl_3$): δ_H 1.32 – 1.10 (m, 8H), 1.24 (s, 6H), 1.24 (s, 6H) 0.88 (t, *J* = 7.0 Hz, 3H), 0.79 (s, 6H), 0.71 – 0.63 (m, 2H) ppm.

^{13}C NMR (101 MHZ, $CDCl_3$): δ_C 82.8, 41.2, 35.6, 33.0, 26.6, 26.3, 24.8, 23.7, 14.2, 5.1 (br.) ppm.

^{11}B NMR (128 MHz, $CDCl_3$): δ_B 34.8 (br. s, 1B) ppm.

IR (film) ν_{max}: 2957, 2929, 2862, 1469, 1370, 1330, 1315, 1146 cm^{-1}.

HRMS (MALDI) calcd. for $C_{15}H_{31}BNaO_2$ $[M+Na]^+$ 277.2312, found 277.2318.

2-(2-((1*R*,4a*S*,10a*S*)-7-Isopropyl-1,4a-dimethyl-1,2,3,4,4a,9,10,10a-octahydrophenanthren-1-yl)ethyl)-4,4,5,5-tetramethyl-1,3,2-dioxaborolane (**42**)

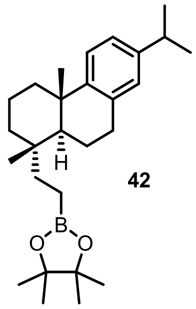

42

Prepared following General Procedure C with dehydroabietic acid (60 mg, 0.20 mmol, 1.0 equiv.), Ir[dF(Me)ppy]$_2$(dtbbpy)PF$_6$ (4.1 mg, 0.0040 mmol, 2.0 mol%), Cs_2CO_3 (65 mg, 0.20 mmol, 1.0 equiv.), vinyl boronic acid pinacol ester (51 µL, 0.30 mmol, 1.5 equiv.) and DMA (2.0 mL), which was irradiated with 1 × Kessil lamp for 62 h. Purification by flash column chromatography (1% EtOAc/pentane) gave the title compound (52 mg, 0.13 mmol, 63%) as a colourless oil. The diastereomeric ratio was determined to be >95:5 by ^{1}H NMR. The relative configuration was determined by NOESY.

TLC: $R_f = 0.22$ (1% EtOAc/pentane, PMA stain).

Optical rotation: $[\alpha]_D^{23}$ +32.0 (*c* 1.0, $CHCl_3$).

^{1}H NMR (500 MHz, $CDCl_3$): δ_H 7.17 (d, *J* = 8.1 Hz, 1H), 6.98 (dd, *J* = 8.1, 2.0 Hz, 1H), 6.88 (d, *J* = 2.1 Hz, 1H), 2.88 – 2.85 (m, 2H), 2.82 (hept, *J* = 6.9 Hz, 1H), 2.25 (dt, *J* = 12.6, 3.4 Hz, 1H), 1.84 – 1.77 (m, 1H), 1.74 (tt, *J* = 13.6, 3.6 Hz, 1H), 1.69 – 1.60 (m, 2H), 1.43 (dd, *J* = 12.3, 2.0 Hz, 1H), 1.41 – 1.32 (m, 4H), 1.26 – 1.20 (m, 1H), 1.25 (s, 12H), 1.23 (d, *J* = 6.9 Hz, 6H), 1.20 (s, 3H), 0.90 (s, 3H), 0.72 – 0.57 (m, 2H) ppm.

^{13}C NMR (126 MHz, $CDCl_3$): δ_C 147.8, 145.3, 135.0, 126.8, 124.3, 123.7, 82.9, 47.1, 38.7, 37.7, 37.6, 36.5, 35.9, 33.4, 30.4, 25.4, 24.8, 24.02, 24.00, 20.5, 19.1, 18.6, 4.4 (br.) ppm.

^{11}B NMR (128 MHz, $CDCl_3$): δ_B 35.3 (br. s, 1B) ppm.

IR (film) ν_{max}: 2957–2867, 1456, 1400, 1378, 1370, 1363, 1331, 1321, 1310, 1165, 1145 cm^{-1}.

HRMS (ESI$^+$) calcd. for $C_{27}H_{44}BO_2$ $[M+H]^+$ 411.3434, found 411.3430.

2,5,7,8-Tetramethyl-2-(2-(4,4,5,5-tetramethyl-1,3,2-dioxaborolan-2-yl)ethyl)chroman-6-ol (**43**)

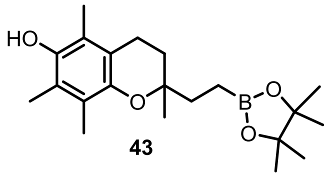

43

Prepared following General Procedure B with 6-hydroxy-2,5,7,8-tetramethylchromane-2-carboxylic acid (Trolox) (75 mg, 0.30 mmol, 1.0 equiv.), Ir[dF(CF$_3$)ppy]$_2$(dtbbpy)PF$_6$ (6.7 mg, 0.0060 mmol, 2.0 mol%), Cs_2CO_3 (97 mg, 0.30 mmol, 1.0 equiv.), vinyl boronic acid pinacol ester (76 µL, 0.45 mmol, 1.5 equiv.) and DMA (6.0 mL), which was irradiated with 2 × Kessil lamps for 62 h. Purification by flash column chromatography (5-10% EtOAc/pentane) gave the title compound (59 mg, 0.16 mmol, 55%) as a colourless solid.

TLC: R_f = 0.20 (5% EtOAc/pentane, $KMnO_4$ stain).

Mpt: 105–107 ºC (CH_2Cl_2).

^{1}H NMR (500 MHz, $CDCl_3$): δ_H 4.21 (s, 1H), 2.66 – 2.55 (m, 2H), 2.15 (s, 3H), 2.11 (s, 6H), 1.84 – 1.70 (m, 3H), 1.66 – 1.60 (m, 1H), 1.25 (s, 12H), 1.20 (s, 3H), 0.94 – 0.84 (m, 2H) ppm.

^{13}C NMR (126 MHz, $CDCl_3$): δ_C 145.6, 144.5, 122.7, 121.0, 118.5, 117.4, 83.0, 74.8, 33.6, 31.1, 24.9, 24.8, 23.1, 20.8, 12.2, 11.8, 11.3, 4.9 (br.) ppm.

^{11}B NMR (128 MHz, $CDCl_3$): δ_B 34.1 (br. s, 1B) ppm.

IR (film) ν_{max}: 3456, 2977 – 2871, 1452, 1418, 1372, 1318, 1261, 1215, 1164, 1143, 1108, 1084 cm^{-1}.

HRMS (ESI$^+$) calcd. for $C_{21}H_{34}BO_4$ $[M+H]^+$ 361.2549, found 361.2559.

2,5,7,8-Tetramethyl-2-(2-(4,4,5,5-tetramethyl-1,3,2-dioxaborolan-2-yl)ethyl)chroman-6-yl acetate (**44**)

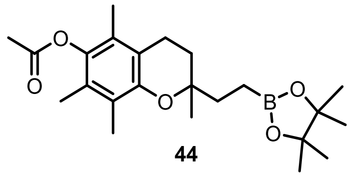

44

Prepared following General Procedure B with 6-acetoxy-2,5,7,8-tetramethylchromane-2-carboxylic acid (88 mg, 0.30 mmol, 1.0 equiv.), Ir[dF(CF_3)ppy]$_2$(dtbbpy)PF_6 (6.7 mg, 0.0060 mmol, 2.0 mol%), Cs_2CO_3 (97 mg, 0.30 mmol, 1.0 equiv.), vinyl boronic acid pinacol ester (76 μL, 0.45 mmol, 1.5 equiv.) and DMA (3.0 mL), which was irradiated with 2 × Kessil lamps for 62 h. Purification by flash column chromatography (5-10% EtOAc/pentane) gave the title compound (67 mg, 0.17 mmol, 56%) as a colourless solid.

TLC: R_f = 0.32 (5% EtOAc/pentane, $KMnO_4$ stain).

Mpt: 116–118 °C (CH_2Cl_2).

^{1}H NMR (500 MHz, $CDCl_3$): δ_H 2.64 – 2.53 (m, 2H), 2.32 (s, 3H), 2.08 (s, 3H), 2.01 (s, 3H), 1.97 (s, 3H), 1.83 – 1.70 (m, 3H), 1.68 –1.61 (m, 1H), 1.25 (s, 12H), 1.21 (s, 3H), 0.93 – 0.83 (m, 2H) ppm.

^{13}C NMR (126 MHz, $CDCl_3$): δ_C 169.8, 149.4, 140.5, 126.6, 124.9, 123.1, 117.4, 83.1, 75.3, 34.0 + 33.4 (rotameric peaks), 30.6, 24.87, 24.85, 23.6 + 23.1 (rotameric peaks), 20.58, 20.58, 13.0, 12.1, 11.9, 4.9 (br.) ppm.

^{11}B NMR (128 MHz, $CDCl_3$): δ_B 34.2 (br. s, 1B) ppm.

IR (film) ν_{max}: 2977 – 2871, 1754, 1369, 1318, 1205, 1164, 1164, 1144, 1108, 1075 cm^{-1}.

HRMS (ESI$^+$) calcd. for $C_{23}H_{36}BO_5$ [M+H]$^+$ 403.2655, found 403.2651.

4-Chloro-N-(4-((2-methyl-4-(4,4,5,5-tetramethyl-1,3,2-dioxaborolan-2-yl)butan-2-yl)oxy)phenethyl)benzamide (**45**)

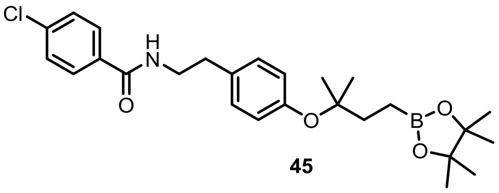

45

Prepared following General Procedure B with bezafibrate (109 mg, 0.300 mmol, 1.00 equiv.), Ir[dF(CF_3)ppy]$_2$(dtbbpy)PF_6 (6.7 mg, 0.0060 mmol, 2.0 mol%), Cs_2CO_3 (98 mg, 0.30 mmol, 1.0 equiv.), vinyl boronic acid pinacol ester (76 µl, 0.45 mmol, 1.5 equiv.) and DMA (3.0 mL), which was irradiated with 2 × Kessil lamps for 62 h. Purification by flash column chromatography (20% EtOAc/pentane) gave the title compound (56 mg, 0.12 mmol, 40%) as an amorphous solid.

TLC: R_f = 0.39 (20% EtOAc/pentane, $KMnO_4$ stain).

^{1}H NMR (400 MHz, $CDCl_3$): δ_H 7.72 – 7.63 and 7.62 – 7.57 (rotameric peaks, 2 × m, 2H), 7.40 – 7.33 (m, 2H), 7.10 – 7.05 (m, 2H), 6.95 – 6.91 (m, 2H), 6.20 – 6.10 (br. m, 1H), 3.70 – 3.64 (m, 2H), 2.89 – 2.84 (m, 2H), 1.78 – 1.74 (m, 2H), 1.22 (s, 18H), 0.96 - 0.92 (m, 2H) ppm.

^{13}C NMR (101 MHz, $CDCl_3$): δ_C 167.6 + 166.5 (rotameric peaks), 154.3 + 154.2 (rotameric peaks), 137.7 + 134.8 (rotameric peaks), 133.5 + 133.3 (rotameric peaks), 133.19 + 133.17 (rotameric peaks), 129.25 + 129.23 (rotameric peaks), 128.9 + 128.7 (rotameric peaks), 128.4 + 126.9 (rotameric peaks), 125.2 + 124.5 (rotameric peaks), 83.1 + 82.8 (rotameric peaks), 80.94 + 80.91 (rotameric peaks), 41.3, 41.2 (rotameric peaks), 36.28 + 36.26 (rotameric peaks), 35.1 + 35.0 (rotameric peaks), 26.2, 25.0, 5.7 (br.) ppm.

^{11}B NMR (128 MHz, $CDCl_3$): δ_B 35.3 (br. s, 1B) ppm.

IR (film) ν_{max}: 3312, 2976 – 2931, 1638, 1597, 1540, 1506, 1487, 1370, 1313, 1231, 1143, 1092, 1014, 967 cm^{-1}.

HRMS (ESI^+) calcd. for $C_{26}H_{36}BClNO_4$ $[M+H]^+$ 472.24511, found 472.242093.

2-(6-(2,5-Dimethylphenoxy)-3,3-dimethylhexyl)-4,4,5,5-tetramethyl-1,3,2-dioxaborolane (46)

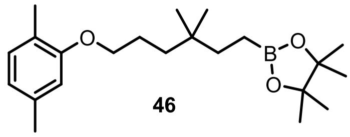

Prepared following General Procedure C with gemfibrozil (50 mg, 0.20 mmol, 1.0 equiv.), Ir[dF(Me)ppy]$_2$(dtbbpy)PF$_6$ (4.1 mg, 0.0040 mmol, 2.0 mol%), Cs_2CO_3 (65 mg, 0.20 mmol, 1.0 equiv.), vinyl boronic acid pinacol ester (51 µl, 0.30 mmol, 1.5 equiv.) and DMA (4.0 mL), which was irradiated with 1 × Kessil lamp for 62 h. Purification by flash column chromatography (4% EtOAc/pentane) gave the title compound (49 mg, 0.14 mmol, 68%) as a colourless oil.

TLC: R_f = 0.55 (4% EtOAc/pentane, $KMnO_4$ stain).

^{1}H NMR (500 MHz, $CDCl_3$): δ_H 7.01 (d, *J* = 7.4 Hz, 1H), 6.66 (dd, *J* = 7.5 Hz, 1.5 Hz, 1H), 6.63 (d, *J* = 1.6 Hz, 1H), 3.91 (t, *J* = 6.6 Hz, 2H), 2.32 (s, 3H), 2.19 (s, 3H), 1.80 – 1.70 (m, 2H), 1.39 – 1.31 (m, 4H), 1.26 (s, 12H), 0.88 (s, 6H), 0.77 – 0.69 (m, 2H) ppm.

^{13}C NMR (126MHz, $CDCl_3$): δ_C 157.3, 136.5, 130.3, 123.7, 120.6, 112.1, 83.0, 68.9, 37.6, 35.6, 33.1, 26.7, 24.9, 24.4, 21.5, 15.9, 5.3 (br.) ppm.

^{11}B NMR (128 MHz, $CDCl_3$): δ_B 33.7 (br. s, 1B) ppm.

IR (film) ν_{max}: 2951 – 2867, 1615, 1585, 1508, 1468, 1370, 1329, 1312, 1264, 1144, 1129, 1039, 967 cm^{-1}.

HRMS (ESI$^+$) calcd. for $C_{22}H_{37}BNaO_3$ $[M+Na]^+$ 383.273198, found 383.274368.

Ethyl (4*R*,5*S*)-2,2-dimethyl-5-(2-(4,4,5,5-tetramethyl-1,3,2-dioxaborolan-2-yl)ethyl)-1,3-dioxolane-4-carboxylate (**47**)

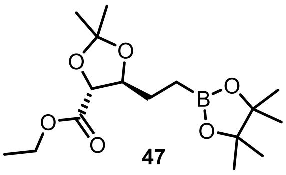

Prepared following General Procedure B with (4*R*,5*R*)-5-(ethoxycarbonyl)-2,2-dimethyl-1,3-dioxolane-4-carboxylic acid (66 mg, 0.30 mmol, 1.0 equiv.), Ir[dF(CF_3)ppy]$_2$(dtbbpy)PF_6 (6.7 mg, 0.0060 mmol, 2.0 mol%), Cs_2CO_3 (98 mg, 0.30 mmol, 1.0 equiv.), vinyl boronic acid pinacol ester (76 µl, 0.45 mmol, 1.5 equiv.) and DMA (6.0 mL), which was irradiated with 2 × Kessil lamps for 62 h. Purification by flash column chromatography (10% EtOAc/pentane) gave the title compound (29 mg, 0.088 mmol, 29%) as a colourless oil. The diastereomeric ratio was determined to be >95:5 by ^{1}H NMR.

TLC: R_f = 0.40 (10% EtOAc/pentane, $KMnO_4$ stain).

Optical rotation: $[\alpha]_D^{22}$ –20 (*c* 1.0, $CHCl_3$).

^{1}H NMR (500 MHz, $CDCl_3$): δ_H 4.28 – 4.18 (m, 2H), 4.13 – 4.07 (m, 2H), 1.94 - 1.86 (m, 1H), 1.82 – 1.74, (m, 1H), 1.45 (s, 3H), 1.43 (s, 3H), 1.29 (t, *J* = 7.1 Hz, 3H), 1.24 (s, 12H), 0.97 – 0.84 (m, 2H) ppm.

^{13}C NMR (126 MHz, $CDCl_3$): δ_C 171.3, 110.9, 83.3, 80.7, 78.9, 61.4, 27.9, 27.3, 25.9, 25.0, 24.9, 14.3, 7.0 (br.) ppm.

^{11}B NMR (128 MHz, $CDCl_3$): δ_B 33.5 (br. s, 1B) ppm.

IR (film) ν_{max}: 2980 – 2936, 1756, 1732, 1370, 1318, 1198, 1144, 1094, 1030, 967 cm^{-1}.

HRMS (ESI$^+$) calcd. for $C_{16}H_{30}BO_6$ $[M+H]^+$ 329.213295, found 329.213479.

The relative configuration of product **47** was determined to be *anti* by oxidation to the corresponding alcohol **S4** and comparison of the spectroscopic data with previous literature reports (Scheme S3).

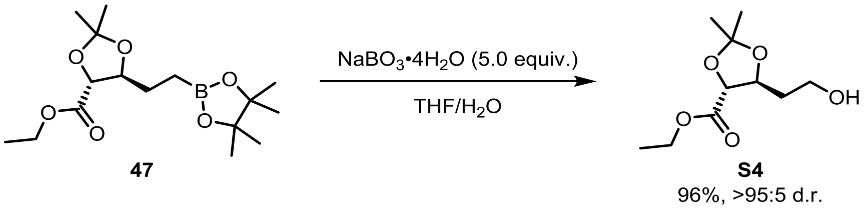

Scheme S3: Oxidation of boronic ester **47** for determination of relative configuration

Ethyl (4*R*,5*S*)-5-(2-hydroxyethyl)-2,2-dimethyl-1,3-dioxolane-4-carboxylate (**S4**)

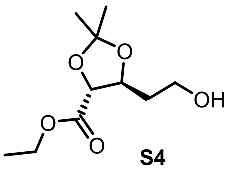

S4

To a stirred solution of ethyl (4*R*,5*S*)-2,2-dimethyl-5-(2-(4,4,5,5-tetramethyl-1,3,2-dioxaborolan-2-yl)eth-yl)-1,3-dioxolane-4-carboxylate (**47**, 25 mg, 0.076 mmol) in THF (0.30 mL) and H_2O (0.30 mL) at room temperature was added $NaBO_3$•$4H_2O$ (58 mg, 0.38 mmol, 5.0 equiv.). After stirring for 4 h, brine (2 mL) was added and the aqueous layer was extracted with Et_2O (2 × 5 mL). The combined organic phases were dried (Mg_2SO_4), filtered and the solvent removed *in vacuo*. The crude product was purified by flash column chromatography (50% EtOAc/pentane) yielding the title compound (16 mg, 0.073 mmol, 96%) as a colourless oil.

TLC: R_f = 0.30 (50% EtOAc/pentane, $KMnO_4$ stain).

^{1}H NMR (500 MHz, $CDCl_3$): δ_H 4.32 – 4.19 (m, 2H), 3.83 (t, *J* = 5.7 Hz, 2H), 2.11 – 2.01, (m, 1H), 1.99 – 1.91 (m, 1H), 1.48 (s, 3H), 1.44 (s, 3H), 1.30 (t, *J* = 7.1 Hz, 3H) ppm.

^{13}C NMR (126 MHz, $CDCl_3$): δ_C 170.9, 111.2, 79.1, 78.01, 61.7, 60.3, 35.9, 27.2, 25.7, 14.3 ppm.

All spectroscopic data are consistent with previous literature reports.[12]

[12] Badalassi, F.; Nguyen, H. K.; Crotti, P.; Reymond, J.-L. *Helv. Chim. Acta* **2002**, *85*, 3090.

8. Deuteration Studies

Deuterium incorporation studies were performed with Boc-Pro-OCs (**51**), the preformed cesium salt of Boc-Pro-OH (**1**), in the presence of deuterium oxide (Scheme S4).

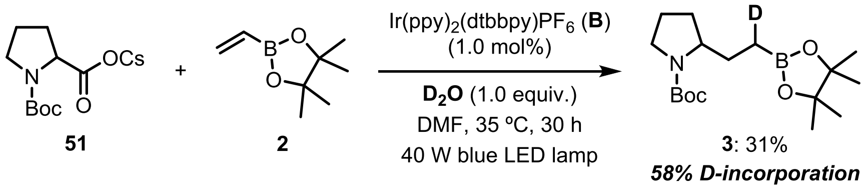

Scheme S4: Deuterium incorporation studies with preformed cesium salt **51**

Boc-Pro-OCs (**51**) was prepared by the slow addition of MeOH/H_2O (2:1, 5.4 mL) to a mixture of Boc-Pro-OH (**1**) (937 mg, 4.35 mmol) and Cs_2CO_3 (709 mg, 2.18 mmol, 0.50 equiv.) [Care: effervescence of CO_2]. The resultant solution was stirred for 15 min at room temperature before removal of the solvents *in vacuo*. The resulting white solid was dried by heating at 50 °C under high-vacuum for 12 h to give a quantitative yield of **51**.

The deuterium incorporation study was carried out as follows: In a N_2-filled glovebox, Boc-Pro-OCs (**51**) (43 mg, 0.12 mmol) and Ir(ppy)$_2$(dtbbpy)PF_6 (1.1 mg, 0.0012 mmol, 1.0 mol%) were added to a dry 7 mL vial. The vial was sealed with a septum and removed from the glovebox before the sequential addition of anhydrous DMF (2.5 mL), vinyl boronic acid pinacol ester (32 µL, 0.19 mmol, 1.5 equiv.) and D_2O (2.2 µL, 0.12 mmol, 1.0 equiv.). The reaction mixture degassed by sparging with nitrogen for 10 minutes. The nitrogen inlet was removed and the vial further sealed with parafilm. The reaction mixture was stirred at 800 rpm and irradiated with 1 × Kessil lamps for 20 h. D_2O (1.0 mL) was added and the mixture stirred for 5 min. The reaction mixture was diluted with water (10 mL) and extracted into ethyl acetate (3 × 10 mL). The organic phase was washed with water (10 mL), brine (10 mL), dried (Na_2SO_4) and filtered before removal of the solvent *in vacuo*. Purification by flash column chromatography (10% EtOAc/pentane) gave the product **3** (12 mg, 0.037 mmol, 31%) as a colourless oil. The product was formed with 58% D-incorporation α to the boronic ester group, as determined by ^{1}H NMR.

HRMS (MALDI) calcd. for $C_{17}H_{31}DBNO_4Na$ $[M+Na]^+$ 349.2383, found 349.2378.

While the incorporation of deuterium into the product of the reaction shown in Scheme S4 confirms the formation of an intermediate α-boryl anion (**50**, Scheme 2), the lack of complete deuteration raises the possibility that alternative mechanisms may also be operating. The 42%

protonated product could either arise from H_2O contaminants in the reaction mixture (e.g., from the DMF, D_2O, or the hydroscopic cesium salt **51**) or via a hydrogen-atom-transfer process between the intermediate α-boryl radical (**49**, Scheme 2) and the solvent DMF. To investigate this further, the reaction between Boc-Pro-OH (**1**) and vinyl boronic ester **2** was performed in DMF-d_7 (Scheme S5). The product **3** was formed in 63% yield without any observed deuterium incorporation, suggesting that the protonated product formed in Scheme S4 arises from H_2O contamination.

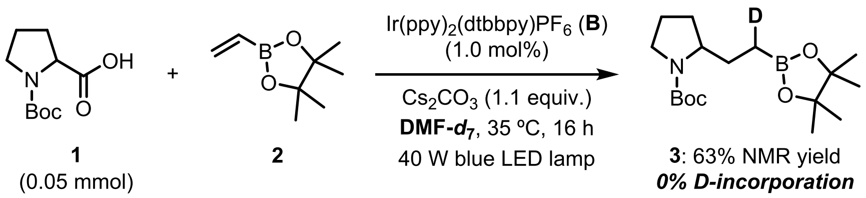

Scheme S5: Deuterium incorporation studies with DMF-d_7

9. Cyclic Voltammetry Studies

Cyclic voltammetry was carried out using iodomethylboronic acid pinacol ester (**S5**), which underwent two single-electron reductions to form anion **S7** *via* radical **S6** (Scheme S6).

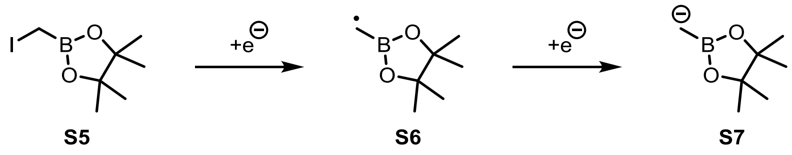

Scheme S6: Reduction of iodide **S5** to anion **S7** *via* radical **S6**

Samples were prepared with 0.025 mmol of **S5** in 4 mL of 5 mM tetra-*n*-butylammonium hexafluorophosphate in dry, degassed MeCN. A glassy carbon working electrode, platinum wire counter electrode, silver wire reference electrode were used. A scan rate over a range between 50 – 200 mV s^{-1} was used and an average reduction potential was taken. The obtained value was referenced to Fc/Fc^+ and converted to SCE by adding 0.38 V.

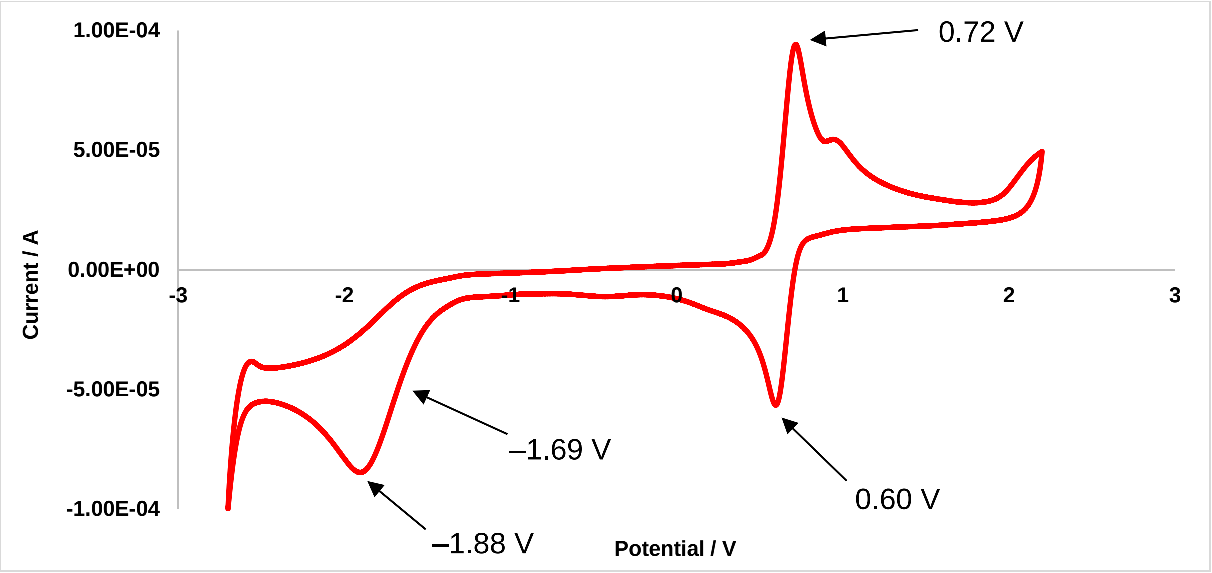

Figure S1. Cyclic voltammogram of **S5** at a scan rate of 100 mV s^{-1}

Taking values from scan rates of 50, 100 and 200 mV s^{-1}, an average value of $E_{p/2}$ = –2.38 V vs Fc/Fc^+ in MeCN was obtained, which corresponds to a value of –2.0 V vs SCE.

10. DFT Calculation of Reduction Potential

The reduction potential, $E_{1/2}^{red}$, of α-boryl radicals was estimated computationally by using the method described by Liu and Guo.[13] Specifically, the value was obtained by computing the quantities associated with the thermodynamic cycle comprising gas-phase ionisation of the α-boryl carbanion of ethyl pinacol boronic ester (EtBpin) and the solvation (MeCN) of both the α-boryl radical and the α-boryl anion (Scheme S7). All DFT calculations were carried out using Gaussian 09.[14]

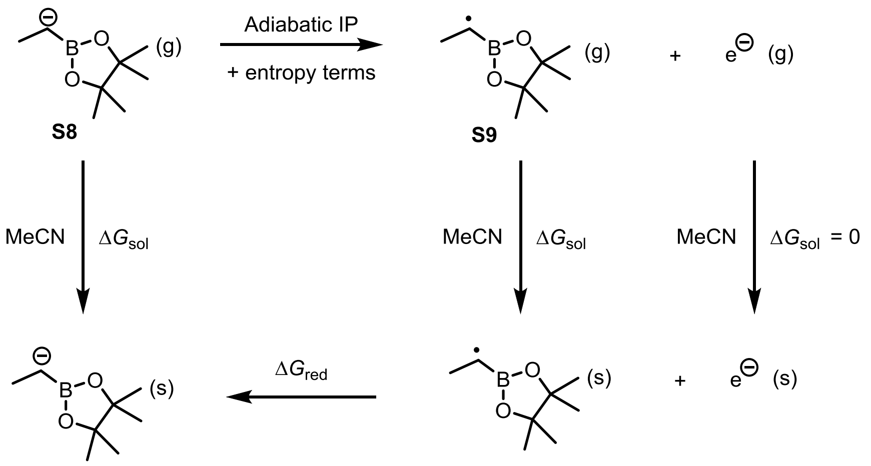

Scheme S7: Thermodynamic cycle for the calculation of $E_{1/2}^{red}$

Using the B3LYP[15] functional and the 6-31+G(d) basis set, gas-phase potential energies (*E*) of the α-boryl carbanion (**S8**) and the α-boryl radical (**S9**) were computed. A frequency calculation at this level of theory allowed zero-point vibrational energies and thermal corrections to be determined. Single-point energy calculations were carried out on the optimised geometries at a higher level of theory, 6-311++G(2df,2p). The solvation free energies in acetonitrile of both the α-boryl carbanion (**S8**) and the α-boryl radical (**S9**) were computed at 6-311++G(2df,2p) using

[13] Fu, Y.; Liu, L.; Yu, H.-Z.; Wang, Y.-M.; Guo, Q.-X. *J. Am. Chem. Soc*. **2005**, *127*, 7227.
[14] Gaussian 09, Revision D.01, M. J. Frisch, G. W. Trucks, H. B. Schlegel, G. E. Scuseria, M. A. Robb, J. R. Cheeseman, G. Scalmani, V. Barone, B. Mennucci, G. A. Petersson, H. Nakatsuji, M. Caricato, X. Li, H. P. Hratchian, A. F. Izmaylov, J. Bloino, G. Zheng, J. L. Sonnenberg, M. Hada, M. Ehara, K. Toyota, R. Fukuda, J. Hasegawa, M. Ishida, T. Nakajima, Y. Honda, O. Kitao, H. Nakai, T. Vreven, J. A. Montgomery, Jr., J. E. Peralta, F. Ogliaro, M. Bearpark, J. J. Heyd, E. Brothers, K. N. Kudin, V. N. Staroverov, T. Keith, R. Kobayashi, J. Normand, K. Raghavachari, A. Rendell, J. C. Burant, S. S. Iyengar, J. Tomasi, M. Cossi, N. Rega, J. M. Millam, M. Klene, J. E. Knox, J. B. Cross, V. Bakken, C. Adamo, J. Jaramillo, R. Gomperts, R. E. Stratmann, O. Yazyev, A. J. Austin, R. Cammi, C. Pomelli, J. W. Ochterski, R. L. Martin, K. Morokuma, V. G. Zakrzewski, G. A. Voth, P. Salvador, J. J. Dannenberg, S. Dapprich, A. D. Daniels, O. Farkas, J. B. Foresman, J. V. Ortiz, J. Cioslowski, and D. J. Fox, Gaussian, Inc., Wallingford CT, 2013.
[15] (a) Becke, A. D. *J. Chem. Phys*. **1993**, *98*, 5648. (b) Lee, C.; Yang, W.; Parr, R. G. *Phys. Rev. B* **1988**, *37*, 785. (c) Colle, R.; Salvetti, O. *Theor. Chim. Acta* **1975**, *37*, 329.

the continuum model (PCM) developed by Tomasi and co-workers;[16] the scaling parameter for acetonitrile was set at ALPHA=1.2, as suggested by Liu and Guo.[13]

Structure	Ethyl Bpin anion	Ethyl Bpin radical
E [Hartree] 6-31+G(p)	–489.9083265	–489.8790349
Thermal Correction to Enthalpy [Hartree] 6-31+G(p)	0.243842	0.245503
Thermal Correction to Free Energy [Hartree] 6-31+G(p)	0.192663	0.194870
E [Hartree] 6-311++G(2df,2p)	–490.0574316	–490.0260448
H [kcal/mol]	–307362.4358	–307249.4480
G [kcal/mol]	–307300.9863	–307281.2206
E [Hartree] PCM MeCN 6-311++G(2df,2p)	–490.1371310	–490.0295160
Δ*G* solv [kcal/mol]	–50.012	–2.178

IP (ionisation potential) = H (radical) – H (anion) = –307249.4480 + 307362.4358
= 112.9878 kcal/mol
= 4.90 eV

IP (corrected) = 4.90 eV + 0.28 eV = 5.18 eV

TΔS (ionisation) = H (radical) – H (anion) – G (radical) + G (anion)
= –307249.4480 + 307362.4358 + 307281.2206 – 307300.9863
= 93.22 kcal/mol

TΔS correction factor for the electron spin degeneracy = 0.82 kcal/mol

TΔS (ionisation, corrected) = 93.22 + 0.82 = 94.04 kcal/mol

From the thermodynamic cycle above, the $E_{1/2}^{red}$ (SCE/MeCN) can be calculated as follows:

$E_{1/2}^{red}$ (SCE/MeCN) = IP + (1/23.06)[–TΔS (ionisation) + ΔG (solv, radical) – ΔG (solv, anion)] – 4.43

[16] Tomasi, J.; Mennucci, B.; Cammi, R. *Chem. Rev*. **2005**, 105, 2999.

(the last term, 4.43, is the absolute reduction potential of the saturated calomel electrode in MeCN, as suggested to be used by Isse and Gennaro)[17]

$E_{1/2}^{red}$ (SCE/MeCN) = 5.18 + (1/23.06)(–94.04 –2.18 + 50.01) – 4.43

$E_{1/2}^{red}$ (SCE/MeCN) = –1.25 V

11. Complete List of Authors for Reference 8

8a) A. Maynard, R. M. Crosby, B. Ellis, R. Hamatake, Z. Hong, B. A. Johns, K. M. Kahler, C. Koble, A. Leivers, M. R. Leivers, A. Mathis, A. J. Peat, J. J. Pouliot, C. D. Roberts, V. Samano, R. M. Schmidt, G. K. Smith, A. Spaltenstein, E. L. Stewart, P. Thommes, E. M. Turner, C. Voitenleitner, J. T. Walker, G. Waitt, J. Weatherhead, K. Weaver, S. Williams, L. Wright, Z. Z. Xiong, D. Haigh and J. B. Shotwell, *J. Med. Chem.*, **2014**, *57*, 1902–1913.

8b) M. J. Luderer, B. Muz, P. de la Puente, S. Chavalmane, V. Kapoor, R. Marcelo, P. Biswas, D. Thotala, B. Rogers and A. K. Azab, *Pharm. Res.*, **2016**, *33*, 2530–2539.

8d) C. P. Decicco, D. J. Nelson, Y. Luo, L. Shen, K. Y. Horiuchi, K. M. Amsler, L. A. Foster, S. M. Spitz, J. J. Merrill, C. F. Sizemore, K. C. Rogers, R. A. Copeland and M. R. Harpel, *Bioorg. Med. Chem. Lett.*, **2001**, *11*, 2561–2564.

[17] Isse, A. A.; Gennaro, A. *J. Phys. Chem. B*. **2010**, *114*, 7894.

12. ^{1}H and ^{13}C NMR Spectra

^{1}H NMR (400 MHz, CDCl$_3$)

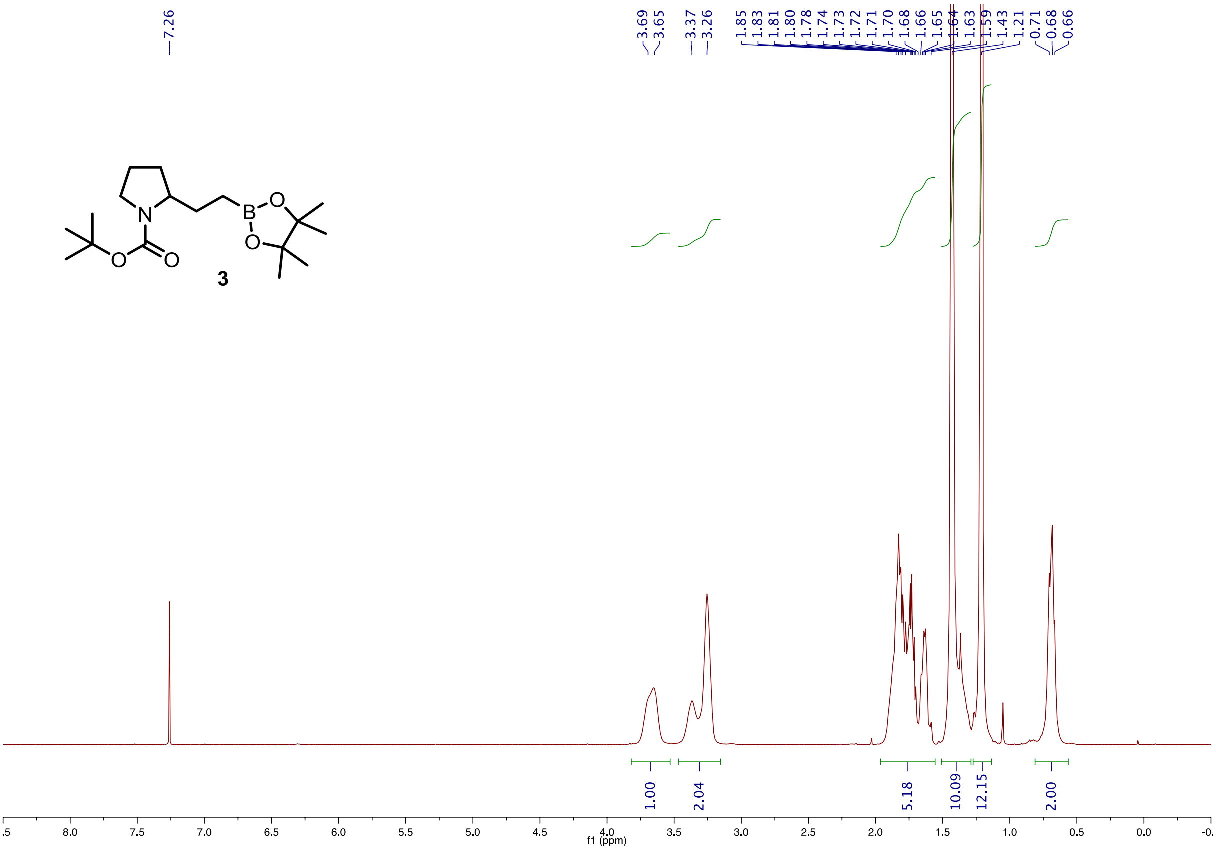

^{13}C NMR (101 MHz, CDCl$_3$)

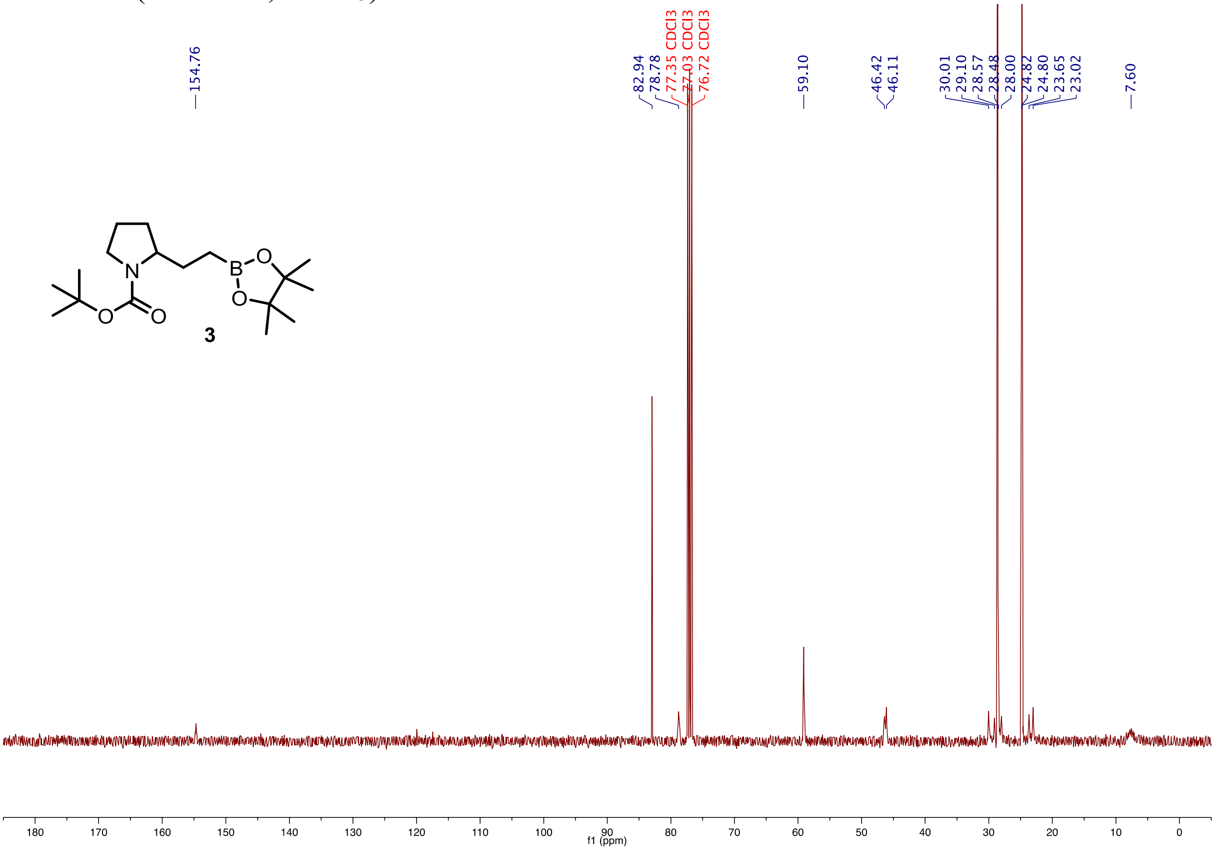

^{1}H NMR (400 MHz, $CDCl_3$)

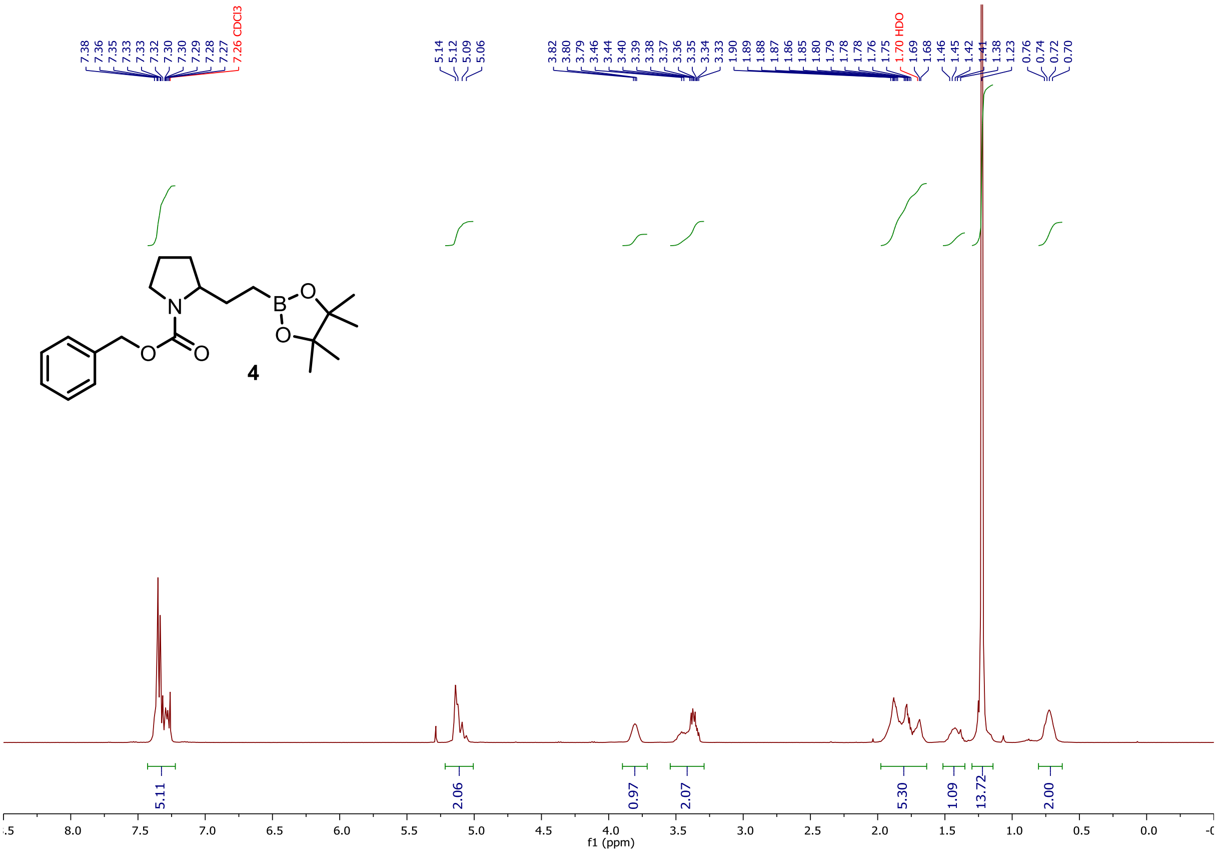

^{13}C NMR (101 MHz, $CDCl_3$)

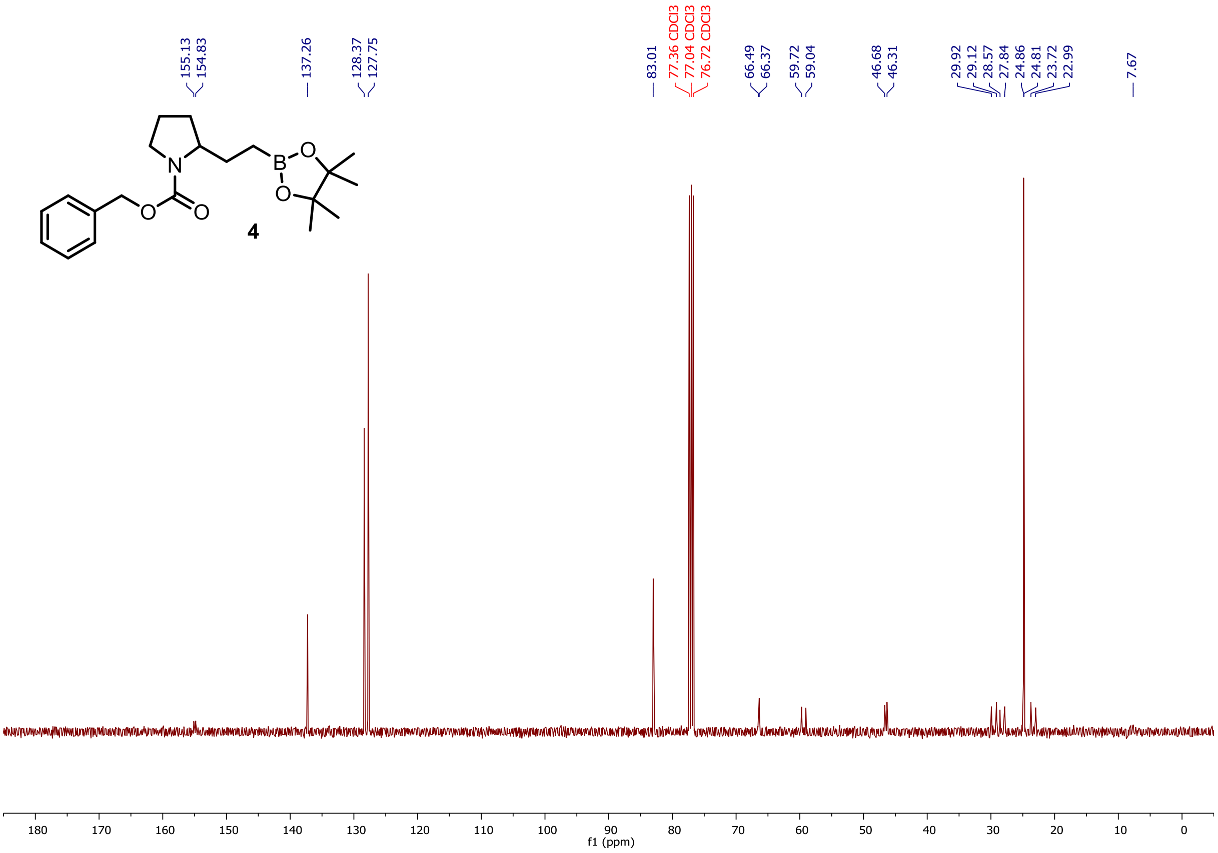

^{1}H NMR (400 MHz, $CDCl_3$)

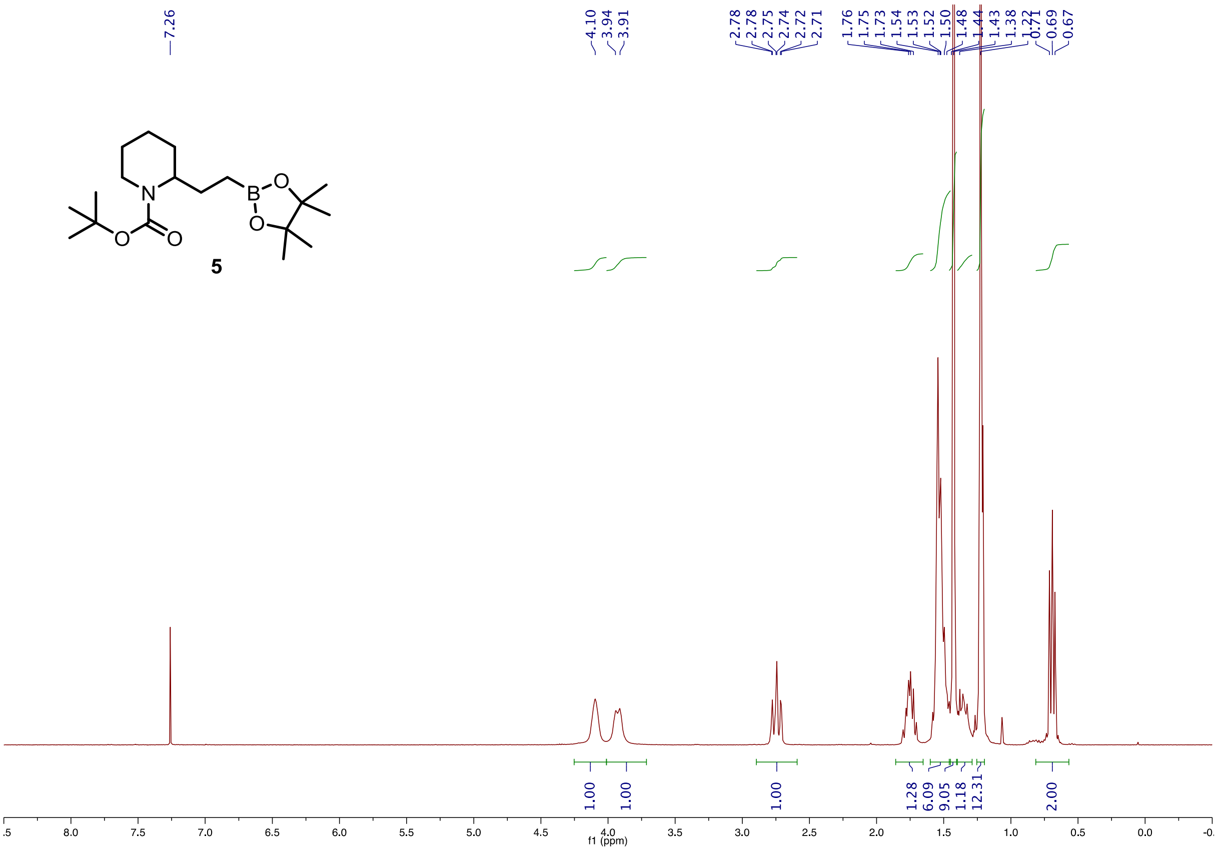

^{13}C NMR (101 MHz, $CDCl_3$)

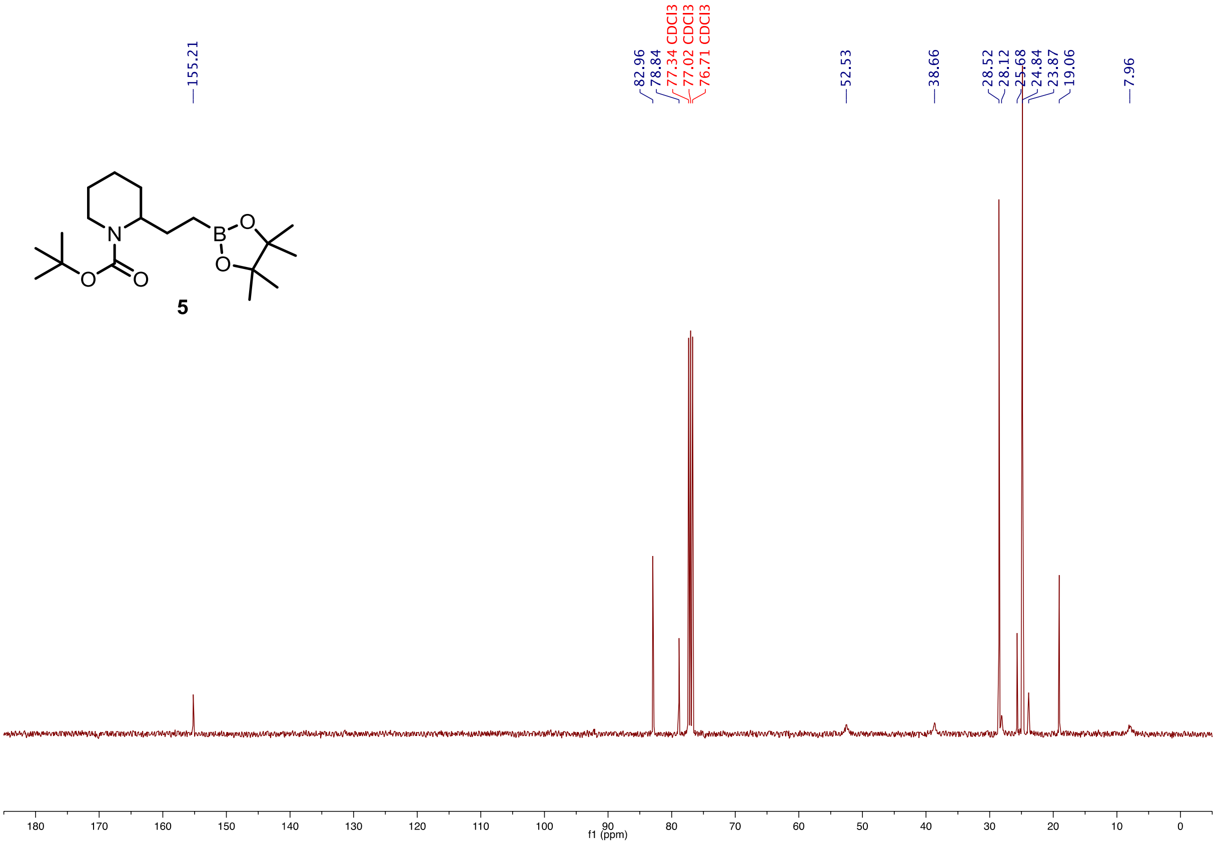

^{1}H NMR (400 MHz, $CDCl_3$)

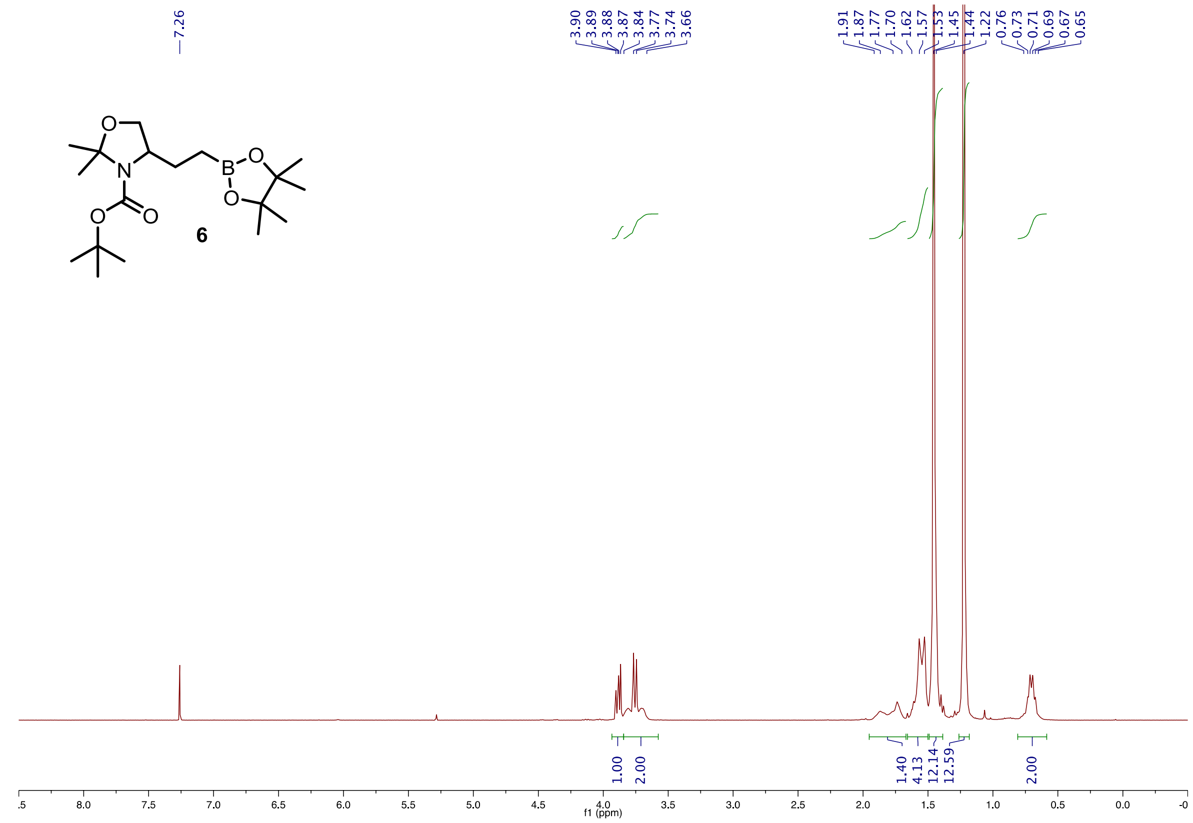

^{13}C NMR (101 MHz, $CDCl_3$)

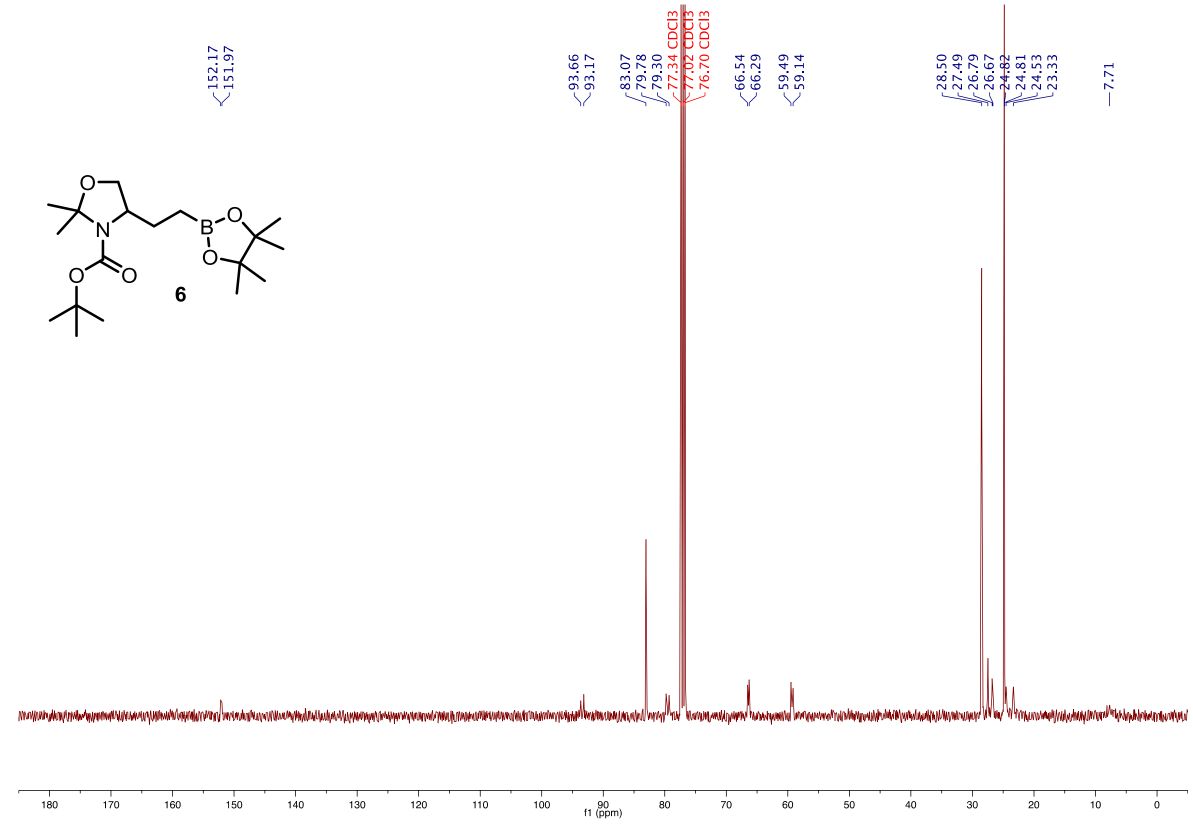

^{1}H NMR (500 MHz, DMSO-d_6, 100 °C)

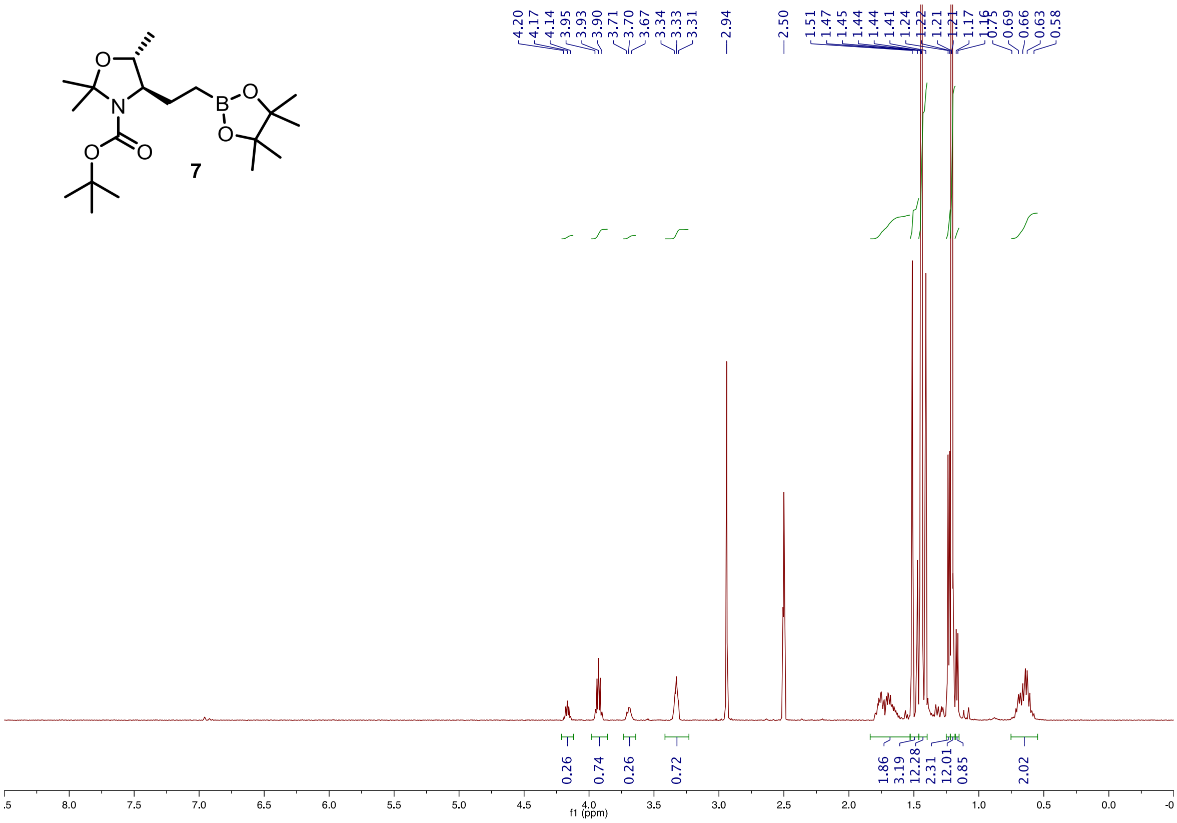

^{13}C NMR (126 MHz, DMSO-d_6, 100 °C)

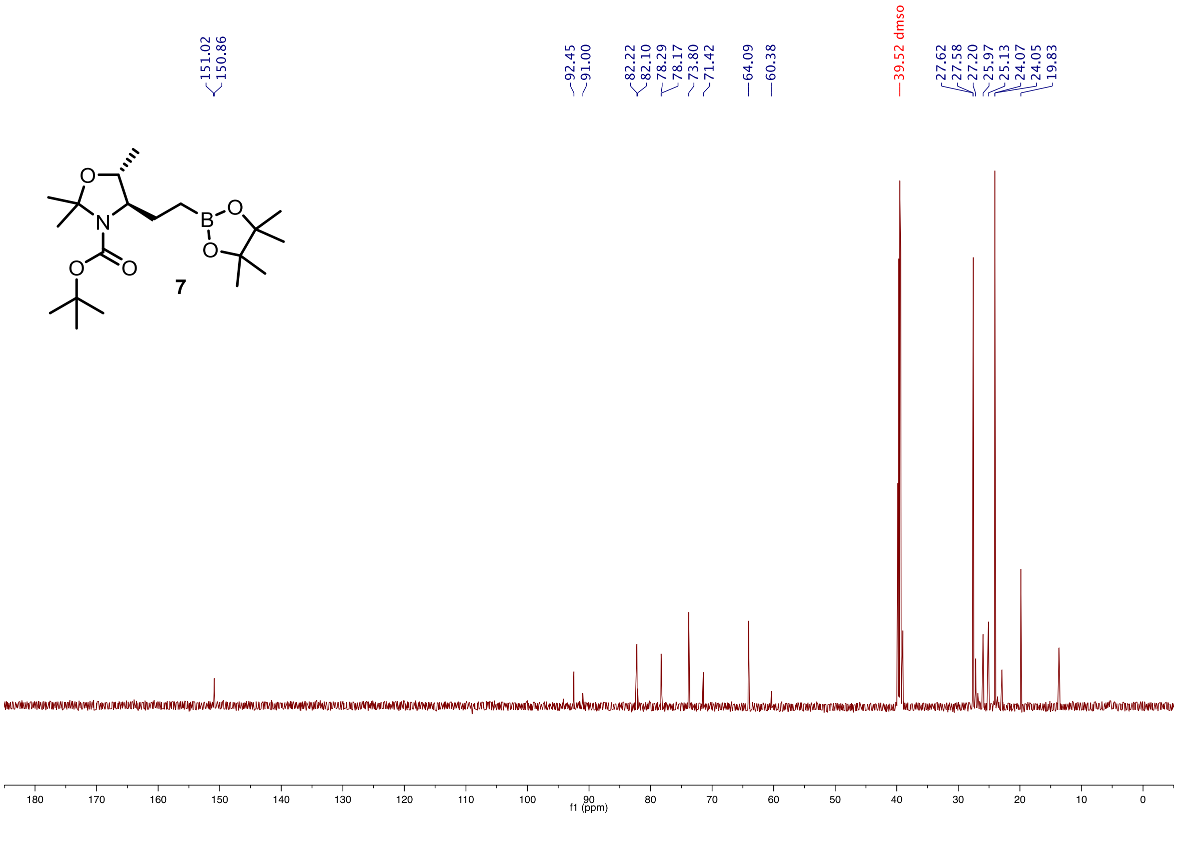

^{1}H NMR (400 MHz, $CDCl_3$)

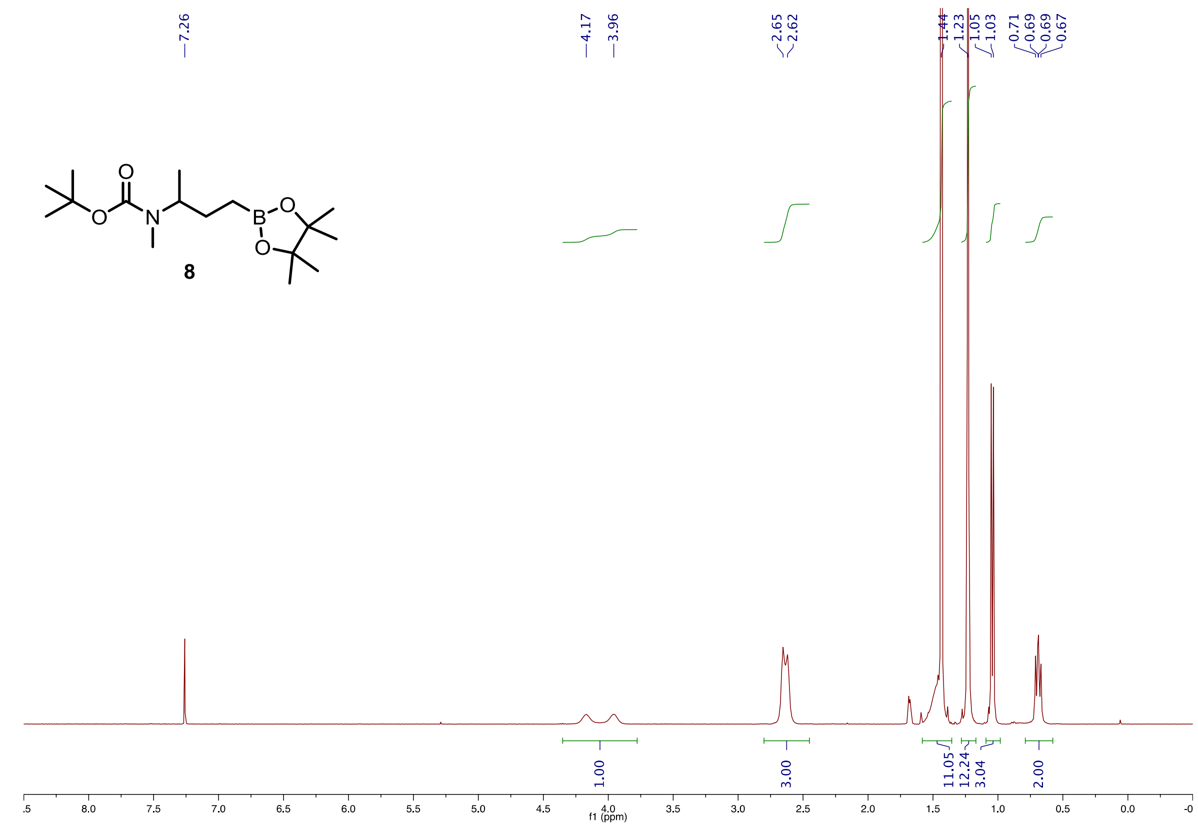

^{13}C NMR (101 MHz, $CDCl_3$)

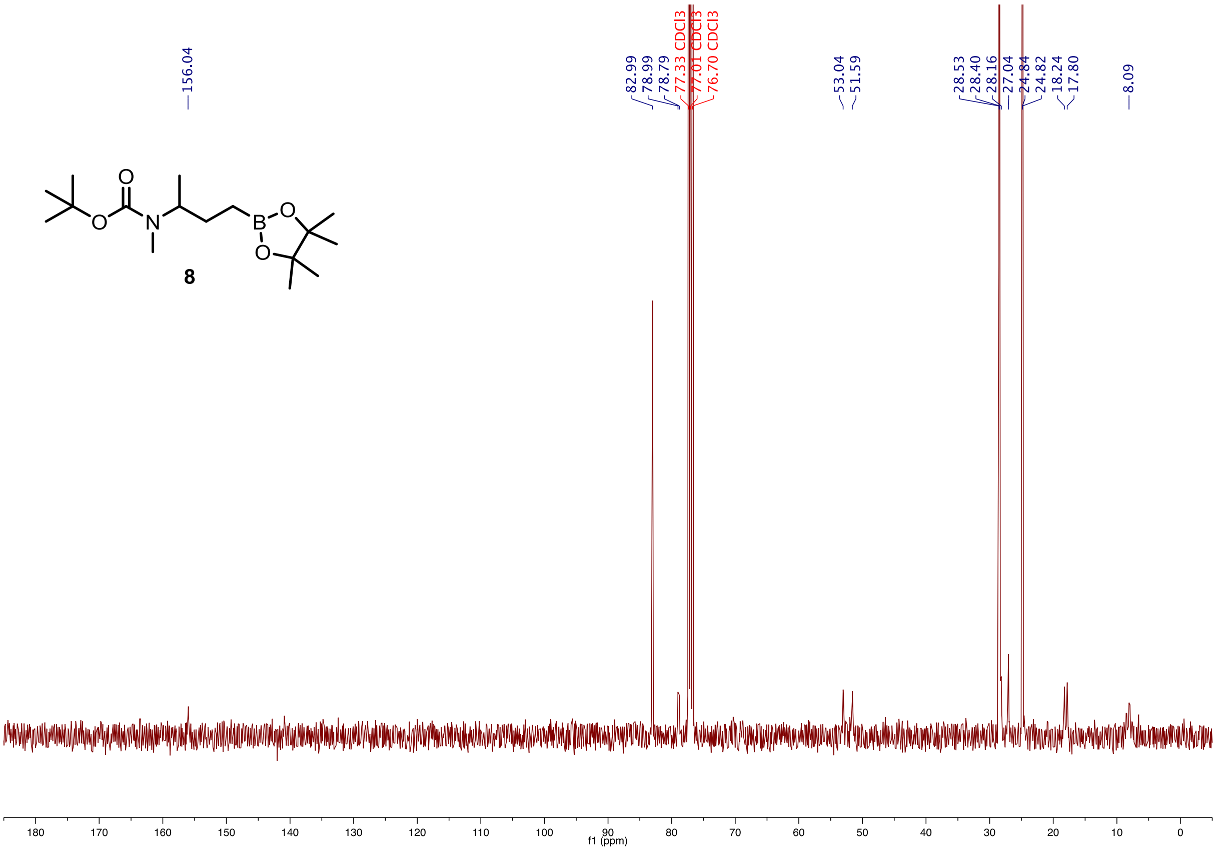

^{1}H NMR (400 MHz, $CDCl_3$)

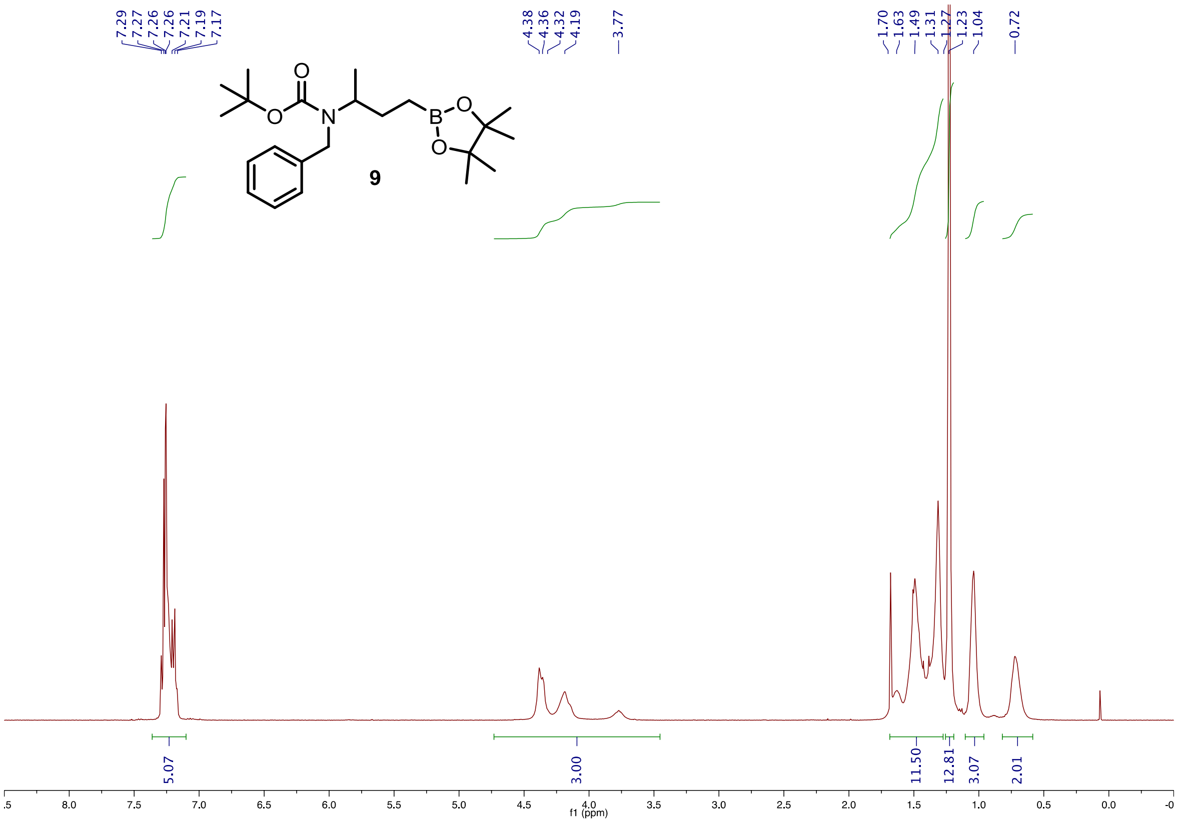

^{13}C NMR (126 MHz, $CDCl_3$)

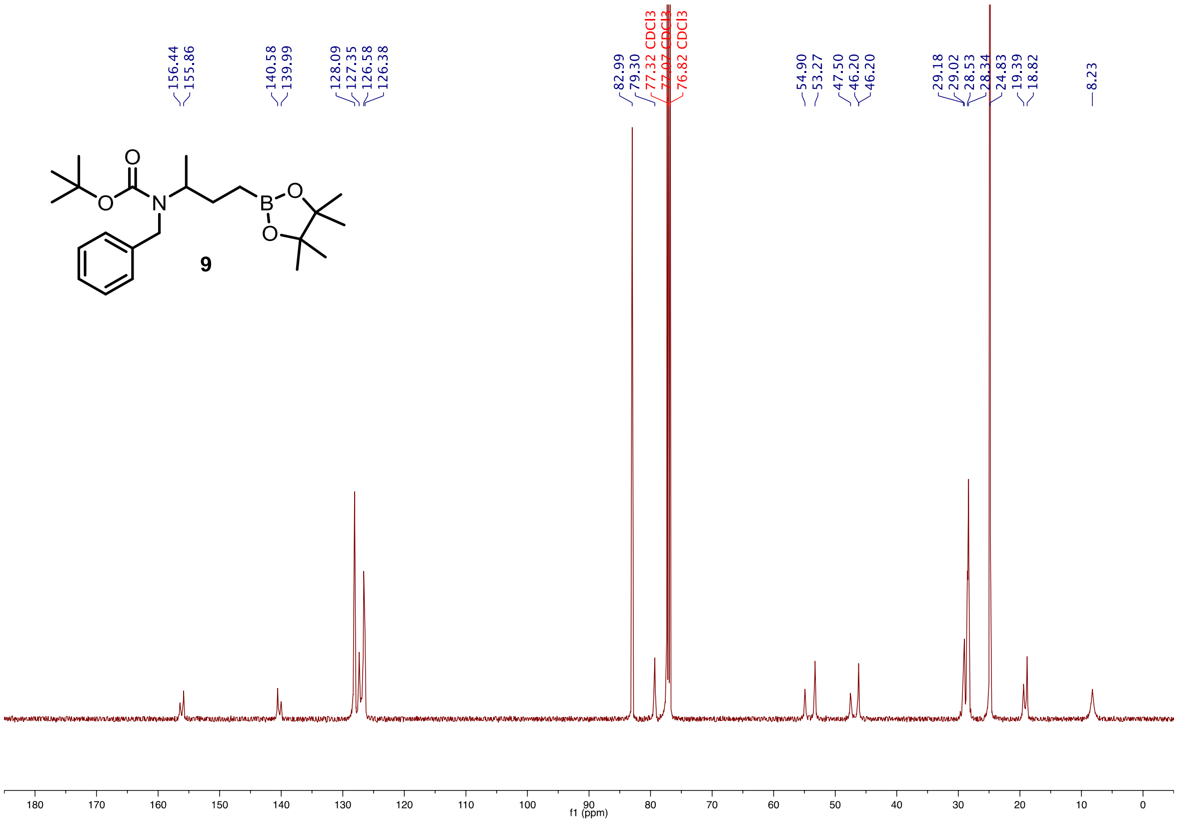

^{1}H NMR (400 MHz, $CDCl_3$)

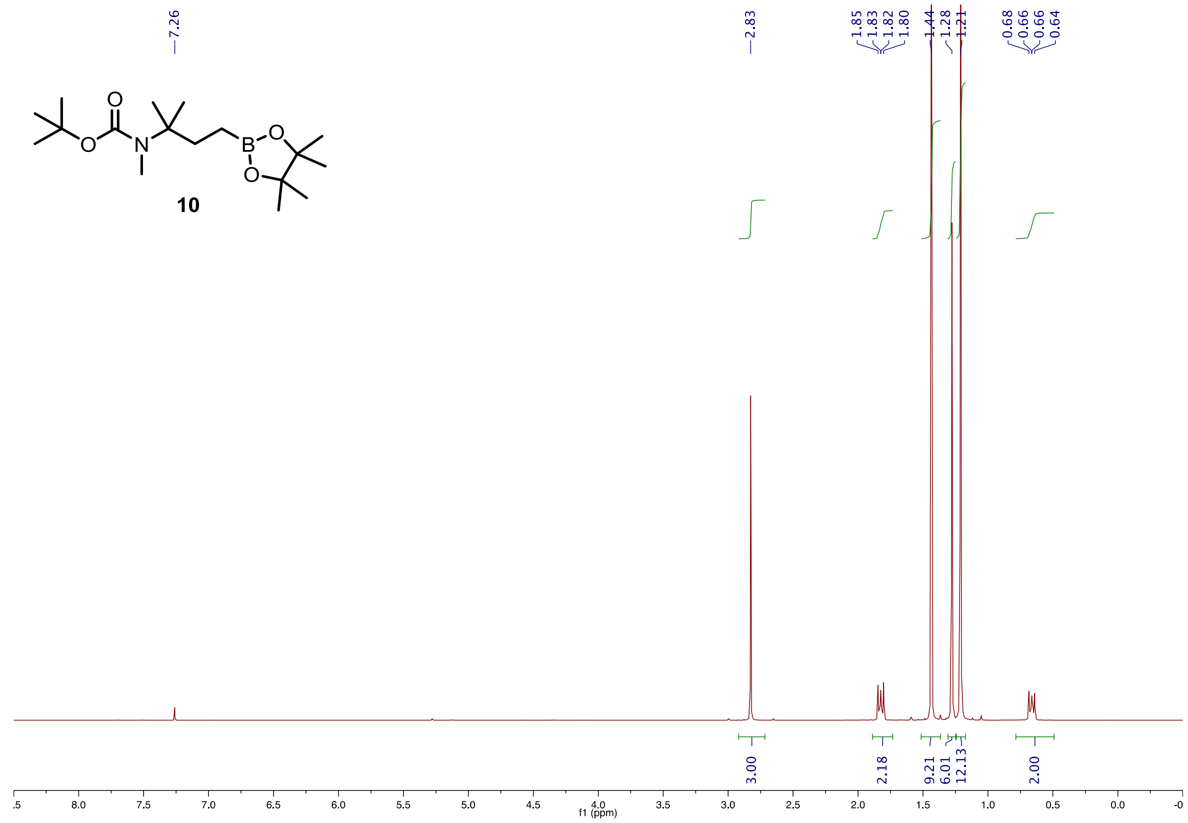

^{13}C NMR (101 MHz, $CDCl_3$)

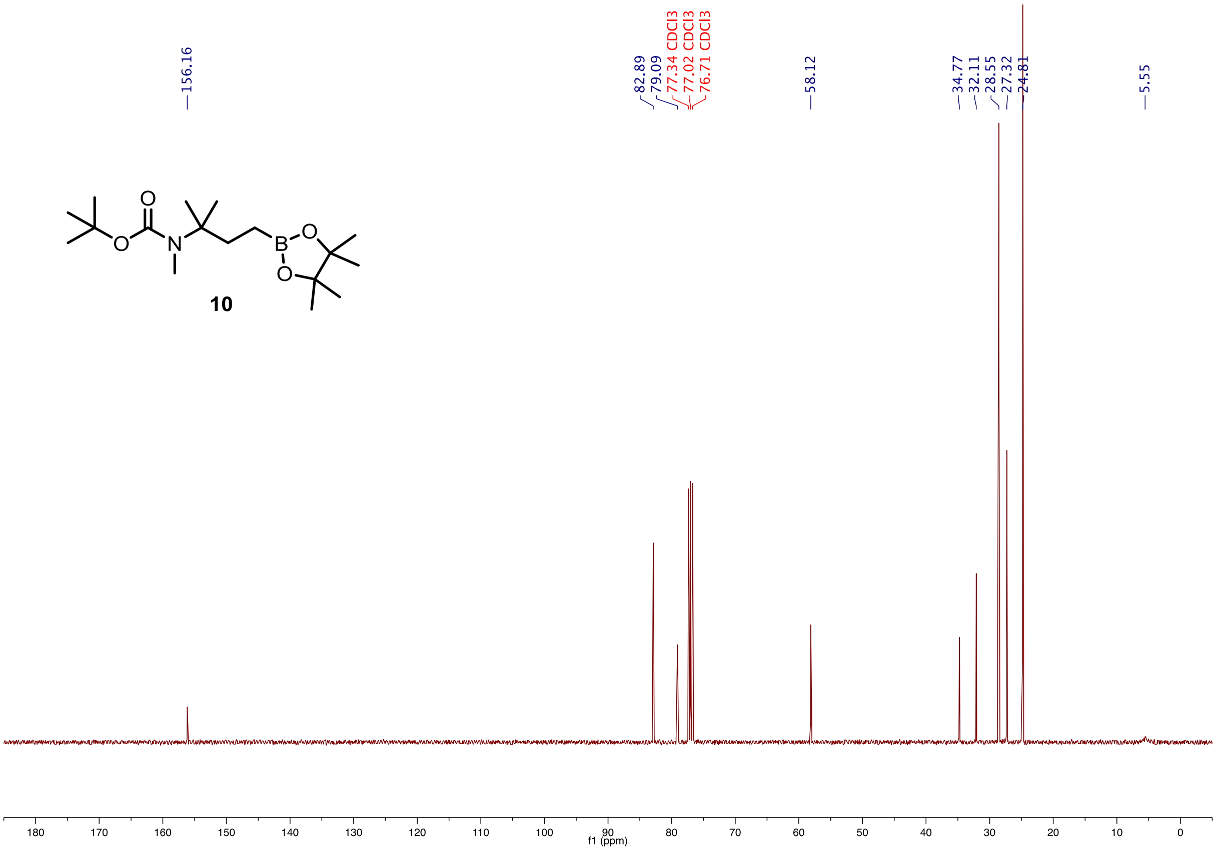

^{1}H NMR (400 MHz, $CDCl_3$)

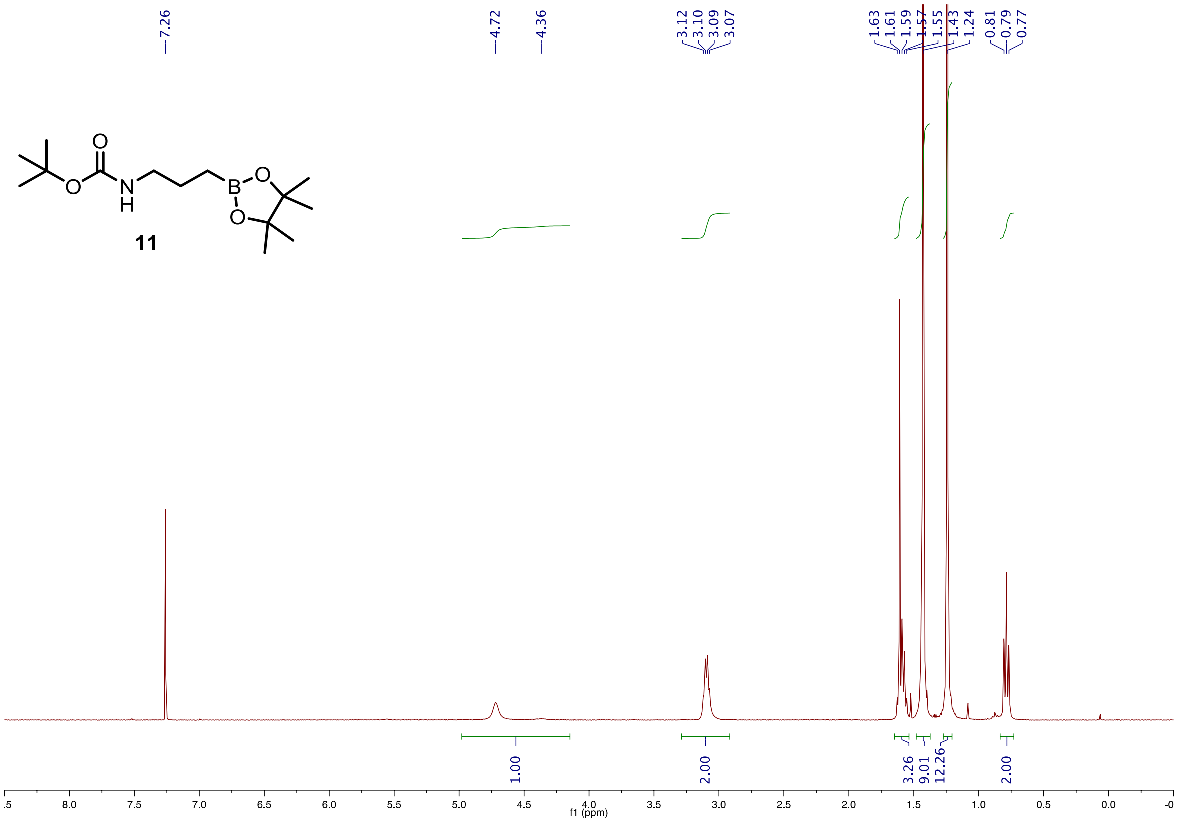

^{13}C NMR (101 MHz, $CDCl_3$)

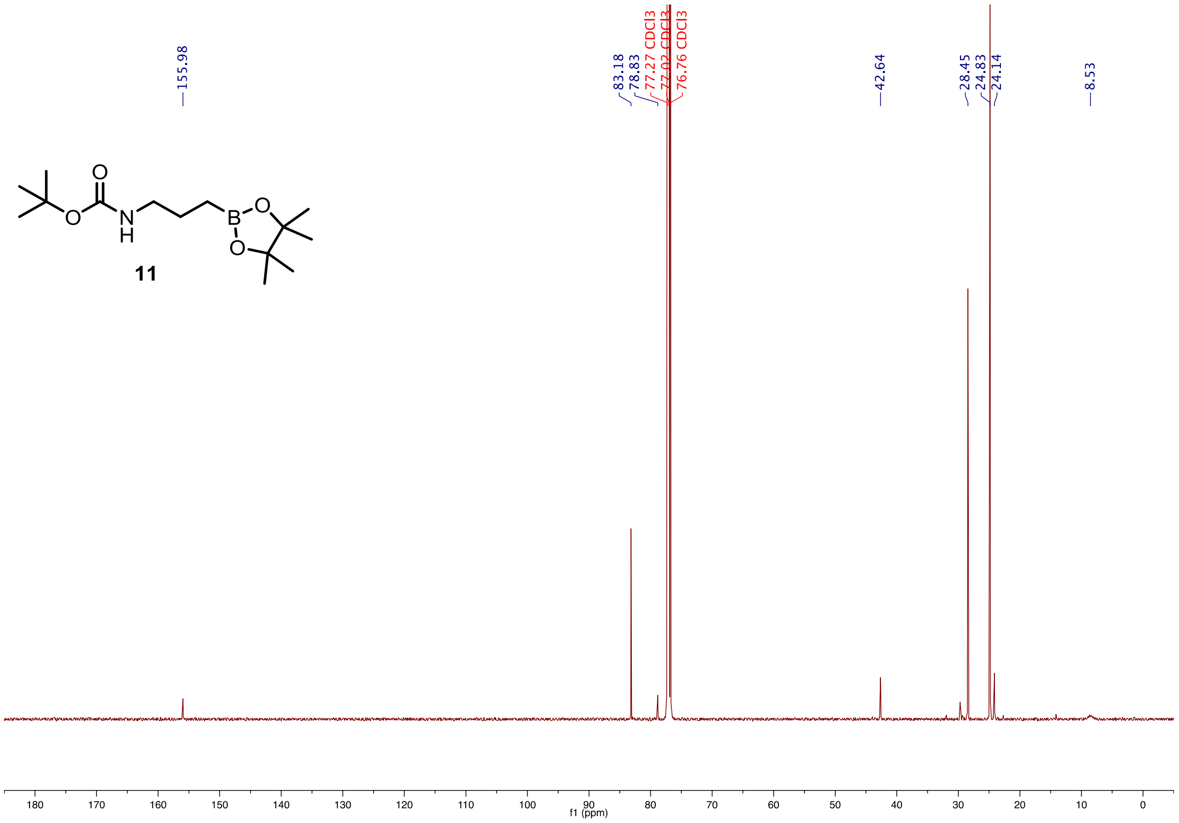

^{1}H NMR (400 MHz, $CDCl_3$)

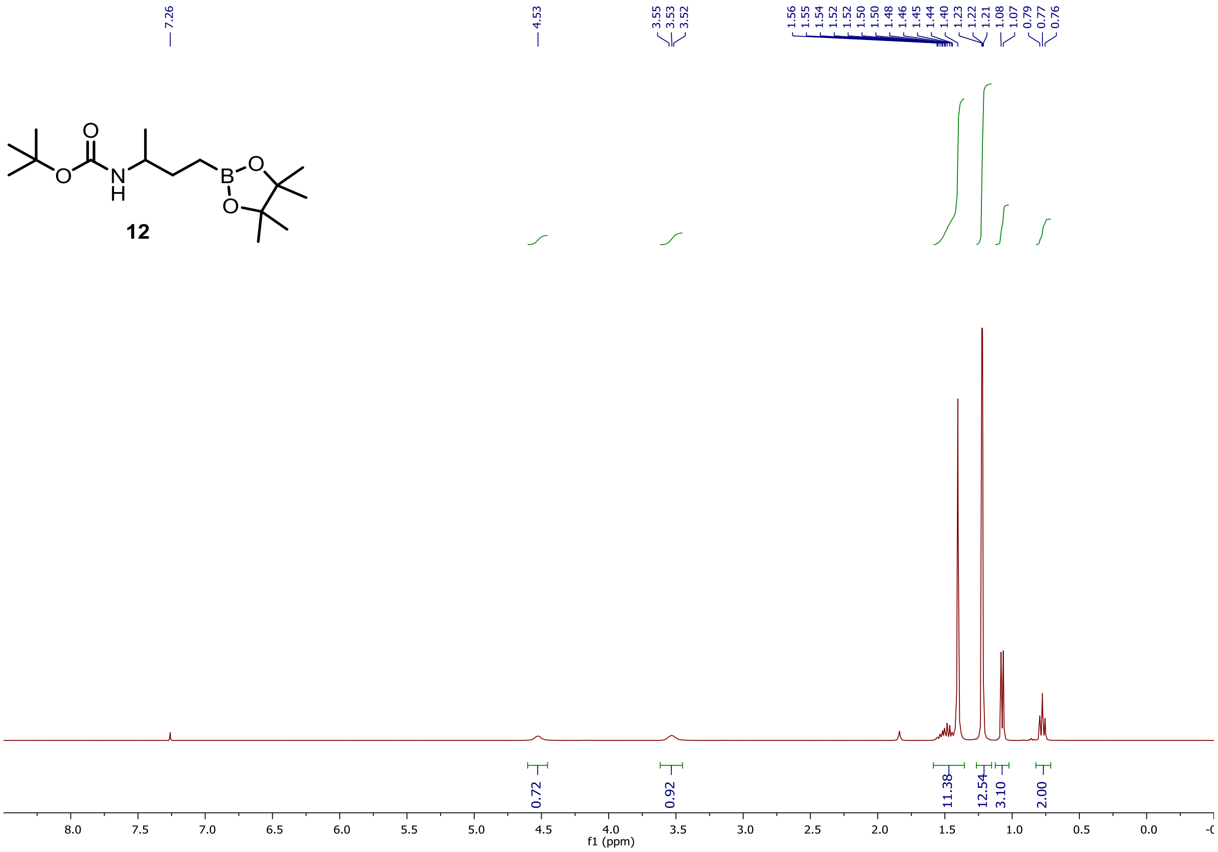

^{13}C NMR (101 MHz, $CDCl_3$)

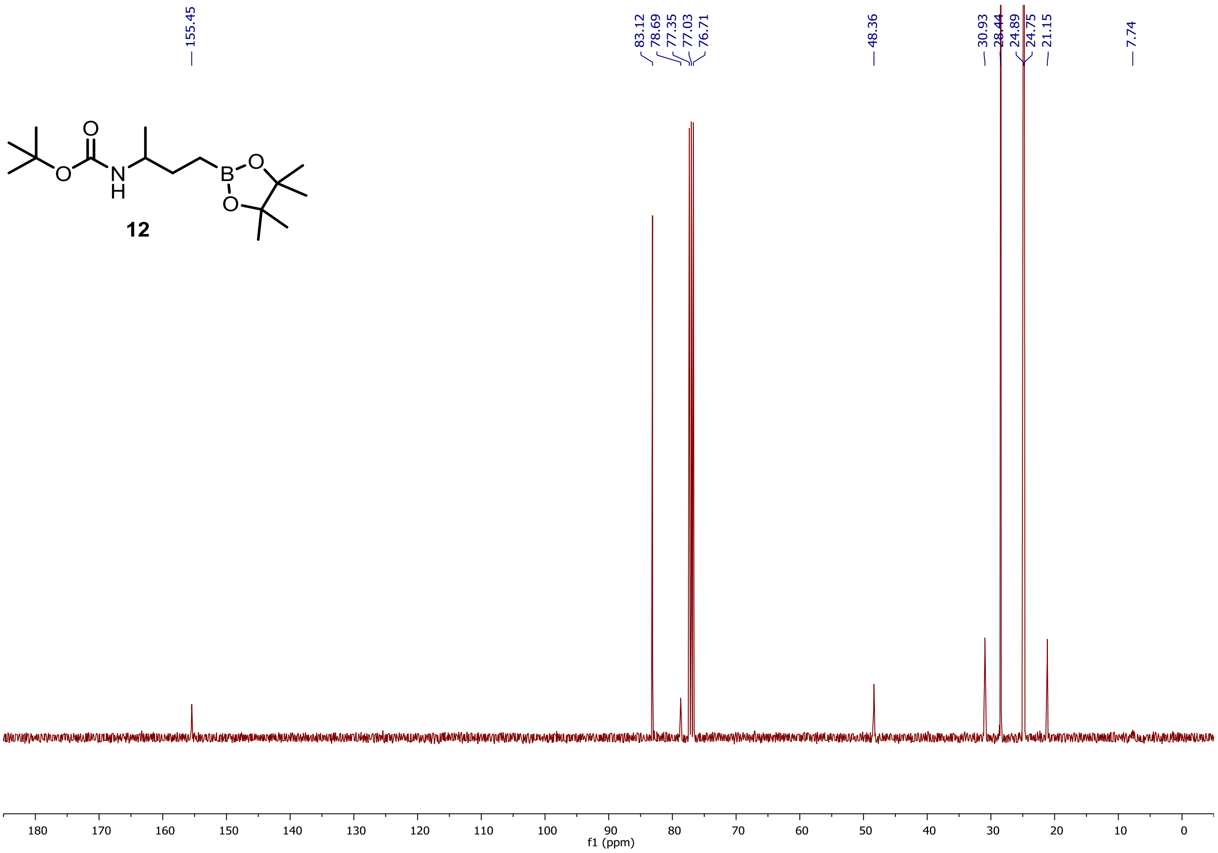

^{1}H NMR (400 MHz, $CDCl_3$)

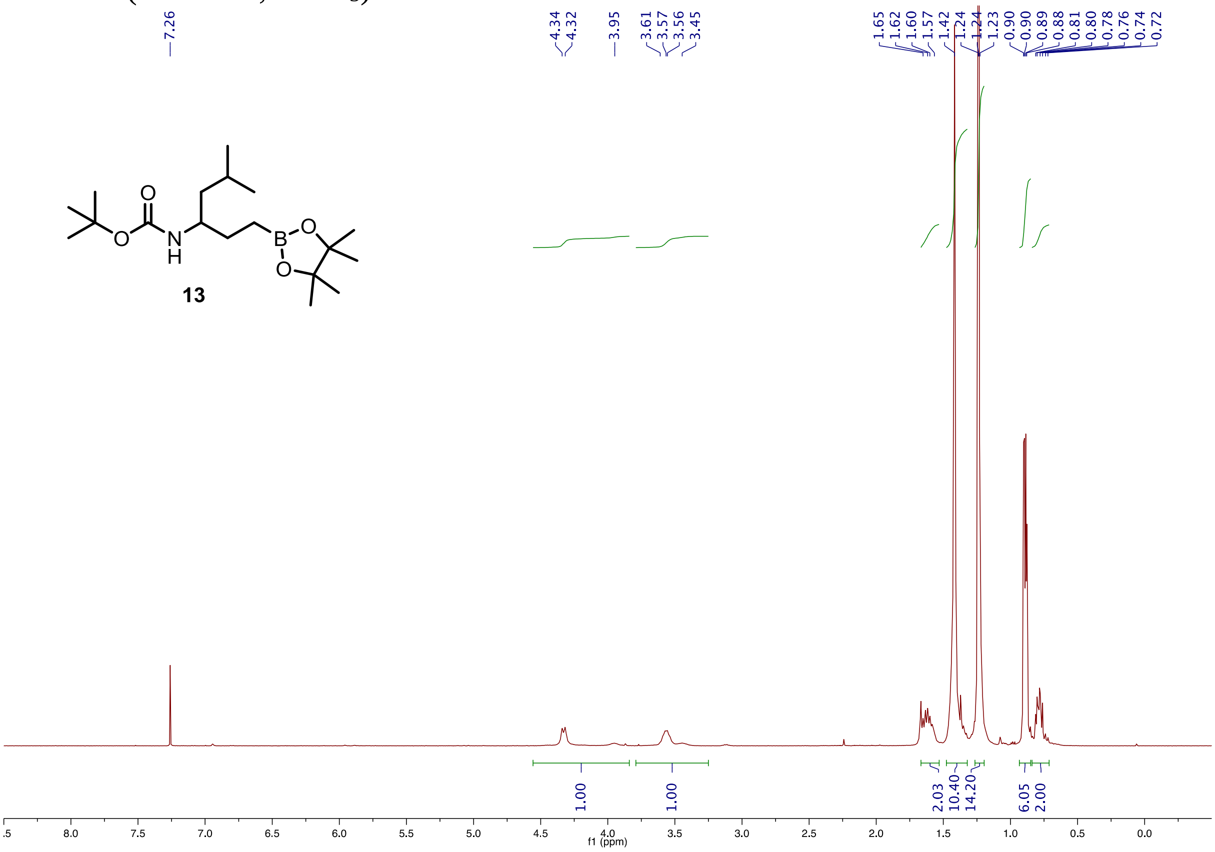

^{13}C NMR (101 MHz, $CDCl_3$)

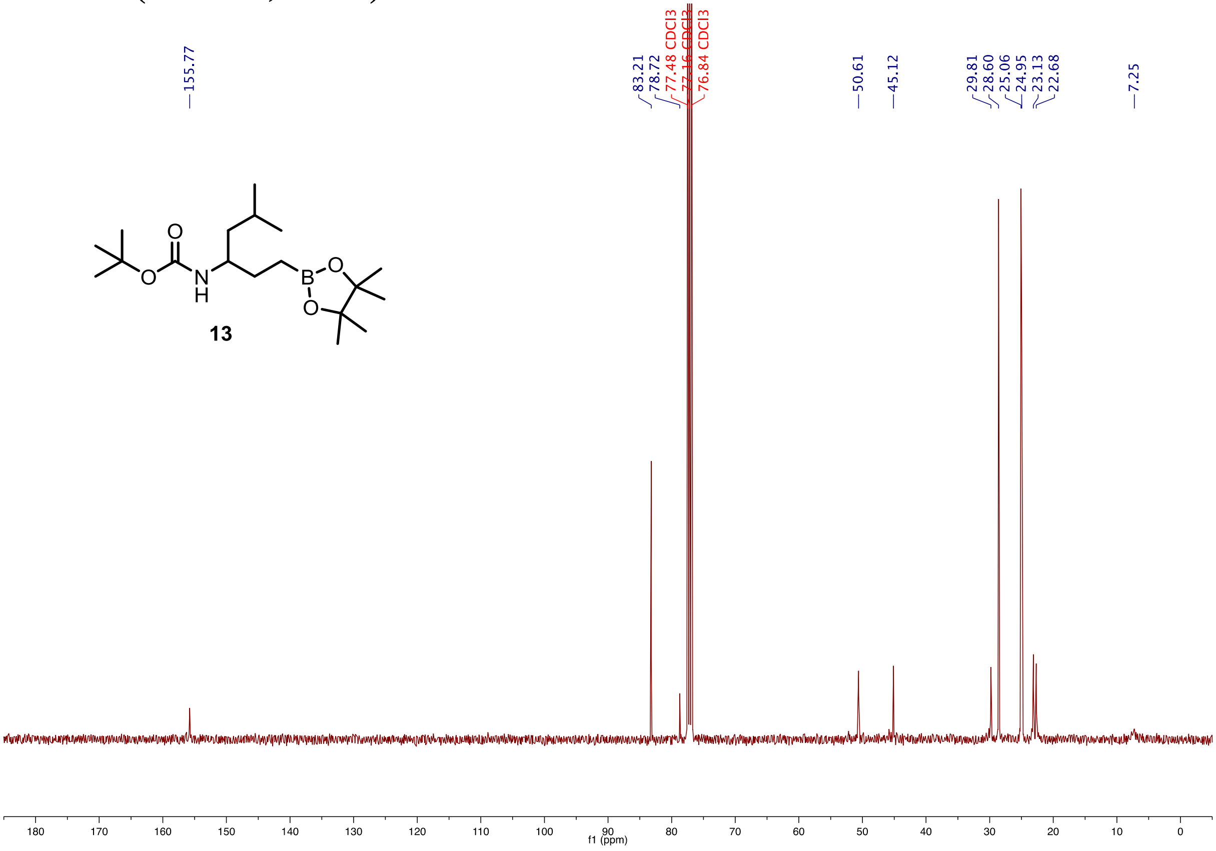

^{1}H NMR (400 MHz, $CDCl_3$)

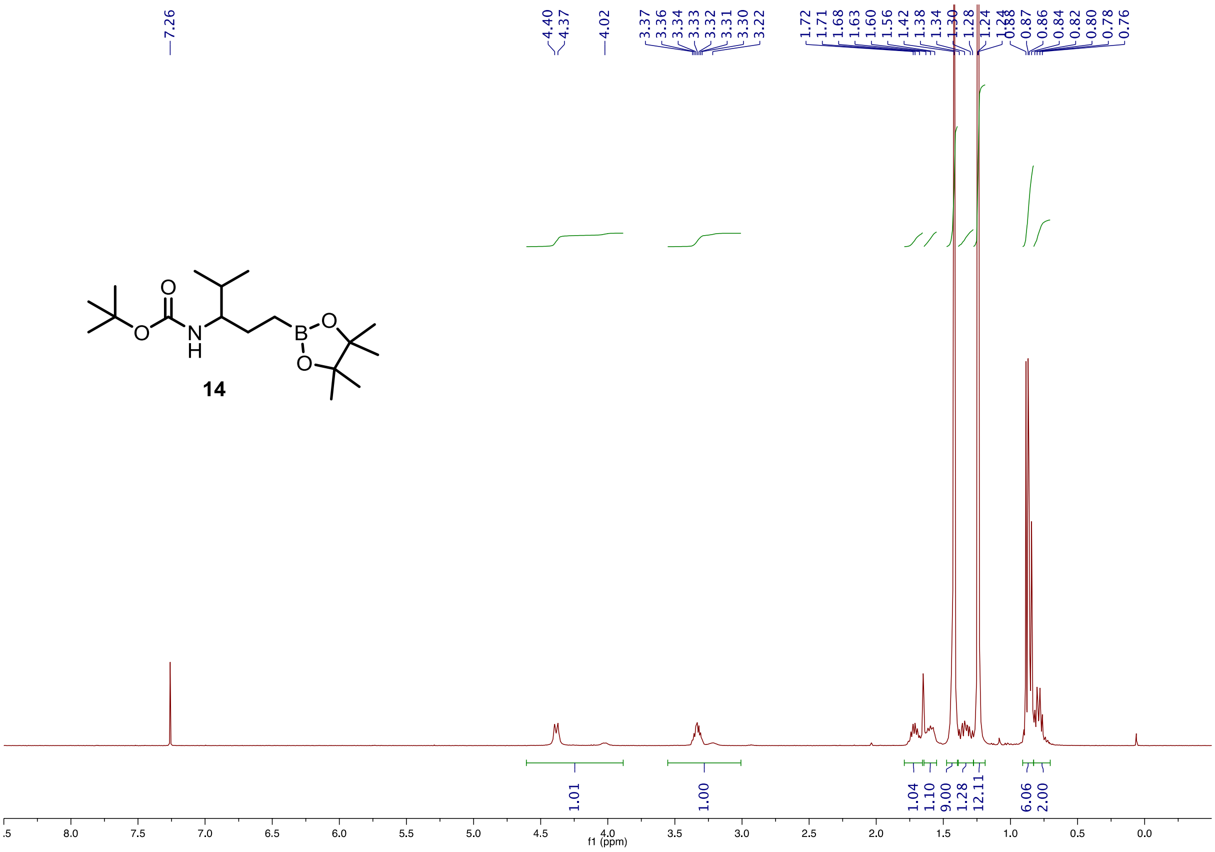

^{13}C NMR (101 MHz, $CDCl_3$)

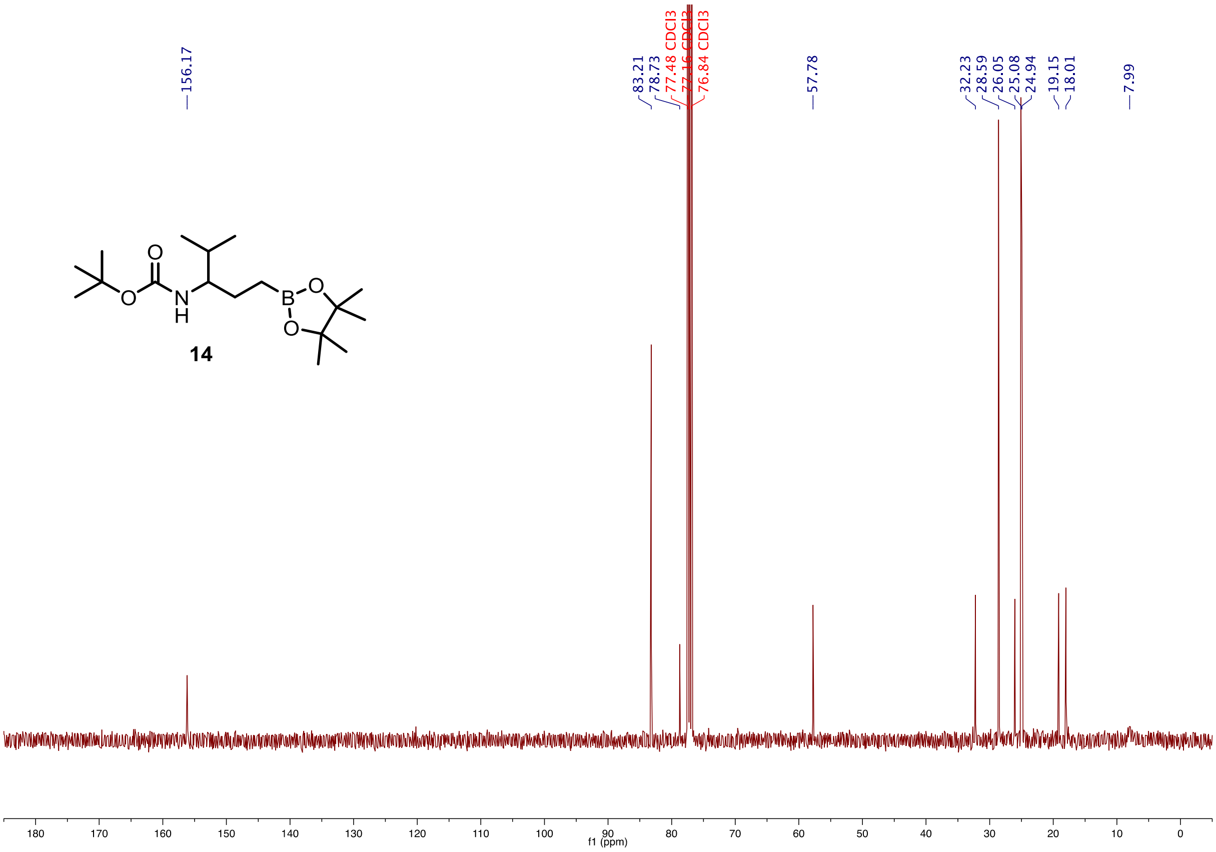

^1H NMR (500 MHz, $CDCl_3$)

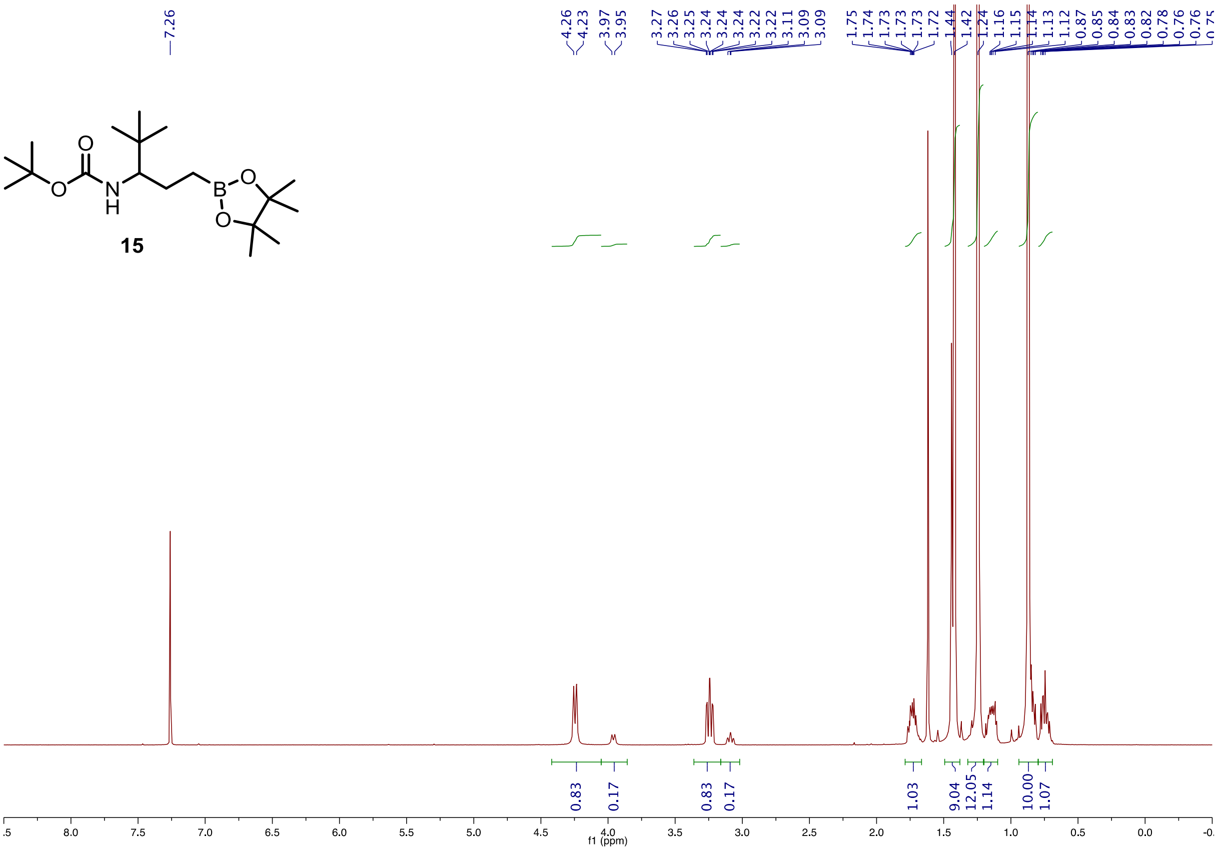

^{13}C NMR (126 MHz, $CDCl_3$)

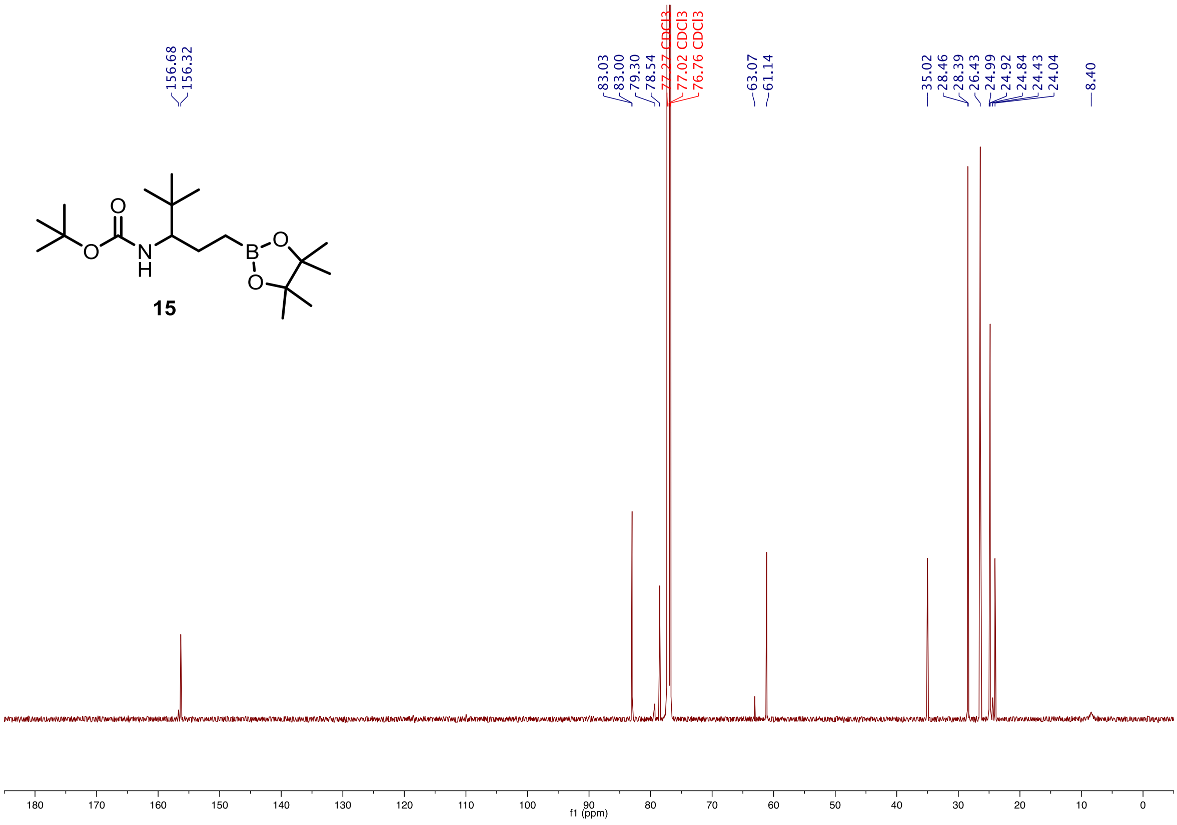

^{1}H NMR (400 MHz, $CDCl_3$)

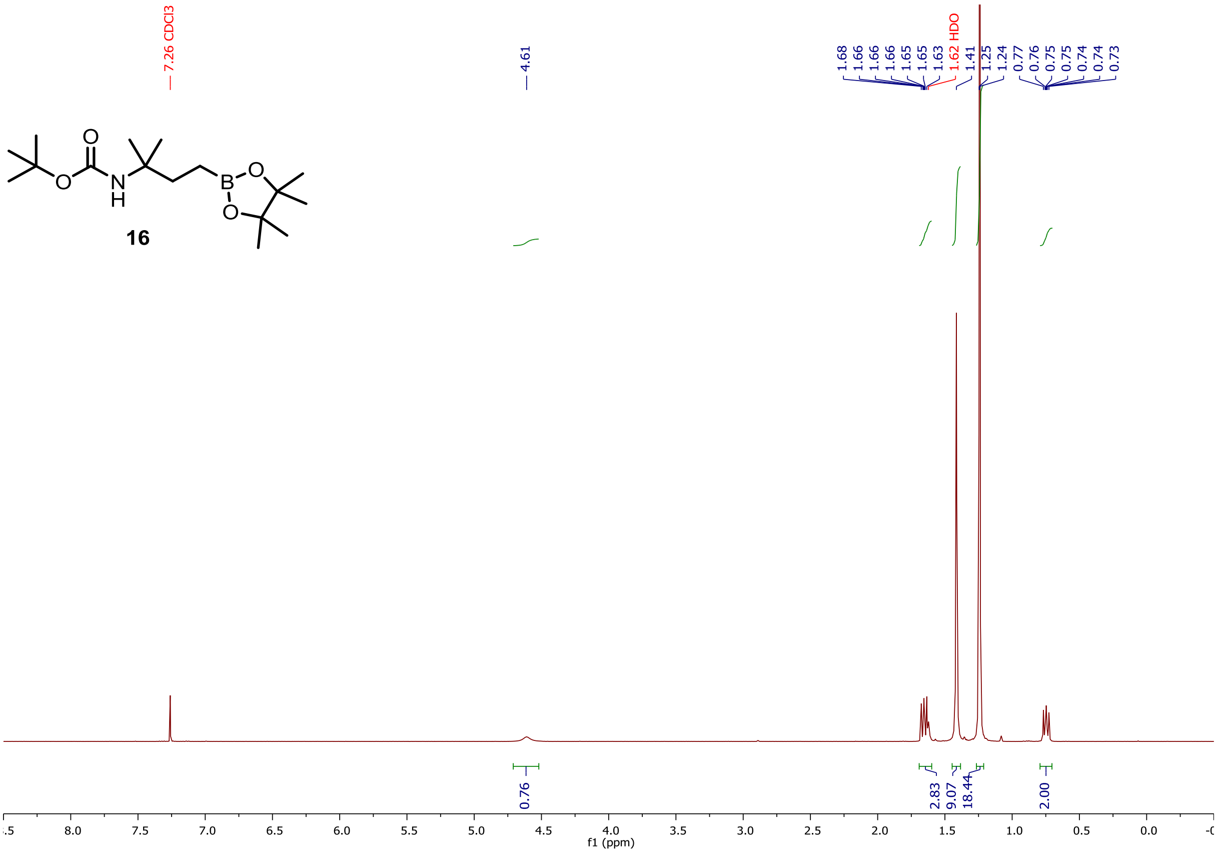

^{13}C NMR (101 MHz, $CDCl_3$)

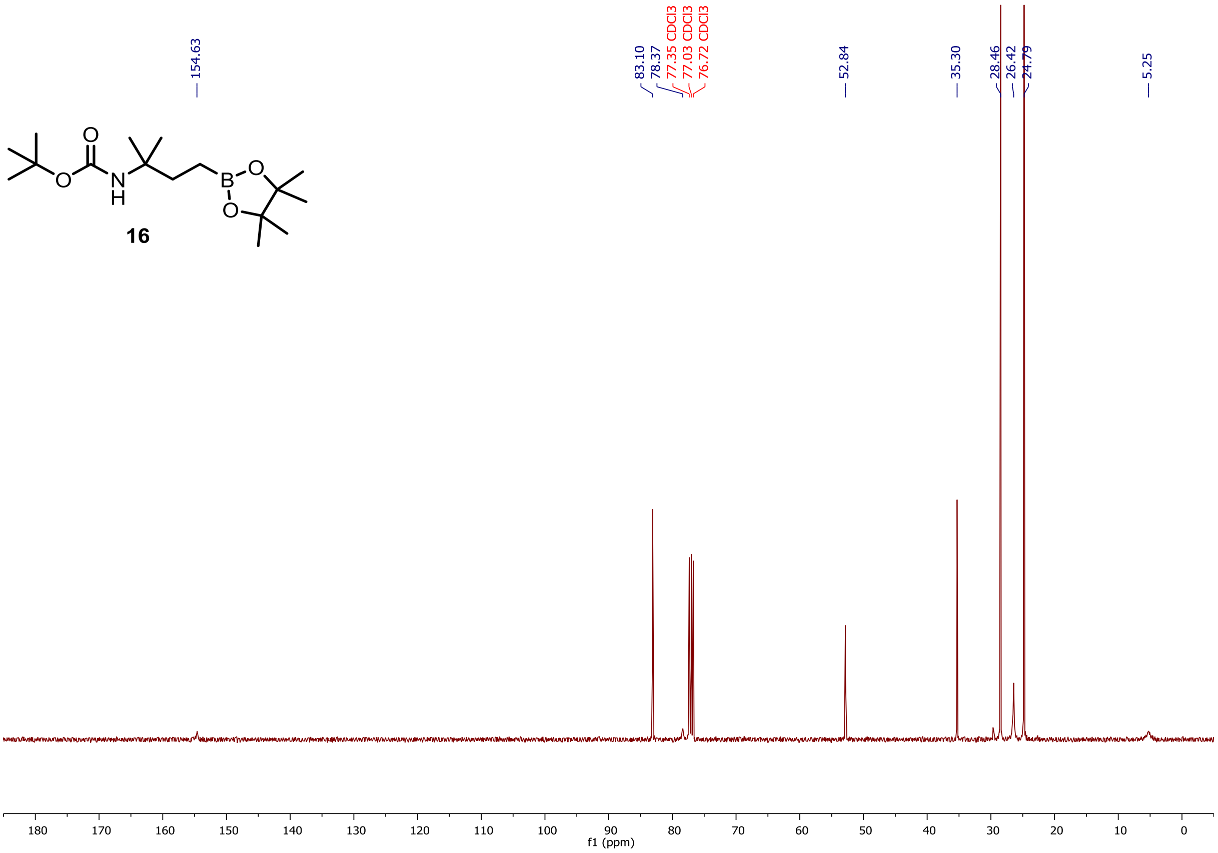

^{1}H NMR (400 MHz, $CDCl_3$)

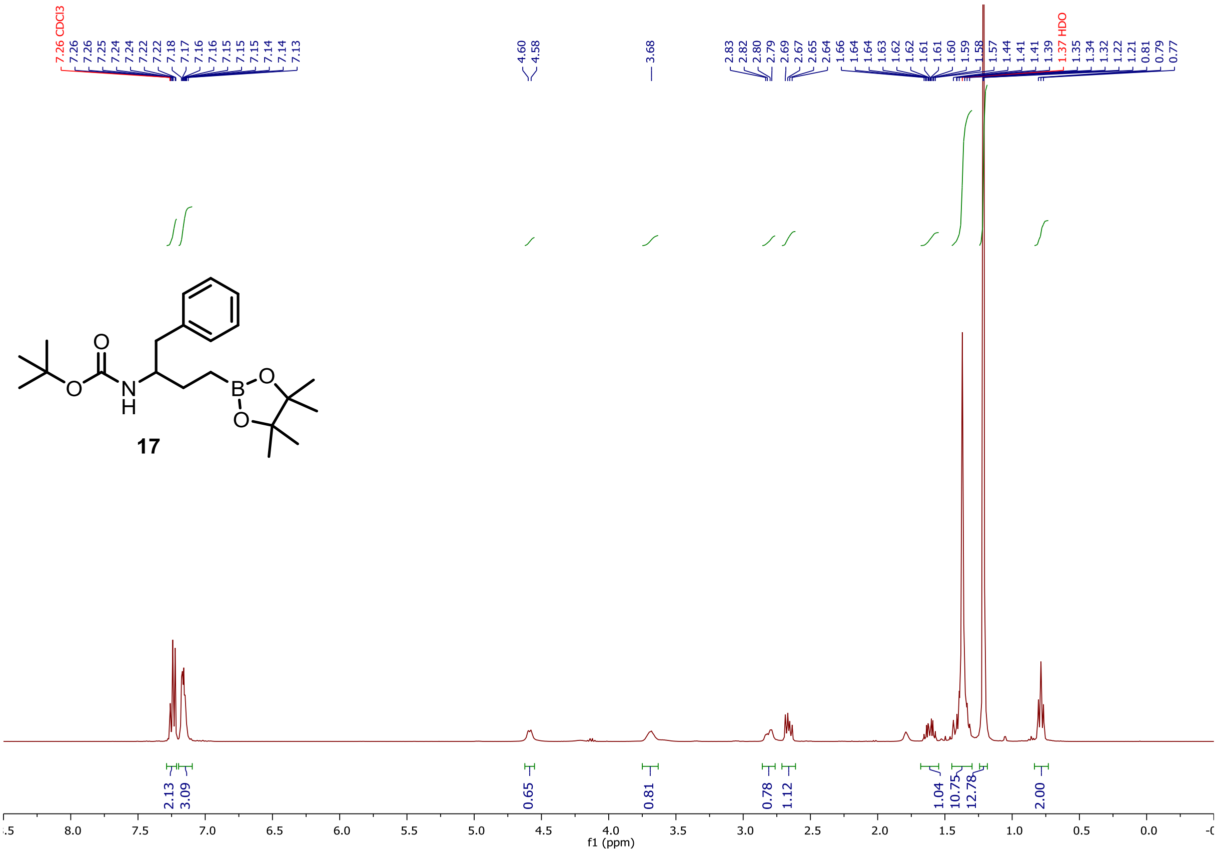

^{13}C NMR (101 MHz, $CDCl_3$)

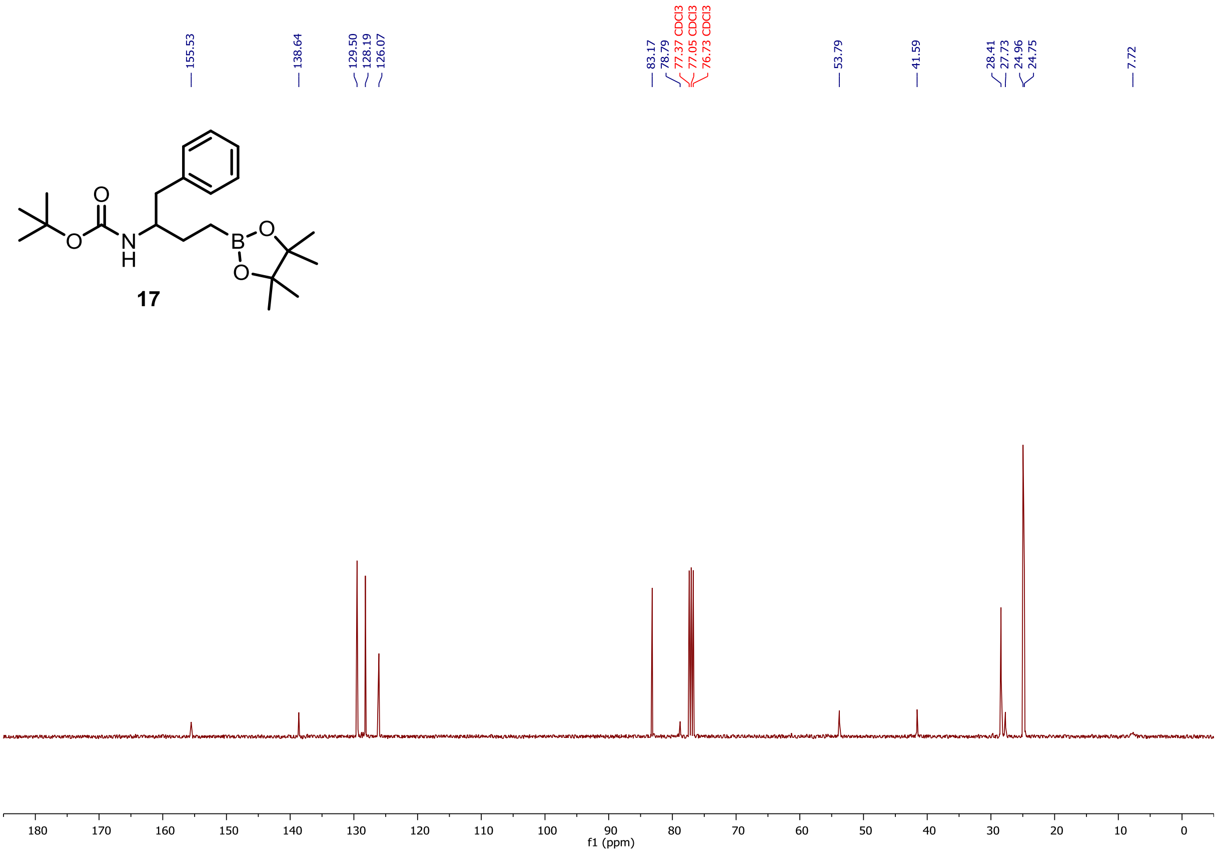

^{1}H NMR (400 MHz, $CDCl_3$)

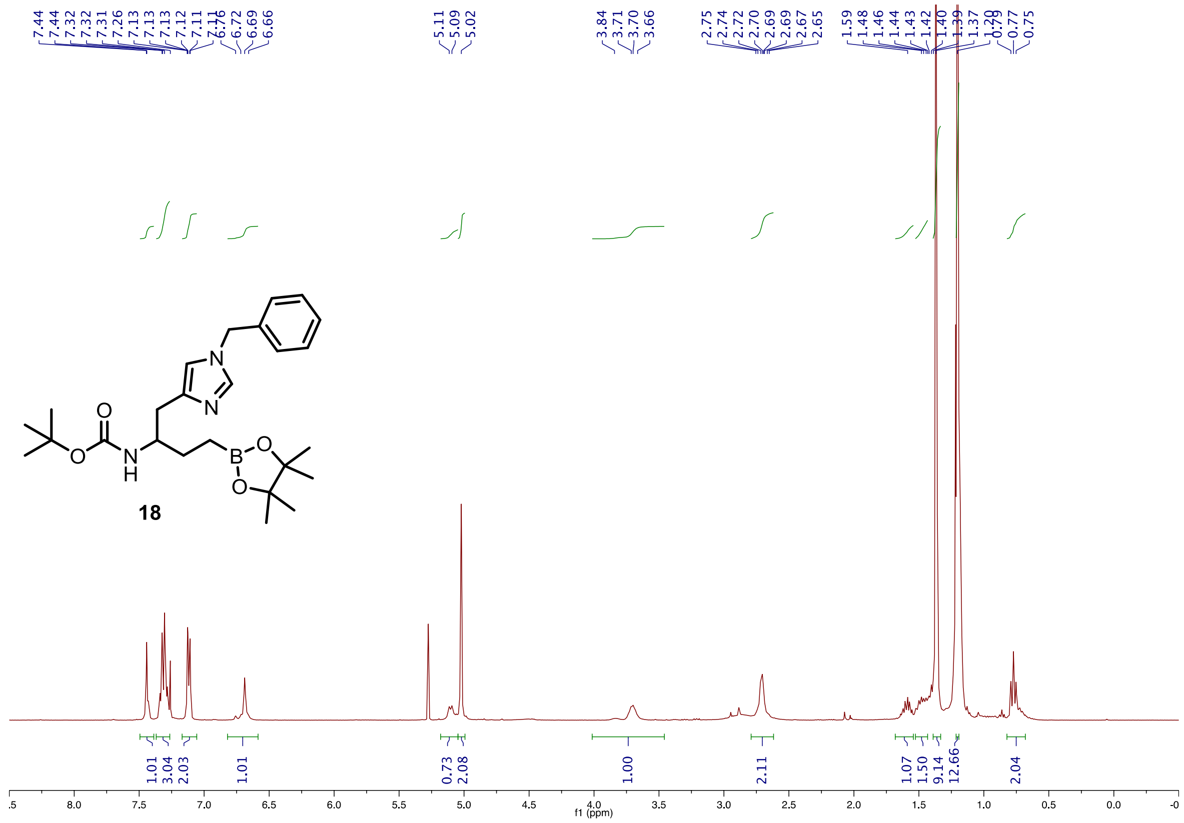

^{13}C NMR (101 MHz, $CDCl_3$)

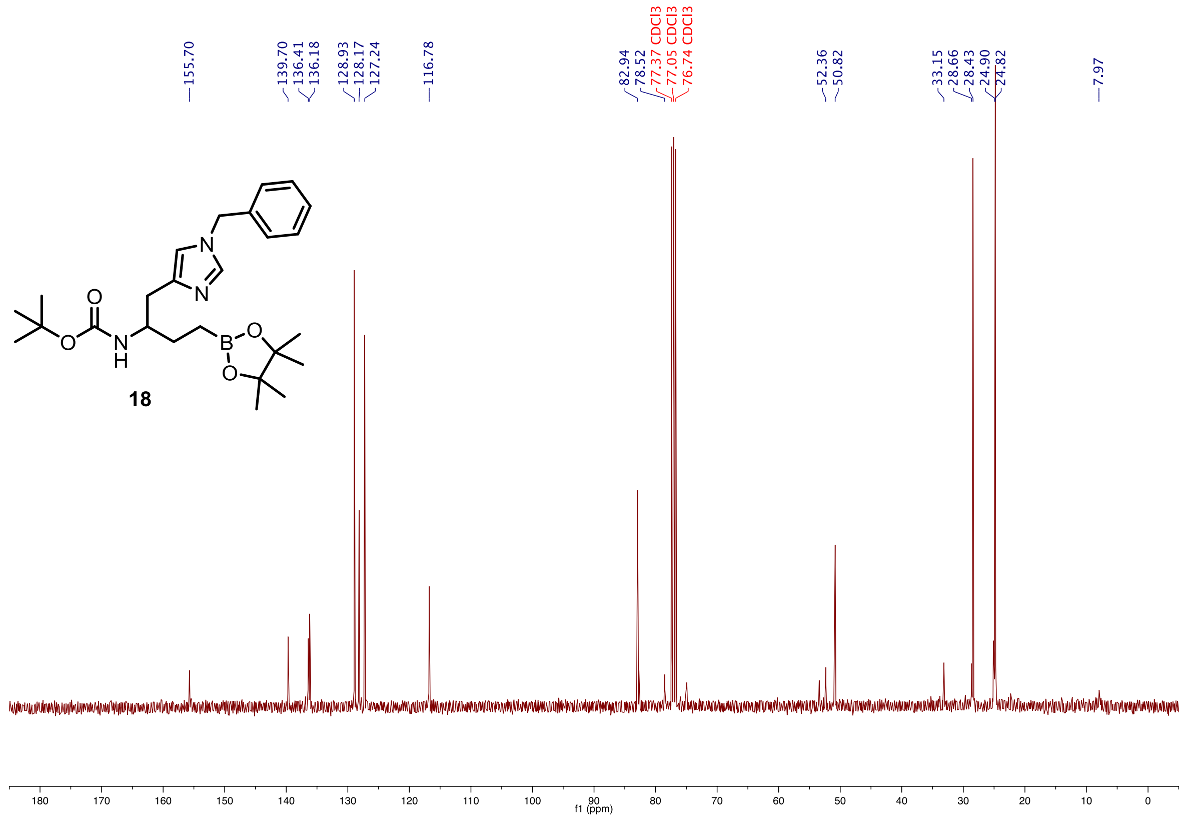

^{1}H NMR (500 MHz, $CDCl_3$)

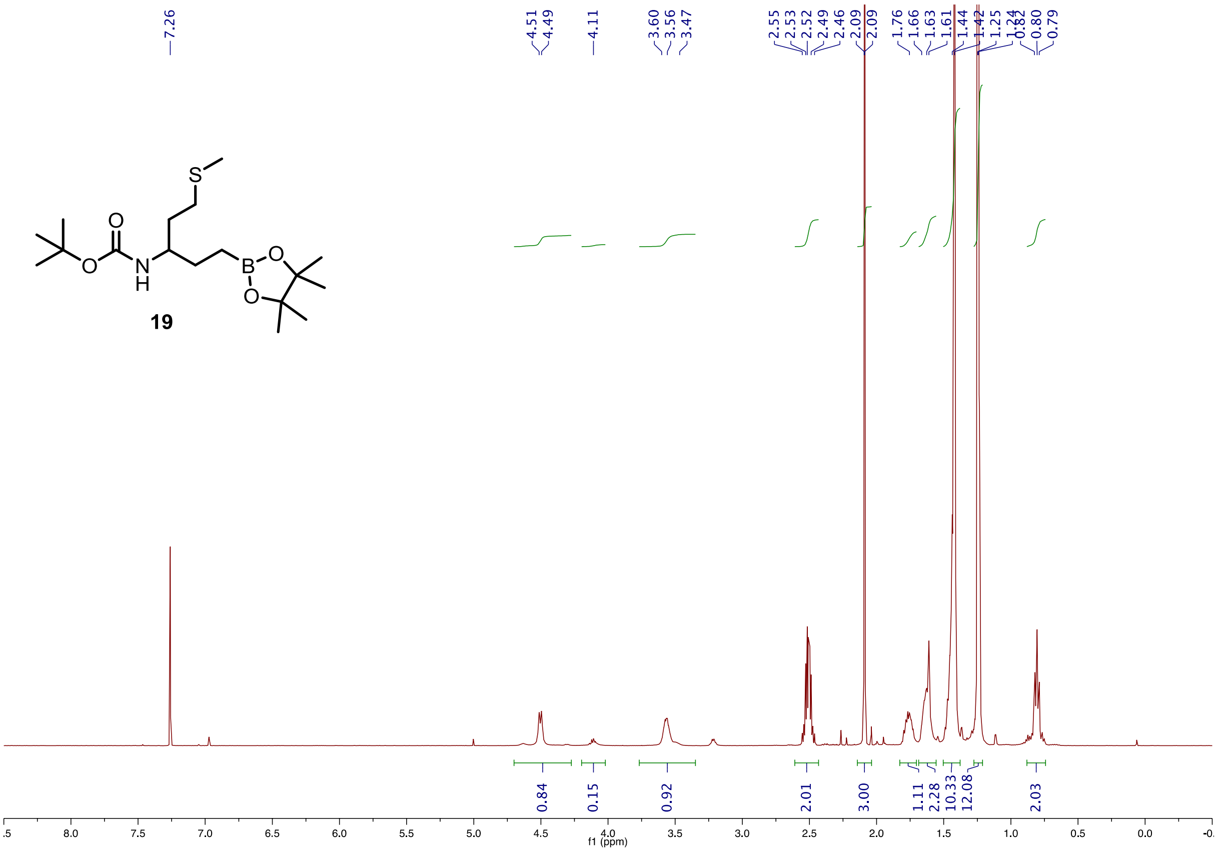

^{13}C NMR (126 MHz, $CDCl_3$)

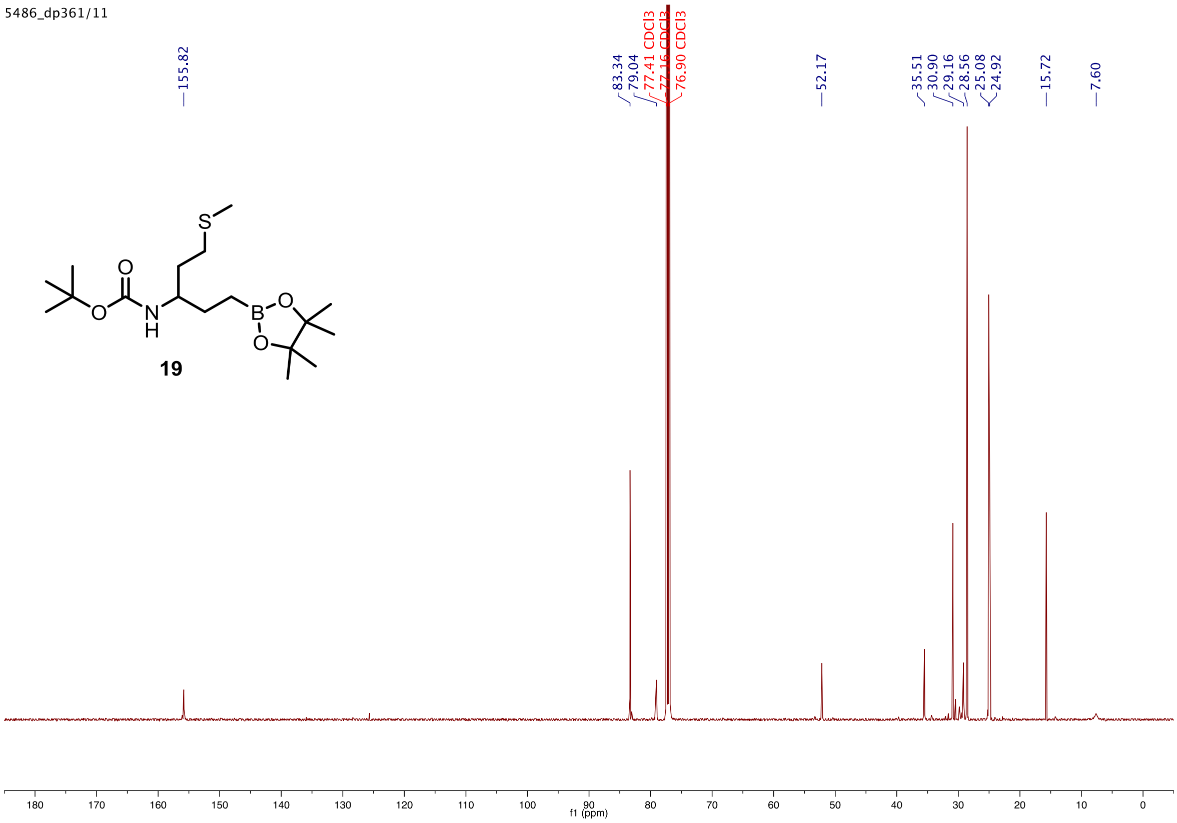

^{1}H NMR (400 MHz, $CDCl_3$)

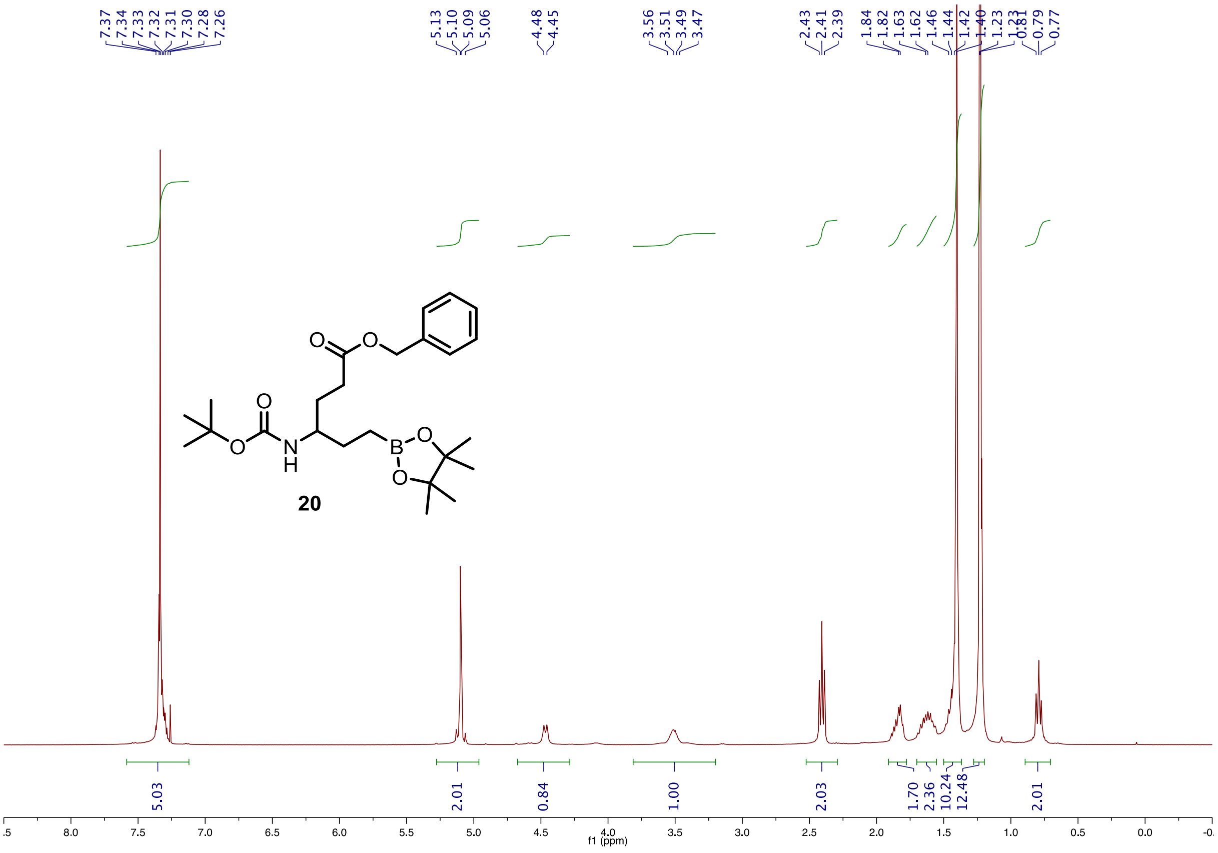

^{13}C NMR (101 MHz, $CDCl_3$)

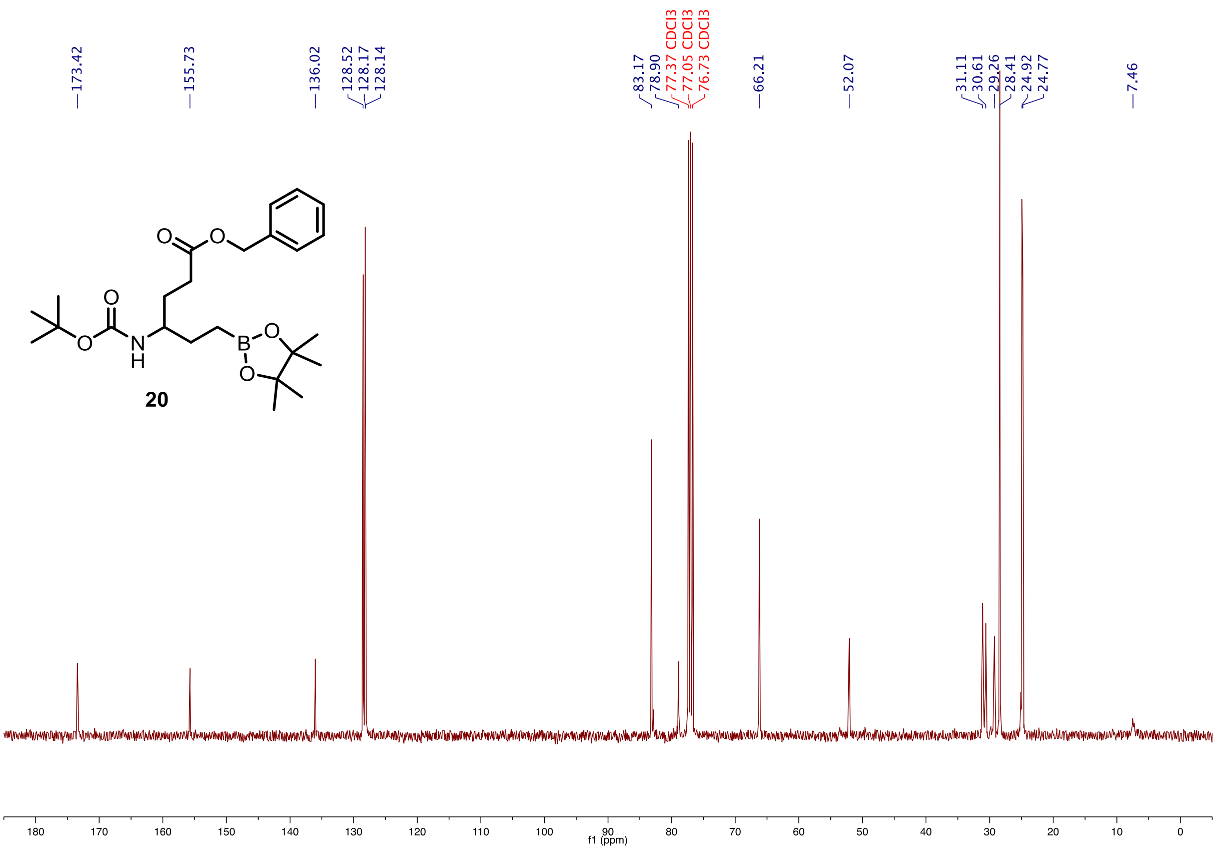

^{1}H NMR (400 MHz, $CDCl_3$)

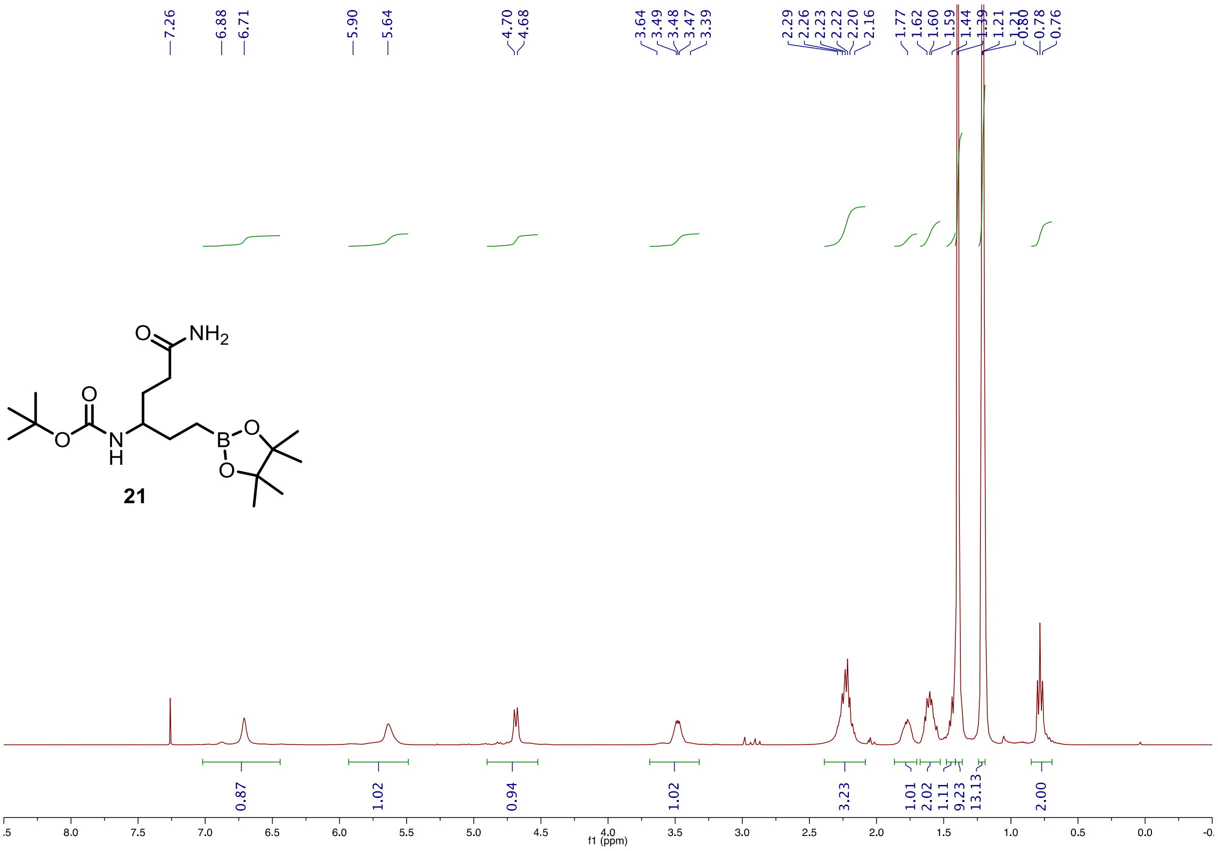

^{13}C NMR (101 MHz, $CDCl_3$)

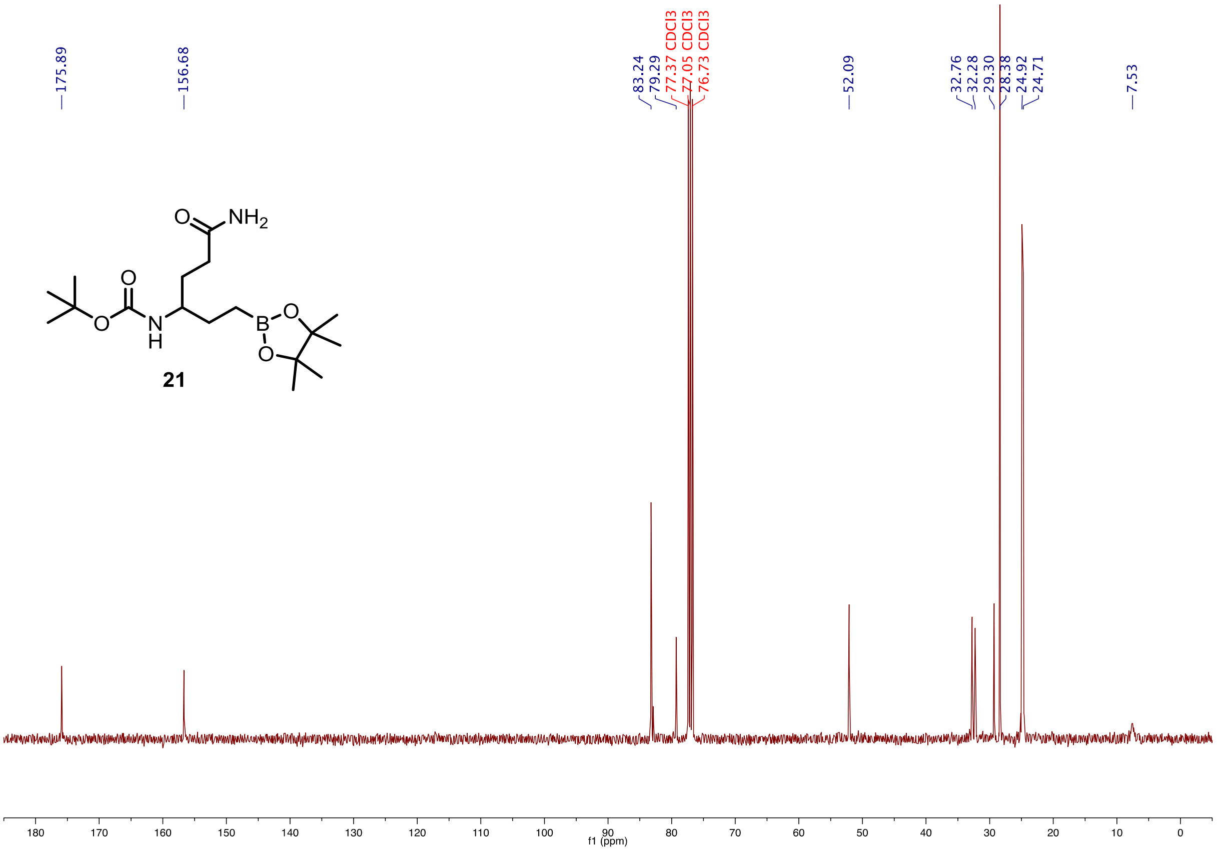

^{1}H NMR (400 MHz, $CDCl_3$)

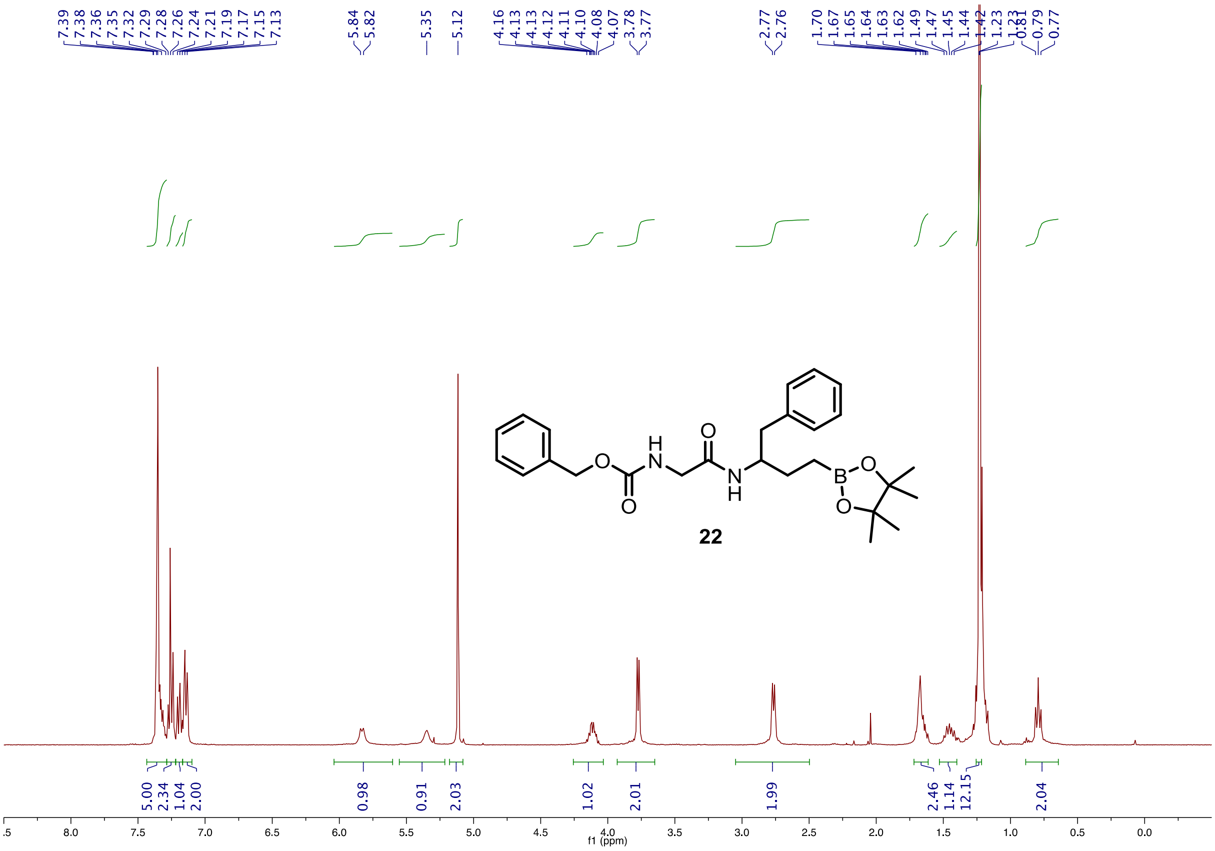

^{13}C NMR (101 MHz, $CDCl_3$)

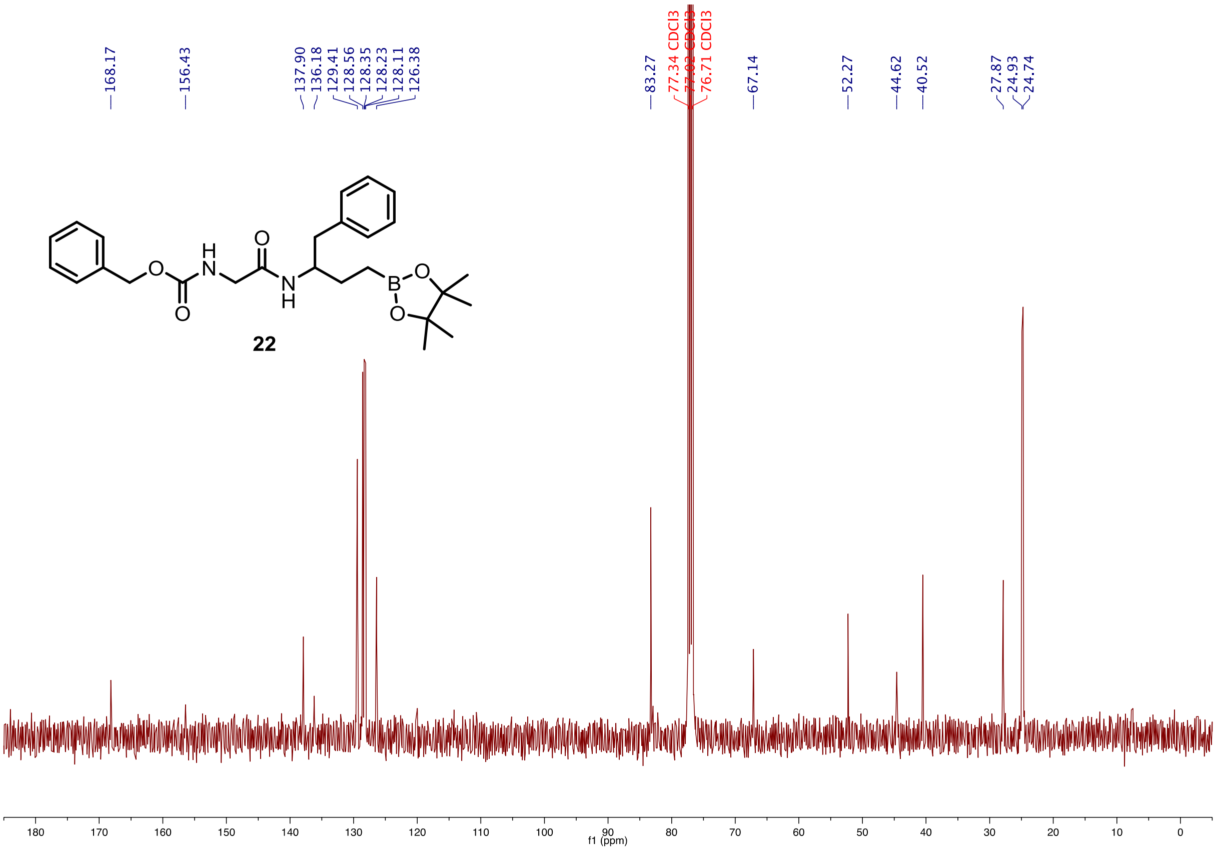

^{1}H NMR (400 MHz, $CDCl_3$)

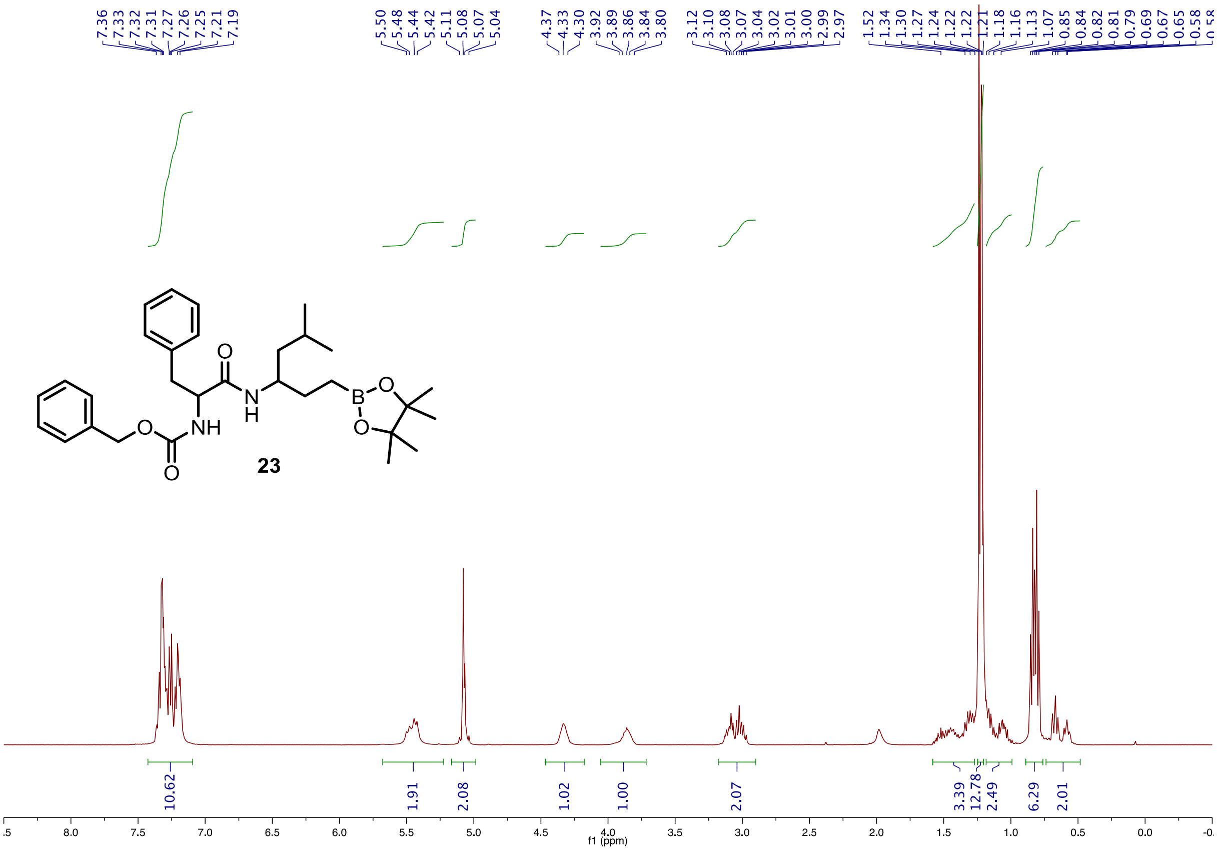

^{13}C NMR (101 MHz, $CDCl_3$)

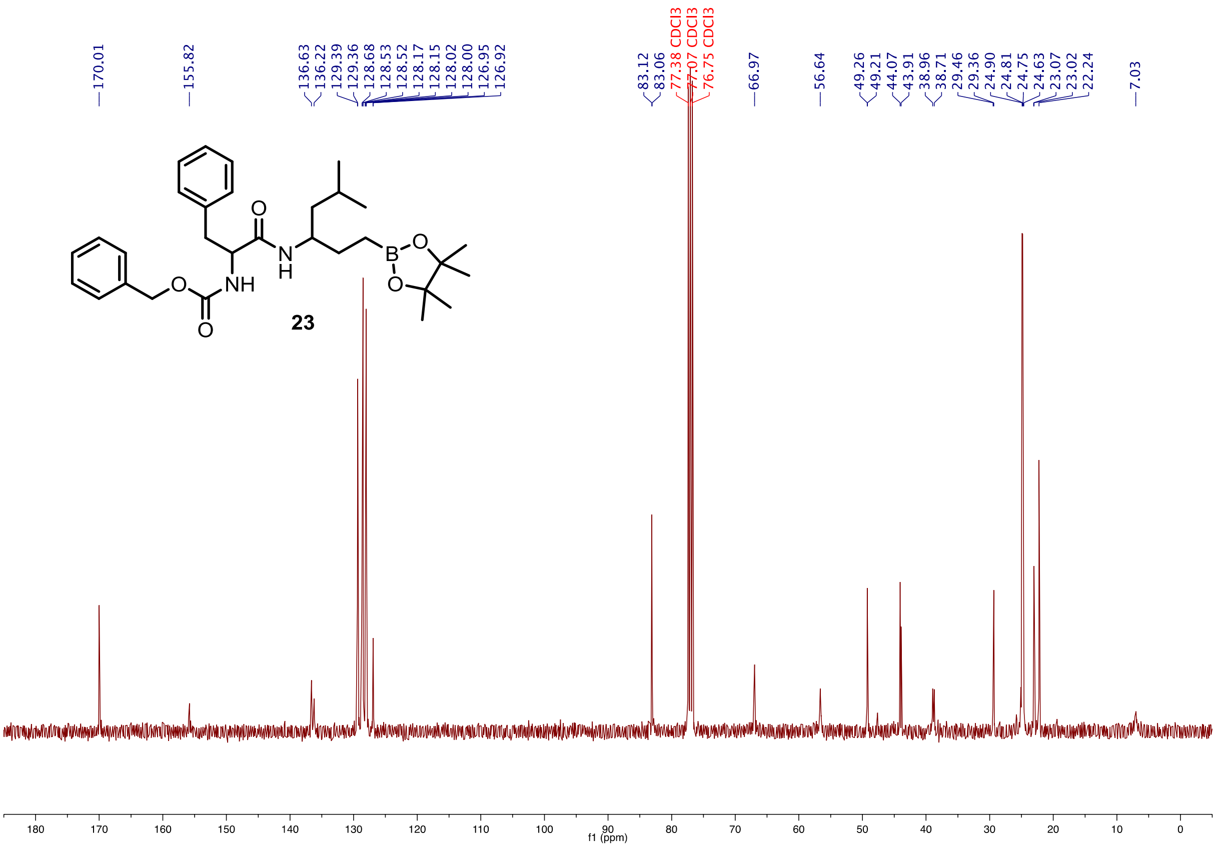

^{1}H NMR (500 MHz, DMSO-d_6, 100 ºC):

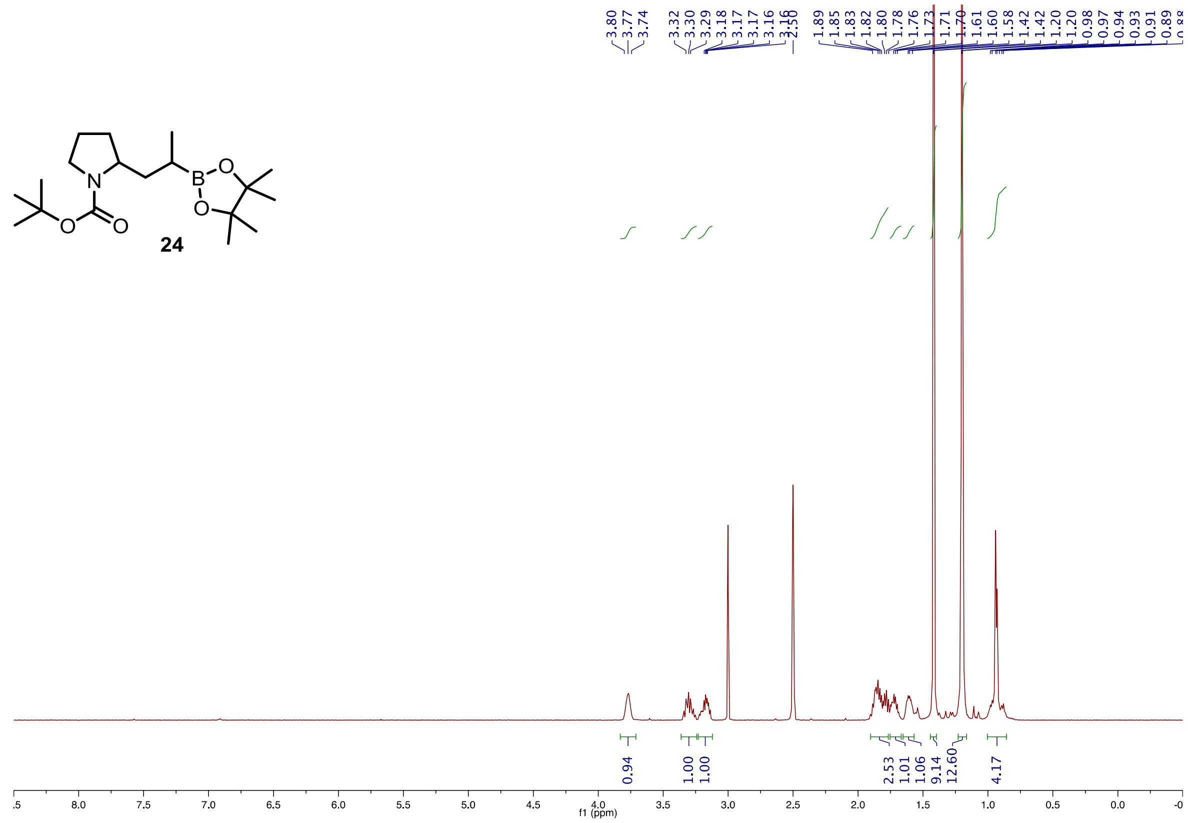

^{13}C NMR (126 MHz, DMSO-d_6, 100 ºC):

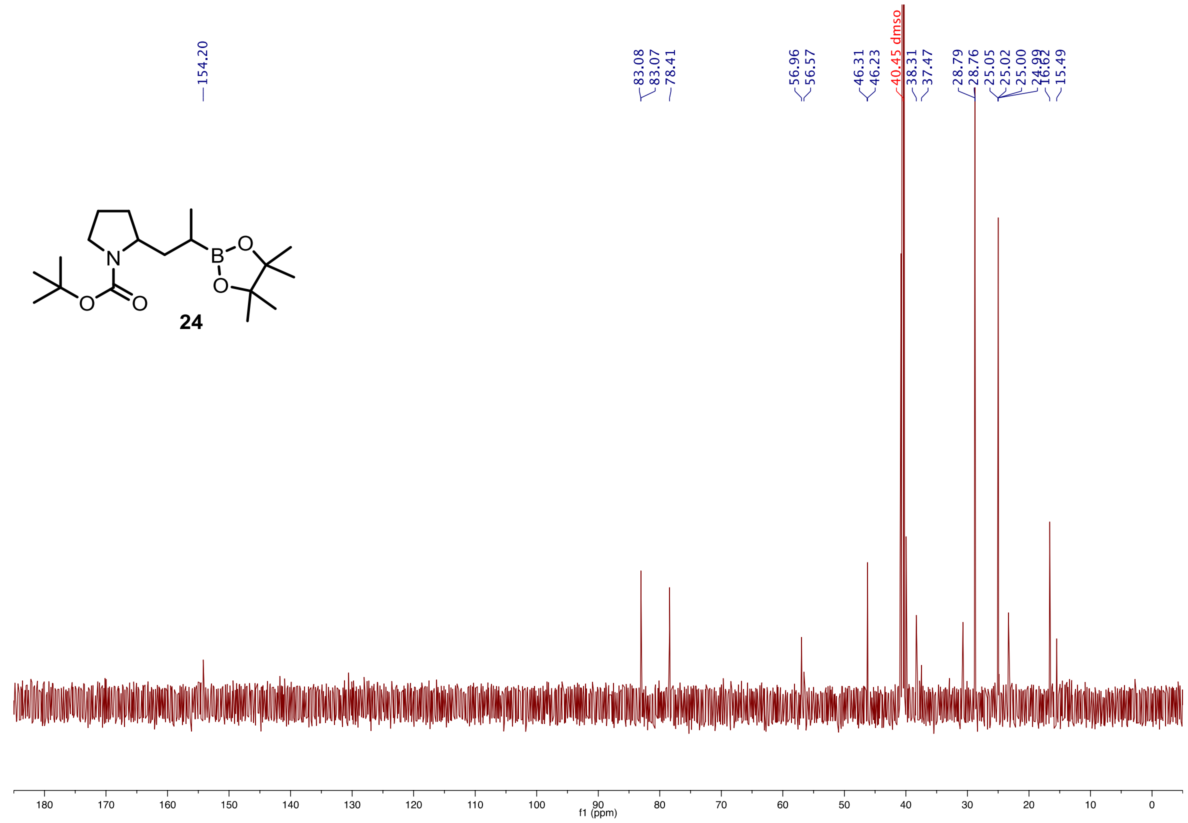

^{1}H NMR (500 MHz, DMSO-d_6, 100 °C)

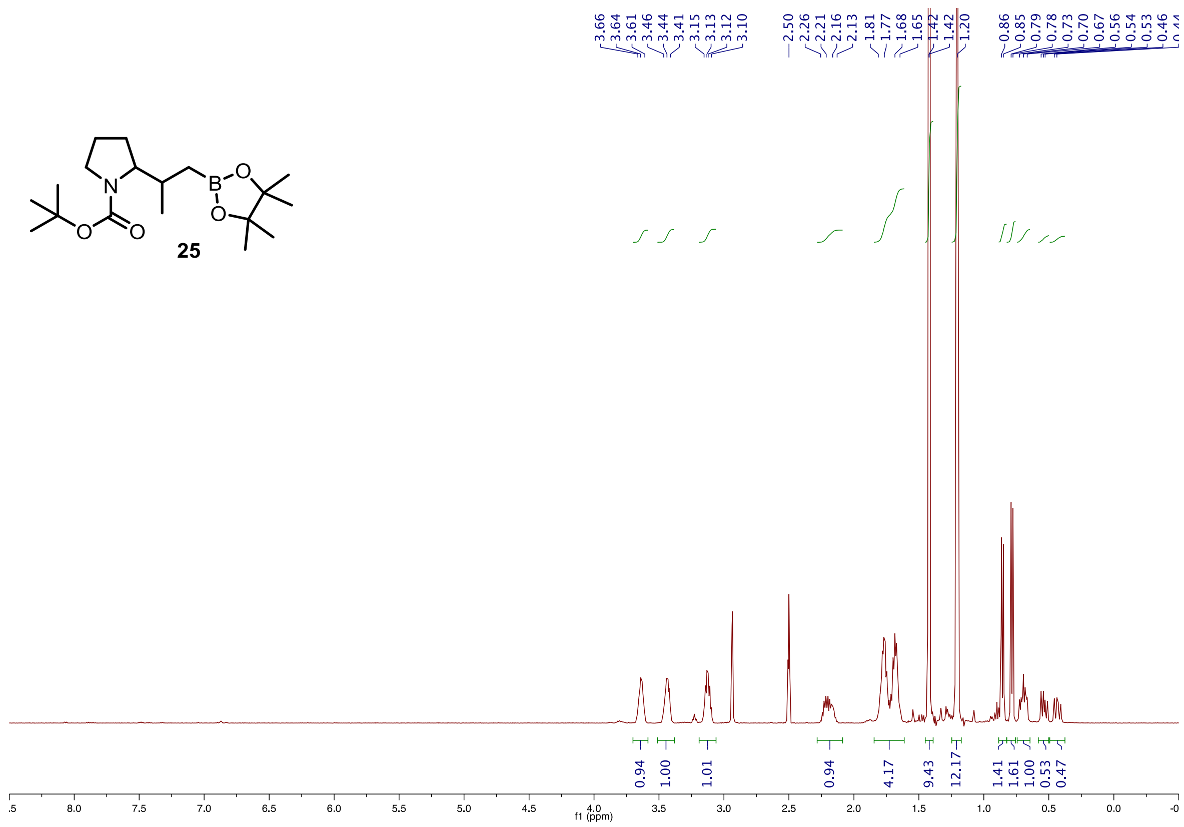

^{13}C NMR (126 MHz, DMSO-d_6, 100 °C)

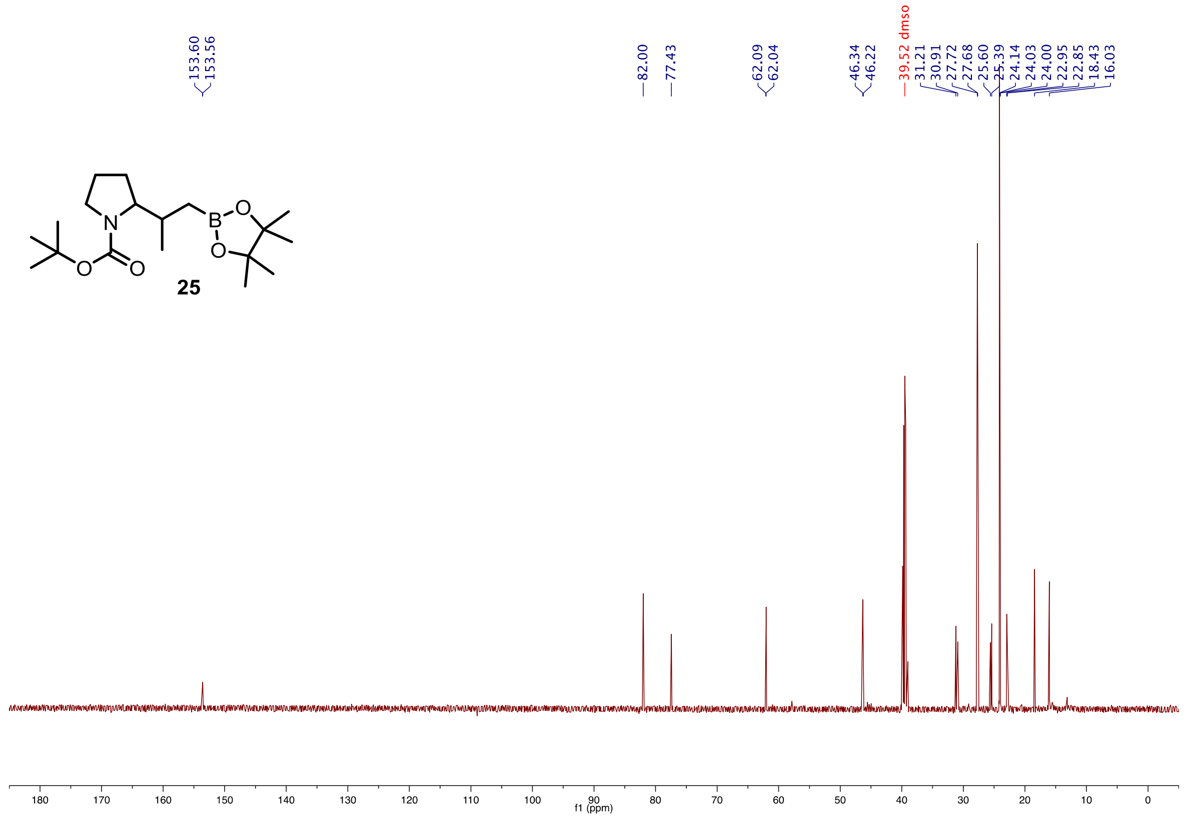

^{1}H NMR (500 MHz, DMSO-d_6, 100 °C)

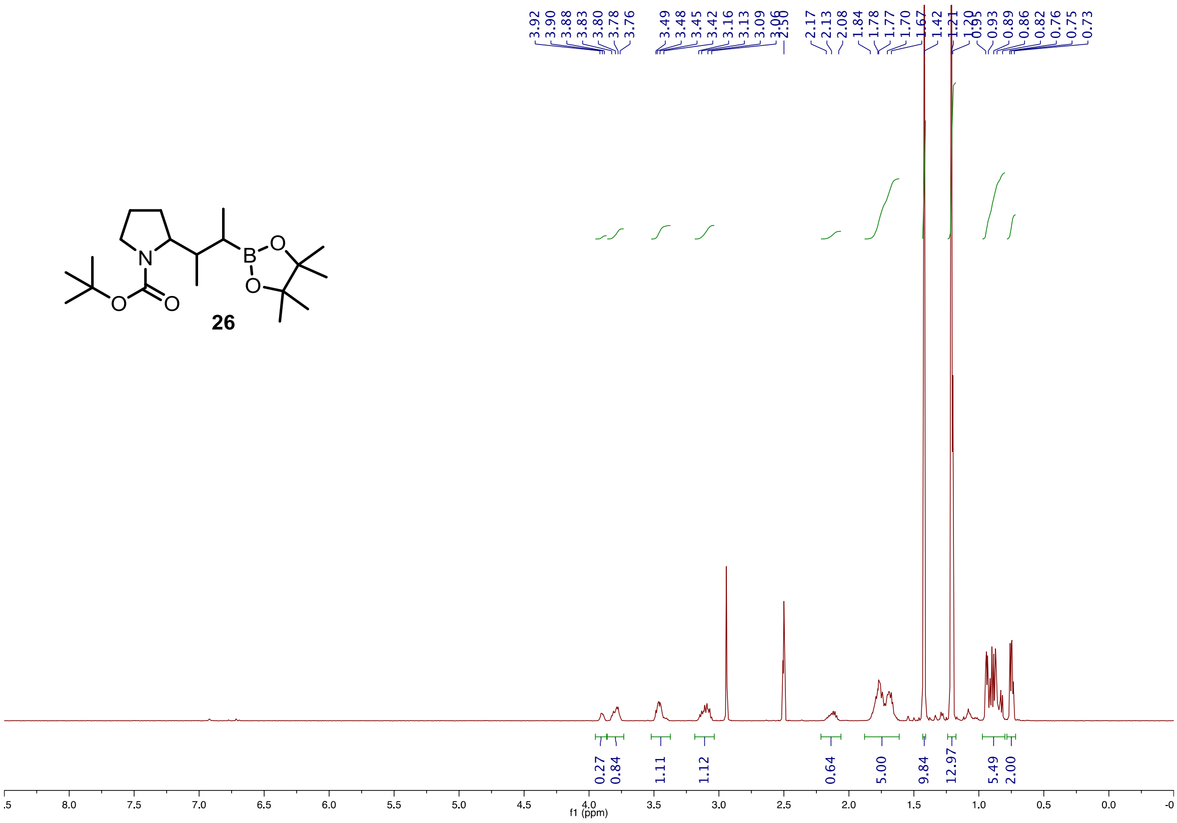

^{13}C NMR (126 MHz, DMSO-d_6, 100 °C)

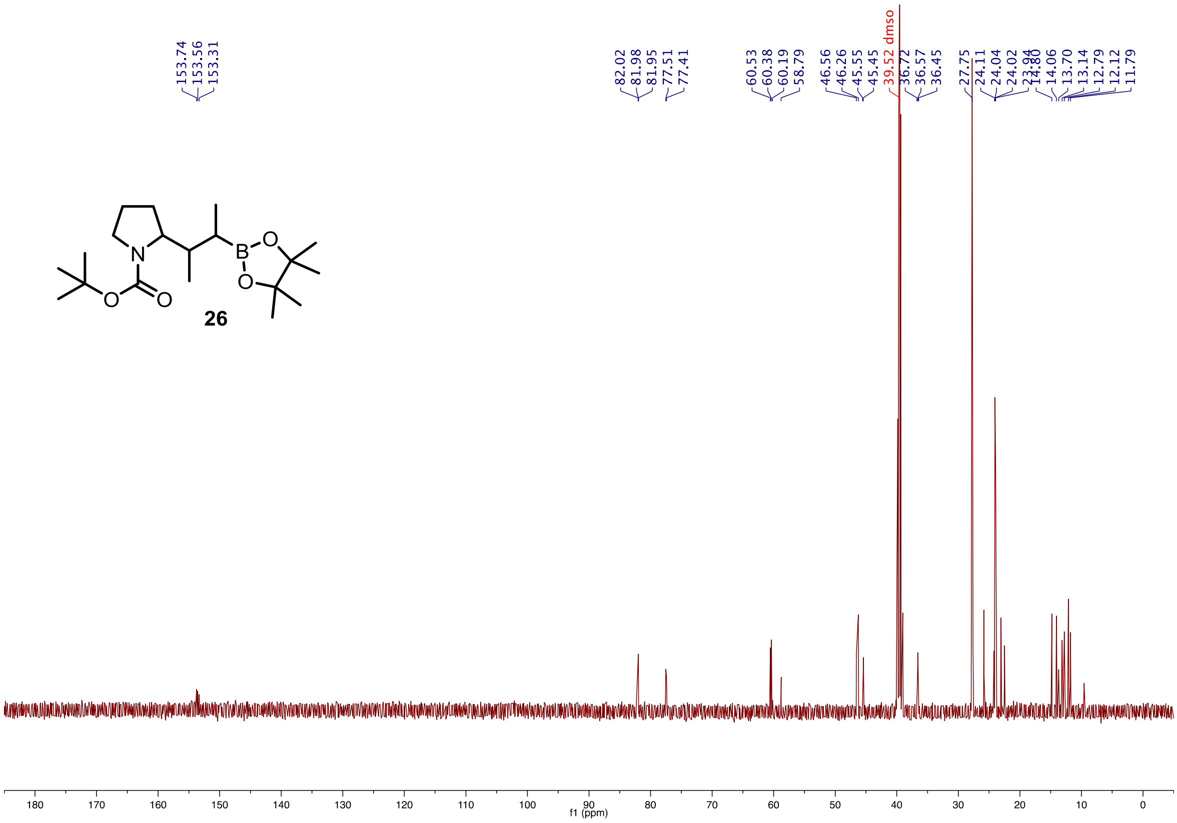

^{1}H NMR (500 MHz, DMSO-d_6, 100 ºC): Major diastereomer.

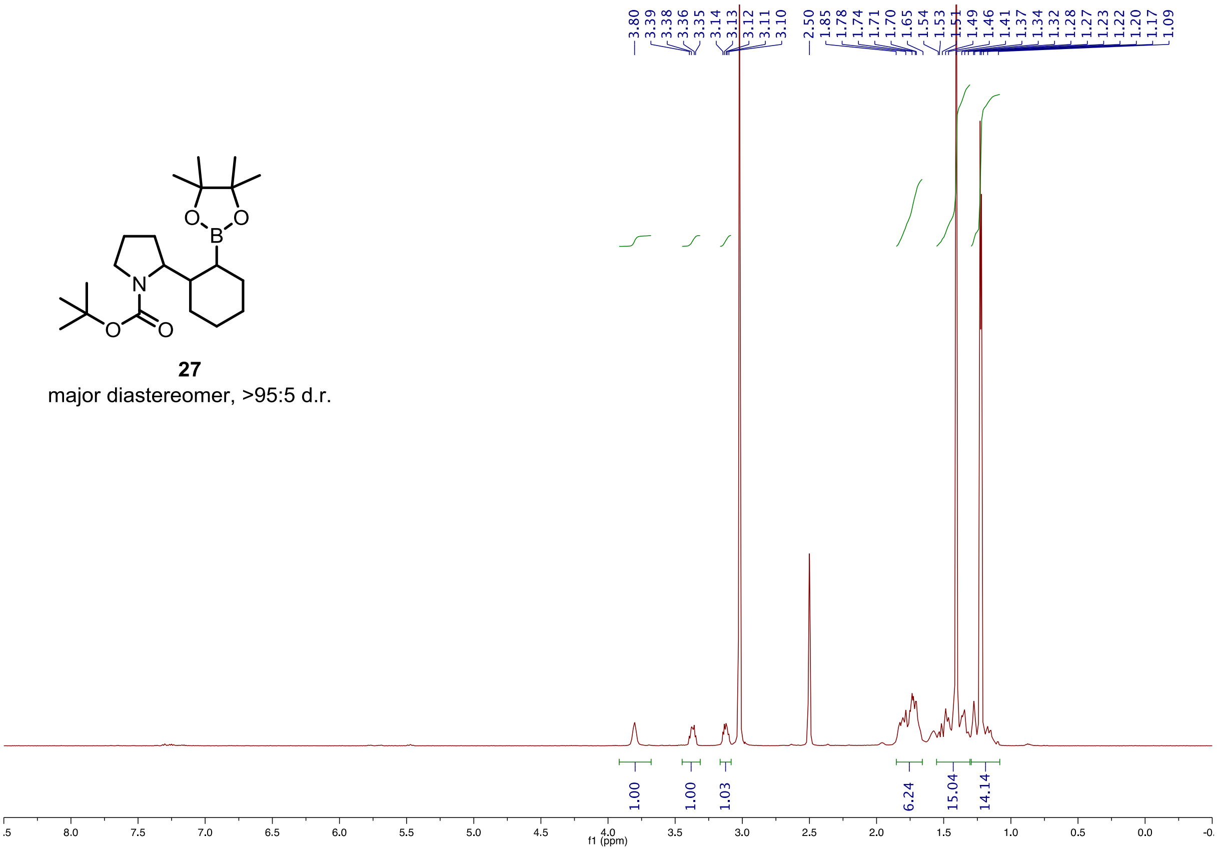

27
major diastereomer, >95:5 d.r.

^{13}C NMR (126 MHz, DMSO-d_6, 100 ºC): Major diastereomer.

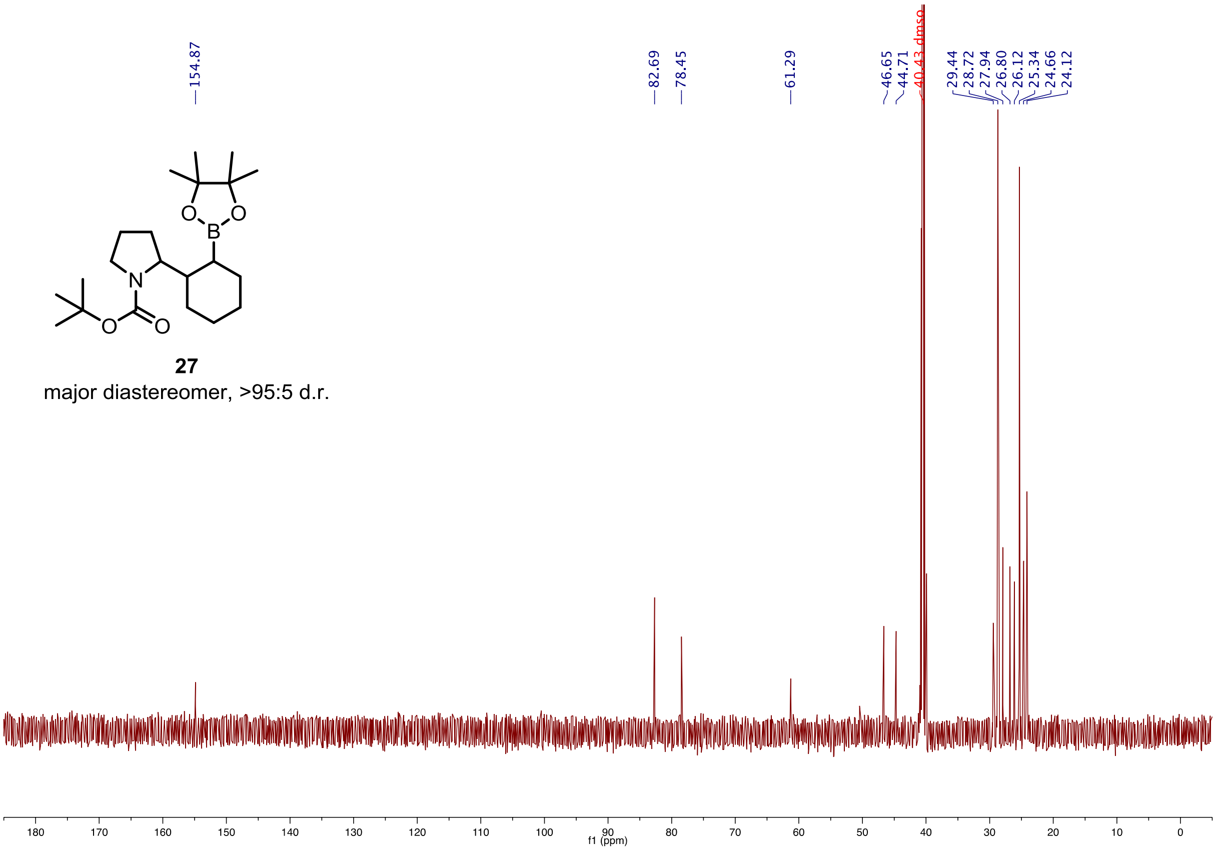

27
major diastereomer, >95:5 d.r.

^{1}H NMR (500 MHz, DMSO-d_6, 100 ºC): Minor diastereomers.

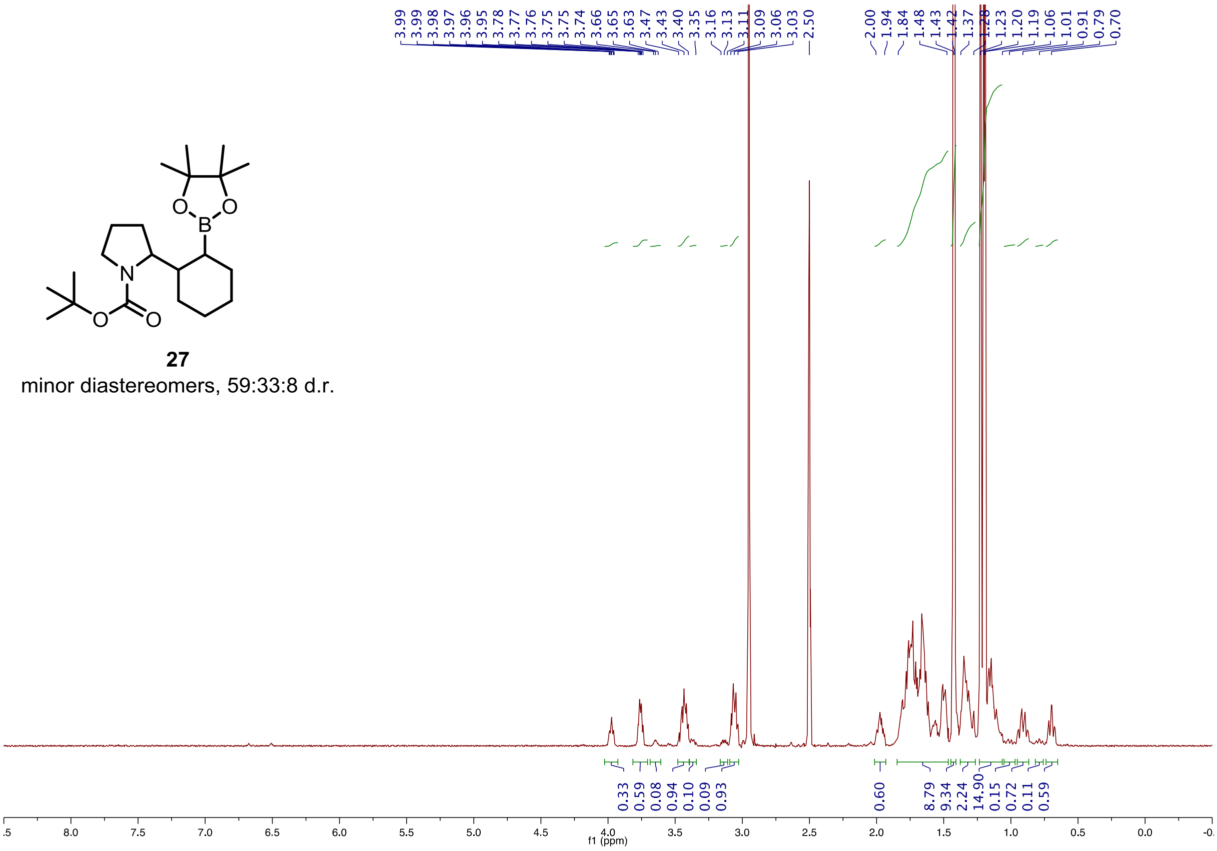

^{13}C NMR (126 MHz, DMSO-d_6, 100 ºC): Minor diastereomers.

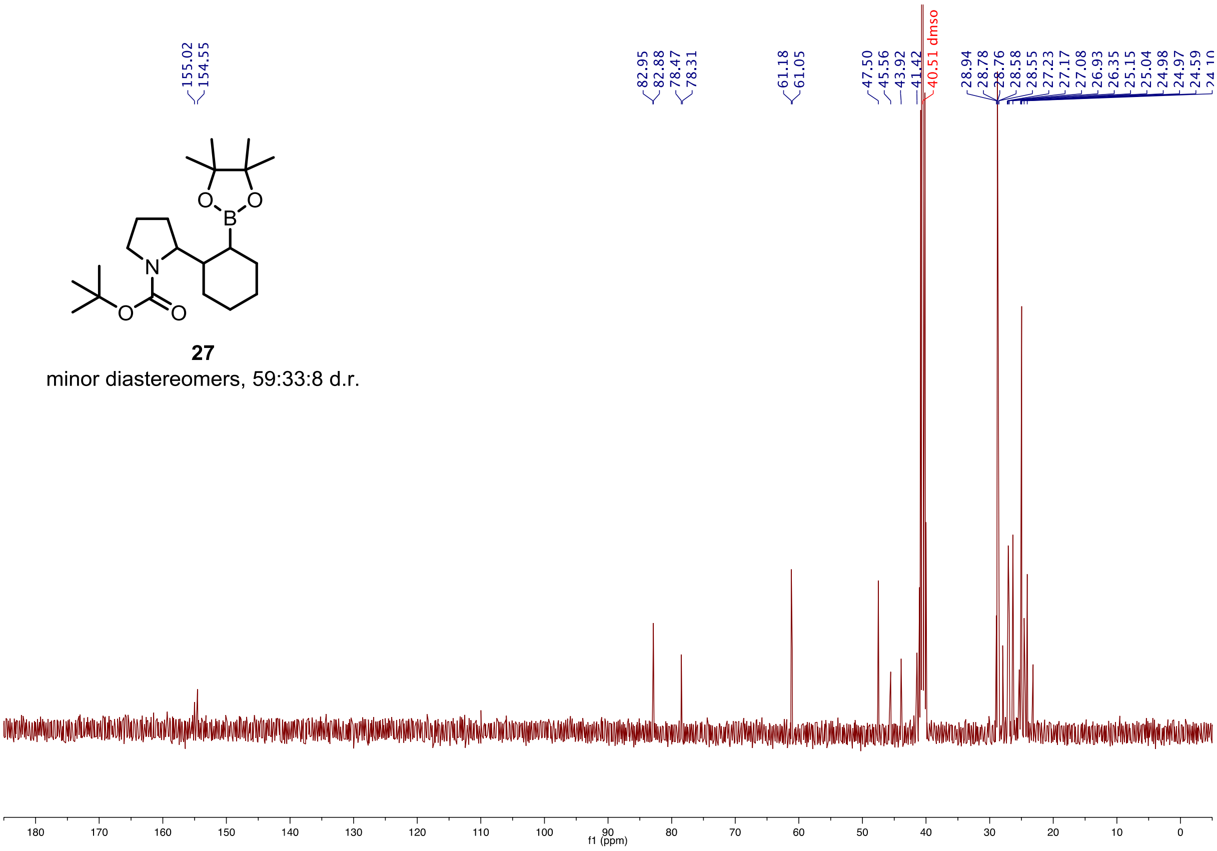

^{1}H NMR (500 MHz, DMSO-d_6, 100 ºC)

28

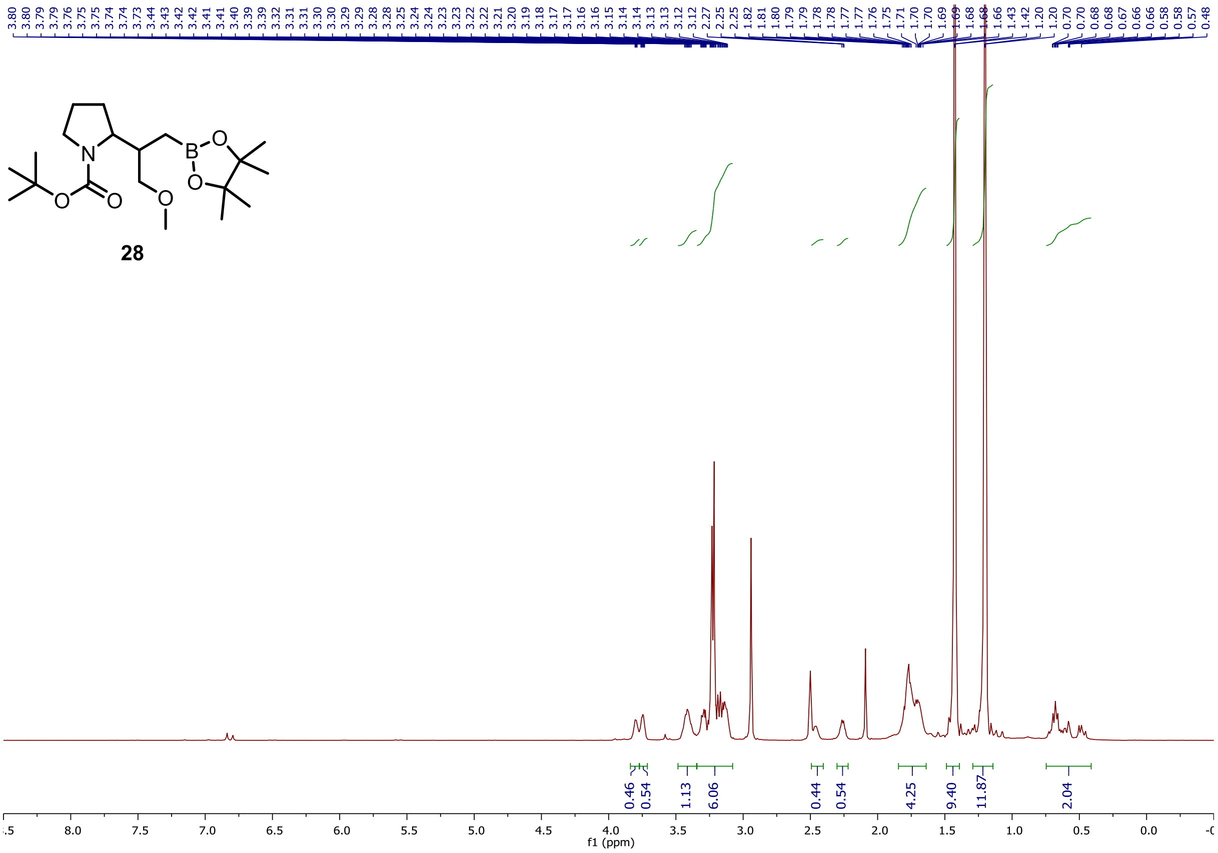

^{13}C NMR (126 MHz, DMSO-d_6, 100 ºC)

28

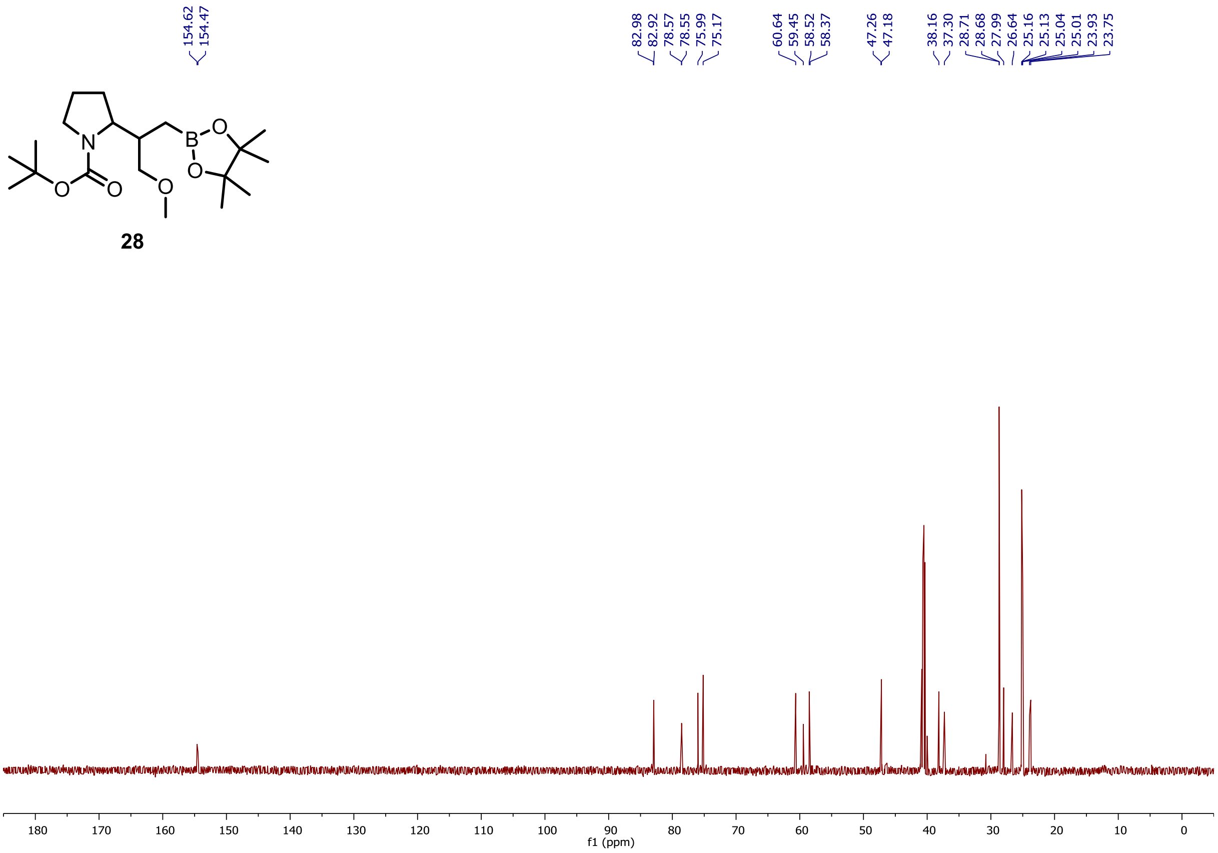

^{1}H NMR (500 MHz, DMSO-d_6, 100 ºC)

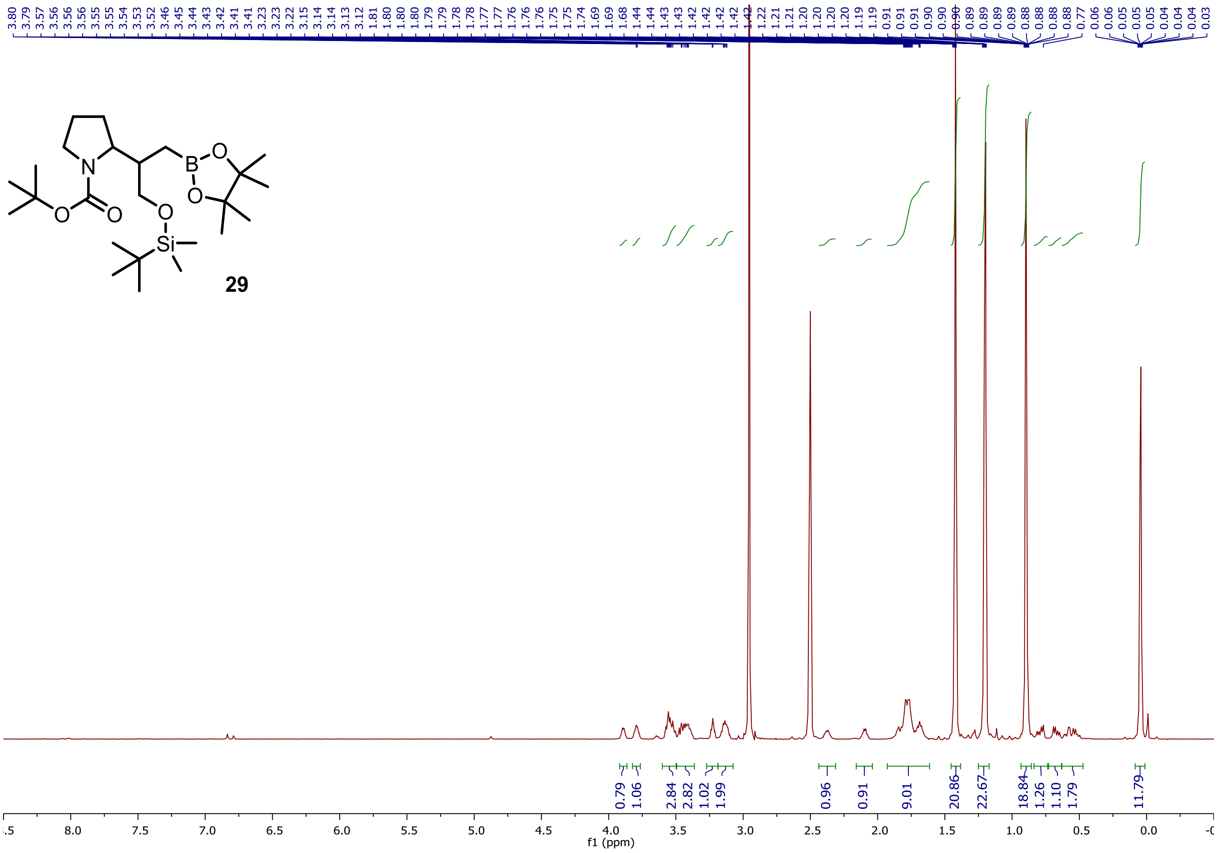

^{13}C NMR (126 MHz, DMSO-d_6, 100 ºC)

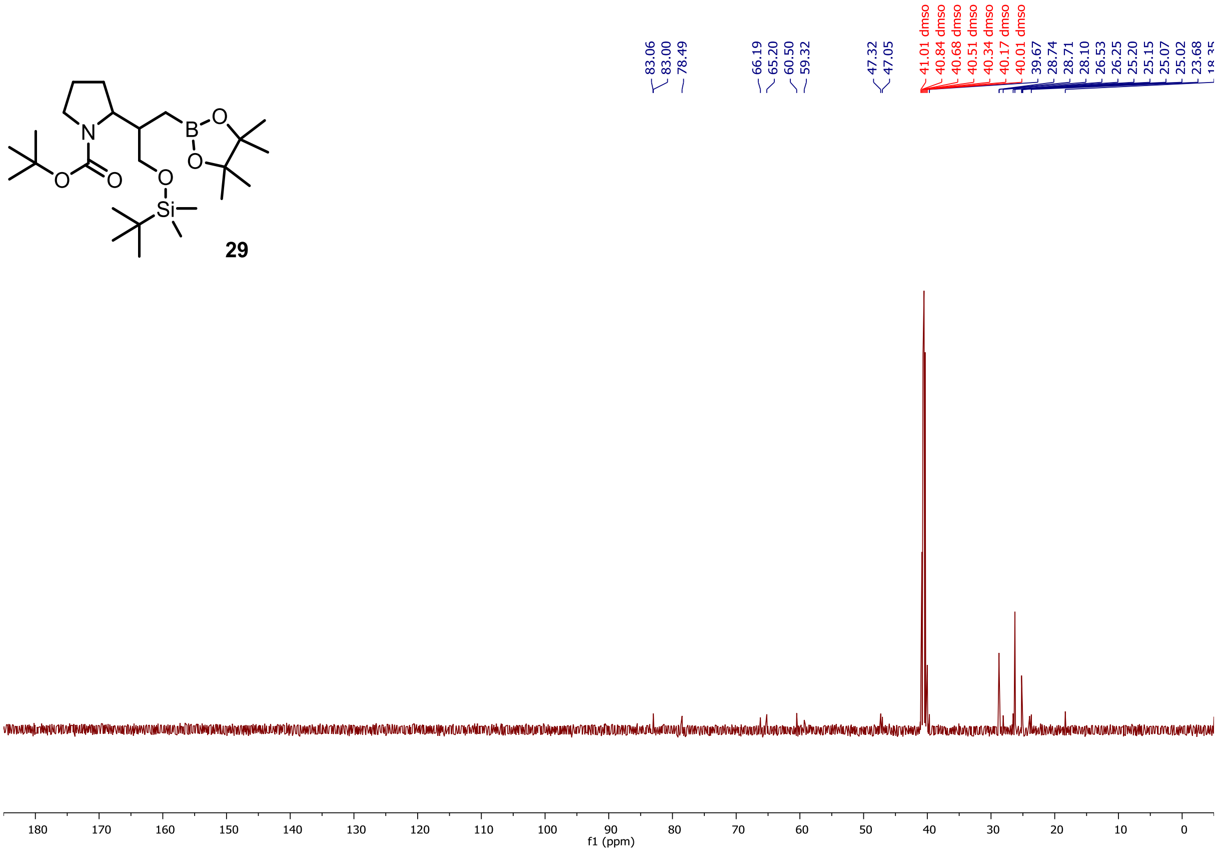

^{1}H NMR (400 MHz, $CDCl_3$)

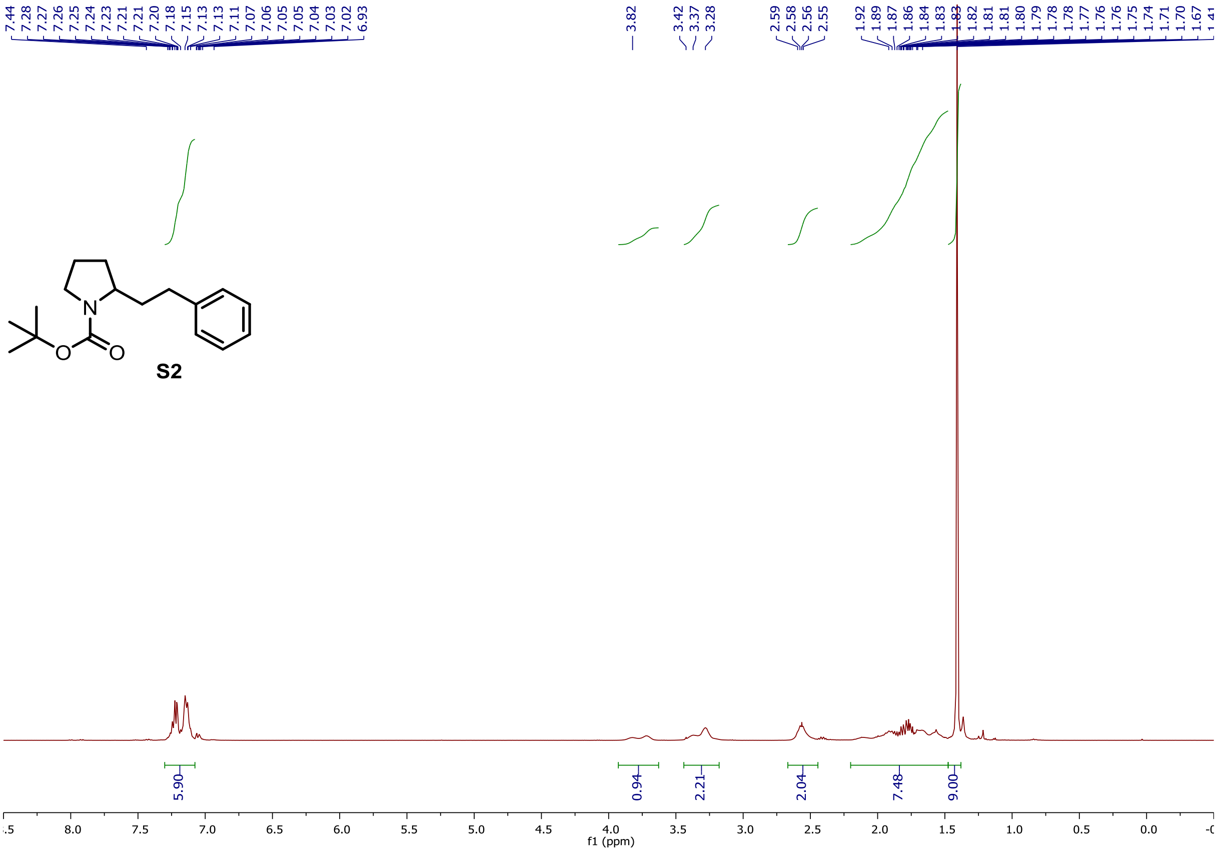

^{13}C NMR (101 MHz, $CDCl_3$)

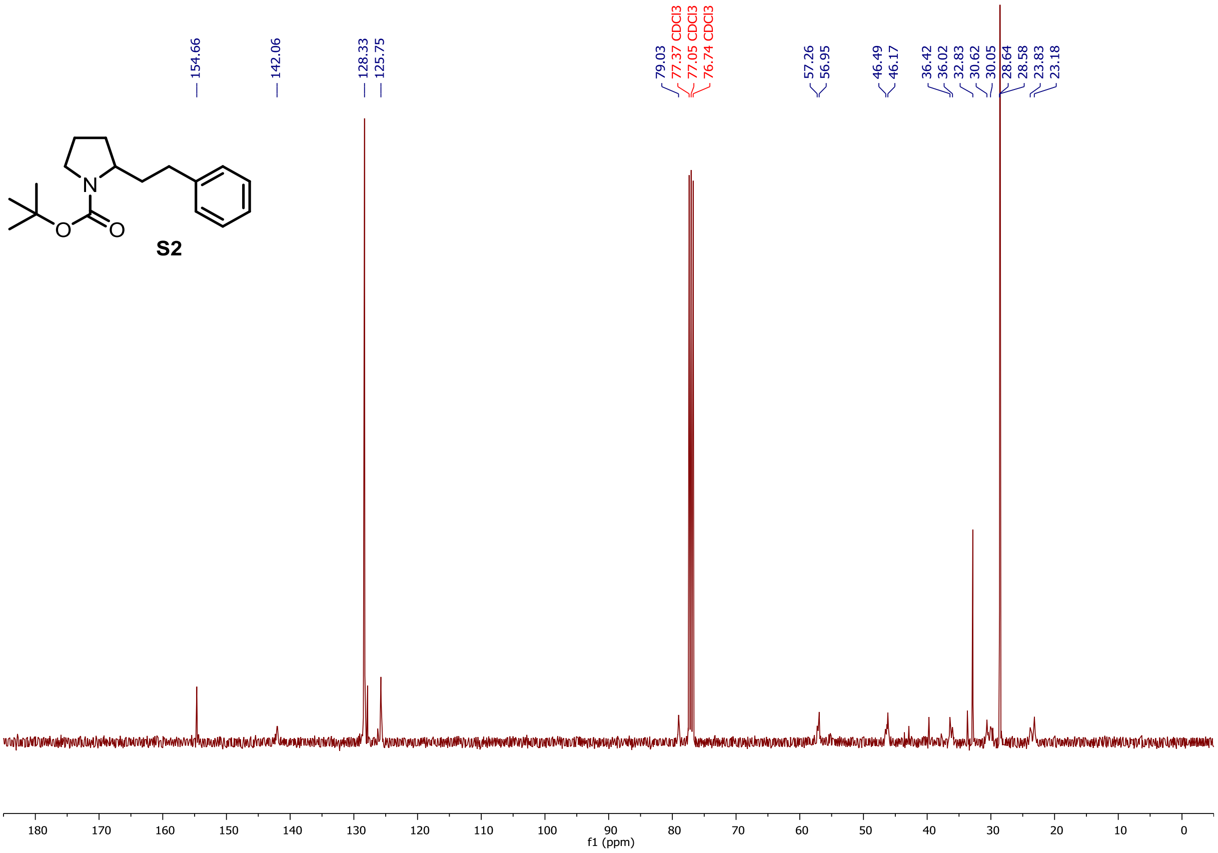

^{1}H NMR (400 MHz, $CDCl_3$)

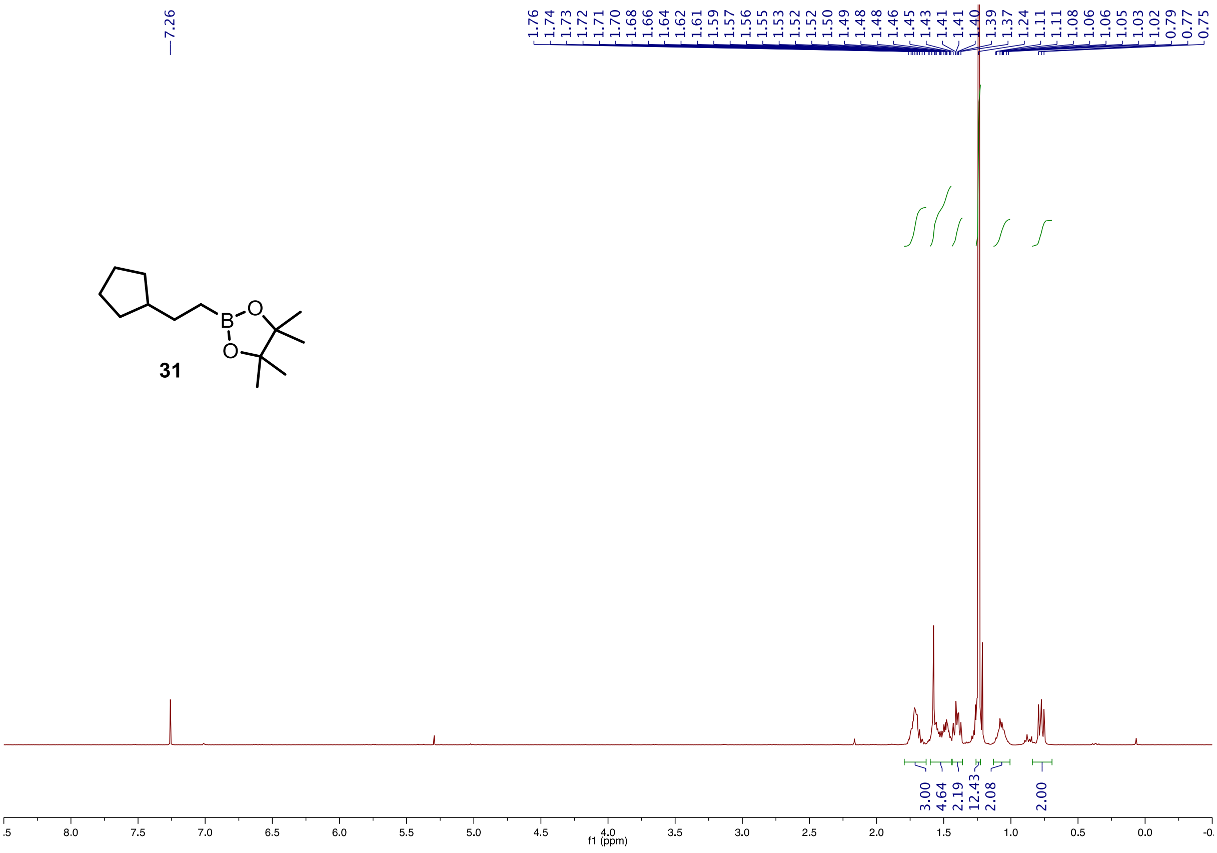

^{13}C NMR (101 MHz, $CDCl_3$)

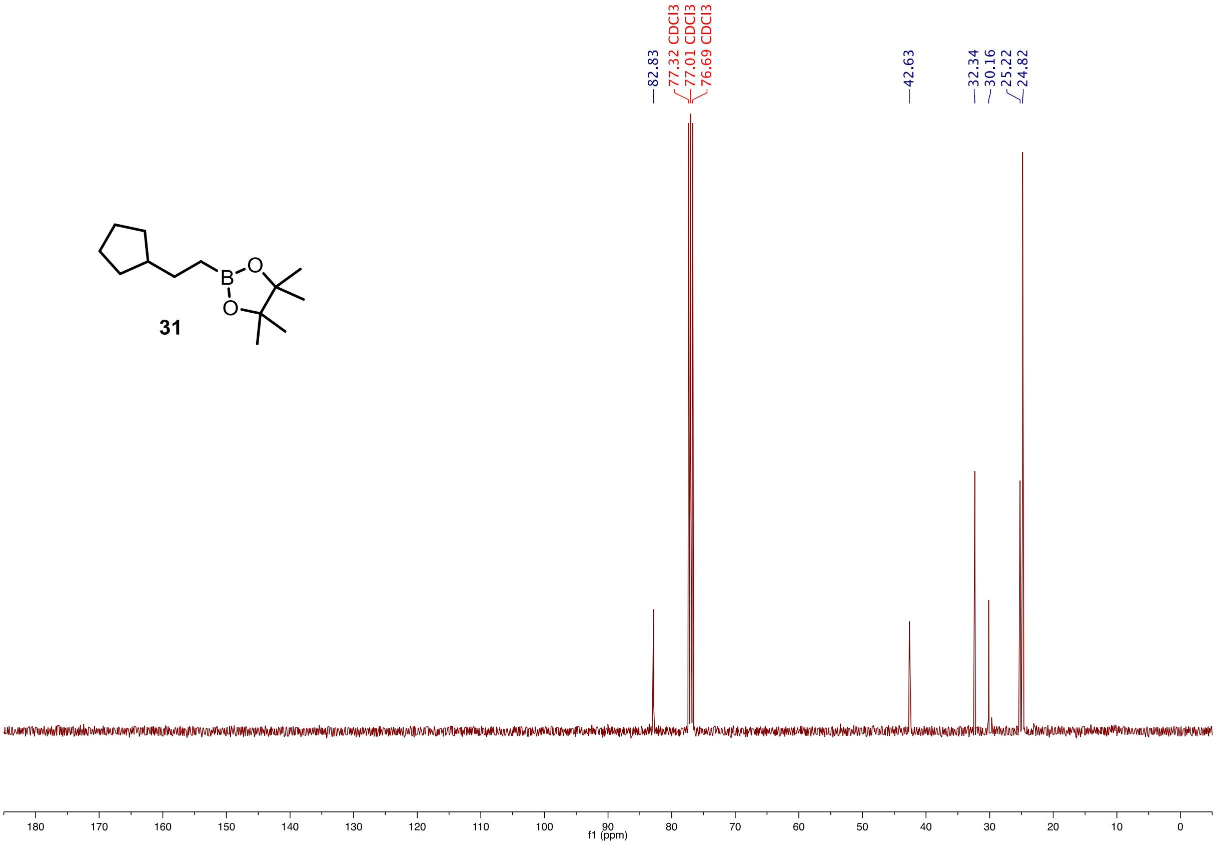

^{1}H NMR (500 MHz, $CDCl_3$)

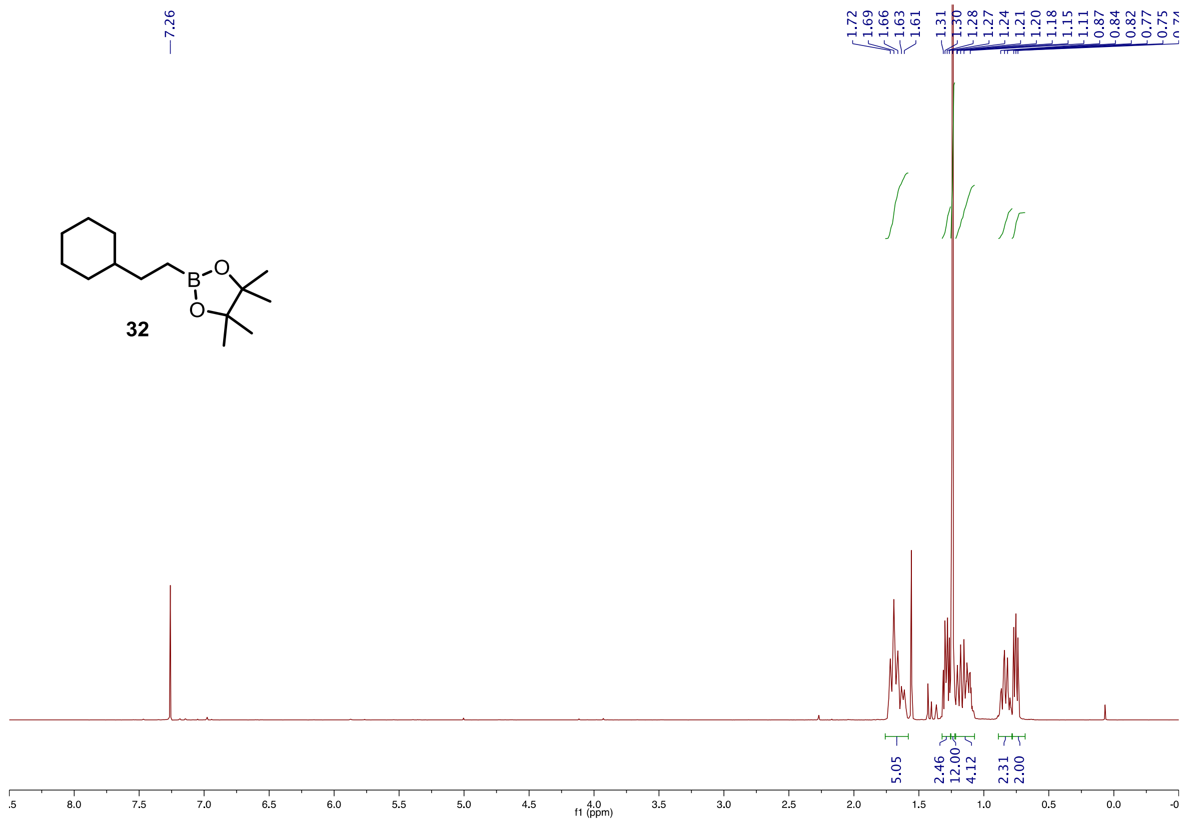

^{13}C NMR (126 MHz, $CDCl_3$)

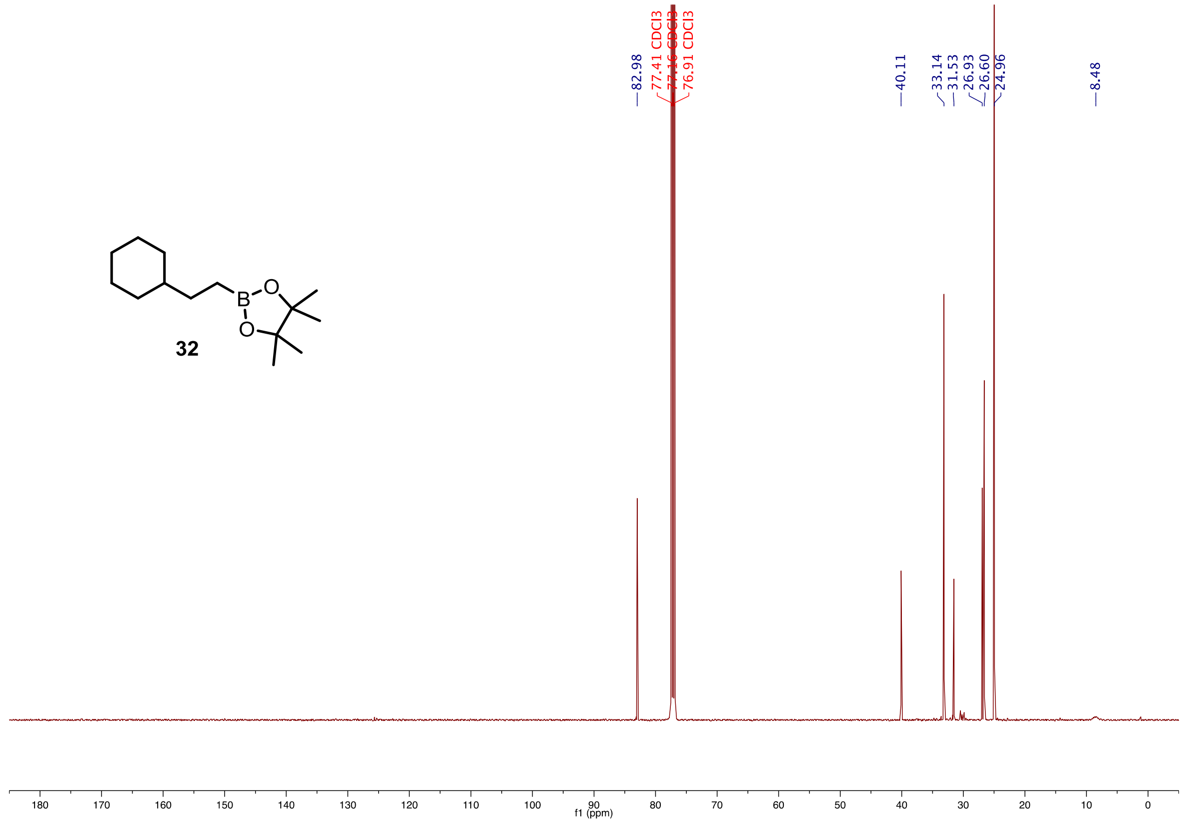

^{1}H NMR (400 MHz, $CDCl_3$)

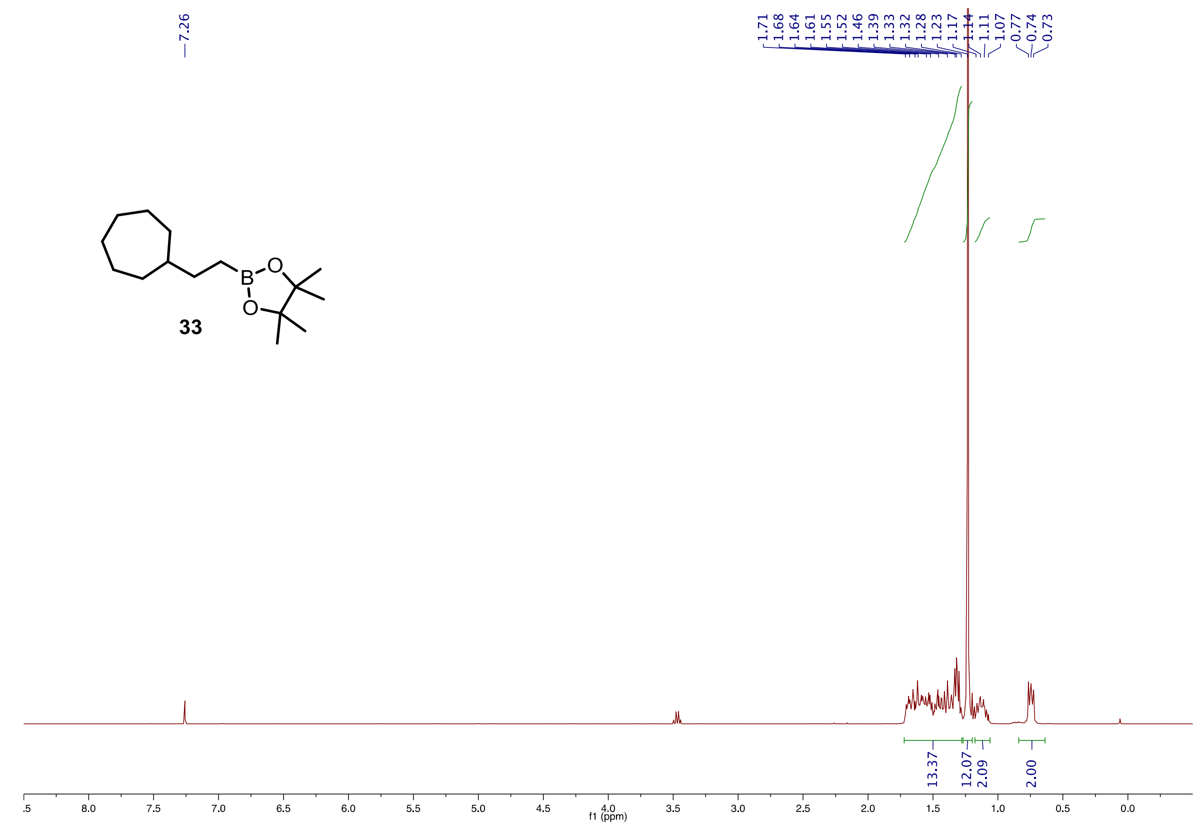

^{13}C NMR (101 MHz, $CDCl_3$)

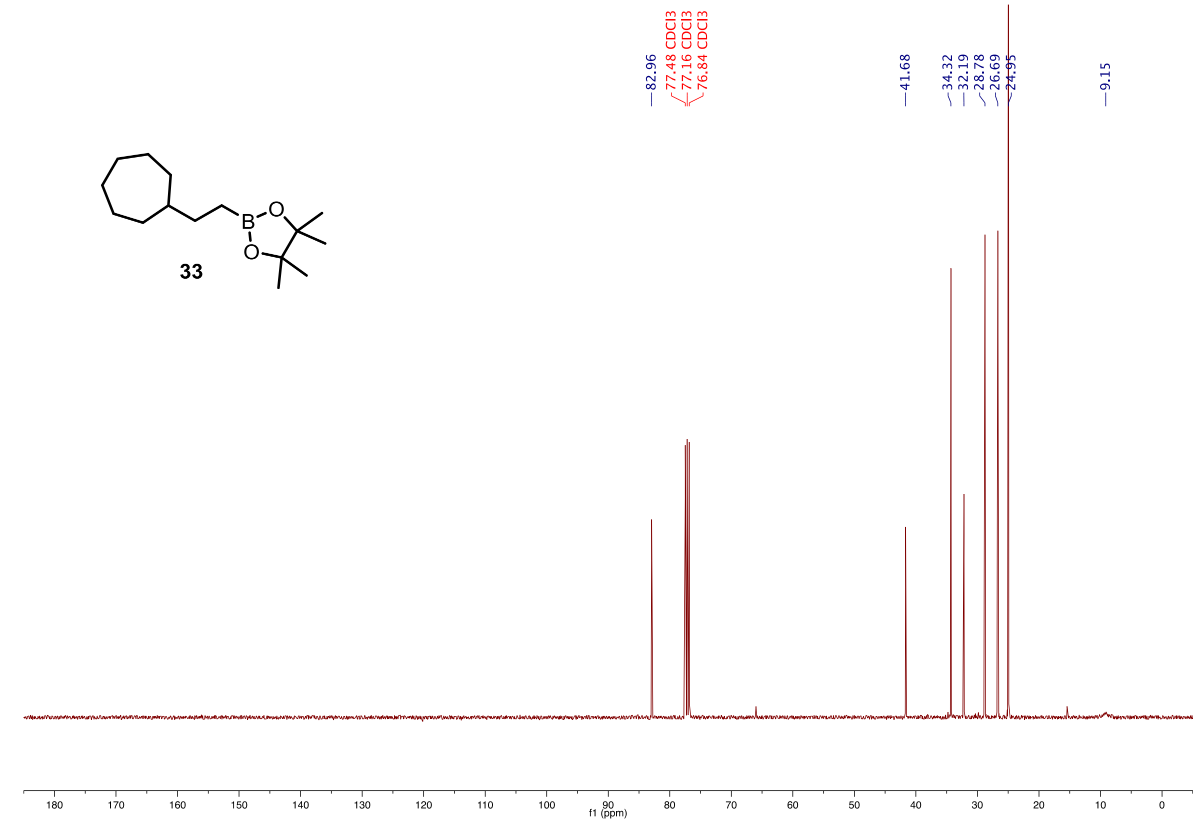

^{1}H NMR (400 MHz, $CDCl_3$)

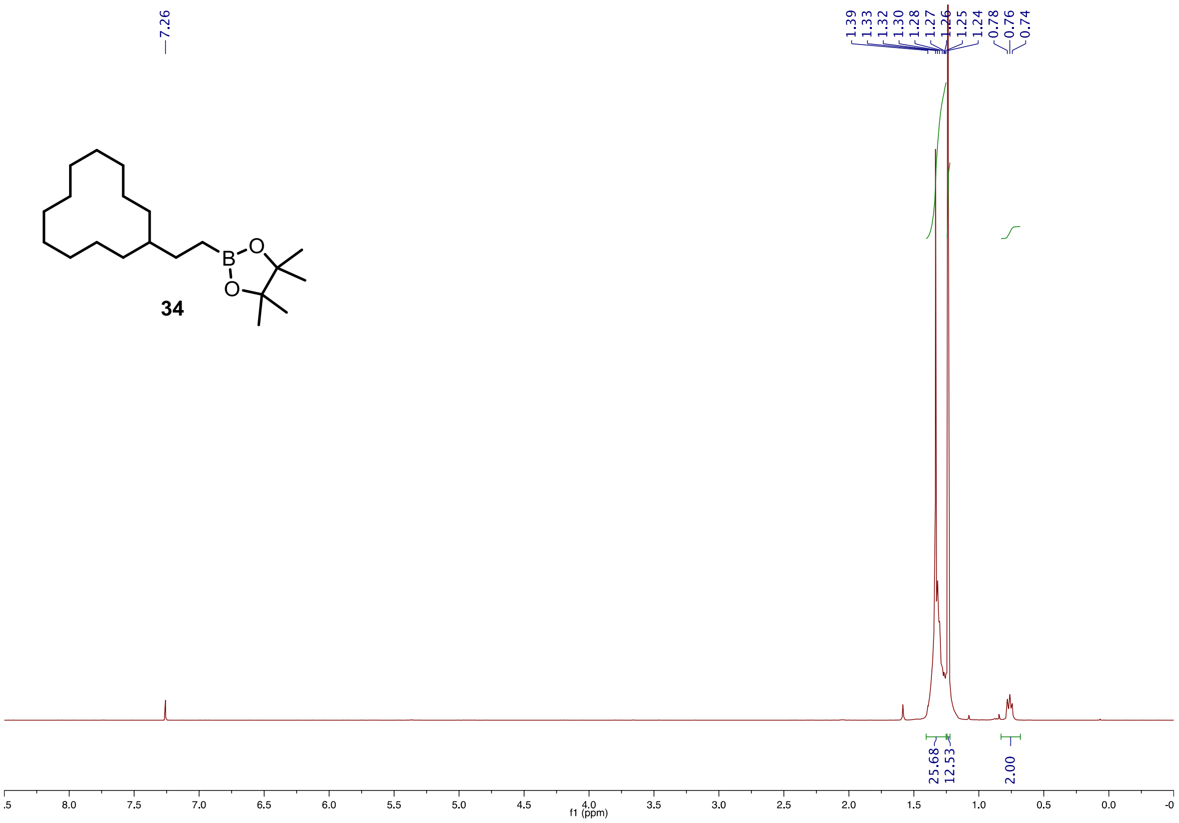

^{13}C NMR (101 MHz, $CDCl_3$)

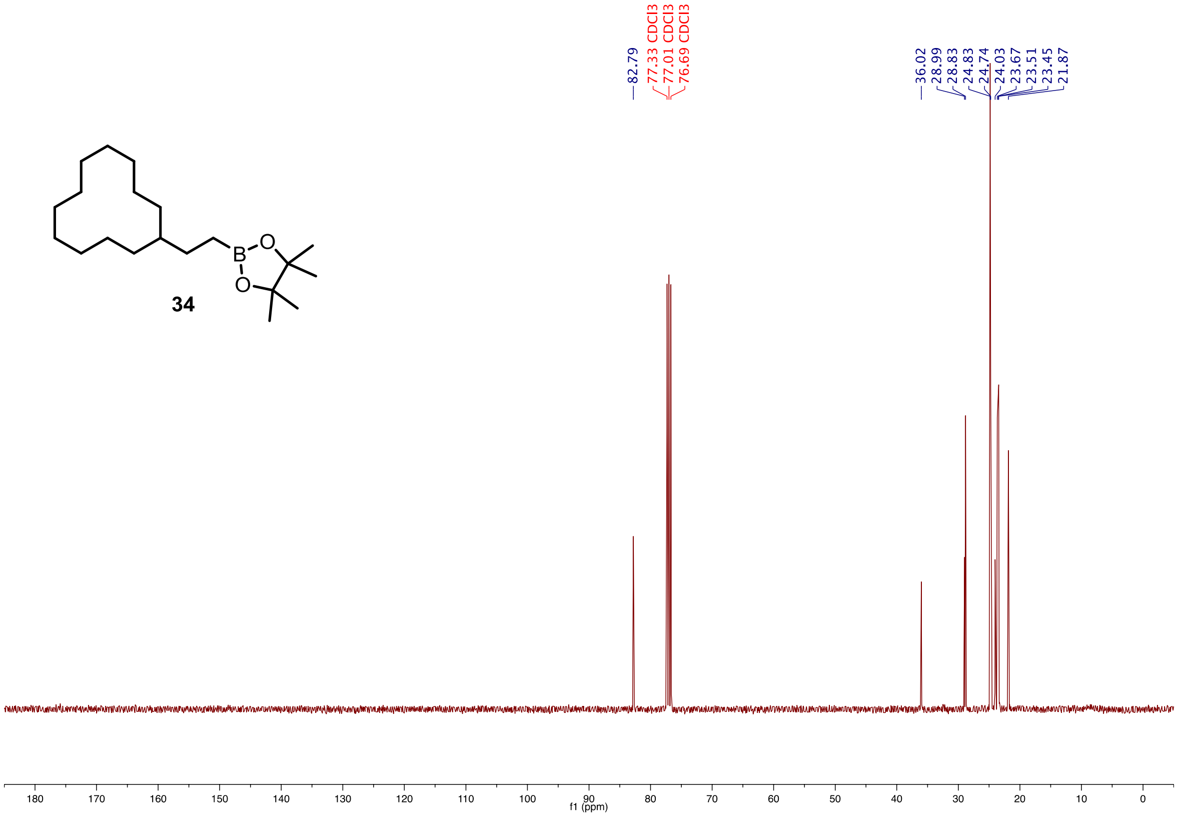

^{1}H NMR (400 MHz, $CDCl_3$)

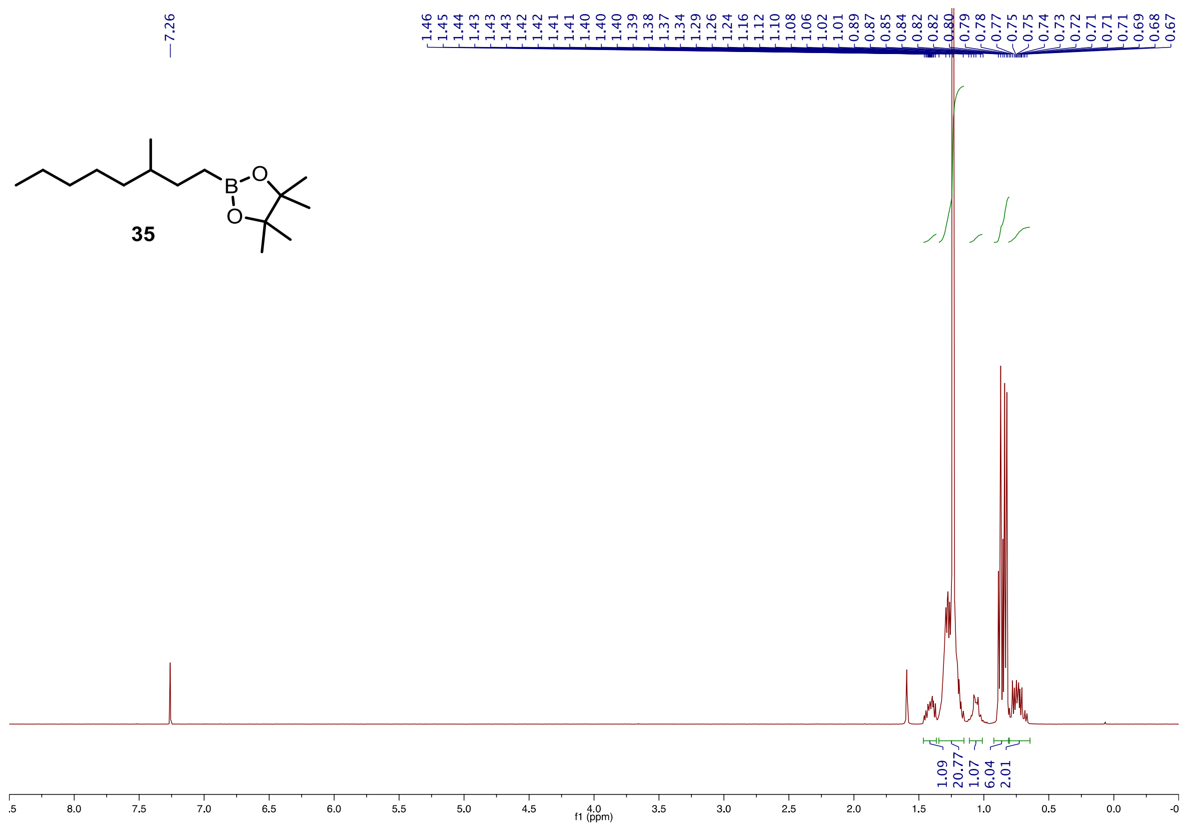

^{13}C NMR (101 MHz, $CDCl_3$)

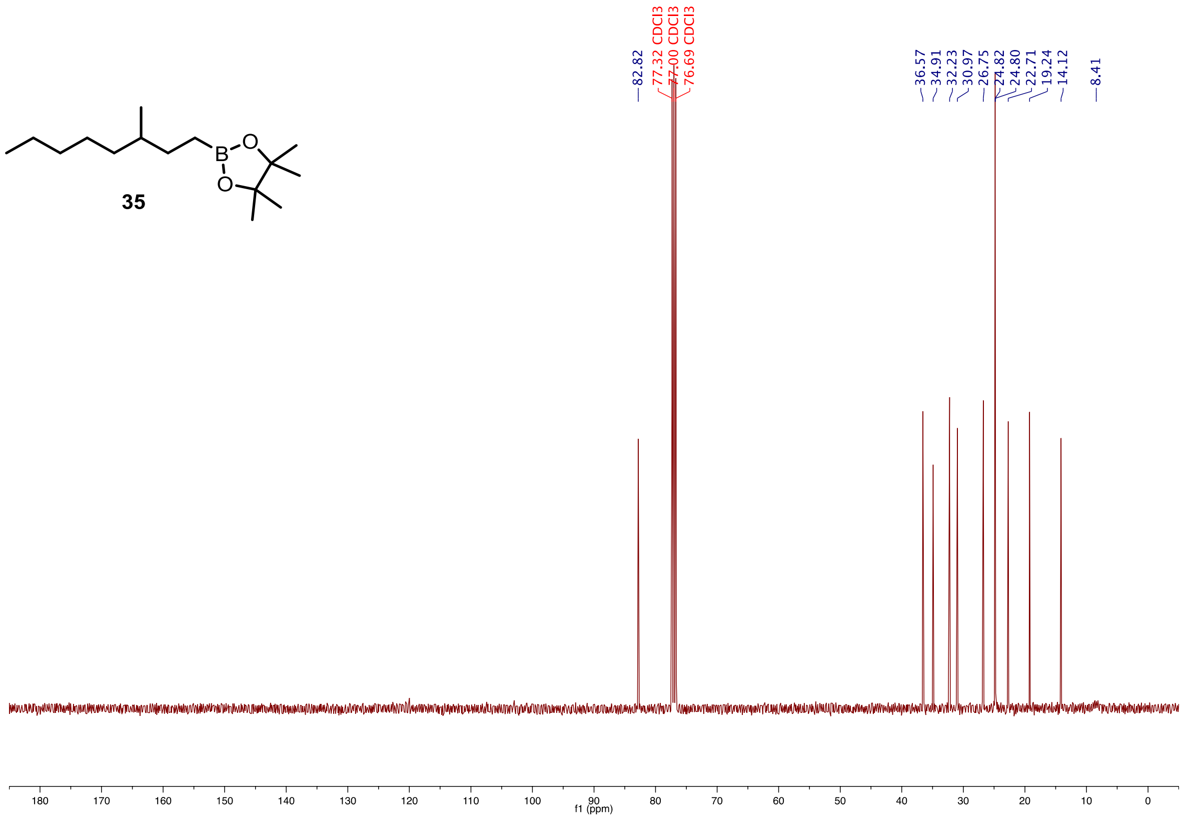

^{1}H NMR (400 MHz, $CDCl_3$)

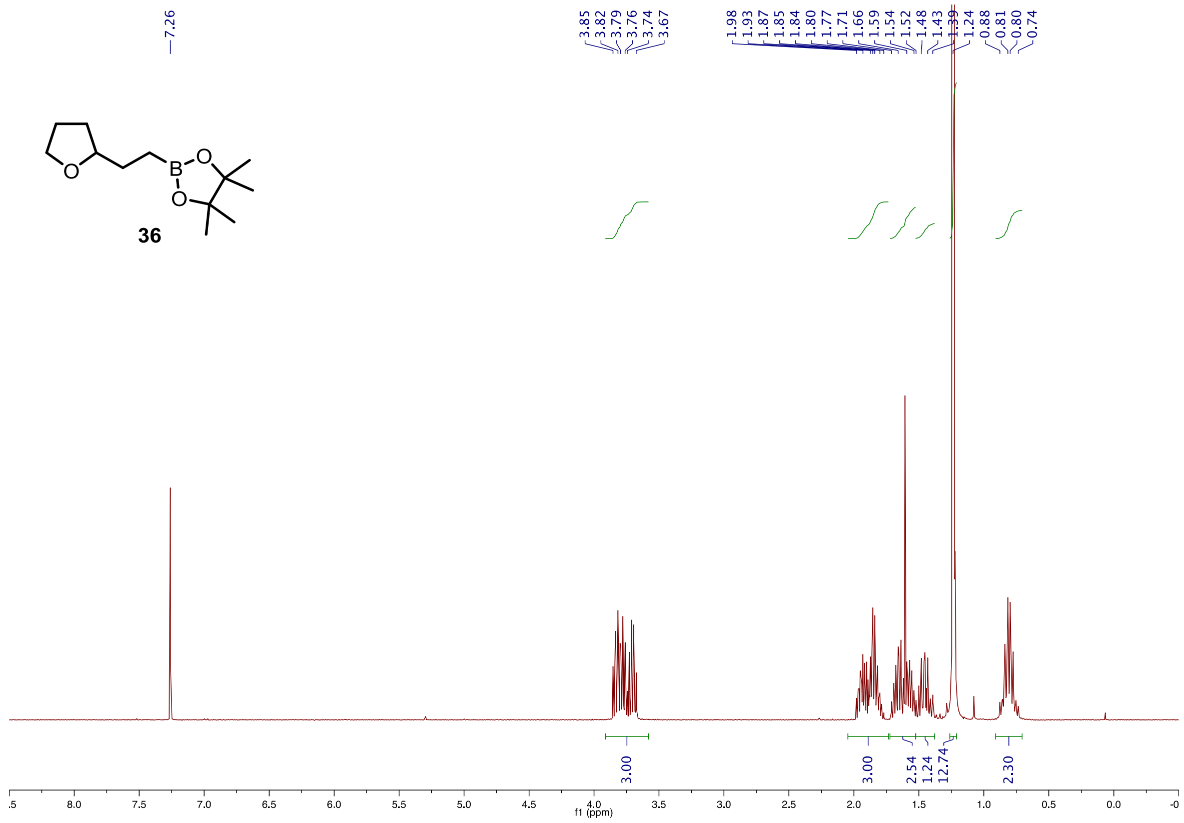

^{13}C NMR (101 MHz, $CDCl_3$)

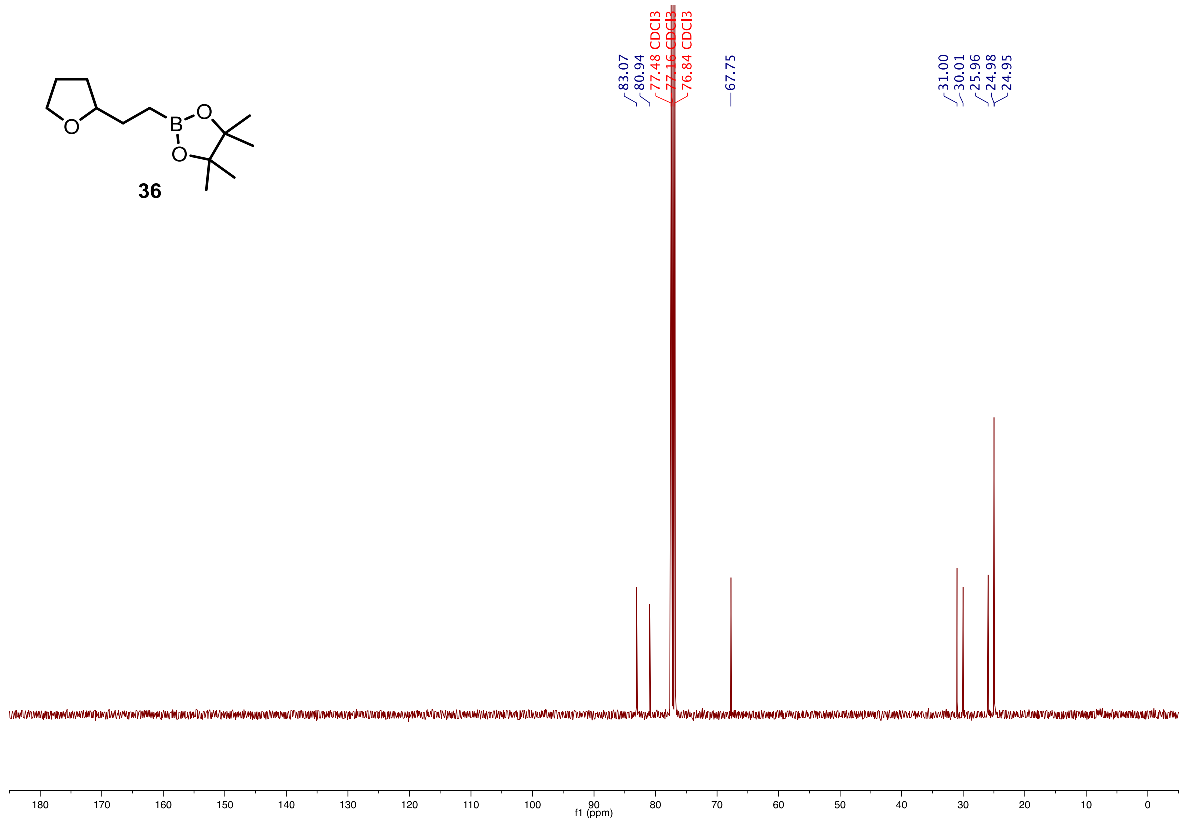

^{1}H NMR (400 MHz, $CDCl_3$)

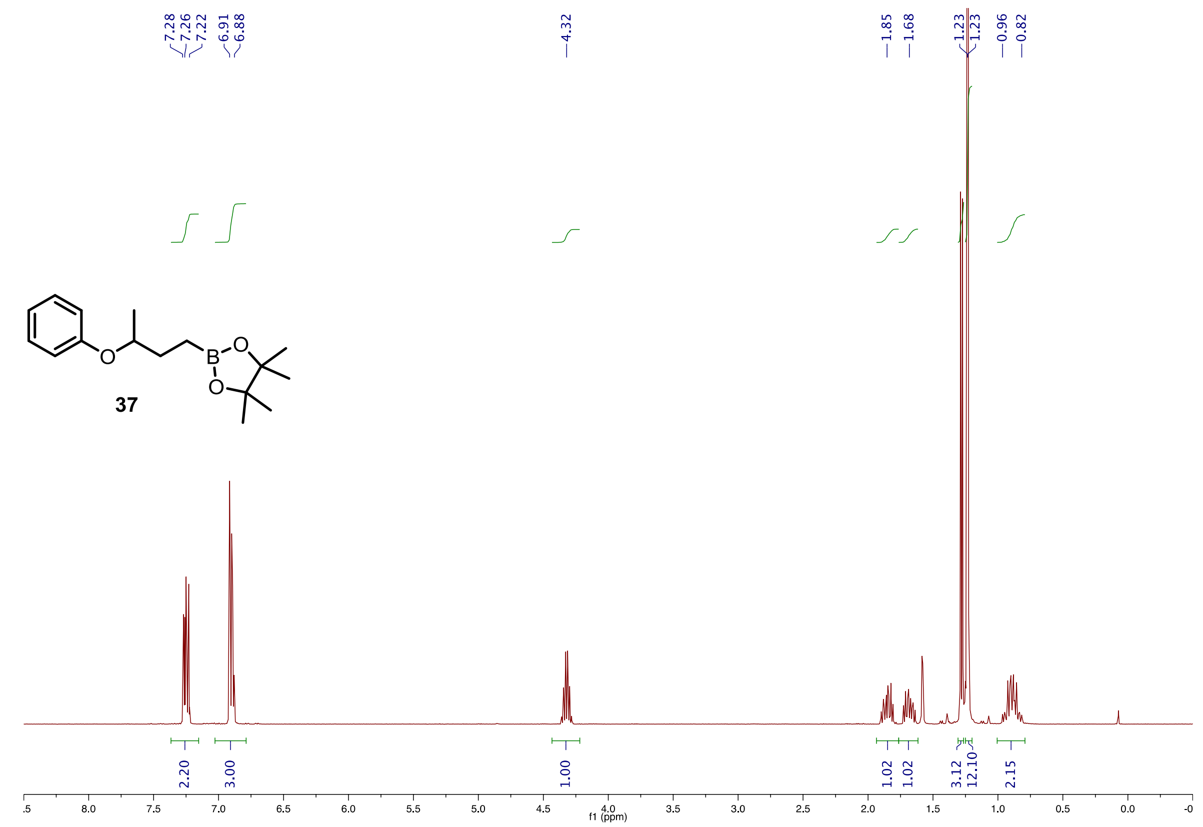

^{13}C NMR (101 MHz, $CDCl_3$)

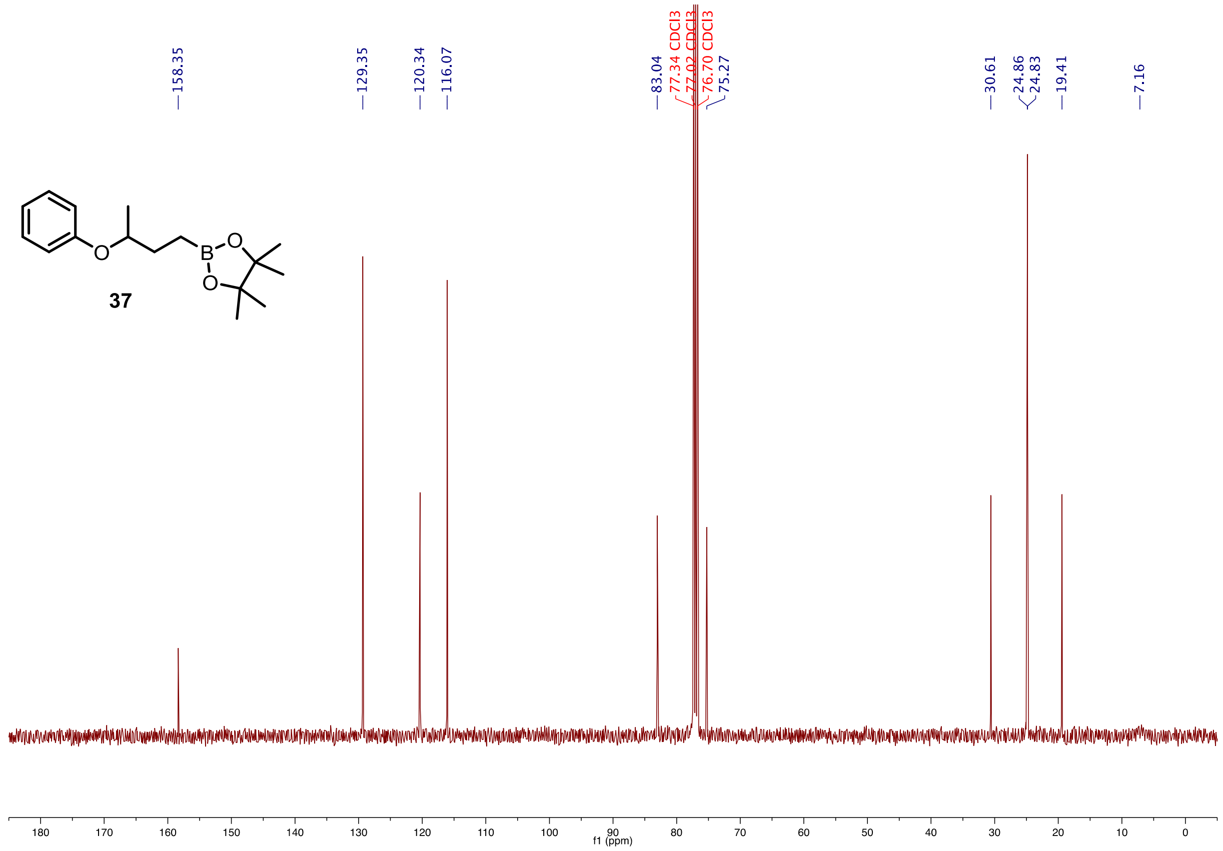

^{1}H NMR (400 MHz, $CDCl_3$)

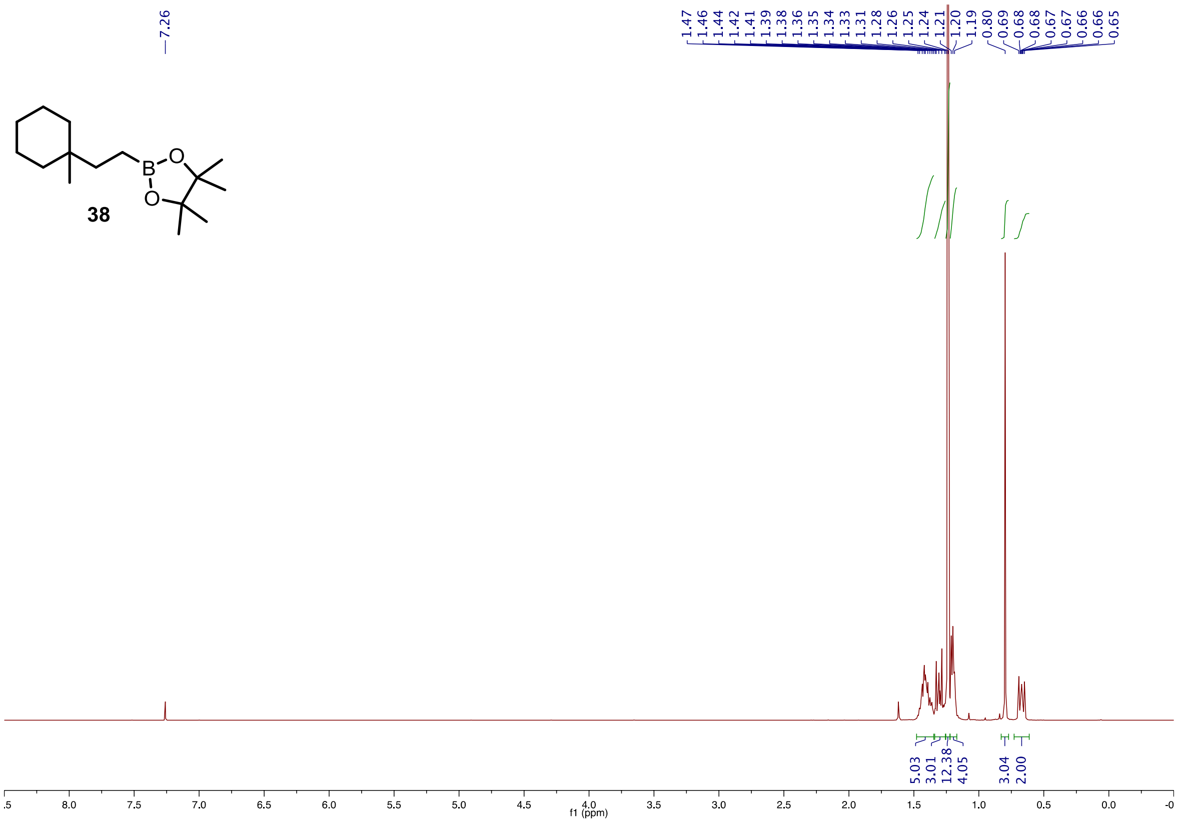

^{13}C NMR (101 MHz, $CDCl_3$)

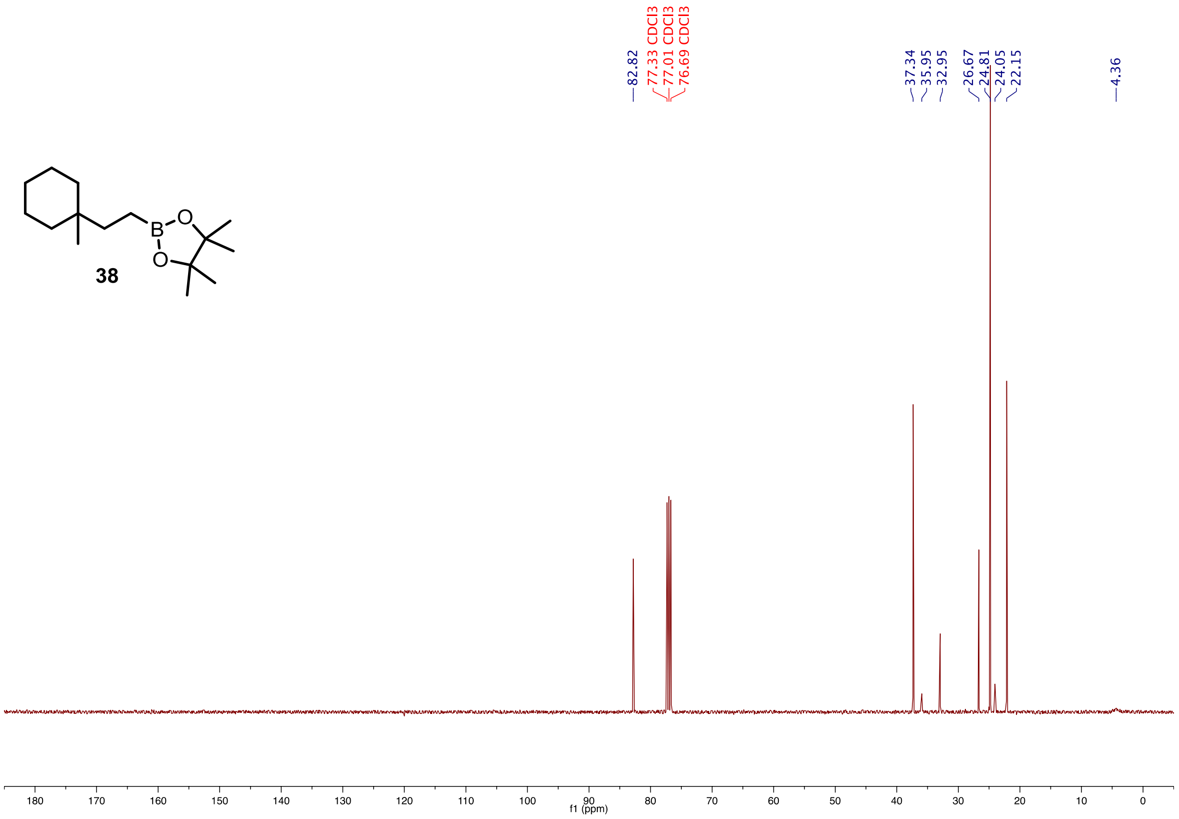

^{1}H NMR (400 MHz, $CDCl_3$)

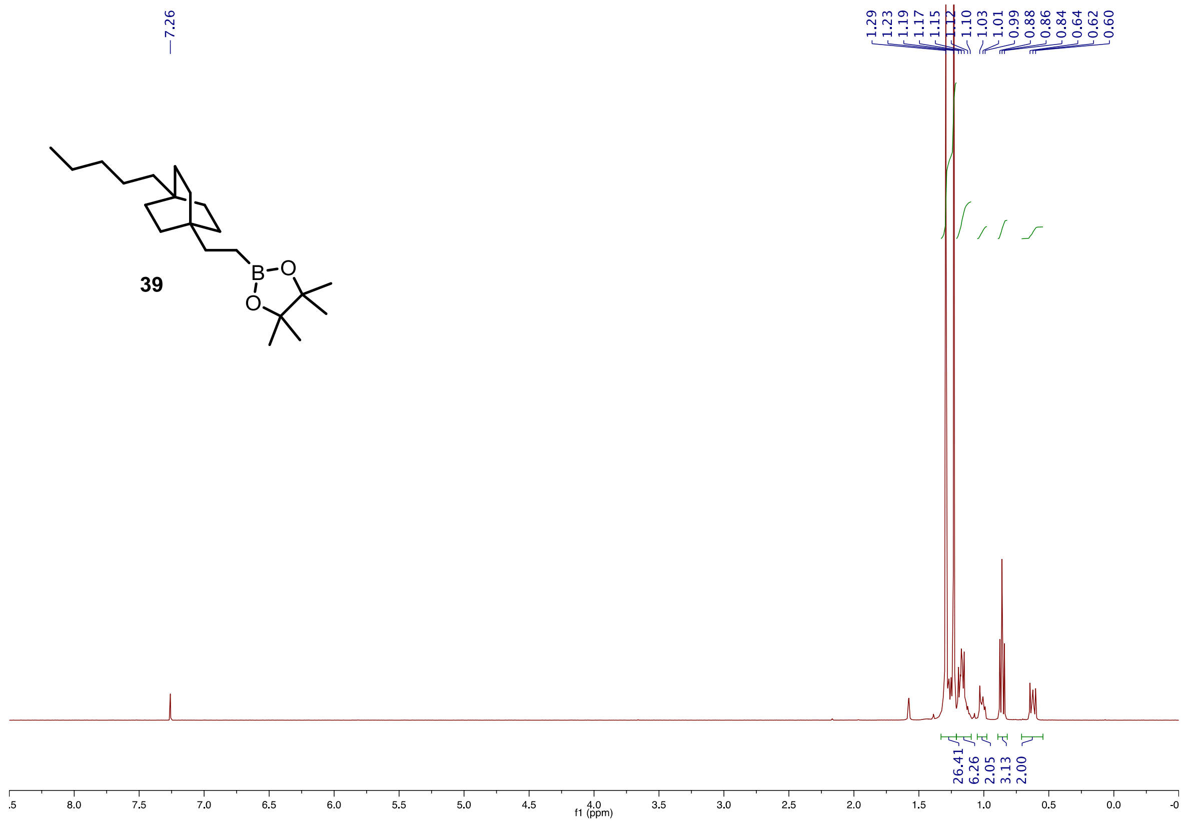

^{13}C NMR (101 MHz, $CDCl_3$)

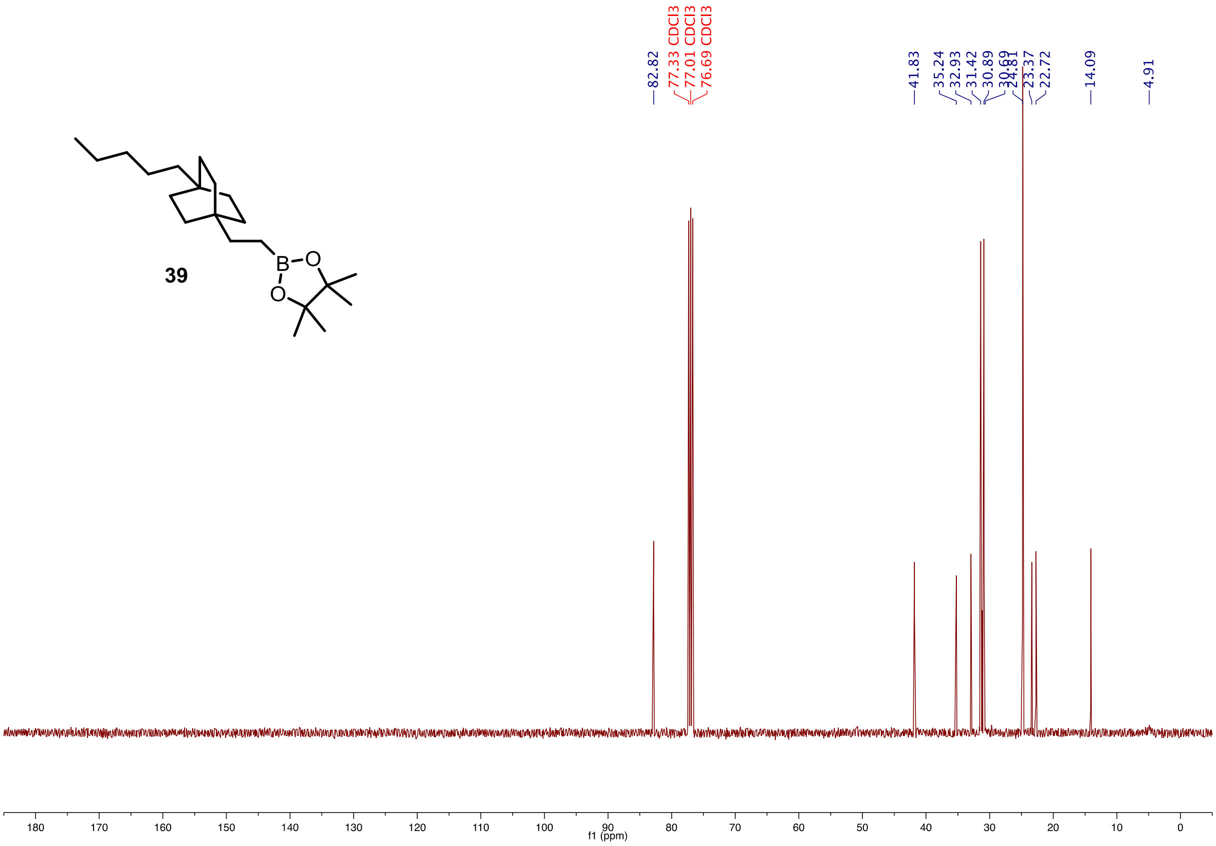

^{1}H NMR (400 MHz, $CDCl_3$)

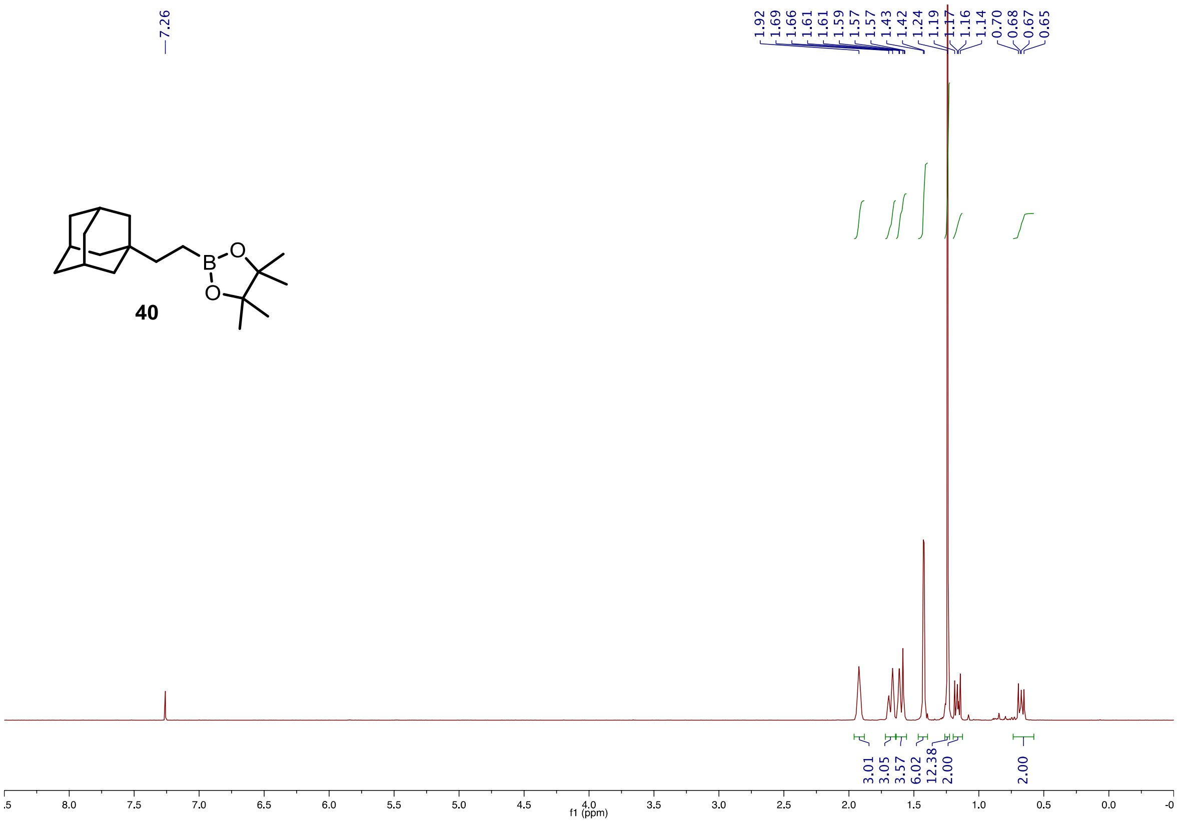

^{13}C NMR (101 MHz, $CDCl_3$)

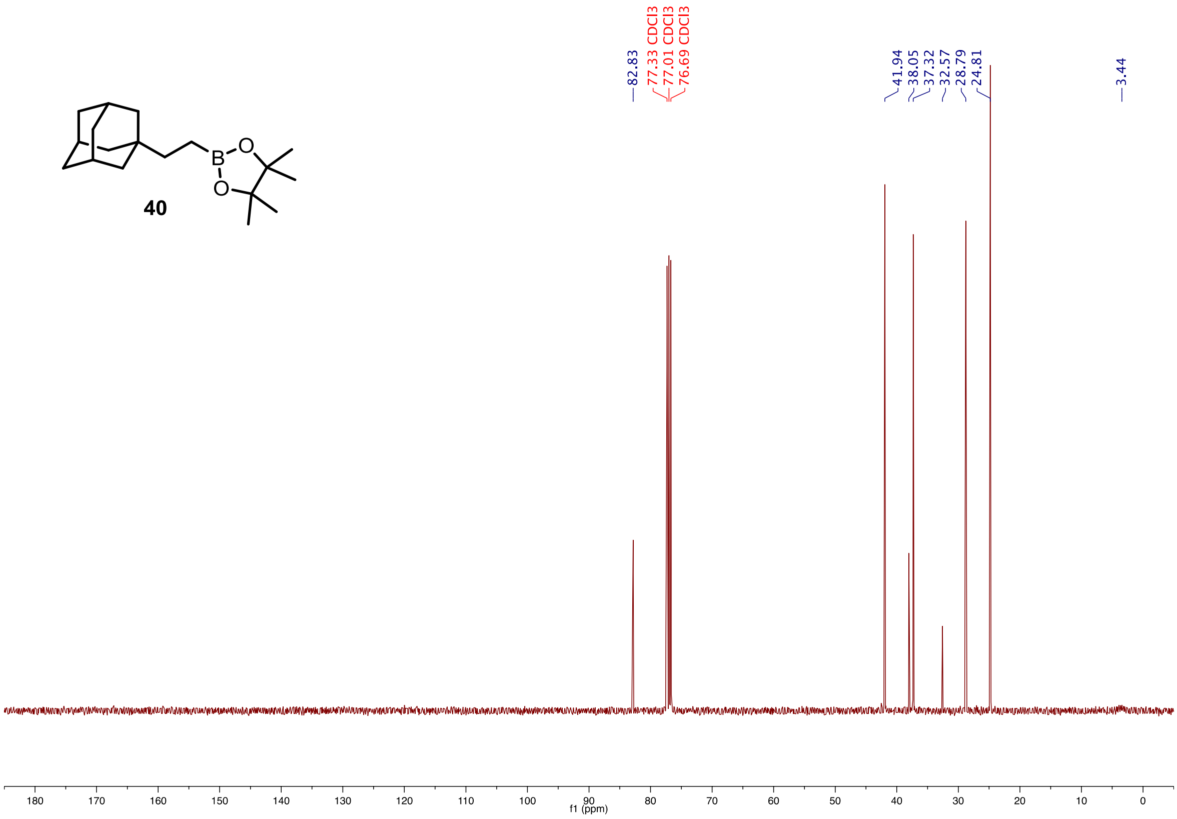

^{1}H NMR (400 MHz, $CDCl_3$)

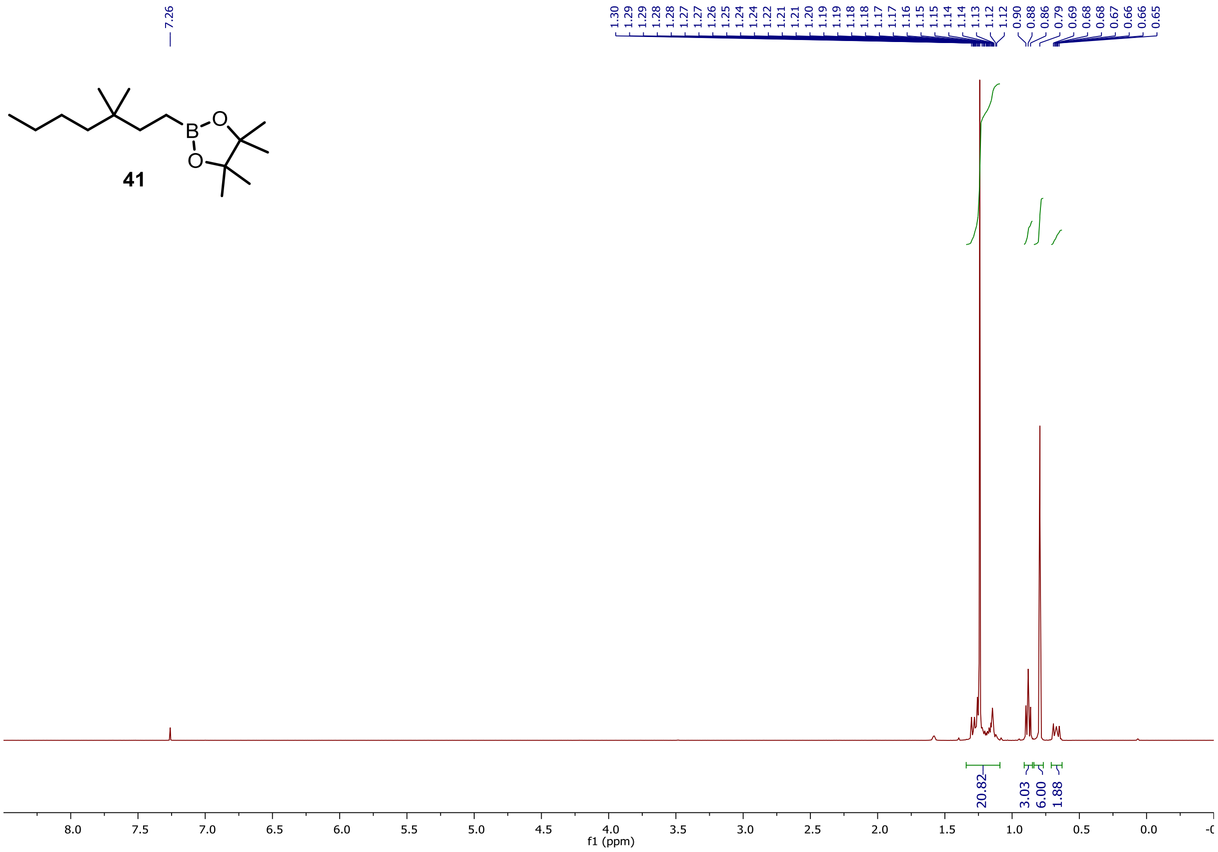

^{13}C NMR (101 MHz, $CDCl_3$)

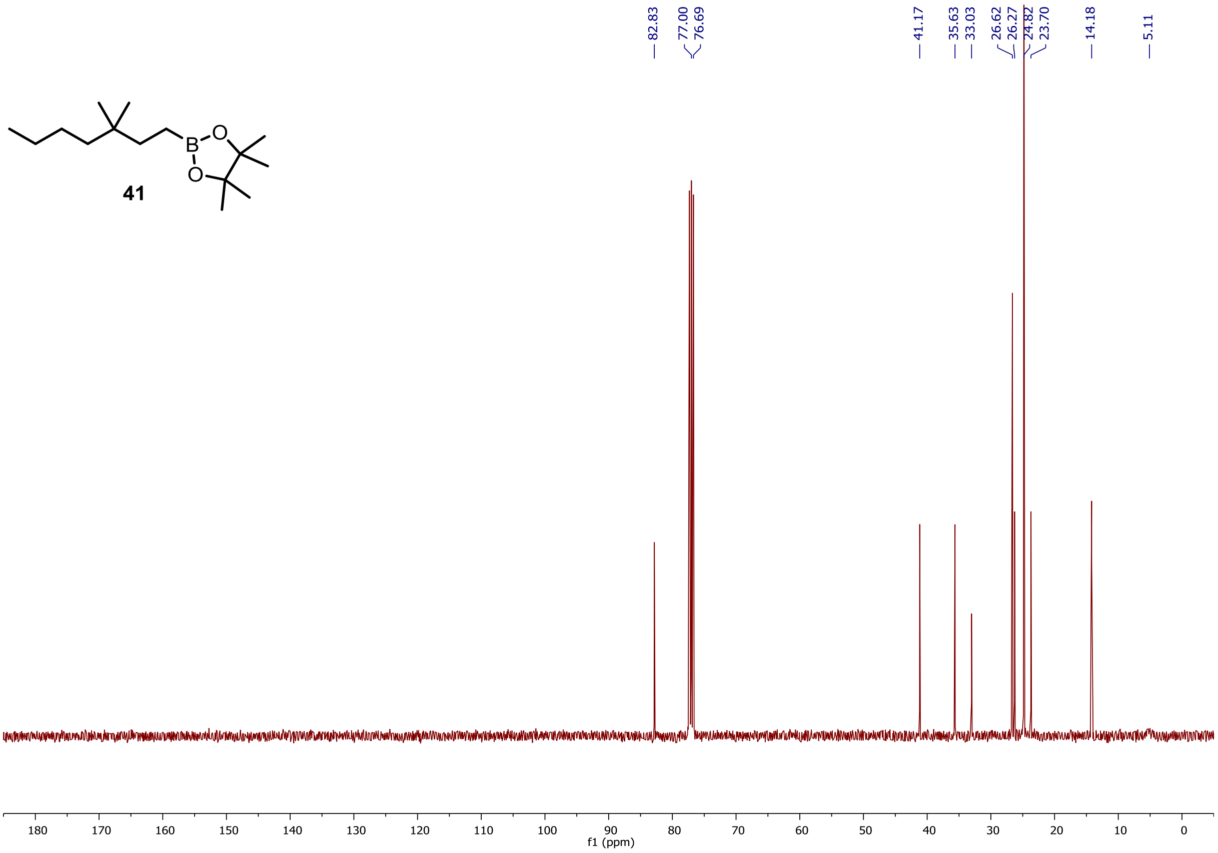

^{1}H NMR (500 MHz, $CDCl_3$)

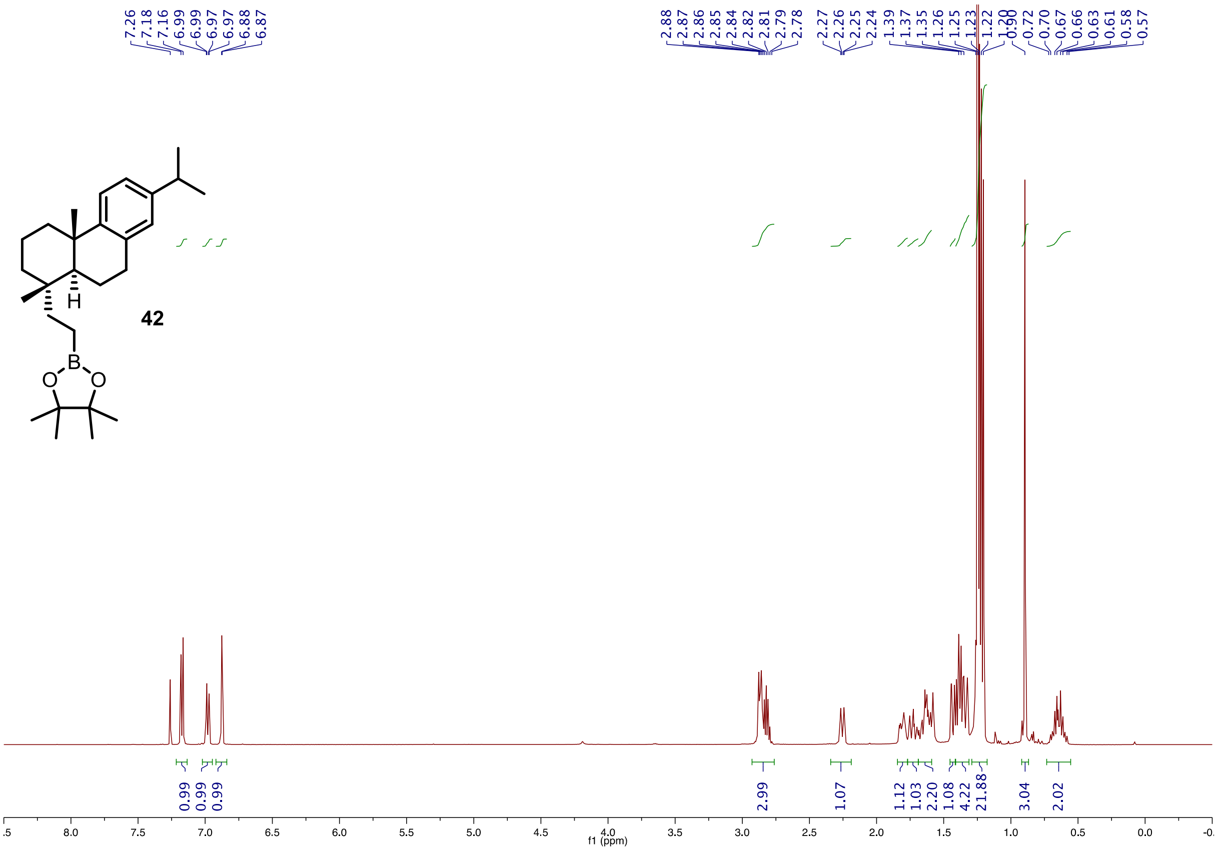

^{13}C NMR (126 MHz, $CDCl_3$)

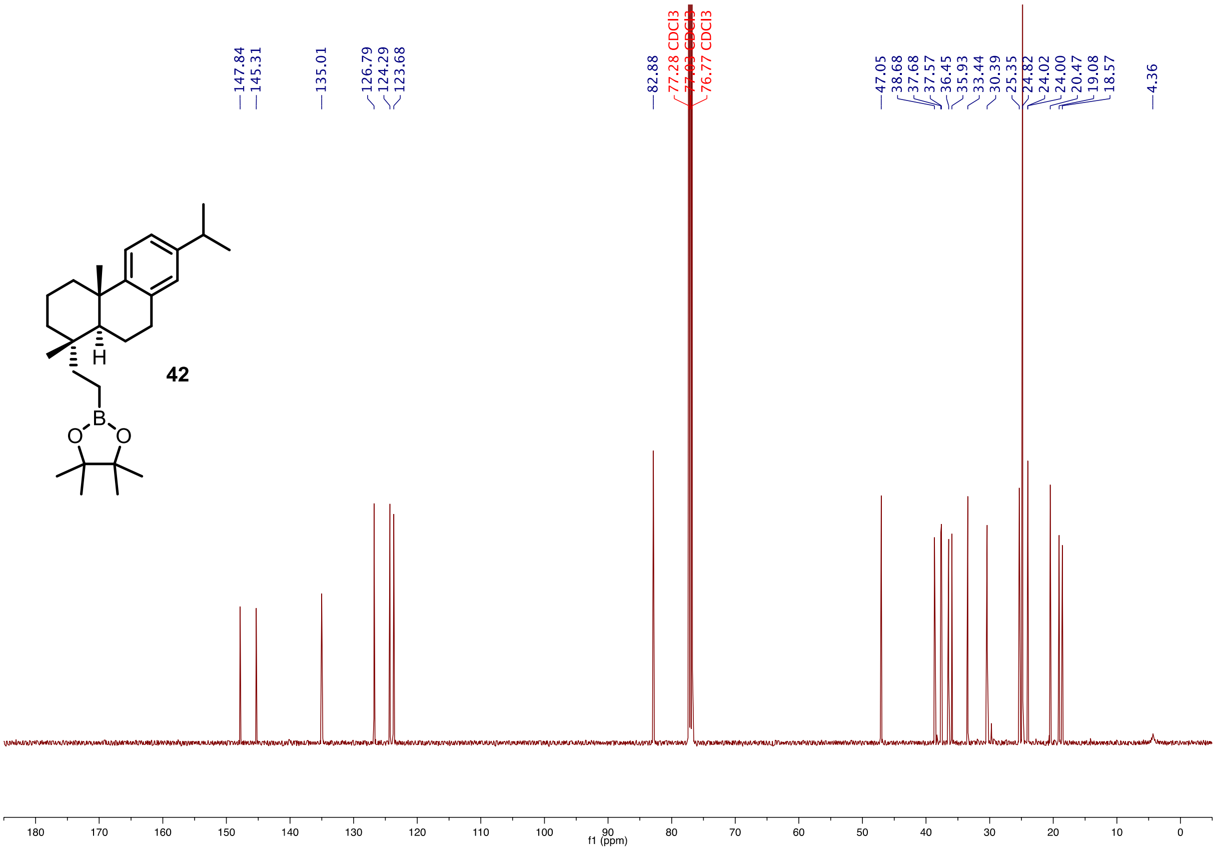

^{1}H NMR (500 MHz, $CDCl_3$)

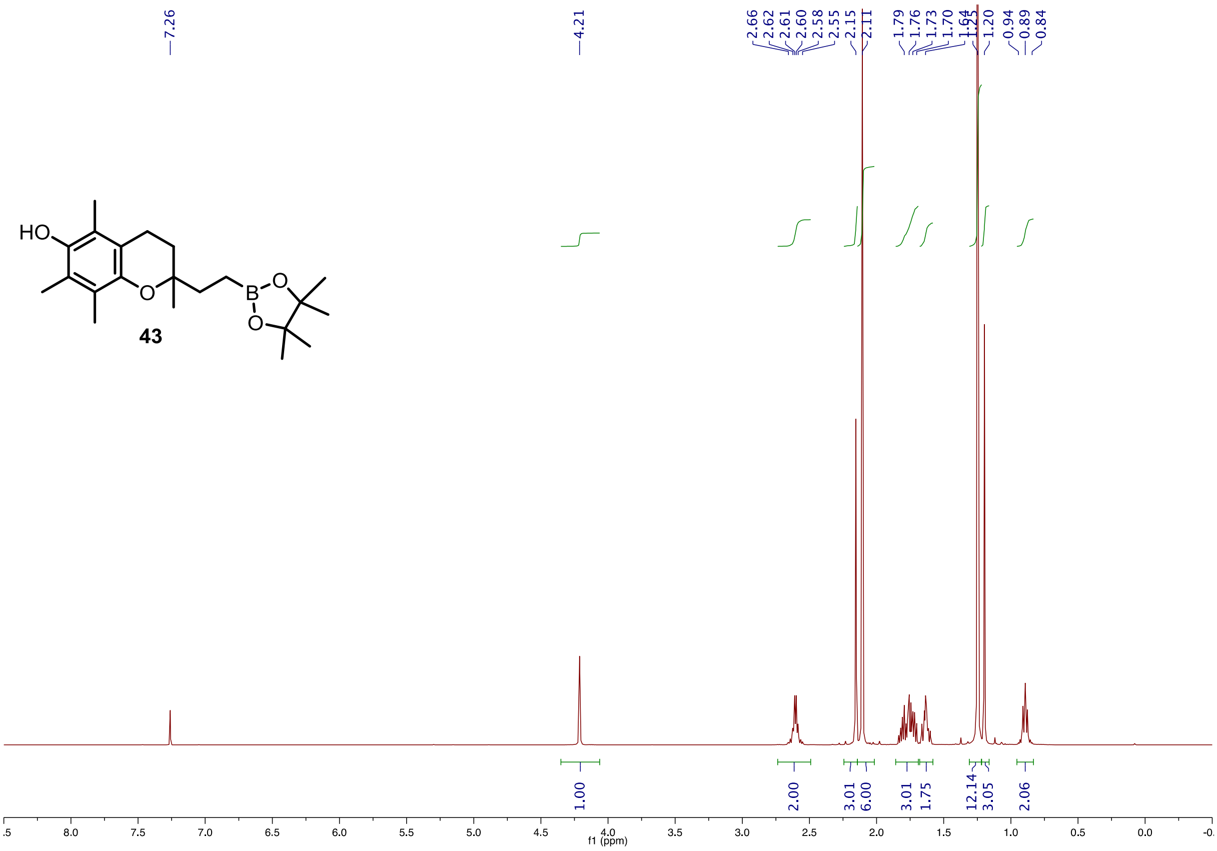

^{13}C NMR (126 MHz, $CDCl_3$)

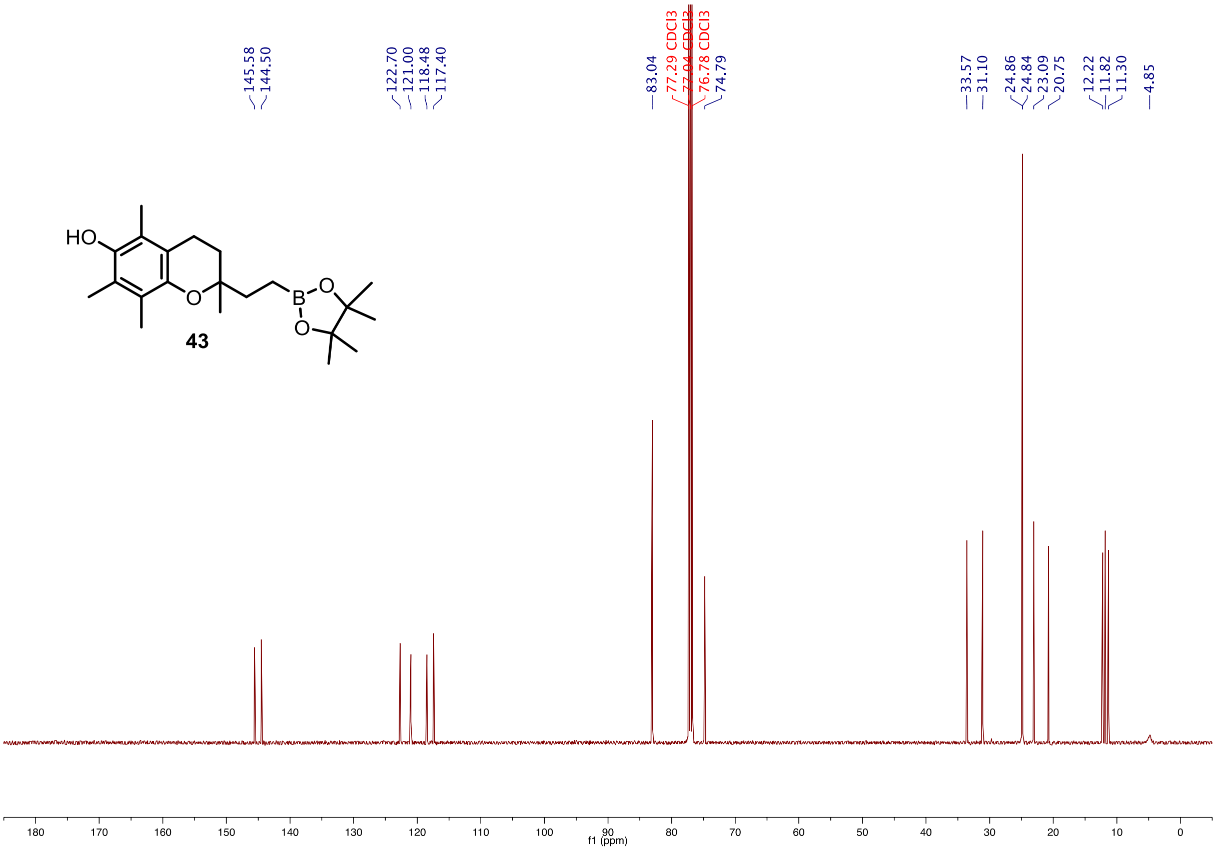

^{1}H NMR (500 MHz, $CDCl_3$)

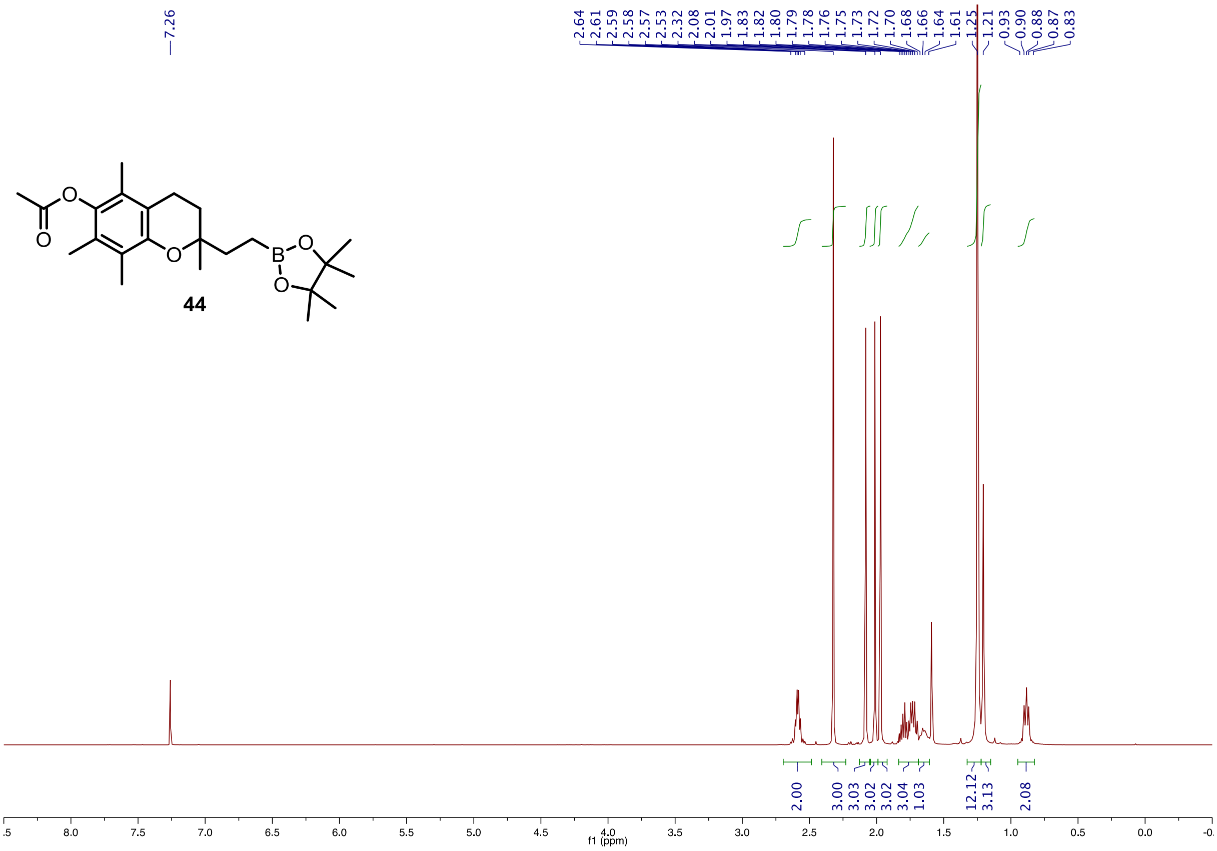

^{13}C NMR (126 MHz, $CDCl_3$)

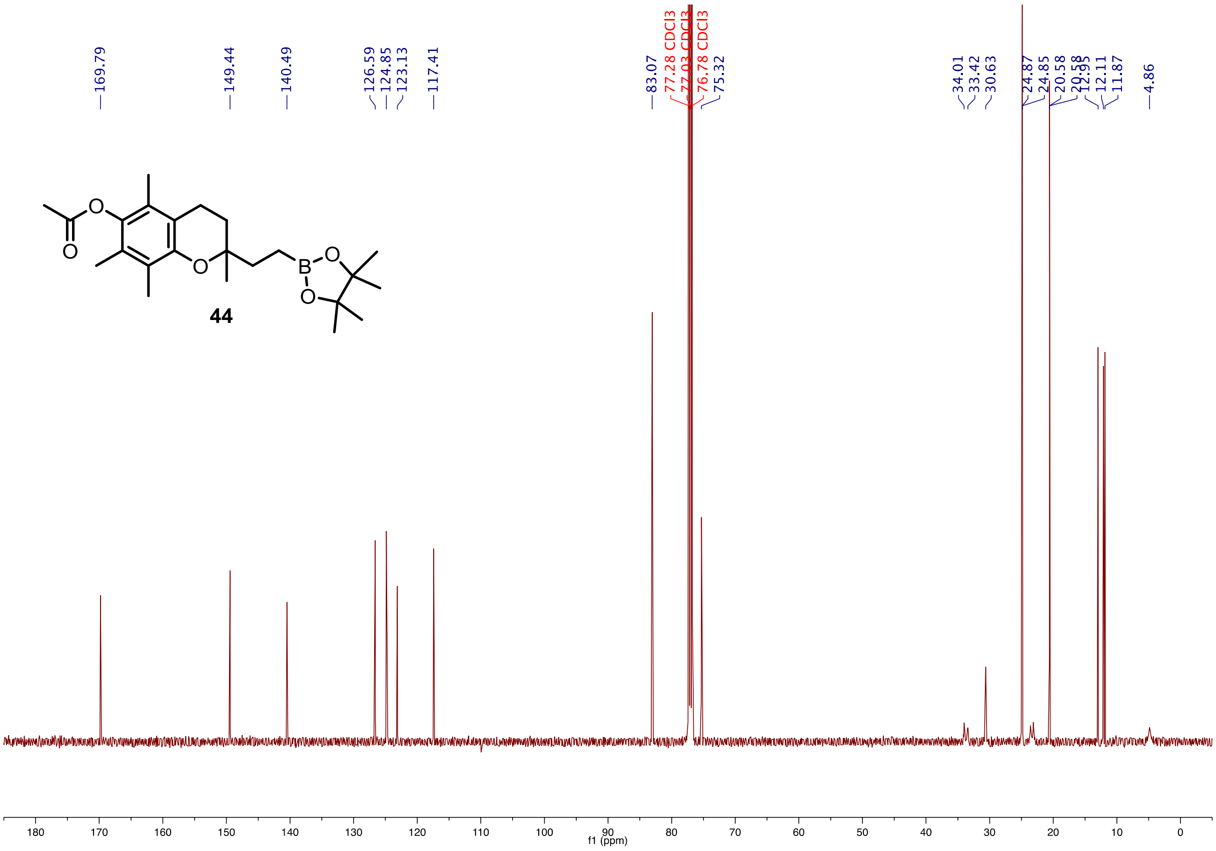

^{1}H NMR (400 MHz, $CDCl_3$)

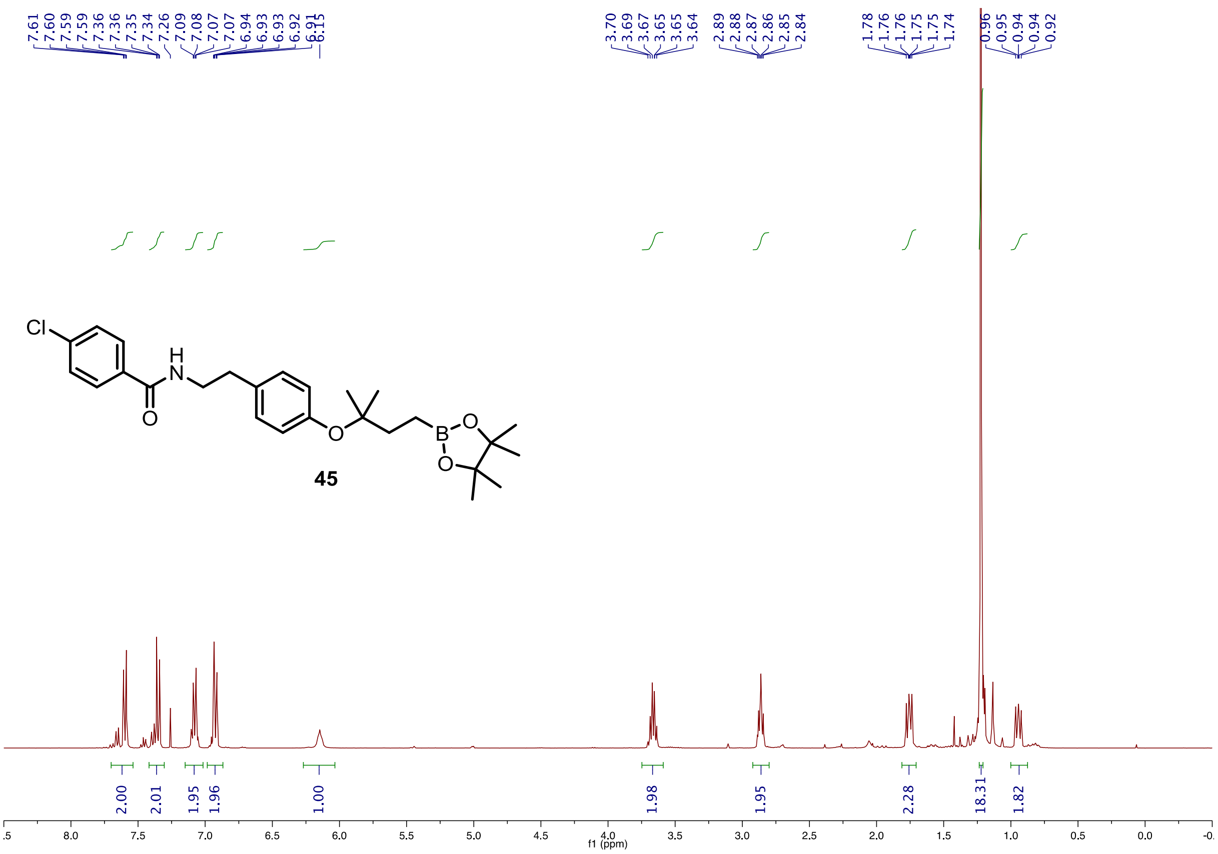

^{13}C NMR (101 MHz, $CDCl_3$)

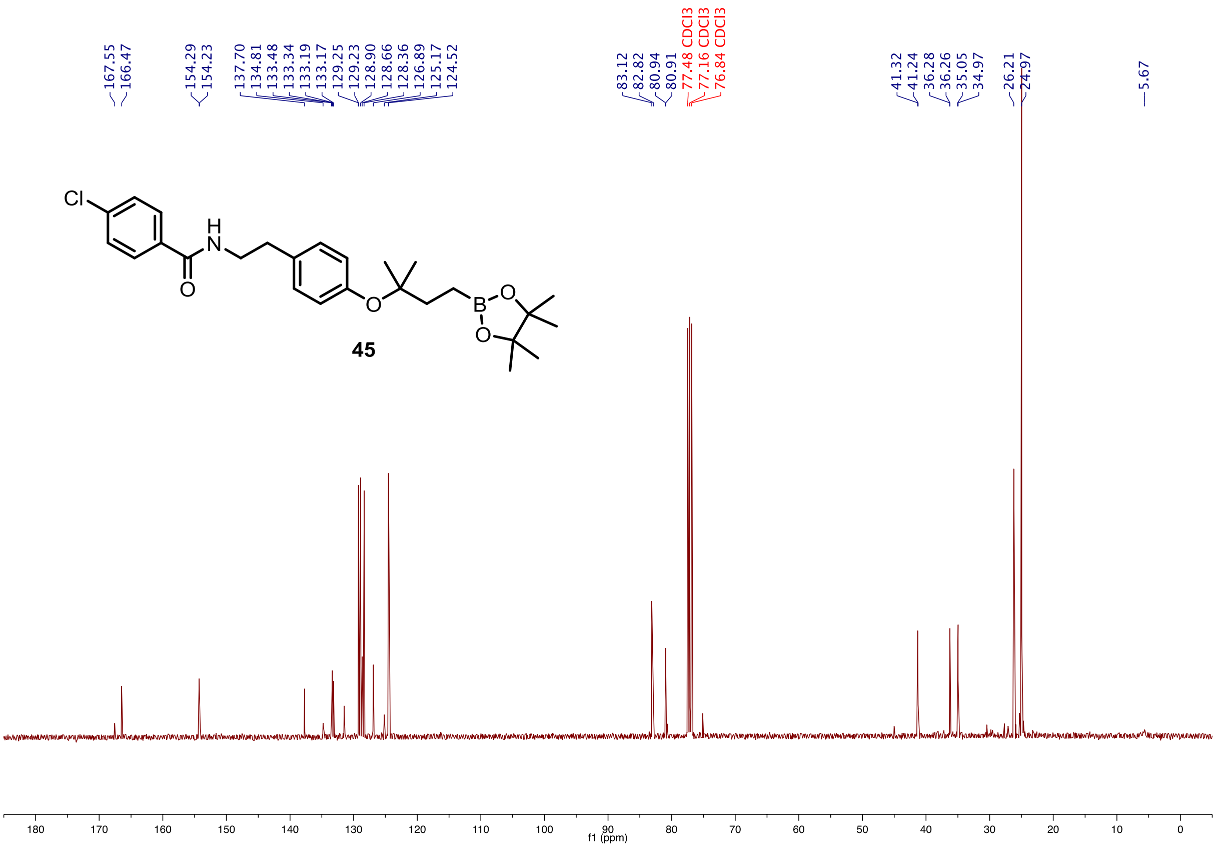

^{1}H NMR (500 MHz, $CDCl_3$)

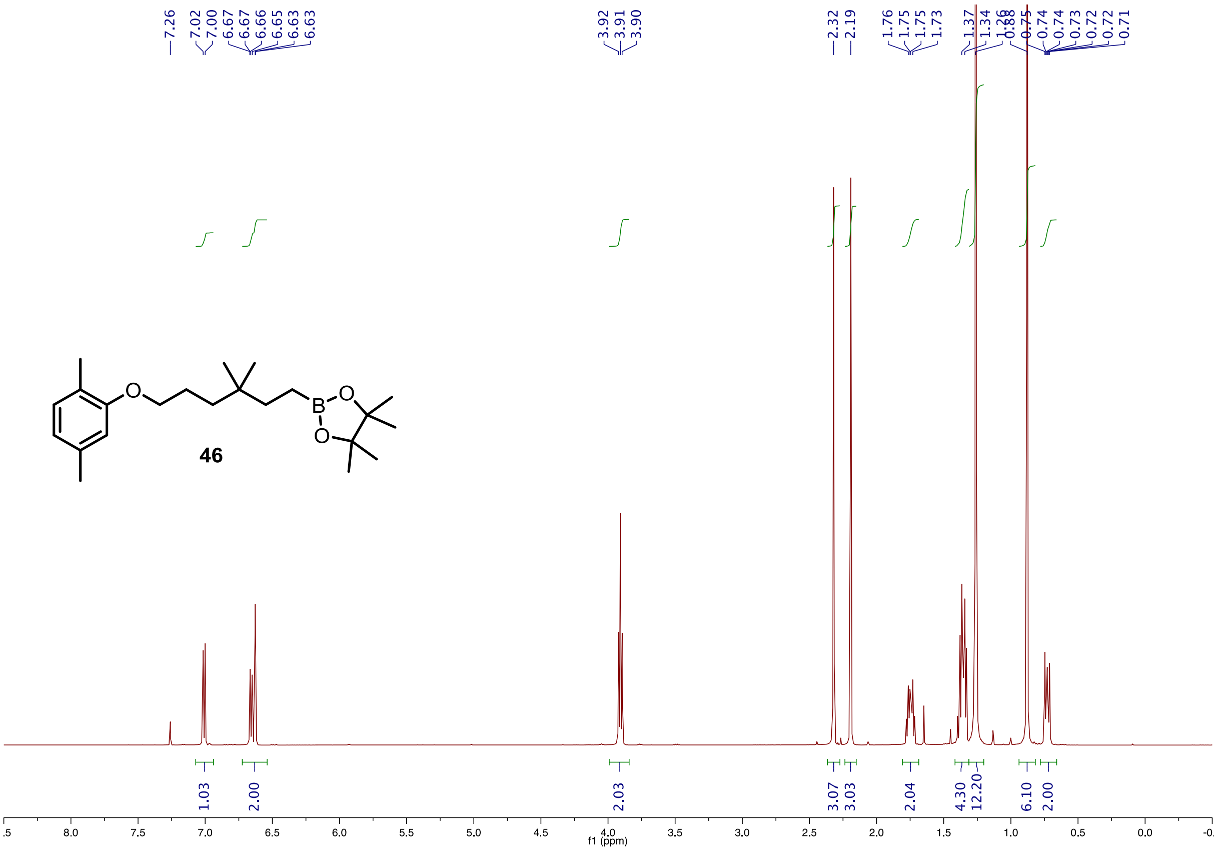

^{13}C NMR (126 MHz, $CDCl_3$)

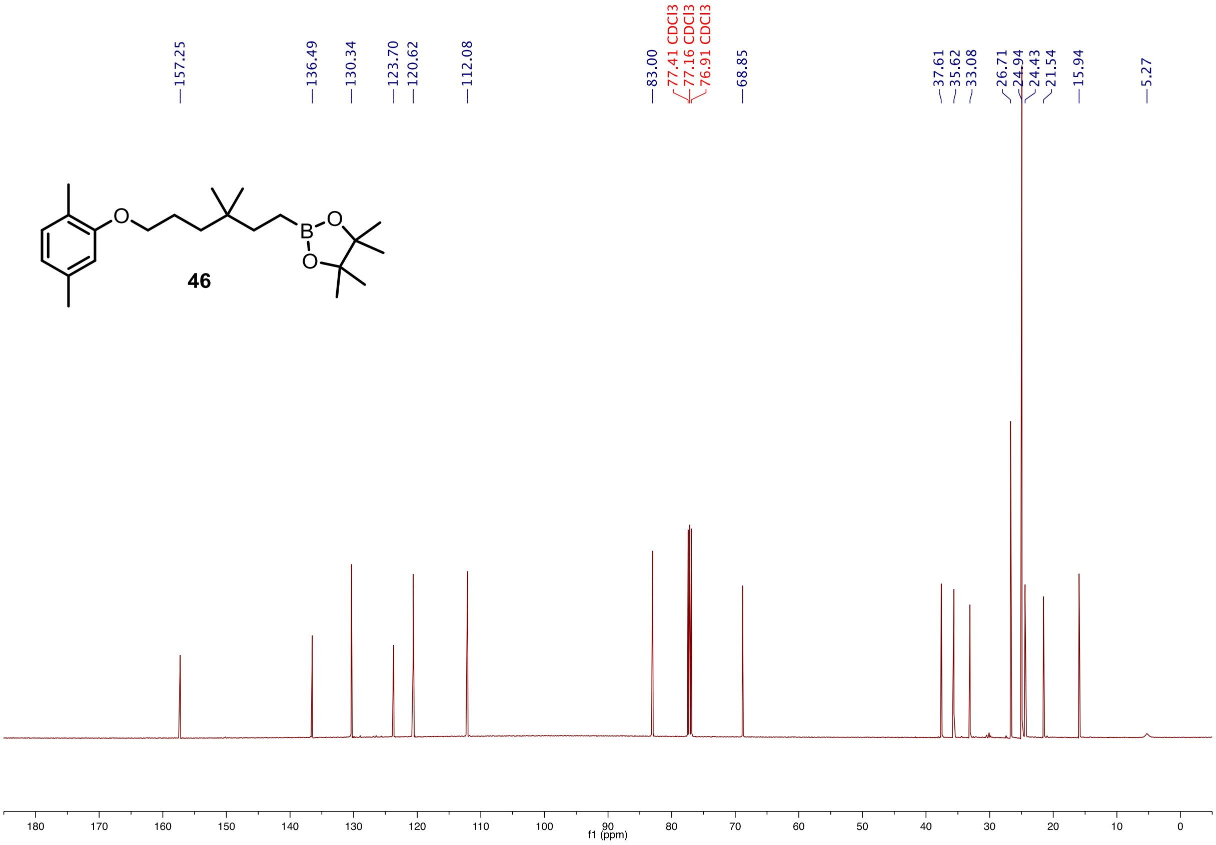

^{1}H NMR (500 MHz, $CDCl_3$)

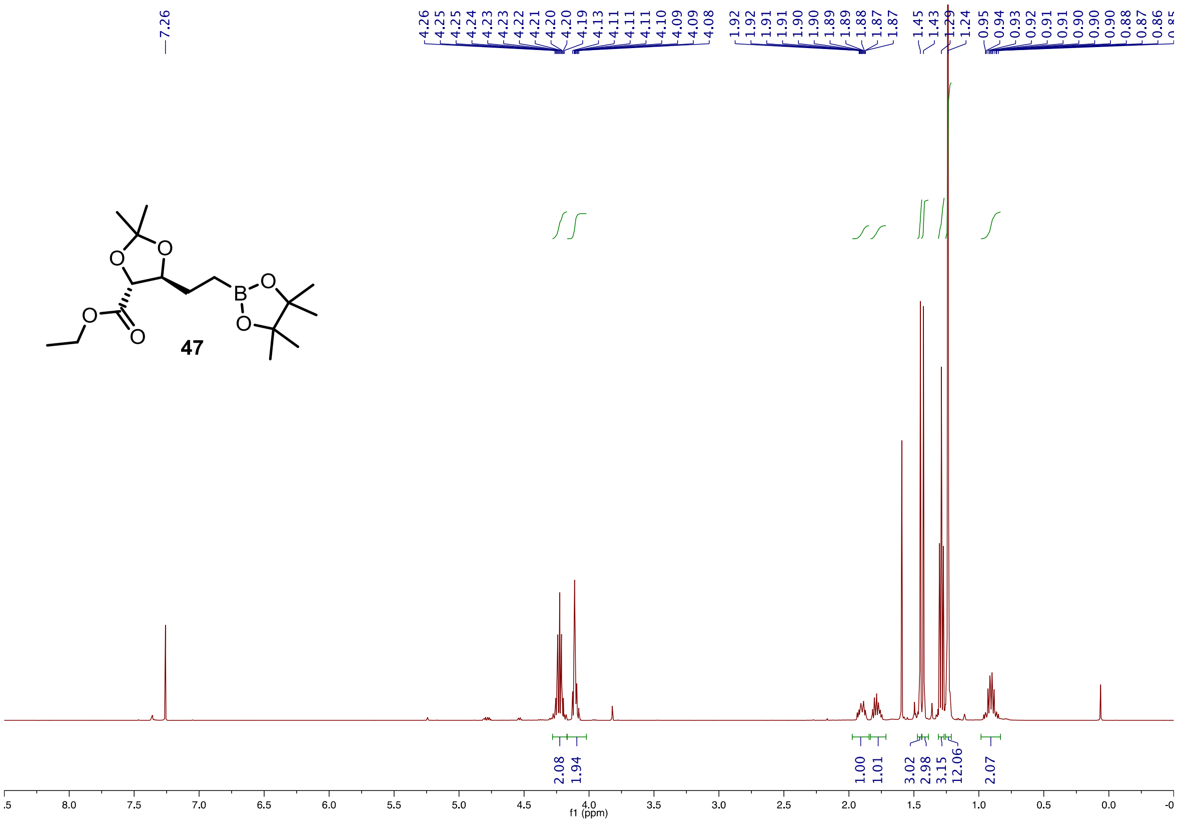

^{13}C NMR (126 MHz, $CDCl_3$)

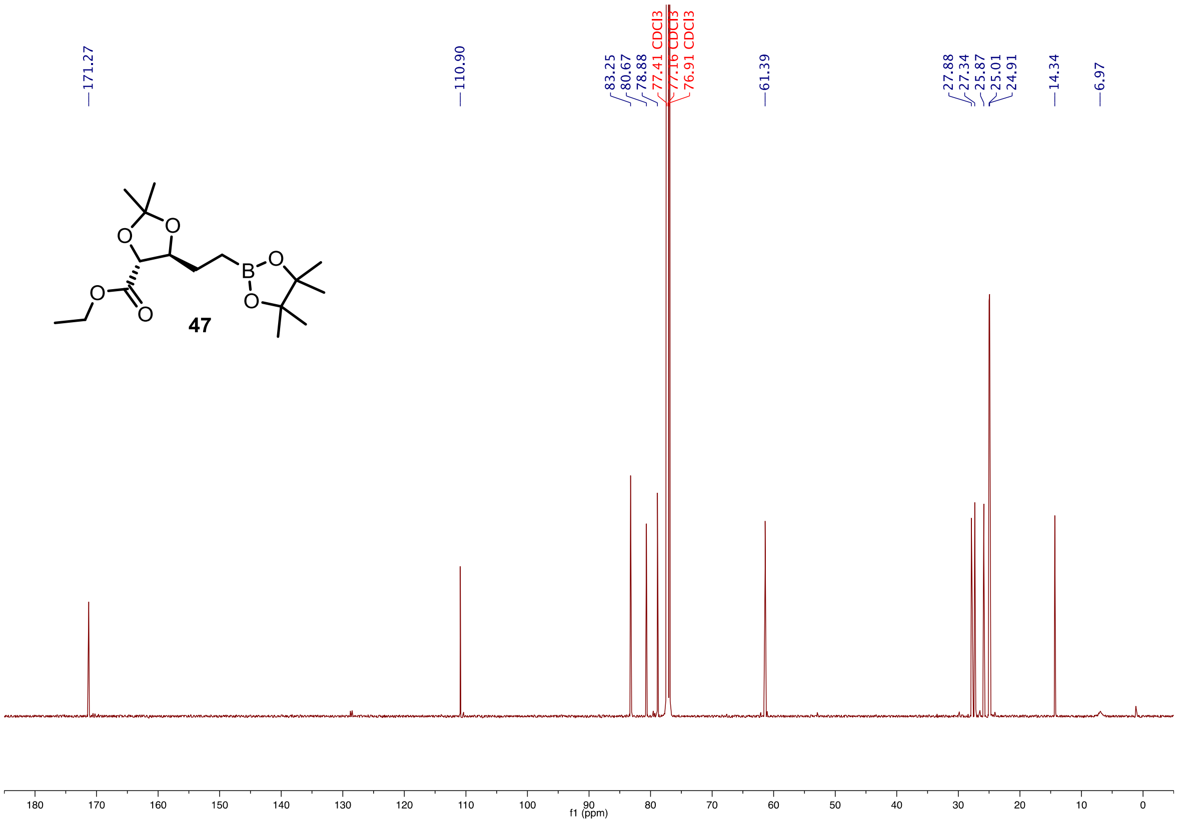

Supplement: Supplementary file 1 — Supplementary [file ANIE-57-2155-s001.pdf]
